# Targeting Acute Myelogenous Leukemia Using Potent Human Dihydroorotate Dehydrogenase Inhibitors Based on the 2-Hydroxypyrazolo[1,5-*a*]pyridine Scaffold: SAR of the Biphenyl Moiety

**DOI:** 10.1021/acs.jmedchem.0c01549

**Published:** 2021-04-12

**Authors:** Stefano Sainas, Marta Giorgis, Paola Circosta, Valentina Gaidano, Davide Bonanni, Agnese C. Pippione, Renzo Bagnati, Alice Passoni, Yaqi Qiu, Carina Florina Cojocaru, Barbara Canepa, Alessandro Bona, Barbara Rolando, Mariia Mishina, Cristina Ramondetti, Barbara Buccinnà, Marco Piccinini, Mohammad Houshmand, Alessandro Cignetti, Enrico Giraudo, Salam Al-Karadaghi, Donatella Boschi, Giuseppe Saglio, Marco L. Lolli

**Affiliations:** † Department of Drug Science and Technology, University of Turin, Via P. Giuria 9, Turin 10125, Italy; ‡ Department of Clinical and Biological Sciences, University of Turin, Regione Gonzole 10, Orbassano, Turin 10043, Italy; § Molecular Biotechnology Center, University of Turin, Via Nizza 52, Turin 10126, Italy; ∥ Division of Hematology, AO SS Antonio e Biagio e Cesare Arrigo, Via Venezia 16, Alessandria 15121, Italy; ⊥ Department of Environmental Health Sciences, Istituto di Ricerche Farmacologiche Mario Negri IRCCS, Via Mario Negri 2, Milano 20156, Italy; # Laboratory of Tumor Microenvironment, Candiolo Cancer Institute, FPO, IRCCS, Candiolo, Strada Provinciale, 142-KM 3.95, Candiolo, Turin 10060, Italy; ∇ Higher Education Mega Center, Institutes for Life Sciences, South China University of Technology, Guangzhou 510641, China; ● Gem Forlab srl, Via Ribes, 5, Colleretto Giacosa, Turin 10010, Italy; △ Gem Chimica srl, Via Maestri del Lavoro, 25, Busca, Cuneo 12022, Italy; × Department of Oncology, University of Turin, Via Michelangelo 27/B, Turin 10125, Italy; ¶ Department of Biochemistry and Structural Biology, Lund University, Naturvetarvägen 14, Box 124, Lund 221 00, Sweden; ◆ Division of Hematology and Cell Therapy, AO Ordine Mauriziano, Largo Filippo Turati, 62, Turin 10128, Italy

## Abstract

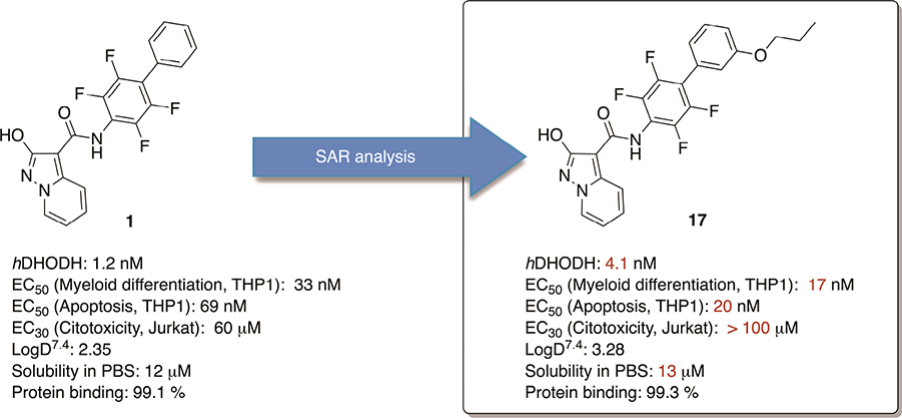

The connection with acute myelogenous leukemia (AML) of dihydroorotate dehydrogenase (*h*DHODH), a key enzyme in pyrimidine biosynthesis, has attracted significant interest from pharma as a possible AML therapeutic target. We recently discovered compound **1**, a potent *h*DHODH inhibitor (IC_50_ = 1.2 nM), able to induce myeloid differentiation in AML cell lines (THP1) in the low nM range (EC_50_ = 32.8 nM) superior to brequinar’s phase I/II clinical trial (EC_50_ = 265 nM). Herein, we investigate the **1** drug-like properties observing good metabolic stability and no toxic profile when administered at doses of 10 and 25 mg/kg every 3 days for 5 weeks (Balb/c mice). Moreover, in order to identify a backup compound, we investigate the SAR of this class of compounds. Inside the series, **17** is characterized by higher potency in inducing myeloid differentiation (EC_50_ = 17.3 nM), strong proapoptotic properties (EC_50_ = 20.2 nM), and low cytotoxicity toward non-AML cells (EC_30_(Jurkat) > 100 μM).

## Introduction

Human dihydroorotate dehydrogenase (*h*DHODH, EC 1.3.99.11) is a flavin-dependent enzyme that plays a fundamental role in *de novo* pyrimidine biosynthesis. In humans, class 2 DHODH is anchored at the inner mitochondrial leaflet where it enzymatically catalyzes the oxidation of dihydroorotate to orotate by involving cofactor flavin mononucleotide (FMN). In order to regenerate FMN, a second redox reaction occurs with coenzyme Q (ubiquinone), which is recruited from the inner mitochondrial membrane and is a key player in the mitochondrial electron transport chain (ETC).^1^

*h*DHODH has been validated as a therapeutic target in diseases that involve wide cellular proliferation, such as autoimmune diseases and cancer.^2−4^ Small molecules that can interfere with *h*DHODH enzymatic activity by targeting the host’s pyrimidine synthesis may also show great potential in reducing viral replication against a broad spectrum of viruses.^5,6^*h*DHODH was initially included in the list of therapeutic options to be tested against SARS-CoV-2 infected cells.^7^ It was then validated as a target for COVID-19^8,9^ and became one of the most interesting therapeutic options for this disease.^10−14^

It is quite recent the discovery^15,16^ that *h*DHODH is also involved in regulating myeloid differentiation in AML; this has opened new scenarios for possible treatments of the disease. As the most common acute leukemia in adults, AML affects the myeloid lineage of white blood cells; if left untreated, it is typically fatal within weeks or months, while current chemotherapies give an over-five-year survival rate of only around 25%. By remaining blocked in an immature form and so losing the ability to differentiate into adult white blood cells, the leukemic blast accumulates in the bone marrow and interferes with the production of normal blood cells. The mechanism that associates *h*DHODH inhibition with myeloid differentiation had not been fully understood.^2,17^ However, the effect seems to be strictly connected to the depletion of pyrimidine biosynthesis being rescued by the presence of excesses uridine,^18^ which bypasses the requirement for *de novo* pyrimidine synthesis by feeding the salvage pathway. This concept was explained well by Sykes et al.,^19^ who were the first to suggest that AML cells, unlike non-leukemia cells, may be particularly sensitive to “pyrimidine starvation” and choose differentiation over self-renewal. This scenario realistically opens the possibility of expanding, to all AML types, the developments in M3 subclass acute promyelocytic leukemia (APL), whose clinical management was completely transformed by the introduction of a differentiation therapy that was based on *all-trans*-retinoic acid (ATRA), in association with a proapoptotic agent (chemotherapy or arsenic trioxide);^20−22^ APL is currently curable in up to 90% of cases.^23^ Looking to the future, using the same powerful treatment strategy for non-APL AML, i.e., forcing differentiation and apoptosis using a *h*DHODH inhibitor, for example, may increase AML-patient survival rates.

**Chart 1:**
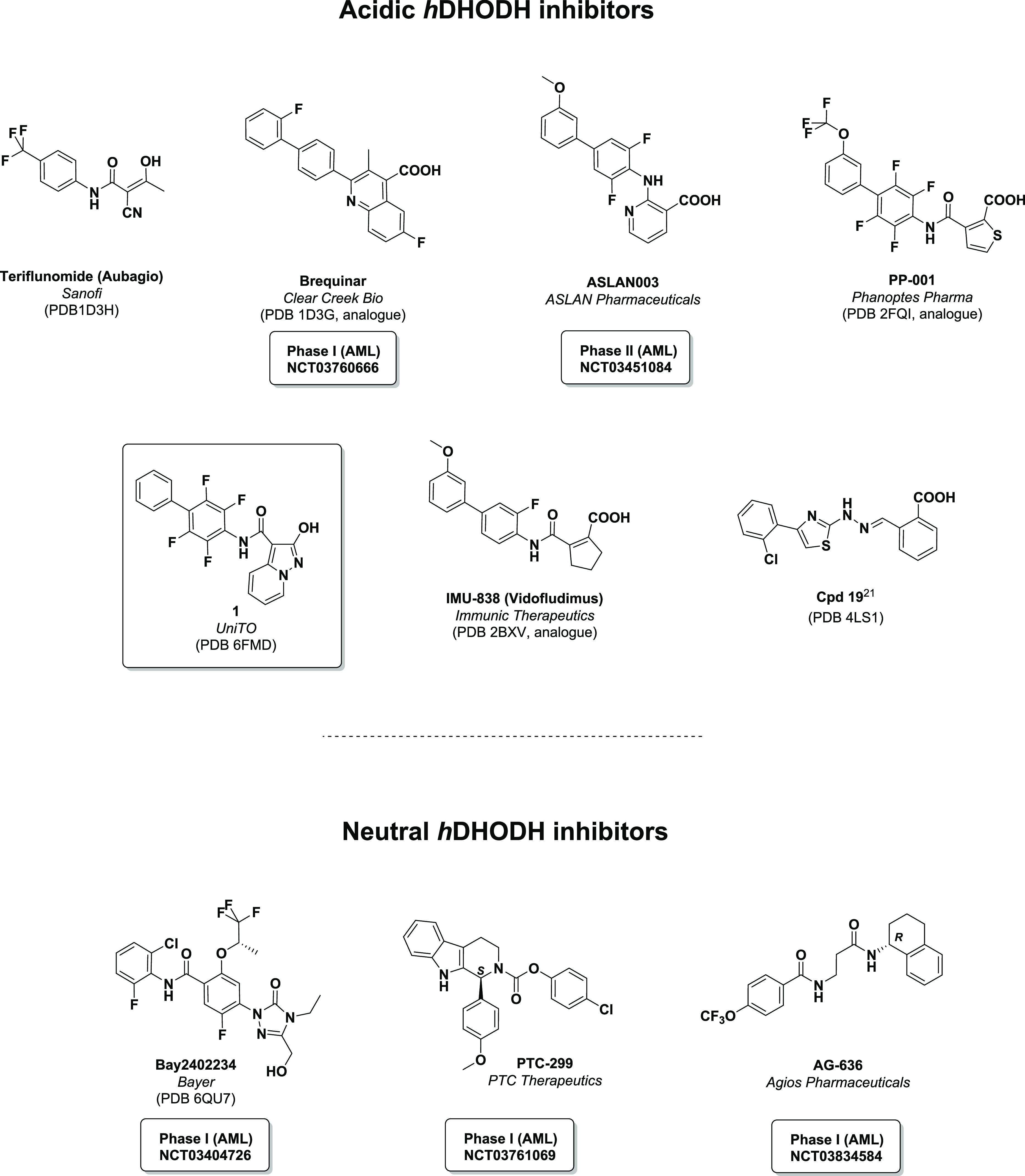
Present Landscape of the Most Potent *h*DHODH Inhibitorsa

Five major companies are currently running phase I/II AML clinical trials with new generation *h*DHODH inhibitors and brequinar (Chart 1).^3^

a Where available, the PDB code of the inhibitor–*h*DHODH complex in the Protein Data Bank is indicated in parentheses as well as the current AML related clinical trials.

Brequinar, designed by Du Pont in 1985,^24^ is one of the most potent *h*DHODH inhibitors discovered to date. Despite showing clear *in vitro* anticancer properties, it had never been tested in AML until November 2018, when Clear Creek Bio, who acquired it the previous year from Bristol Myers Squibb, obtained an active IND for the study of brequinar in relapsed/refractory AML. Brequinar is currently in a phase I/II clinical trial for the treatment of patients with relapsed/refractory AML (NCT03760666). A second acid DHODH inhibitor in a clinical trial, in addition to brequinar, is ASLAN003 (ASLAN Pharmaceuticals), which is currently being evaluated in a phase IIa clinical trial in AML patients (NCT03451084).^25^ Li et al. have designed, in a hit-to-lead process, a series of benzylidenehydrazinyl-substituted thiazoles of which “**cpd 19**” was the most potent.^26^ Although still in the preclinical stage, **cpd 19** is comparable to brequinar, at the enzymatic level, in presenting notable antiarthritic efficacy and acceptable pharmacokinetic profiles *in vivo*. On the other hand, there is a small group of neutral inhibitors in trials: PTC-299 (PTC Pharmaceuticals, phase I since Oct 29, 2018, NCT03761069), AG 636 (Agios Pharmaceuticals, phase I since Feb 18, 2019, NCT03834584), and Bayer’s BAY-2402234 (BY, in phase I clinical trials since January 2018, NCT03404726). Recently, the pharmacochemical properties of this latter have been well detailed by Christian et al.^27^

In 2018, the authors, developing a modulation of hydroxyazole scaffolds,^28,29^ discovered compound **1**,^18^ (Chart 1) and took it as being representative of a novel class of *h*DHODH inhibitors that are based on an unusual carboxylic group bioisostere, 2-hydroxypyrazolo[1,5-*a*]pyridine, which is effectively able to mimic the interactions of brequinar carboxylate in the ubiquinone binding site.^18,29^ As it demonstrates comparable potency to brequinar itself on the enzymatic level (IC_50_ of 1.2 nM vs 1.8 nM, respectively), **1** was found to induce myeloid differentiation in AML cell lines (THP1) in the low nM range (EC_50_ = 32.8 nM), which is around one log digit superior to the phase I candidate brequinar (EC_50_ = 265 nM).

In this work, we move forward from that discovery in two directions. On one hand, we have thoroughly explored the structure–activity relationships (SARs) of this class of compounds and have attempted to provide analogues with better potency and drug-like profiles. On the other, we continue the investigation into the drug-like properties of **1**, in particular, its *in vitro* metabolism and *in vivo* toxicity, in order to evaluate whether it may be a suitable candidate for future *in vivo* testing. The data obtained during the study, which were supported by *in silico* design based on the crystallographic poses of **1**, as well as by extensive biochemical and physicochemical characterization, have been compared with those of clinical trial leads (brequinar and BAY-2402234). Moreover, we have also compared the apoptotic, differentiating, and cytotoxic properties of the synthesized compounds in AML cell lines.

## Results and Discussion

### Target-Compounds Design

Since the ligands designed herein must be able to reach the inner mitochondrial leaflet, where *h*DHODH is located,^30^ lipophilicity plays a central role in the translation of *h*DHODH enzymatic activity into a substantial effect on cells. While developing **1**, we observed how the log *P* and log *D* of compounds were correlated to the potency of their differentiation effect in AML cell lines. For instance, two acidic inhibitors with comparable IC_50_ values, such as **1** and brequinar, but with different log *D* values (2.35 and 1.83, respectively) have different effects; **1** induced myeloid differentiation at a concentration that was 1 log lower than that of brequinar. Also other researchers recently identify LogD^7.4^ as a crucial parameter in the design of *h*DHODH inhibitors owing cell efficacy.^31^ As higher lipophilicity is usually associated with reduced solubility and adverse ADME, we directed the further modulation of **1** to the identification of the favored modulation able to obtain the excellent balance between the compound’s lipophilicity and solubility to achieve the desired cellular effect while also retaining high enzymatic inhibition activity.

The cocrystal structure of *h*DHODH in complex with **1** (PDB code 6FMD) provided insight into the ligand-binding mode and was used to support the *in silico* studies. Similarly to brequinar, **1** effectively binds the so-called “lipophilic patch”,^30^ which is the pathway followed by ubiquinone (coenzyme Q) to reach FMN. Acidic hydroxypyrazolo[1,5-*a*]pyridine forms an ion bridge with the side chain of Arg136 and a hydrogen bond interaction with Gln47. The pyridine moiety extends toward *subsites 3* and *4*, fitting between Val134 and Val143. The last interaction with *h*DHODH occurs in the lipophilic channel defined as subsite 1, within the tetrafluorobiphenylic scaffold, with residues Met43, Leu42, Leu46, Ala59, Phe62, Phe98, Leu68, Leu359, and Pro364.

**Figure 1:**
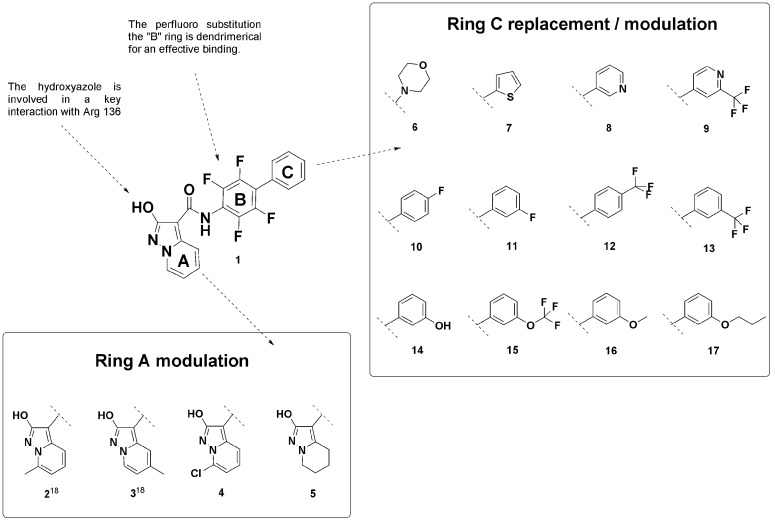
Compounds involved in the SAR exploration.

Figure 1 shows the three different portions of the compound **1** structure (rings A, B, and C), which were the subject of this SAR study, as well as the designed analogues (compounds **2**–**17**). The SAR of the pyridine moiety (A ring), started in a previous study,^18^ showed that position C7 best tolerated the substitution of the hydrogen. Compounds **4** and **5** were designed to complete the investigation of the A ring and the interaction with subsite 4. Moving on the biphenyl scaffold, the SAR of this substructure was already partially investigated inside the development of other hydroxyazole analogues, and on the basis of the SAR-transfer concept,^32^ we can assume that analogues with a biphenylic scaffold share similar SAR, since X-ray structures clearly show superimposable binding modes.^29^ In particular, because it was highlighted that the complete saturation of the B ring with fluorine is fundamental to maintaining high binding affinity,^28,29,33^ the tetrafluorophenyl scaffold (B ring) was retained in the development of compound **1** analogues.

**Figure 2:**
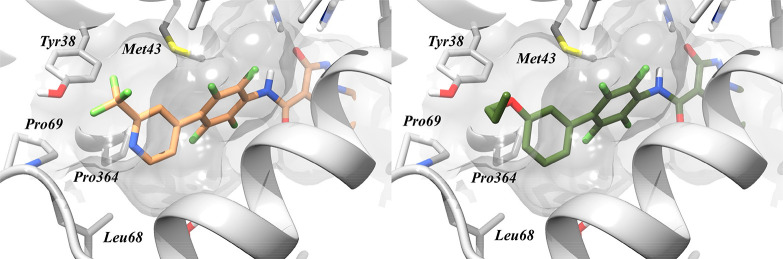
View of the entrance of the ubiquinone binding site, with the predicted binding mode of **9**, on the left, and **17** on the right. The protein structure used for the docking simulation has PDB code 6FMD.

Moving now to the outer second ring (C ring), previous SAR studies carried out on **1** analogues have identified in this position possible opportunities for modulation.^28^ The binding mode of **1** and its derivatives places the C ring next to the entrance of the ubiquinone binding pocket, where the phenyl is mainly involved in hydrophobic interactions with Phe62, Pro364, Leu68, and Tyr38 (Figure 2).

As shown in Figure 2, substituents in the *meta* position are located in an empty area on the border between the pocket and the vacuum while the *para* substituents are considered to crash with the lipophilic residue of subsite 1. Earlier studies^28,29,33^ already identified the *meta* substitution as the most tolerated by the binding pocket; the solvent exposure of this modulation might lead to marginal effects on inhibitor activity, and in this sense, the *meta* position may be quite strategic for the development of *h*DHODH inhibitors with increased lipophilicity that retain low nM enzymatic activity profiles.

In this occasion, with the purpose to reinforce the SAR of this part of the structure, first we designed four compounds (**6**–**9**) to investigate the possible phenyl bioisosteric replacement of the C ring. In the following, as guided by *in silico* methodologies (Table S1, docking scores), we designed eight compounds (**10**–**17**) to investigate the effect of different lipophilic substitutions in the *meta* and *para* positions of the phenyl ring. We left out the modulation of the *ortho* position in order to leave unchanged the optimal dihedral angle between rings B and C obtained in brequinar as well as in **1**, as explored by Bonomo et al. with *ortho* substituents.^33^

### *h*DHODH Inhibitory Activity and SAR

We evaluated the recombinant *h*DHODH inhibition activity of compounds **4**–**17** using two clinical-trial candidates (brequinar and BAY-2402234) and **1** as comparisons. While BAY-2402234 was purchased from a commercial source, brequinar was synthesized following known procedures. In order to complete the scenario and prepare the discussion of the following cell-based studies, LogD^7.4^, solubility in PBS, and protein binding were also measured for each compound.

### SAR Analysis of the A Ring

As reported in our previous publication,^18^ the interaction between **1** and the small lipophilic pocket created by Val134 and Val143 (subsite 4) was explored using molecular dynamics (MD) free energy perturbation (FEP),^34^ as a possible source of further modulation. Of the four sites on the A ring (positions 4–7), *in silico* analyses suggested that position 7 is the most profitable for hydrogen substitution. Moreover, the study indicated that chlorine derivatives were generally preferred over methyl ones. Moving to experimental work, taking into account the MD/FEP results, a derivative with a chlorine substituent in position 7 (**4**, IC_50_ = 3.4 nM) was synthesized. Compared to the methyl analogue (**2**, IC_50_ = 4.3 nM), the chlorine is better tolerated, leading to an analogue of **1** with comparable activity but higher LogD^7.4^ (Table 1). We therefore also considered a reduced 4,5,6,7-tetrahydropyrazolo[1,5-*a*]pyridine analogue, **5** (IC_50_ = 5.8 nM), which gave a slight decrease in potency compared to **1**.

**Table 1:** Enzymatic Inhibitor Activity of Compounds **2**–**5**, Brequinar, Bay2402234, ASLAN003, and **1**, with relative LogD^7.4^, Solubility, and Protein Binding^a^

compd	*h*DHODH, IC_50_ ± SE (nM)	LogD^7.4^ ± SDc	solubility (μM) in PBS	protein binding (% bond)
Brequinar	1.8 ± 0.3	1.83 ± 0.02	229	98.83
BAY-2402234	6.0 ± 0.6 (1.2 from lit.^27^)	2.7^27^	<1^31^	90.1^27^
ASLAN003	35^25^	nd	nd	>99^25^
**1**	1.2 ± 0.2	2.35 ± 0.02	12	99.10
**2**	4.3 ± 0.5	2.70 ± 0.02	<LOD	nd
**3**	35 ± 3	2.47 ± 0.09	<LOD	nd
**4**	3.4 ± 0.5	2.81 ± 0.13	<LOD	nd
**5**	5.8 ± 0.4	2.36 ± 0.02	<LOD	nd

a The effect of the compounds is expressed as IC_50_ values. Limit of detection (LOD): 6 μM. The “nd” notation indicates that the compound was not tested in that specific assay.

While the A ring modulations did not result in increased inhibitory activity compared to **1**, higher LogD^7.4^ values were observed in all compounds but were unfortunately all associated with reduced solubility. Solubility in PBS was not measured, as the concentration of the soluble fraction was below the LOD value (6 μM). In terms of protein binding, any significant improvement was observed.

### SAR Analysis of the C Ring: Phenyl Replacement/Modulation

Moving to the C ring, we assigned the first four compounds (**6**–**9**) to the investigation of its possible isosteric replacement (Table 2).

**Table 2:** Enzymatic Inhibitor Activity of Compounds **6**–**17** and Relative LogD^7.4^, Solubility, and Protein Binding^a^

compd	*h*DHODH, IC_50_ ± SE (nM)	LogD^7.4^ ± SDc	solubility (μM) in PBS	protein binding (% bond)
**6**	90.9 ± 13.1	0.66 ± 0.08	438	nd
**7**	1.35 ± 0.45	nd	nd	nd
**8**	6.23 ± 0.63	0.98 ± 0.03	47.3	99.58
**9**	150 ± 15	1.84 ± 0.06	20.2	nd
**10**	17.7 ± 3.30	insoluble	<LOD	nd
**11**	2.03 ± 0.44	2.09 ± 0.04	<LOD	99.96
**12**	71.8 ± 9.42	insoluble	<LOD	nd
**13**	6.34 ± 0.63	2.69 ± 0.03	<LOD	99.94
**14**	2.78 ± 0.32	1.82 ± 0.09	55.3	nd
**15**	2.30 ± 0.33	3.27 ± 0.19	8.1	100
**16**	2.75 ± 0.31	2.46 ± 0.04	74.3	99.95
**17**	4.09 ± 0.62	3.28 ± 0.12	12.9	100

a The effect of the compounds is expressed as IC_50_ values. Limit of detection (LOD): 6 μM. The “nd” notation indicates that the compound was not tested in that specific assay.

The incorporation of a morpholine substituent (**6**, IC_50_ = 90.9 nM) was not well tolerated, as a phenyl ring, and resulted in around a 50-fold potency decrease compared to **1**. The introduction of heteroatoms that may interact with the lipophilic subpocket, composed of Pro69 and Leu68, may induce repulsive interactions, as the potency decrease is also observed for pyridine derivate **9** (Figure 2). However, **6** was the most soluble of the series, showing almost twice the solubility of brequinar. The replacement of the C ring with classical bioisostere thiophen (**7**, IC_50_ = 1.35 nM) retained the inhibitory profile. The optimal LogD^7.4^ range for optimal drug absorption, via the phenomena of passive permeability or diffusion, is considered to be in the range between 1 and 3.^35^ In the case of *h*DHODH inhibitors, the literature indicates an optimal LogD^7.4^ value superior to 2.50 reduced adsorption issue.^31^ In terms of activity, the replacement of the phenyl position of **1** with a classic isostere nitrogen, as in **8** and **9**, resulted in losses of activity (IC_50_ = 6.23 nM and 150 nM), as *meta* replacement is better tolerated. To better understand this result, **9** must be compared with **13** (IC_50_ = 6.34 nM) in which the −CF_3_ in the *meta* position is still present, but the nitrogen is ideally removed. The two pyridine analogues **8** and **9** display better solubility than **1**, 4 and 1.5 times, respectively. In terms of protein binding, any significant improvement was observed.

Moving on, we investigated the positions on the C ring that are suitable for substitution in compounds **10**–**17**. The binding mode of **1** and derivatives places the C ring next to the entrance of the ubiquinone binding pocket (Figure 2), exposing the *meta* position to an empty area of the binding site, on the border between the pocket and the vacuum. With **10**–**13**, we investigated the effect of different lipophilic substitutions, such as F and CF_3_, in the *para* (**10**, **12**) and *meta* (**11**, **13**) positions of the C ring. Analyzing the results (Table 2), it can be observed how *meta* replacement, **11** and **13** with IC_50_ = 2.03 nM and IC_50_ = 6.34 nM, respectively, was better tolerated than the *para* isomers, **10** and **12** with IC_50_ = 17.7 nM and IC_50_ = 71.8 nM. While finding the activity of **11** to be in the same range as **1** is not surprising, as the fluorine is a classical proton bioisosteric replacement, this cannot be said for **13**, for which small lipophilic groups, such as −CF_3_, are well accepted. This replacement validated the predicted binding mode of **13**, in which a trifluoromethyl is placed in an empty area of the binding site. These modulations resulted in compounds being more lipophilic, as expected, but unfortunately, this property is associated with insolubility, and these values are largely below the reference limit of 6 μM. Focusing on substitution on the *meta* position, we obtained compound **14** (IC_50_ = 2.78 nM), which is comparable to **1** itself in terms of potency but characterized by better solubility (around 5 times), as the oxygen atom is able to form hydrogen bonds with water, and has a LogD^7.4^ comparable with that of brequinar itself. By ideally modulating **14**, we introduced substitution to the phenolic oxygen, giving **15**–**17**. This modulation resulted in an IC_50_ that is comparable to that of **1**, proving the predicted binding mode again, and was associated with an increase in LogD^7.4^ for each compound. The most interesting compounds are **15** and **17** (IC_50_ = 2.30 and 4.09 nM), which are characterized by the introduction of an alkyloxy group. These are the most interesting compounds in the series here described, as they are comparable to the lead **1** in terms of potency although showing similar solubility but higher LogD^7.4^ (above the 2.5 threshold).

### Cell-Based Assays: Differentiation, Apoptosis, and Cytotoxicity

**Figure 3:**
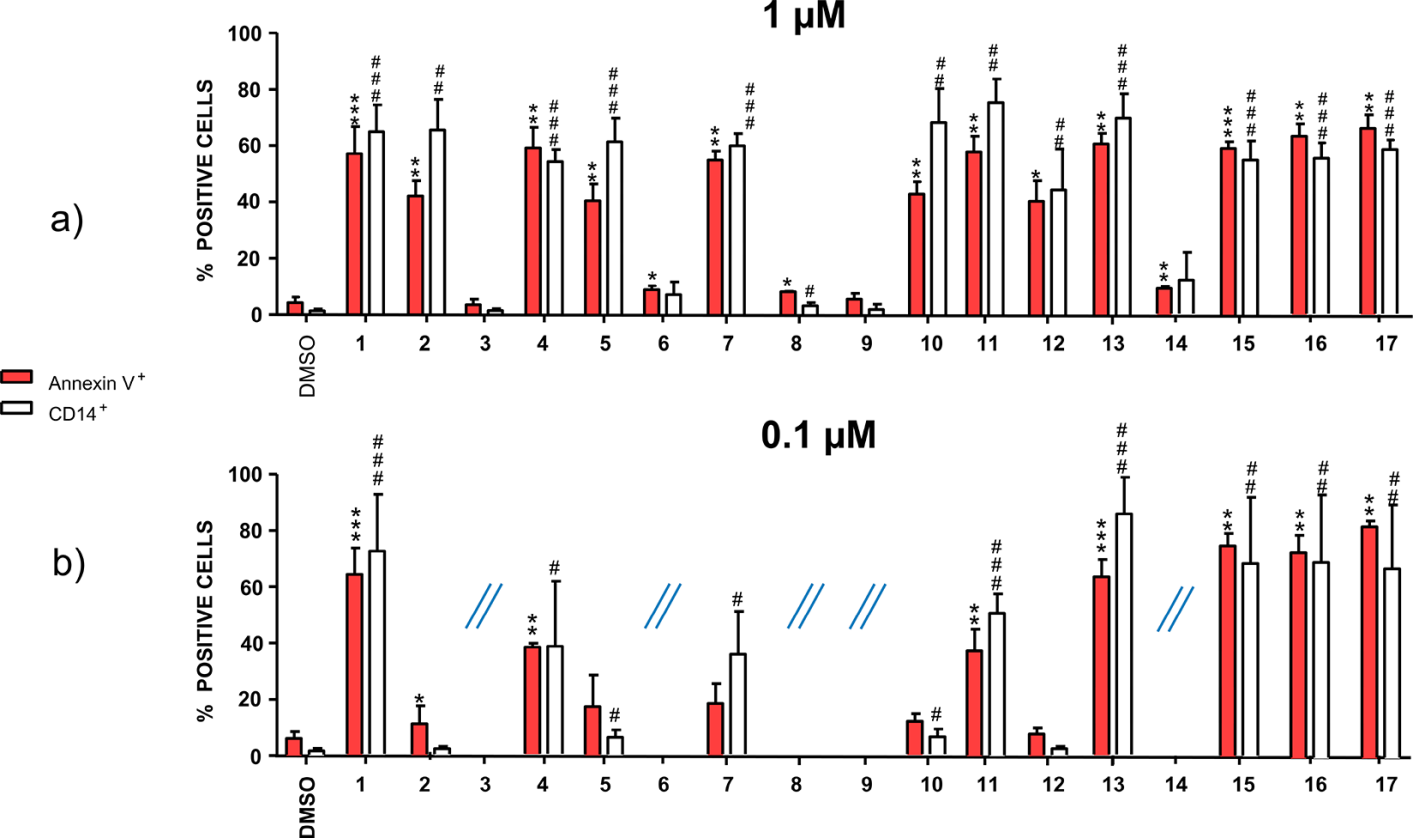
Differentiation (CD14 expression, white histogram) and apoptosis (annexin V expression, red histogram) as induced by inhibitors **1**–**17** at 1 μM (a) and 0.1 μM (b) in THP1 cells. DMSO (dimethyl sulfoxide) acts as the negative control group as it was used to solubilize *h*DHODH inhibitors. ∗, ∗∗, ∗∗∗ represent the statistical significance for apoptosis (respectively *p* < 0.05, < 0.01, and <0.001). #, ##, and ### represent the statistical significance for differentiation (respectively *p* < 0.05, <0.01, and <0.001). The statistical significance is calculated by comparing the compounds to DMSO.

Several research groups have observed that *h*DHODH inhibitors can induce differentiation and apoptosis in multiple AML models.^15,18,27^ An ideal *h*DHODH inhibitor should work at low concentrations in AML cells but should be nontoxic against non-AML cells or at least only be toxic at high concentrations. This would guarantee strong specificity against AML, minimizing systemic toxicity. In order to assess the biological activities of the new *h*DHODH inhibitors discussed above, we evaluated their ability to induce differentiation and apoptosis in the THP1 and U937 AML cell lines and their cytotoxic effects on non-AML cells. The differentiation process was tracked by analyzing CD14 or CD11b expression, as these antigens are typically present respectively in THP1 or U937 mature myeloid cells; the apoptotic rate was assessed with annexin V, whose expression indicates the beginning of the apoptotic process. We performed a preliminary selection by treating only THP1 cells with *h*DHODH inhibitors at 1.0 μM (Figure 3a), and the most promising molecules were then challenged in a new experiment at 0.1 μM (Figure 3b). The best compounds were finally characterized in detail, their EC_50_ values for differentiation and apoptosis in both THP1 and U937 were assessed, their toxicity profiles on non-AML cells were evaluated, and their performance was compared to that of brequinar and BAY-2402234 (Table 3).

**Table 3:** Analysis of the Biological Activity (Enzymatic Inhibitor Activity, Differentiation, Apoptosis, and Cytotoxicity) of Compounds **1**, **11**, **13**, **15**–**17** on THP-1 and U937, Compared to Brequinar, BAY-2402234, and **1**^a^

compd	*h*DHODH, IC_50_ ± SE (nM)	differentiation EC_50_ THP1 (μM) (CL 95%)	apoptosis EC_50_ THP1 (μM) (CL 95%)	differentiation EC_50_ U937 (μM) (CL 95%)	apoptosis EC_50_ U937 (μM) (CL 95%)	cytoxicity Jurkat (μM) (effect ≥30% ± SD)
Brequinar	1.8 ± 0.3	0.2486 (0.1326–0.4658)	0.2640 (0.1659–0.4213)	0.1886 (0.1045–0.3431)	0.3222 (0.1302–0.7971)	48 ± 1^18^
BAY-2402234	6.0 ± 0.6 (1.2 from lit.^27^)	0.0024 (0.0013–0.0044)	0.0034 (0.0020–0.0058)	nd	nd	36 ± 4
**1**	1.2 ± 0.2	0.0397 (0.0206–0.0766)	0.0723 (0.0418–0.124)	0.0260 (0.005649–0.1037)	0.0404 (0.0239–0.0684)	60 ± 1^18^
**11**	2.03 ± 0.44	0.0676 (0.0468–0.0974)	0.2504 (0.1754–0.3596)	0.0407 (0.0191–0.1169)	0.0682 (0.0132–0.0563)	39 ± 3
**13**	6.34 ± 0.63	0.0383 (0.0225–0.0651)	0.0511 (0.0316–0.0823)	0.0342 (0.0175–0.0677)	0.0600 (0.0092–0.432)	>100 μM
**15**	2.30 ± 0.33	0.0315 (0.0173–0.0574)	0.0396 (0.0251–0.0639)	0.0086 (0.0011–0.0826)	0.0353 (0.0185–0.0679)	>100 μM
**16**	2.75 ± 0.31	0.0312 (0.0164–0.0594)	0.0379 (0.0175–0.0903)	0.0311 (0.0124–0.0834)	0.0428 (0.0125–0.1497)	68 ± 7
**17**	4.09 ± 0.62	0.0173 (0.0113–0.0173)	0.0202 (0.0131–0.0312)	0.0046 (0.0013–0.0350)	0.0167 (0.0073–0.0396)	>100 μM

a The differentiation and apoptotic data are expressed as EC_50_, and the cytotoxic effect was determined as the concentration that induced cytotoxicity in more than 30% of the Jurkat cells. The “nd” notation indicates that the EC_50_ and cytotoxicity of the compound were not determined.

First of all, we can observe that apoptosis and differentiation are substantially associated, with no compound inducing just one phenomenon or the other. This suggests that they are both the consequence of the same mechanism, i.e., pyrimidine starvation. The performance of these new *h*DHODH inhibitors confirms our preliminary hypothesis. As observed earlier, *h*DHODH inhibitors with comparable IC_50_ at the enzymatic level can have different effects on cells, depending on their log *D*. For example, **1** (IC_50_ = 1.2 nM, LogD^7.4^ = 2.35) can induce differentiation at a concentration that is 1 log lower than possible with brequinar (IC_50_ = 1.8 nM, LogD^7.4^ = 1.83). The close correlation between cell-based potency and LogD^7.4^ in *h*DHODH inhibitors has also been underlined by Gradl et al. In particular, they observed that, in a series of BAY-2402234 analogues, clogD values lower than 2.5 led to compounds with low cellular activity, probably due to insufficient lipophilicity.^31^

Compounds **6**, **8**, **9**, and **14** confirmed this phenomenon. In fact, despite potently inhibiting *h*DHODH at the enzymatic level, they were basically inactive in cells even at 1 μM. It is possible, in fact, that the low LogD^7.4^ of these compounds prevented them from reaching the target deep inside the second mitochondrial leaflet. We have also previously observed that one-digit nM enzymatic inhibition IC_50_ values are usually needed to observe potent myeloid differentiation.^18^ This could explain the inactivity of **3** at 1 μM (Figure 3a).

When challenged at 0.1 μM (Figure 3b), only five compounds (**11**, **13**, **15**, **16**, and **17**) besides **1** were still highly active. Interestingly, they were all characterized by *meta* substitution, confirming again how substituents in *meta* position are favored compared to the *para* analogues.

**Figure 4:**
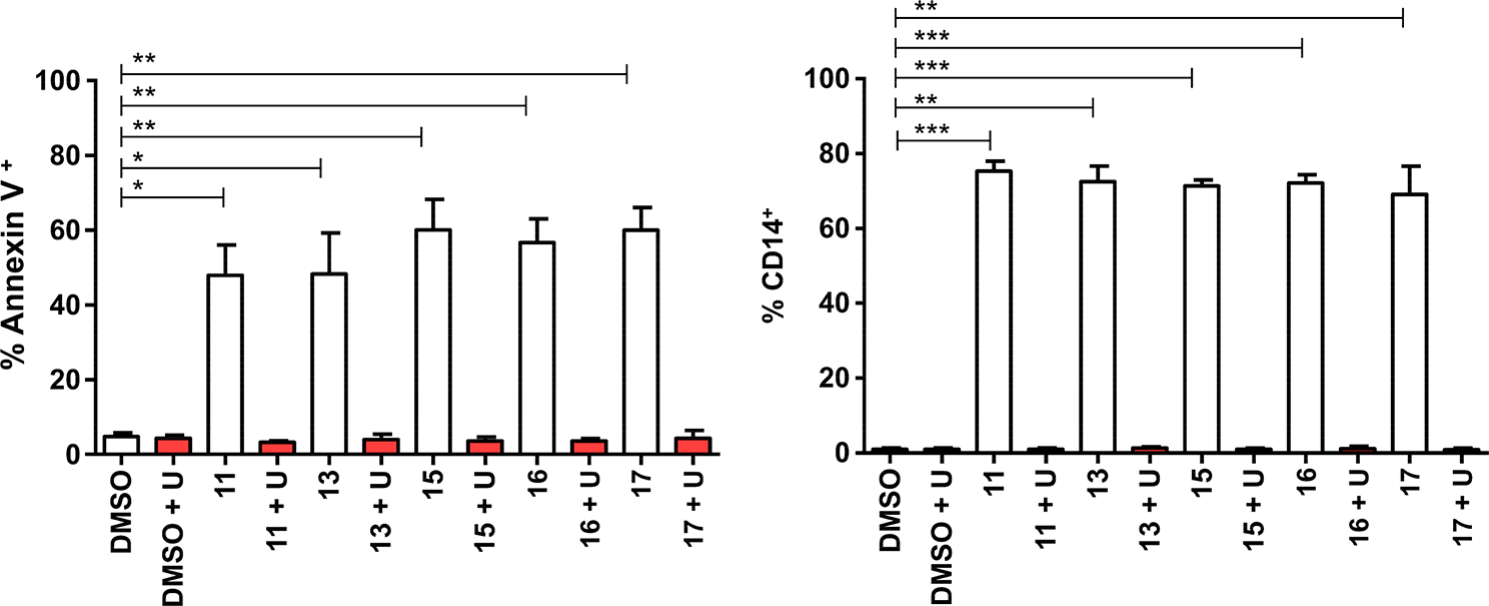
Differentiation (CD14 expression, left panel) and apoptosis (annexin V expression, right panel) induced by inhibitors **11**, **13**, **15**, **16**, **17** at 1 μM with and without uridine at 100 μM. ∗, ∗∗, ∗∗∗ represent the statistical significance for apoptosis (respectively *p* < 0.5, <0.01, and <0.001). The statistical significance is calculated by comparing the compounds to DMSO.

In order to demonstrate that apoptosis and differentiation were indeed caused by pyrimidine depletion rather than off-target effects, experiments with the best five compounds in the series were repeated in the presence of uridine. As already mentioned, uridine is a downstream product of *h*DHODH and is basically the antidote to *h*DHODH inhibitors. Accordingly, when differentiation and apoptosis experiments were performed in the presence of uridine, the complete rescue of the phenomena was observed (Figure 4). AML is a very heterogeneous pathology, so in order to test the effects of selected compounds on other leukemic cells, differentiation and apoptosis assays were performed also on the U937 cell line. Table 3 shows that, of the selected compounds, **13** and **15**–**17** are at least as effective as the lead **1** in terms of myeloid differentiation and proapoptotic profile. In **11**, the introduction of the *m*-fluorine was unable to significantly increase the LogD^7.4^ and hence improve performances. The best results were obtained with compounds **15** and **17**, which were characterized by a differentiation EC_50_ (31.5 and 17.4 nM, respectively) and apoptosis EC_50_ (39.6 and 20.2 nM, respectively) in THP-1, **17** being superior to phase I/II brequinar of 14 and 13 times, respectively, on the same cell line. Moving to U937, more sensible to *h*DHODH inhibitors, **15** and **17** are been observed as both more effective (differentiation EC_50_ of 8.6 and 4.6 nM, respectively; apoptosis EC_50_ of 35.3 and 16.7 nM, respectively), **17** being able to improve its efficacy in comparison with brequinar reaching 41 times (differentiation) and 19 times (apoptosis). Importantly, the toxicity profiles of our compounds, and especially **15** and **17**, are extremely favorable and superior to those of **1**, brequinar, and BAY-2402234.

### *In Vitro* Metabolic Profiles of **1**, **15**, and **17**

Moving in parallel to the exploration of the SAR of this class of *h*DHODH inhibitors, we continued the investigation into the drug-like properties of **1**, the lead first-generation that displayed an optimal toxicity profile and was highly selective on-target,^18^ in order to evaluate whether it may be a suitable candidate for future *in vivo* testing. Moreover, we characterized also the *in vitro* metabolic profile of compounds **15** and **17**, being the most interesting of the series. Here we characterize the major metabolic pathways responsible for the metabolism of compounds **1**, **15**, and **17***in vitro* using rat-liver microsomes and therefore move the *in vivo* evaluation forward. The *in vitro* metabolic profiles of compounds **1**, **15**, and **17** were assessed using the following combination of methods: (C) incubation at 37 °C with active rat-liver microsomes and a regenerating system that slowly generated coenzyme units over the incubation time, leading to a better reproduction of *in vivo* behavior; (C1) incubation at 37 °C with heat-inactivated microsomes (via a 10 min heating cycle at 90 °C) and a regenerating system; (C2) incubation at 37 °C with microsomes without a regenerating system; and finally, (B) incubation with the blank medium. SyGMa (systematic generation of potential metabolites) software, a tool that lists predicted metabolites with associated empirical probability scores, was used to identify putative metabolites, which were then investigated by analyzing samples with liquid chromatography coupled to high-resolution mass spectrometry (HPLC–HRMS). For each series of samples (C, C1, and C2), incubation was stopped after 15, 30, 60 min and after 120 min (*t* = 120). The full-scan MS data acquired for all of the samples were analyzed to find the *m*/*z* values of the predicted molecular structures. In order to exclude interfering signals, the results obtained were compared to blank samples and common background peaks were not considered. In sample C, we found for compound **1** peaks whose accurate mass data were in accordance with the monohydroxylated, dihydroxylated, and methoxylated metabolites (Table 4) and for compound **17** peaks in accordance with monohydroxylated and dealkylated metabolites (Table 5). For compound **15**, we did not identify appreciable concentration of metabolites, proving the metabolic stability of the OCF_3_ moiety. Moreover, as expected, we did not identify the same metabolites in samples C1 and C2, confirming the fundamental role of CYP450 in phase I metabolism.

**Table 4:**
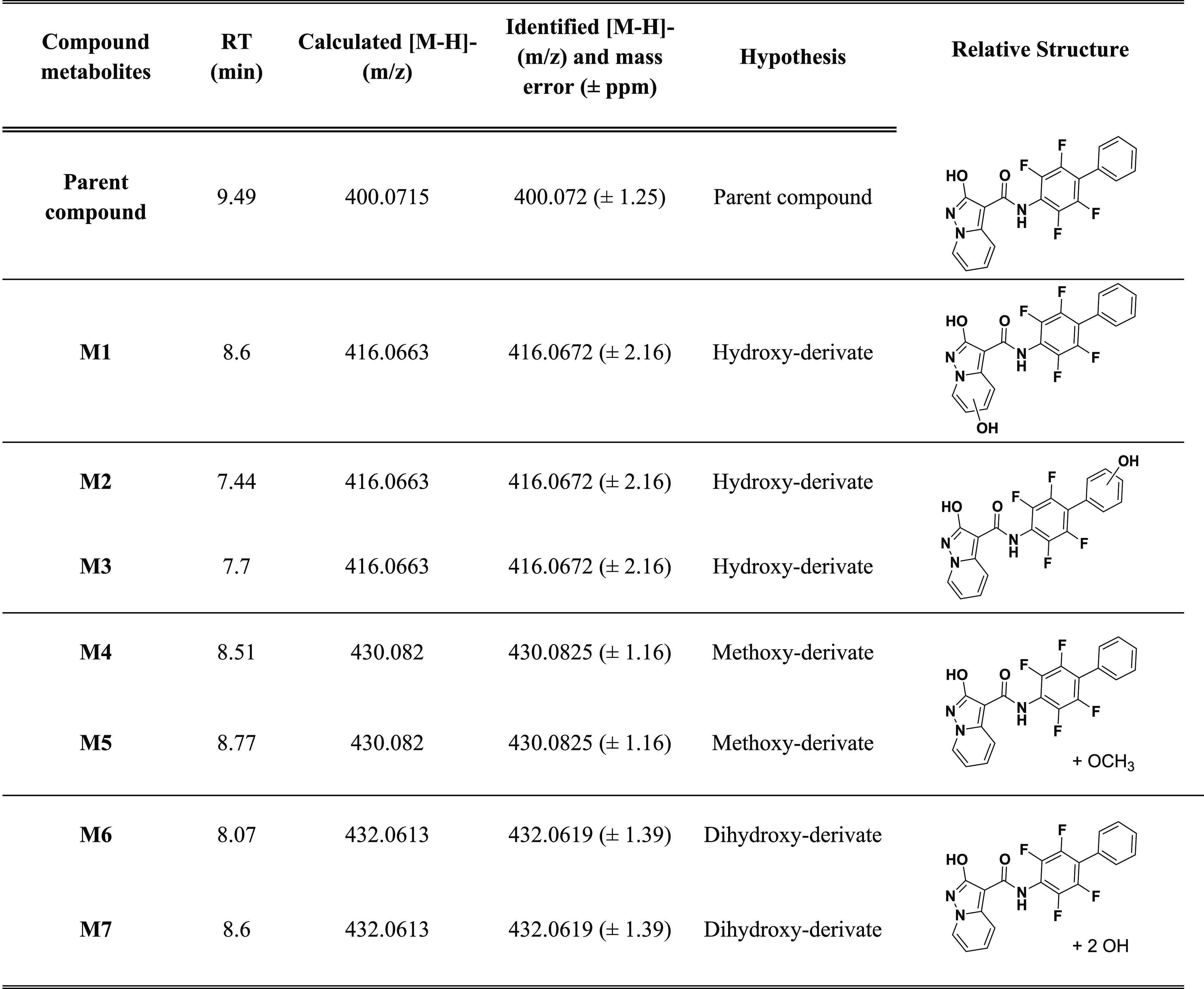
List of Metabolites of **1**, with Chromatographic Retention Times, Calculated Accurate Masses (*m*/*z* M – H^–^), Identified Accurate Masses (*m*/*z* M – H^–^) in Samples, Chemical Formulas, and Structures

**Table 5:**
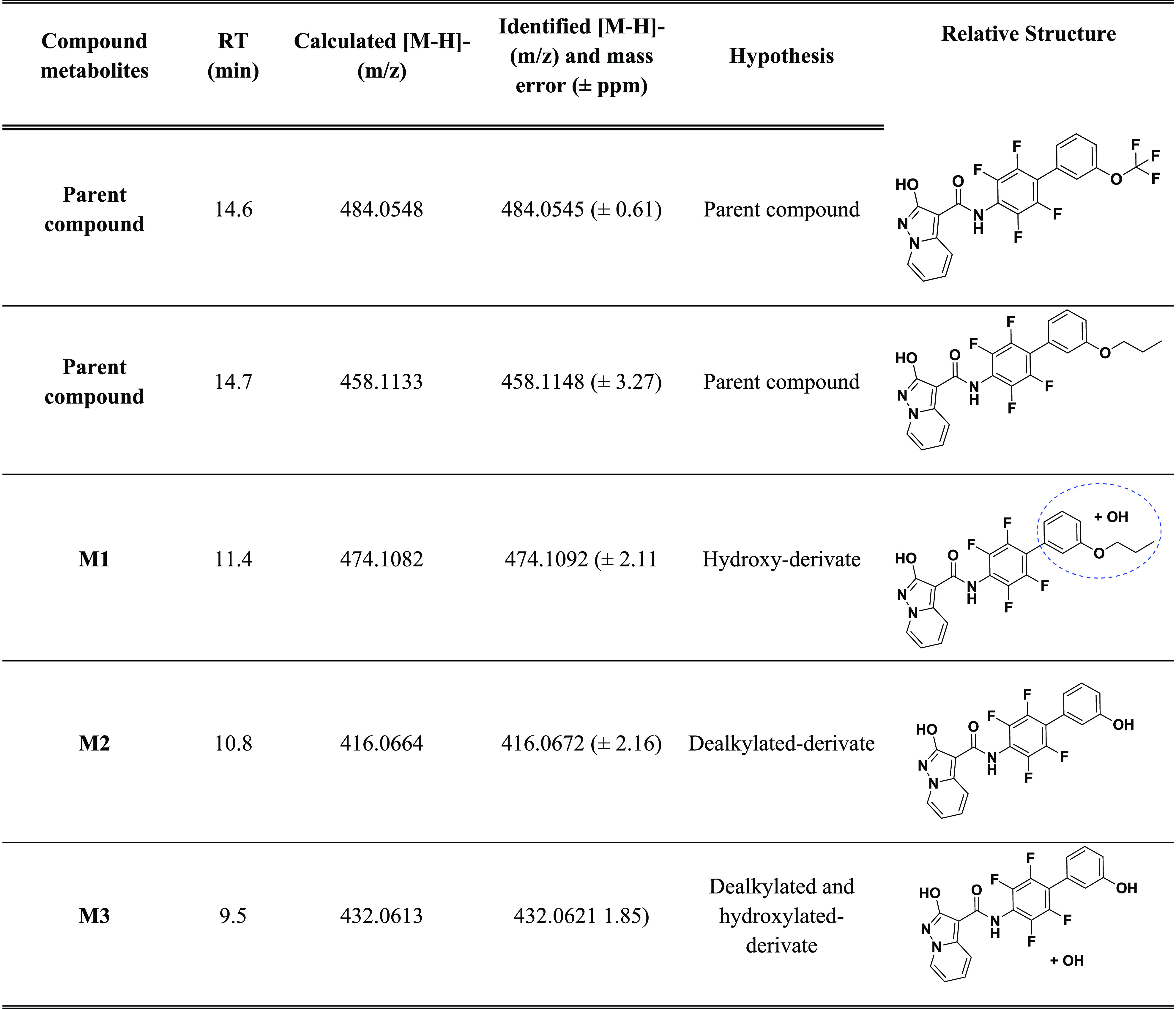
List of Metabolites of **15** and **17**, with Chromatographic Retention Times, Calculated Accurate Masses (*m*/*z* M – H^–^), Identified Accurate Masses (*m*/*z* M – H^–^) in Samples, Chemical Formulas, and Structures

**Figure 5:**
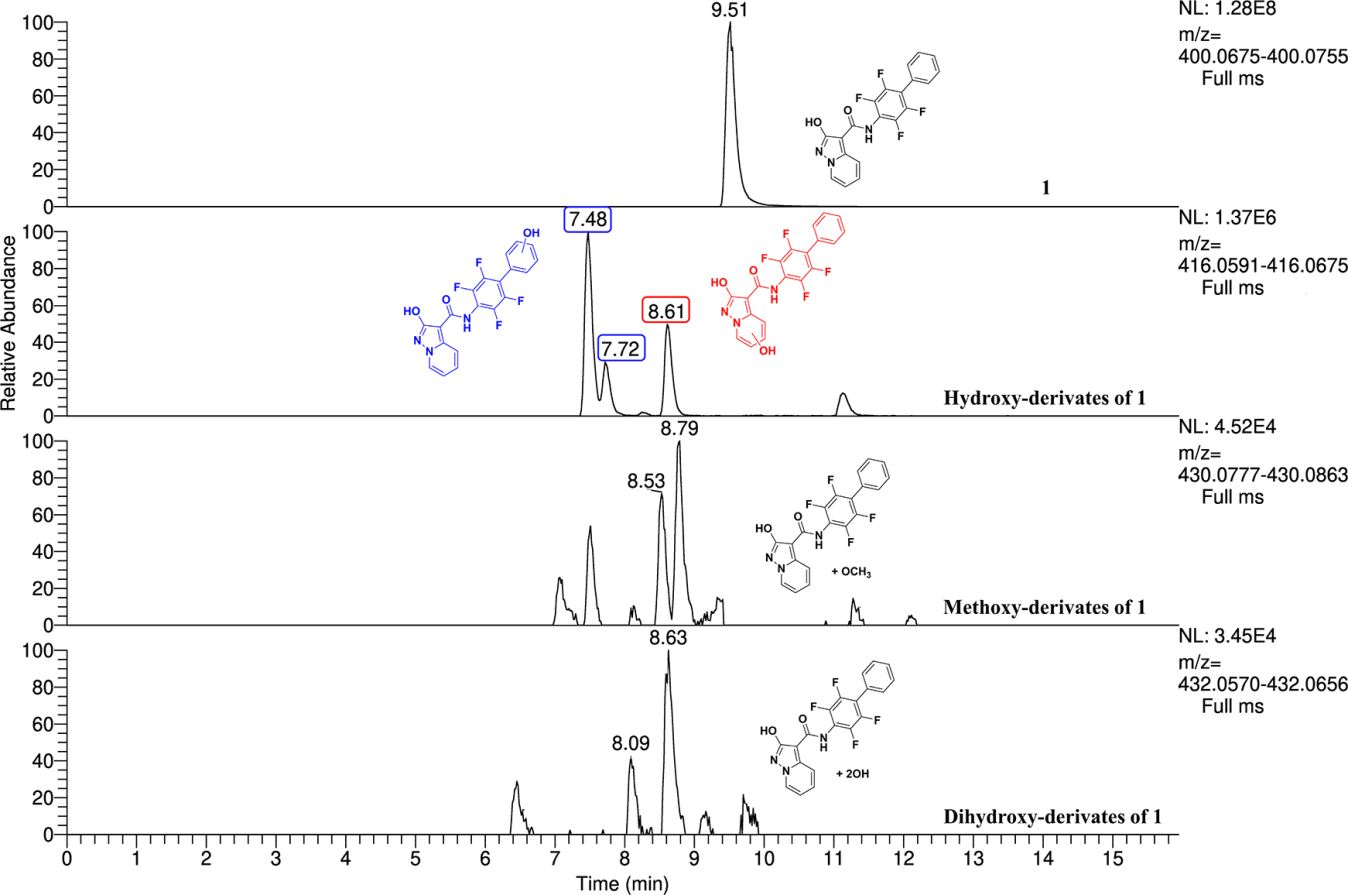
Extracted ion chromatograms of identified metabolites of **1** in sample C after incubation (time-point 2 h).

**Figure 6:**
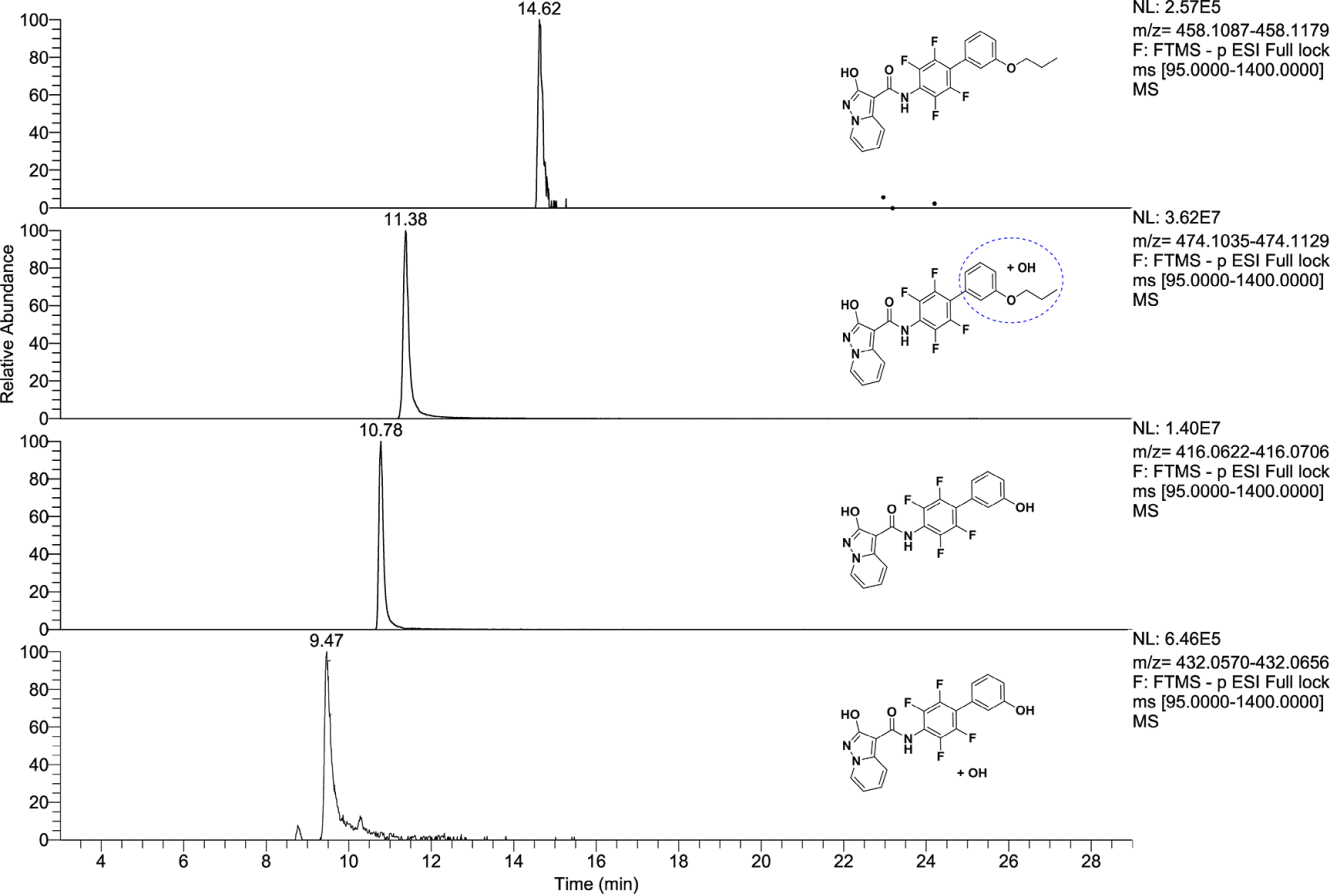
Extracted ion chromatograms of identified metabolites of **17** in sample C after incubation (time-point 2 h).

In order to confirm the presence and the chemical structures of the metabolites, a second set of experiments, based on the MS2 fragmentation of selected peaks (MS2-DIA analysis), was performed. In this way, we confirmed the structures of **1** and its metabolites that were found in sample C by interpreting the MS and MS2 spectra of each chromatographic run. Following the criteria proposed by Schymanski et al.,^36^ the metabolites identified are to be considered as “probable structures” (level 2b) or “tentative candidates” (level 3). Figures 5 and 6 report extracted ion chromatograms for the putative metabolites. The different retention times of the hydroxy and methoxy derivatives of **1** indicate that there may have been modifications to different parts of the molecule.

The interpretation of the fragmentation spectra allowed the metabolites with hydroxyl substitution on the pyrimidine ring and on the phenyl ring to be distinguished (Figure S1). The high-resolution MS2 spectra revealed mutually exclusive ions and were thus capable of distinguishing metabolites that originated from the same precursor ion. For instance, for hydroxylated metabolites of compound **1** (precursor ion *m*/*z* 416.0672), we found the fragment ions at *m*/*z* 256.0391 when the −OH group was on the phenyl ring (ring C) but at *m*/*z* 240.0442 when the hydroxylation was on the pyrimidine ring (ring A). However, the MS2 spectra could not provide information on the exact ring position of hydroxylation. The same analysis was performed also for hydroxylated metabolites of compound **17** (precursor ion *m*/*z* 474.1082); we found the fragment ion at *m*/*z* 314.08 confirming the −OH group was on the C phenyl ring or on the propyl chain. We can hypothesize the hydroxylation on propyl chain for the presence of the fragment ion at *m*/*z* 255.03 corresponding to the dealkylated fragment. However, the MS2 spectra could not provide information on the exact position of hydroxylation for M2 of **17**.

An examination of the results for compounds **1** and **15**, which had undergone P-450-mediated biotransformation for an incubation period of 2 h, highlighted that compounds **1** and **15** are metabolically stable and 98% of compound **1** and 100% of compound **15** were recovered, while for compound **17** a fast metabolism was observed, and after 30 min the parent compound was no longer present. We can conclude that the strategy that allowed reaching to **17** is effective to obtain potent cell-based effective *h*DHODH inhibitors without losing solubility, as **15** if compared to **1**. However, because for compound **17** a metabolic weakness was observed, it must be improved in this sense.

### *In Vivo* Toxicity Profile of **1**

As at the moment compound **1** has the best balance between cell activity and drug-like properties, we choose it to start *in vivo* studies. We decided to assess its potential toxic effects and adopted a similar administration schedule as reported for brequinar in Sykes et al.;^15^ Balb/c mice were treated with 10 and 25 mg/kg of the drug (every 3 days, via intraperitoneal injection, ip) for 35 days. First of all, none of the mice in the various treatment groups died during the trial and all the animals were alive at the end of the experiment. The mice were checked and weighed before treatment. As described in Figure S2, no statistically significant loss of weight was observed over time at both compound **1** concentrations up to the end of the treatment. In addition, food uptake was normal, and no differences were observed in the two treatment groups, compared to controls (Figure S3).

Finally, to better test whether compound **1** induced pathological changes in hematological profile and in kidney and liver function, we pooled the blood samples of the mice at the end of the treatments and, in collaboration with the Veterinary Analysis Laboratory (Turin, Italy), performed hematological profiling and the biochemical analysis of renal and hepatic function parameters. As indicated in Table S2, no differences in blood count and the kidney and liver tests were observed after 35 days of treatment in the mice at both 10 and 25 mg/kg of compound **1**, compared to controls. Taken together, these data demonstrate that compound **1** presents a low *in vivo* toxicity profile and could be a good candidate for future *in vivo* efficacy experiment.

## Chemistry

**Scheme 1:**
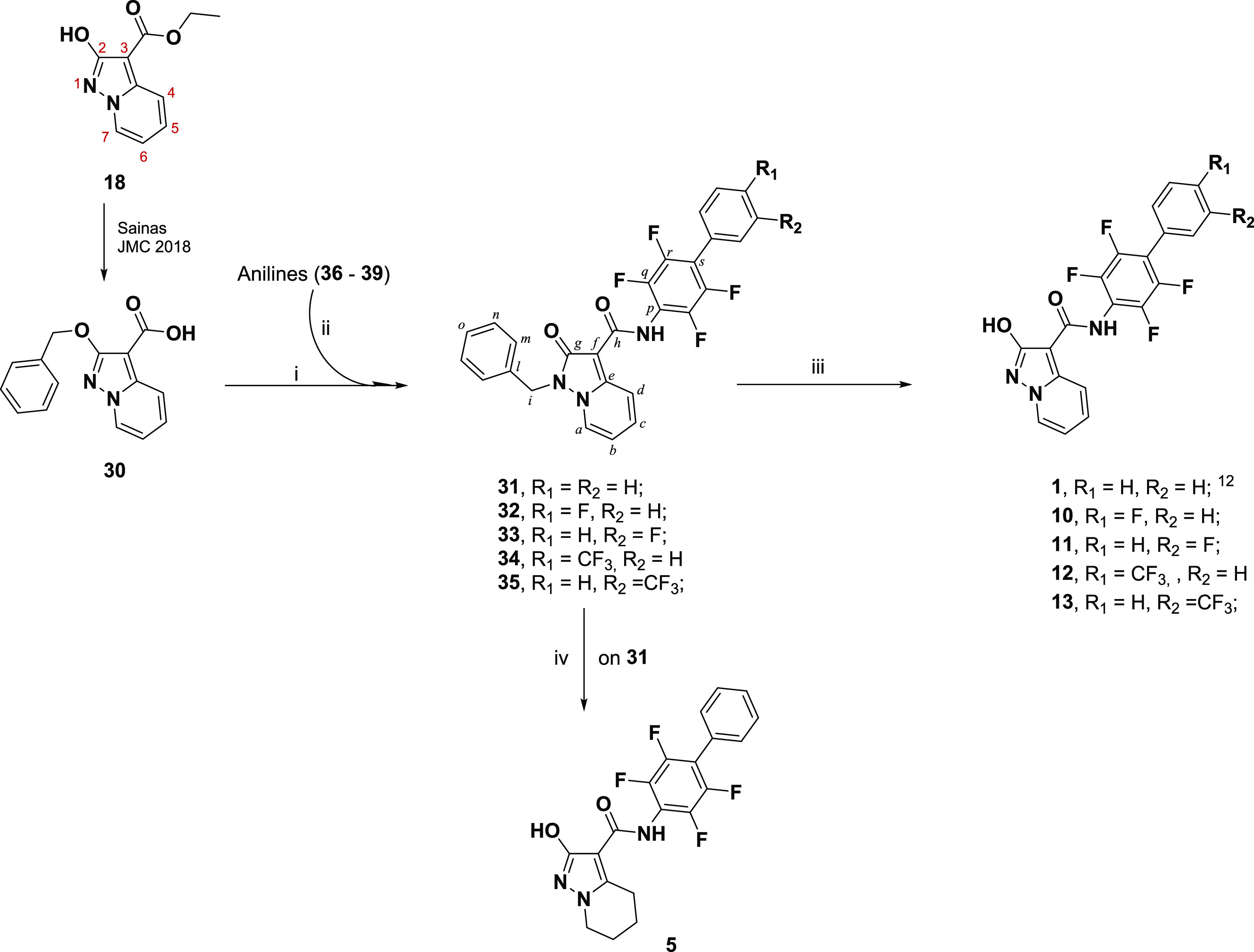
Synthetic Methodologies for the Synthesis of Targets **1**, **5**, **10**–**13** (i) Oxalyl chloride, dry DMF, dry THF; (ii) AlMe_3_, dry toluene, reflux; (iii) H_2_, Pd/C, 37% w/w HCl, ethanol; (iv) H_2_, Pd/C, dry THF, 40 bar, 65 °C, SynthWAVE.

For the syntheses of target compounds **5** and **10**–**13**, a chemical strategy, which had already been tested to obtain lead compound **1**, was used (see Scheme 1).^18^ The scheme starts from protected 2-hydroxypyrazolo[1,5-*a*]pyridine building block **30**, which is obtained from **18** in two steps.^18^ From **30**, the corresponding acyl chloride was obtained and used directly without further purification. Due to their poor reactivity with acyl chloride, each aniline (**36**–**39**; a detailed description of the synthetic methodologies for the functionalized aniline used in this manuscript has been included in the Supporting Information) was converted into the more reactive dimethylaluminum amide, which was reacted with the above acyl chloride to give the desired amides **31**–**35** in a 31–40% yield range. Note how, during the coupling step, the benzylic protecting group transferred from the exocyclic oxygen to the endocyclic N1 nitrogen in the pyrazolo[1,5-*a*]pyridine system. During the synthesis of **1**,^18^ the removal of the benzyloxy moiety from **31** via hydrogenation was always impacted by the presence of a side reaction that led to traces of reduced compound **5**. On this occasion, **5** was obtained in a 44% yield by applying stronger catalytic hydrogenation conditions (40 bar) and using SynthWAVE apparatus. On the other hand, to avoid such side reactions, compounds **32**–**35** were converted to the desired target compounds **10**–**13** by applying room-pressure catalytic hydrogenation in the presence of 1.0 equiv of 37% w/w HCl.

**Scheme 2:**
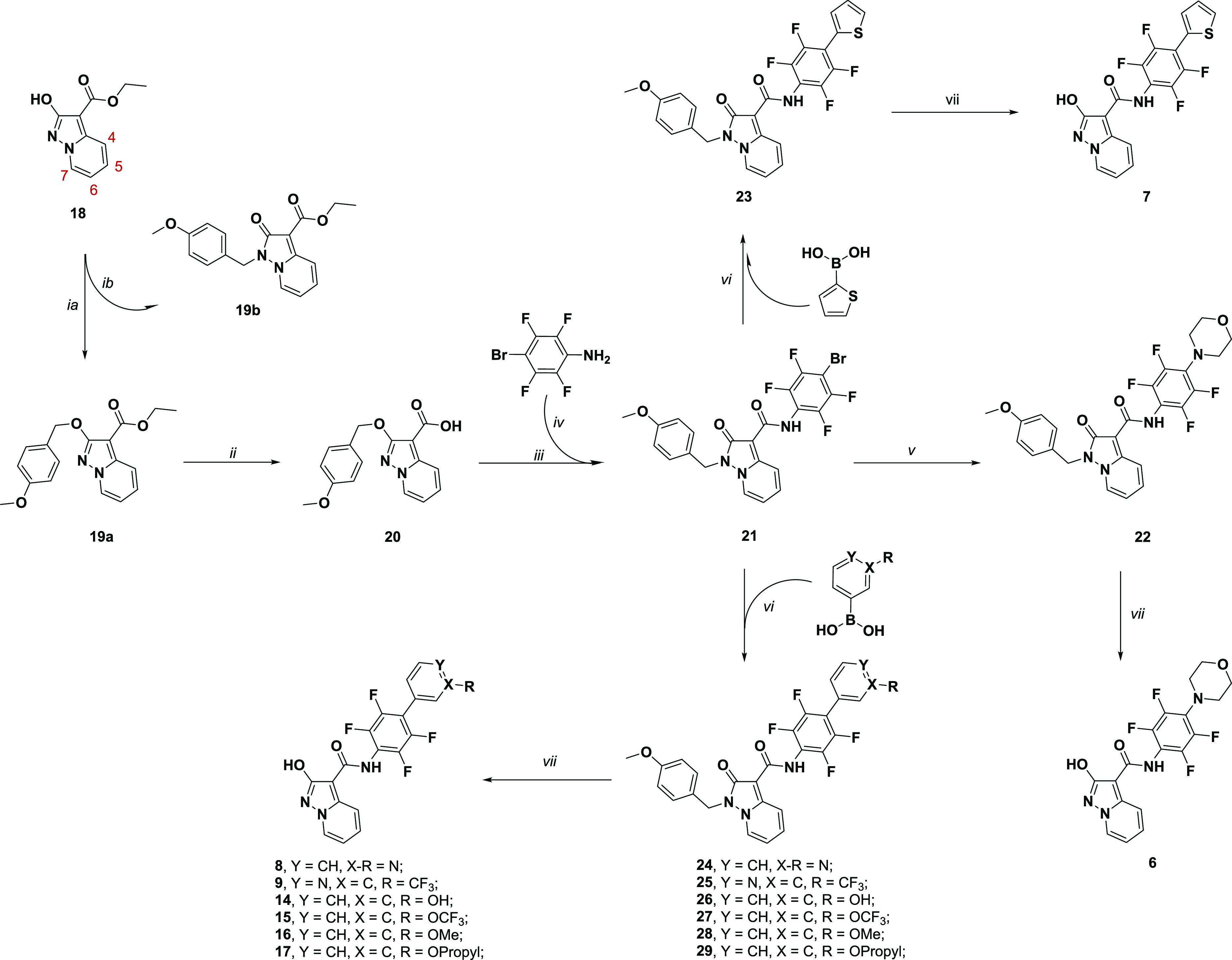
Synthetic Methodologies for the Synthesis of Targets **6**–**9**, **14**–**17** (i) (a) Cs_2_CO_3_, 4-MeOBnBr, dry DMF; (b) flash chromatography; (ii) (a) 5 M NaOH, ethanol, 75 °C; (b) HCl 2M, rt; (iii) nitrogen atmosphere, oxalyl chloride, dry DMF, dry THF; (iv) AlMe_3_, dry toluene, reflux; (v) nitrogen atmosphere, morpholine, Cs_2_CO_3_, Pd(OAc)_2_, BINAP, dry toluene, sealed tube, 110°C; (vi) (a) Pd(Ph_3_)_4_, K_2_CO_3_, dioxane/water (9:1 v/v), 1 h rt; (b) corresponding boronic acid, reflux; (vii) thioanisole, trifluoroacetic acid, 70 °C.

For the synthesis of compounds **6**–**9** and **14**–**17**, we designed a more convenient synthetic approach that made use of the late-step Suzuki coupling of compound **21**, as a common intermediate. Once again, the scheme started from 2-hydroxypyrazolo[1,5-*a*]pyridine **18**, which was protected by a 4-methoxybenzylic group. We exchanged the benzylic protecting group with a 4-methoxybenzylic one as it can be easily removed in acidic conditions, which are also applicable to molecules containing sulfur atoms and pyridine rings, both known to poison metal catalysts during hydrogenation.^37^ The reaction with 4-methoxybenzyl bromide afforded a mixture of the regioisomers **19a** and **19b** at a ratio of 61% and 27%, respectively. The mixture was resolved by flash chromatography, and the structure characterization of each isomer was attributed using the benzylic ^13^C chemical shift (70.7 and 43.2 for ArCH_2_O and ArCH_2_N, respectively) according to a previous 2D NMR spectra analysis reported by Sainas et al., 2018,^18^ and ^13^C chemical shift analysis of other oxygen vs nitrogen alkylated compounds.^38−45^ Ester **19a** was then hydrolyzed under basic conditions to obtain the corresponding acid **20** (quantitative yield), which was then used for the preparation of the common intermediate **21**. Starting from acid **20**, the corresponding acyl chloride was obtained via treatment with oxalyl chloride and was used without any further purification in the reaction with the dimethylaluminum amide of 2,3,5,6-tetrafluoro-4-bromoaniline, giving the desired amide **21** in a 55% yield. Once again, the benzylic protecting group transferred from the exocyclic oxygen to the endocyclic N1 nitrogen in the pyrazolo[1,5-*a*]pyridine system. Compound **21** was used as a common building block for desired compounds **22**, **23**–**29**. First, Buchwald–Hartwig coupling conditions,^46^ with morpholine, were used to obtain **22** (59% yield), while a Suzuki reaction, involving the corresponding boronic acids, gave **23**–**29** (yield range: 70–94%). Compounds **22**–**29** were then converted to the desired targets **6**–**9**, **14**–**17** via treatment with trifluoroacetic acid (TFA) in the presence of thioanisole (Scheme 2), which was used as a scavenger of the benzylic cation.

**Scheme 3:**
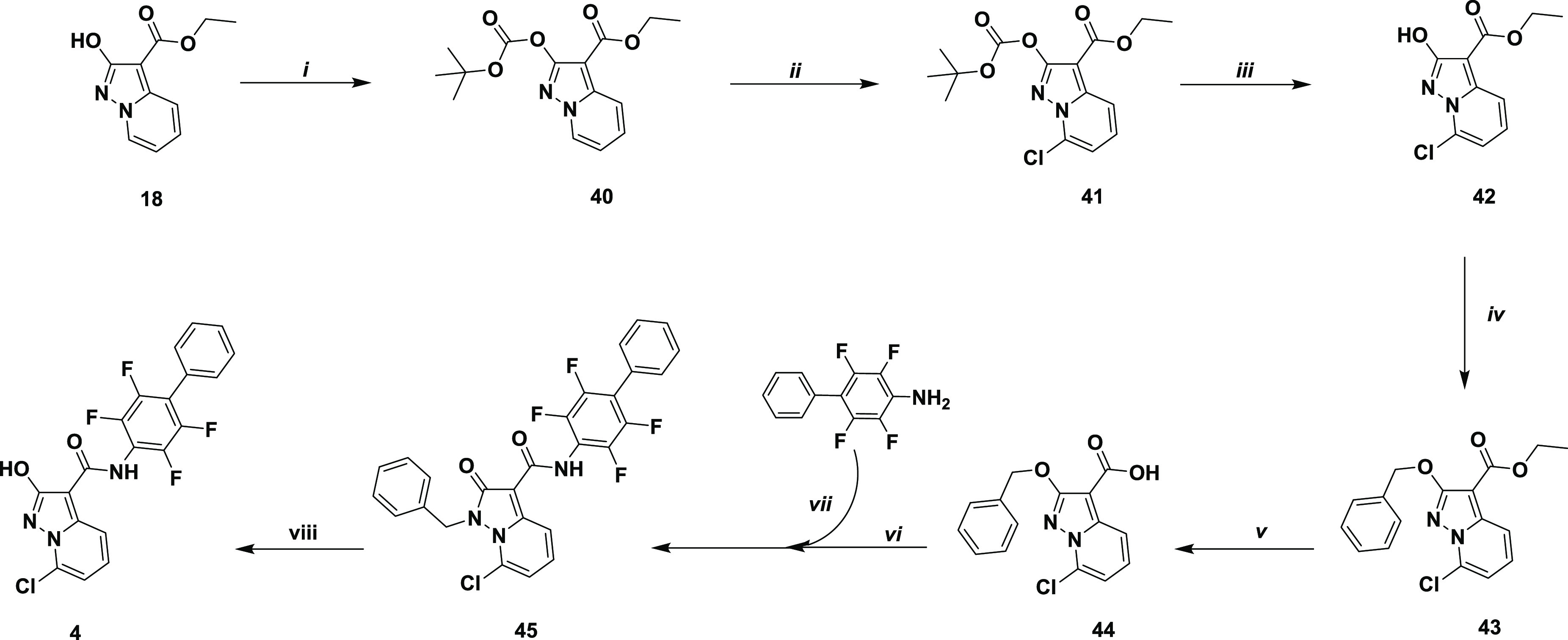
Synthetic Methodologies for the Synthesis of Compound **4** (i) Cs_2_CO_3_, *tert*-butoxycarbonyl anhydride, dry THF, reflux; (ii) (a) nitrogen atmosphere, lithium hexamethyldisilylazide (LiHMDS, 1.0 M, dry THF), −78 °C, 1 h; (b) nitrogen atmosphere, hexachloroethane rt; (iii) trifluoroacetic acid, dry dichloromethane, rt; (iv) benzyl bromide, Cs_2_CO_3_, dry DMF, rt; (v) (a) 6 M NaOH, ethanol, 75 °C; (b) 2M HCl, rt; (vi) nitrogen atmosphere, oxalyl chloride, dry DMF, dry THF; (vii) AlMe_3_, dry toluene, reflux; (viii) thioanisole, trifluoroacetic acid, 70 °C.

A dedicated synthetic scheme was applied for the synthesis of compound **4**; the hydroxyl group of **18** was O-protected with a Boc group to afford **40**.^39^ By use of lithium hexamethyldisilylazide on **40**, the pyrazolo[1,5-*a*]pyridine moiety was selectively deprotonated on the 7 position. Subsequently, quenching the lithium salt of **40** with hexachloroethane, which was used as an electrophile source of Cl^+^, afforded compound **41** in a good yield.^47^ In order to move the reaction scheme forward and prepare the subsequent coupling steps, the Boc group was ideally exchanged for a benzylic group that is more stable in acidic conditions. The Boc group was quantitatively removed under mild acidic conditions (TFA), giving hydroxyazole **42**, which was reacted with benzyl bromide affording compound **43** (90% over two steps). It is worth noting that, in this case, the endocyclic N1 benzylated isomer was obtained only in traces because of the presence of a chlorine in position 7. Ester **43** was then hydrolyzed under basic conditions to give the corresponding acid **44** (quantitative yield), which was then used for the preparation of amide **45**, under the conditions described above; 2,3,5,6-tetrafluoro-4-phenylaniline was activated with trimethylaluminum and reacted with the **44** acid chloride to give the desired amide **45** in a 38% yield. Compound **45** was then converted to the desired target **4** via treatment with TFA in the presence of thioanisole (Scheme 3), which was used as a scavenger of the benzylic cation.

## Conclusions

In this work, we have investigated the drug-like properties and SAR of compound **1**, the lead of a novel class of *h*DHODH inhibitors that are based on an unusual carboxylic group bioisostere, 2-hydroxypyrazolo[1,5-*a*]pyridine. Starting from **1** and investigating its SAR, we have identified the *meta* position of its C ring as the most favorable for substitution. Of the series produced, comparable enzymatic IC_50_ values resulted in dramatic differences in cellular activity. In particular, all modulations that were intended to improve solubility profiles and result in more polar compounds gave compounds with reduced cellular differentiation and lower apoptotic activity. On the other hand, all substituents with improved LogD^7.4^ resulted in compounds with improved cellular potency. Among the new derivatives, good results were obtained with **17**, which was able to induce myeloid differentiation U937 with an EC_50_ of 4.6 nM, 41 times more effective than phase I/II brequinar. Despite being a strong proapoptotic agent, EC_50_(U937) = 17 nM), **17** seems to be a safe compound as it is characterized by low cytotoxicity toward non-AML cells (EC_30_(Jurkat) > 100 μM), which indicates lower toxicity than **1** and brequinar itself. It is worth noting how the improvement of **17**, in terms of cell activity, was not associated with lower solubility, as the solubility of **17** and **1** is just comparable. It must be noted also that the same strategy allowed the design of a second candidate (**15**), superior to either the lead **1** and brequinar, although, in this case, less soluble than both leads. However, when we investigated the *in vitro* metabolism for compounds **1**, **15**, and **17**, while compounds **1** and **15** showed good metabolic stability in rat hepatic liver microsomes after incubation for 2 h, compound **17** showed a weaker *in vitro* stability, being converted in its hydroxylated metabolite, because this deserves to be subject to further modification.

Being that compound **1** is the candidate with the best balance between cell activity and drug-like properties, we choose it to start *in vivo* studies. Compound **1** showed a nontoxic *in vivo* profile when administered at doses of 10 and 25 mg/kg every 3 days for 5 weeks in Balb/c mice. These data demonstrate that compound **1** presents a low *in vivo* toxicity profile and could be a good candidate for future *in vivo* efficacy experiment

We can conclude that this class of *h*DHODH inhibitors contains candidates, such as **1**, **15**, and **17**, that are characterized by strong antileukemic activity and an optimal toxicity profile and whose performance is at least comparable to that of other competitors that are already in clinical trials. Compound **1** for its good activity, stability, and toxicity profile will be considered for *in vivo* tests on animal model.

## Experimental Section

### Chemistry. General Methods

All chemical reagents were obtained from commercial sources (Sigma-Aldrich, Alfa Aesar, FluoroChem) and used without further purification. Thin-layer chromatography (TLC) was carried out to monitor reaction progress. Analytical-grade solvents (acetonitrile, diisopropyl ether, diethyl ether, dichloromethane [DCM], dimethylformamide [DMF], ethanol 99.8% v/v, ethyl acetate [EtOAc], hexane, methanol [MeOH], petroleum ether bp 40–60 °C [petroleum ether], toluene) were used without further purification. When needed, solvents were dried over 4 Å molecular sieves. Tetrahydrofuran (THF) was distilled from Na and benzophenone under N_2_ immediately prior to use. Thin layer chromatography (TLC) was carried out on silica gel on 5 cm × 20 cm plates at a 0.25 mm layer thickness. Anhydrous Na_2_SO_4_ was used as a drying agent for the organic phases. Compound purification was achieved either using flash column chromatography on silica gel (Merck Kieselgel 60, 230–400 mesh ASTM) and the eluents indicated in the procedures for each compound or using CombiFlash Rf 200 (Teledyne Isco) with 5–200 mL/min, 200 psi (with an automatic injection valve), and RediSep Rf Silica columns (Teledyne Isco), with the eluents indicated in the procedures for each compound. Compounds synthesized in our laboratory generally varied between 90% and 99% purity. Biological experiments were performed on compounds with a purity of at least 95%. Purity was checked using two UHPLC analytical methods. HPLC analyses were performed on an UHPLC chromatographic system (PerkinElmer, Flexar). The analytical columns were an UHPLC Acquity CSH fluoro-phenyl (2.1 mm × 100 mm, 1.7 μm particle size, Waters) and a reverse-phase (RP) C18 Phenomenex column (2.1 mm × 100 mm, 1.7 μm particle size). Compounds were dissolved in acetonitrile and injected through a 20 μL loop. The mobile phase consisted of acetonitrile/water with 0.1% trifluoroacetic acid (ratio between 60/40 and 40/60, depending on the compound’s retention factor). UHPLC retention times were obtained at flow rates of 0.5 mL/min, and the column effluent was monitored at 230, 254, and 262 nm, referenced against a 360 nm wavelength. Melting points (mp) were measured on capillary apparatus (Büchi 540). Final mp determination was achieved by placing the sample at a temperature that was 10 °C below the mp and applying a heating rate of 1 °C min^–1^. All compounds were routinely checked by ^1^H and ^13^C NMR and mass spectrometry. The IR spectra of solid compounds were recorded on an FT-IR (PerkinElmer SPECTRUM BXII, KBr dispersions), using the diffuse reflectance apparatus DRIFT ACCY. MS spectra were performed on a Waters Micromass ZQ equipped with an ESCi source for electrospray ionization mass spectra. ^1^H and ^13^C NMR spectra were performed on a JEOL ECZR600. The following abbreviations are used for coupling patterns: br = broad, s = singlet, d = doublet, dd = doublet of doublets, t = triplet, q = quartet, m = multiplet. Chemical shifts (δ) are given in parts per million (ppm). In this work, protons and carbons are labeled (*a*, *b*, *c*, *d*, *e*, *f*, *g*, *h*, *l*, *m*, *n*, *o*, *p*, *q*, *r*, and *s*) according to Scheme 1. Values marked with an asterisk (∗, ∗∗, and ∗∗∗) are interchangeable. Detailed ^13^C spectra of tetrafluorinated biphenyl compounds (final compounds **4**–**17** and protected final compounds) have not been entirely reported due to their especially complicated patterns (attributable to the multiple couplings between fluorine and carbon atoms). For these spectra, only the ^13^C signals, caused by the heterocyclic substructure and nonaromatic carbons, are assigned. For the final compounds **4**–**17**, HRMS spectra were recorded on an LTQ-Orbitrap XL Plus (Thermo Scientific, Bremen, Germany) mass spectrometer, equipped with an atmospheric pressure interface and an ESI ion source instrument. Compounds **18**, **30**, and **31** were prepared according to previously described procedures.^18^

### Ethyl 2-((4-Methoxybenzyl)oxy)pyrazolo[1,5-*a*]pyridine-3-carboxylate (**19a**) and Ethyl *N*-(4-Methoxybenzyl)-2-oxopyrazolo[1,5-*a*]pyridine-3-carboxylate (**19b**)

4-Methoxybenzyl bromide (645 mg, 3.20 mmol, 1.10 equiv) was added dropwise to a mixture of **18** (600 mg, 2.91 mmol) and Cs_2_CO_3_ (2.295 g, 7.04 mmol, 2.4 equiv) in dry DMF (15 mL). The reaction mixture was stirred overnight at room temperature, and water (100 mL) was then added. The mixture was extracted using EtOAc (4 × 70 mL), and the combined organic layer was dried under Na_2_SO_4_ and evaporated under reduced pressure to give a yellow oil. This oil showed two spots on the TLC (eluent: petroleum ether/EtOAc 60/40 v/v) that were ascribed to the two pyrazolo[1,5-*a*]-pyridine regioisomers. The mixture was separated using flash chromatography (eluent: petroleum ether/EtOAc 2/1 v/v, after elution of first isomer dichloromethane/MeOH 95/5 v/v).

### **19a**

First isomer eluted. White solid after a first trituration with hexane, followed by filtration and a second trituration with water (111.3–112.5 °C). Yield 61%. ^1^H NMR (600 MHz, chloroform-*d*) δ 1.40 (*t*, *J* = 7.1 Hz, 3H, -OCH_2_C*H*_3_); 3.81 (*s*, 3H, -OC*H*_3_), 4.36 (*q*, *J* = 7.1 Hz, 2H, -OC*H*_2_CH_3_), 5.43 (*s*, 2H, -OC*H*_2_Ar), 6.83 (*t*, 1H, *J* = 6.7 Hz, *H*-*b*), 6.91 (*d*, 2H, *J* = 8.6 Hz, *H*-*n*), 7.35 (*t*, 1H, *J* = 7.7 Hz, *H*-*c*), 7.48 (*d*, 2H, *J* = 8.5 Hz, *H-m*), 8.00 (*d*, 1H, *J* = 8.8 Hz, *H*-*d*), 8.29 (*d*, 1H, *J* = 6.8 Hz, *H*-*a*); ^13^C NMR (151 MHz, chloroform-*d*) δ 14.7 (-OCH_2_*C*H_3_), 55.4 (-O*C*H_3_), 59.7 (-O*C*H_2_CH_3_), 70.7 (-O*C*H_2_Ar), 88.5 (*C-f*), 112.6 (*C-b*), 113.9 (*C-n*), 118.3 (*C-d*), 127.8 (*C-c*)*, 128.9 (*C-a*)*, 129.0 (*C-l*)*, 129.3(*C-m*), 142.9 (*C-e*), 159.5 (*C-o*), 163.4 (*C-g*)*, 165.2 (*C-h*)*. MS (ES+): 327 (M + 1).

### **19b**

Second isomer eluted. White solid. (158.3–159.2 °C, from diisopropyl ether). Yield 27%. ^1^H NMR (600 MHz, DMSO-*d*_6_) δ 1.28 (*t*, 3H, *J* = 7.1 Hz, -OCH_2_C*H*_3_), 3.69 (*s*, 3H, -OC*H*_3_), 4.21 (*q*, 2H, *J* = 7.1 Hz, -OC*H*_2_CH_3_), 5.35 (*s*, 2H, -NC*H*_2_Ar), 6.88 (*d*, 2H, *J* = 8.5 Hz, *H*-*n*), 6.96 (*t*, 1H, *J* = 6.8 Hz, *H*-*b*), 7.19 (*d*, 2H, *J* = 8.4 Hz, *H*-*m*), 7.58 (*t*, 1H, *J* = 8.0 Hz, *H*-*c*), 7.91 (*d*, 1H, *J* = 8.8 Hz, *H*-*d*), 8.45 (*d*, 1H, *J* = 6.8 Hz, *H*-*a*); ^13^C NMR (151 MHz, DMSO-*d*_6_) δ 14.6 (-OCH_2_*C*H_3_), 43.2 (-N*C*H_2_Ar), 55.1 (-O*C*H_3_), 58.5 (-O*C*H_2_CH_3_), 83.5 (*C-f*), 112.4 (*C-b*), 114.3 (*C-n*), 116.3 (*C-d*), 125.3 (*C-a*), 125.7 (*C-l*), 128.8 (*C-m*), 132.4 (*C-c*), 142.8 (*C-e*), 159.0 (*C-o*), 160.0 (*C-h*)*, 163.2 (*C-g*)*. MS (ES+): 327 (M + 1).

### 2-((4-Methoxybenzyl)oxy)pyrazolo[1,5-*a*]pyridine-3-carboxylic Acid (**20**)

6 M NaOH (5.0 equiv) was added to a solution of compound **19a** (785 mg, 2.40 mmol) in EtOH (20 mL). The mixture was stirred for 4 h at 75 °C and then neutralized with 6 M HCl and was concentrated under reduced pressure. The mixture was cooled to 0 °C, then acidified with 2 M HCl until pH 2 was reached, granting a suspension. This suspension was filtered to give **20** as a white solid (162.8–163.9 °C dec, from water). Yield 90%. ^1^H NMR (600 MHz, DMSO-*d*_6_) δ 3.76 (*s*, 3H, -OC*H*_3_), 5.34 (*s*, 2H, -OC*H*_2_Ar), 6.96 (*d*, 2H, *J* = 8.4 Hz, *H*-*n*), 7.02 (*t*, 1H, *J* = 6.7 Hz, *H*-*b*), 7.45 (*d*, 2H, *J* = 8.3 Hz, *H*-*m*), 7.51 (*t*, 1H, *J* = 7.9 Hz, *H*-*c*), 7.92 (*d*, 1H, *J* = 8.8 Hz, *H*-*d*), 8.66 (*d*, 1H, *J* = 6.7 Hz, *H*-*a*), 12.07 (*s*, 1H, −COO*H*); ^13^C NMR (151 MHz, DMSO-*d*_6_) δ 55.1 (-O*C*H_3_), 70.1 (-O*C*H_2_Ar), 87.6 (*C-f*), 113.1 (*C-b*), 113.8 (*C-n*), 117.3 (*C-d*), 128.4 (*C-a*)*, 128.5 (*C-l*)*, 129.5 (*C-c*)*, 129.9 (*C-m*), 142.3 (*C-e*), 159.2 (*C-o*), 163.5 (*C-h*)*, 164.4 (*C-g*)*. MS (ES+): 299 (M + 1).

### *N*-(4-Bromo-2,3,5,6-tetrafluorophenyl)-2-((4-methoxybenzyl)oxy)pyrazolo[1,5-*a*]pyridine-3-carboxamide (**21**)

Oxalyl chloride (0.54 mL, 6.30 mmol, 3.0 equiv) and dry DMF (1 drop) were added to a cooled (0 °C) solution of **20** (630 mg, 2.10 mmol) in dry THF (15 mL) kept under a nitrogen atmosphere. The resulting mixture was stirred for 2 h at room temperature. In parallel, a 2 M solution of AlMe_3_ in toluene (1.8 mL, 3.57 mmol, 1.7 equiv) was added to a solution of 4-bromo-2,3,5,6-tetrafluoroaniline (769 mg, 3.15 mmol, 1.5 equiv) in dry toluene (10 mL) under a nitrogen atmosphere. The resulting suspension was stirred 3 h at room temperature. The solution of acyl chloride was then concentrated under reduced pressure, and the residue was dissolved in dry THF (10 mL; this step was repeated three times to eliminate all gaseous residues). The acyl chloride was dissolved in dry toluene (15 mL), and the solution was added to the suspension described above. The reaction mixture was stirred at 85 °C overnight, then cooled to room temperature, quenched with methanol, and evaporated. The residue was dissolved in EtOAc (80 mL), 0.5 M HCl (50 mL) was then added, and the layers were separated. The aqueous phase was extracted twice with EtOAc, and the combined organic layers were washed with brine, dried, and evaporated under reduced pressure. The crude material was purified using flash chromatography (eluent: petroleum ether/EtOAc/DCM 2/1/1 v/v/v) to afford the title compound as a white solid (177.4–178.0 °C, triturated with diisopropyl ether). Yield 55%. ^1^H NMR (600 MHz, chloroform-*d*) δ 3.79 (*s*, 3H, -OC*H*_3_), 5.41 (*s*, 2H, -NC*H*_2_Ar), 6.77 (*t*, 1H, *J* = 6.9 Hz, *H*-*b*), 6.90 (*d*, 2H, *J* = 8.5 Hz, *H*-*n*), 7.21 (*d*, 2H, *J* = 8.5 Hz, H-*m*), 7.46 (*t*, 1H, *J* = 7.9, Hz, *H*-*c*), 7.75 (*d*, 1H, *J* = 6.9 Hz, *H*-*a*), 8.27 (*t*, 1H, *J* = 8.8 Hz, *H*-*d*), 9.98 (*s*, 1H, -N*H*); ^13^C NMR (151 MHz, chloroform-*d*) δ 45.2 (-N*C*H_2_Ar), 55.5 (-O*C*H_3_), 87.1 (*C-f*), 96.4 (*t*, *J* = 22.6 Hz, *C-s*)*, 112.9 (*C-b*), 115.0 (*C-n*), 117.0 (*t*, *J* = 14.8 Hz, *C-p*)*, 118.3 (*C-d)*, 123.1 (*C-a*), 124.2 (*C-l*), 128.6 (*C-m*), 131.8 (*C-c*), 142.5 (*C-e*), 142.8 (*dd*, *J* = 251.6, 14.9 Hz, (*C-r*)**, 145.2 (*dd*, *J* = 246.4, 14.2 Hz, (*C-q*)**, 160.0 (*C-o*)***, 161.4 (*C-h*)***, 162.2 (*C-g*)***. MS (ES+): 524/526 (M + 1).

### 1-(4-Methoxybenzyl)-2-oxo-*N*-(2,3,5,6-tetrafluoro-4-morpholinophenyl)-1,2-dihydropyrazolo[1,5-*a*]pyridine-3-carboxamide (**22**)

Cs_2_CO_3_ (782 mg, 2.4 mmol, 3.00 equiv) was added to a solution of **21** (420 mg, 0.80 mmol, 1.00 equiv) and morpholine (209 mg, 2.40 mmol, 3.00 equiv) in toluene (30 mL). After degasification with nitrogen for 10 min, Pd(OAc)_2_ (18 mg, 0.08 mmol, 0.10 equiv) and BINAP (100 mg, 0.16 mmol, 0.20 equiv) were added, and the mixture was degassed again for 5 min. The resulting suspension was heated at 110 °C in a sealed flask under a nitrogen atmosphere. After 3.5 h, the heating was stopped, the mixture concentrated to reduced pressure, and water was added. The resulting suspension was extracted with EtOAc (3 × 50 mL). The combined organic fractions were collected, dried, and concentrated under reduced pressure. The crude product was purified by flash chromatography (eluent: petroleum ether/EtOAc/DCM 1/1/1 v/v/v) giving a solid that was triturated with diisopropyl ether to give the title compound as a white solid (237.2–237.5 °C dec). Yield: 59%. ^1^H NMR (600 MHz, chloroform-*d*) δ 3.24–3.28 (*m*, 4H, -NC*H*_2_CH_2_O-), 3.79 (*s*, 3H, -OC*H*_3_), 3.81–3.85 (*m*, 4H, -NCH_2_C*H*_2_O-), 5.40 (*s*, 2H, -NC*H*_2_Ar), 6.74 (*t*, 1H, *J* = 6.9 Hz, *H-b*), 6.90 (*d*, 2H, *J* = 8.5 Hz, *H-n*), 7.21 (*d*, 2H, *J* = 8.5 Hz, *H-m*), 7.44 (*t*, 1H, *J* = 7.9 Hz, *H-c*), 7.73 (*d*, 1H, *J* = 6.9, Hz, *H-a*), 8.28 (*d*, 1H, *J* = 8.9 Hz, *H-d*), 9.75 (*s*, 1H, -N*H*); ^13^C NMR (151 MHz, chloroform-*d*) δ 45.1 (-N*C*H_2_Ar), 51.5 (-N*C*H_2_CH_2_O-), 55.5 (-O*C*H_3_), 67.5 (-NCH_2_*C*H_2_O-), 87.2 (*C-f*), 111.3 (*t*, *J* = 15.5 Hz, *C-p*)*, 112.6 (*C-b*), 114.9 (*C-n*), 118.3 (*C-d*), 123.1 (*C-a*), 124.3 (*C-l*), 127.8 (*t*, *J* = 11.0 Hz, *C-s*)*, 128.6 (*C-m*), 131.6 (*C-c*), 142.5 (*C-e*), 143.2 (*d*, *J* = 248.2 Hz, *C-q*)**, 143.5 (*d*, *J* = 247.9, Hz, *C-r*)**, 160.0 (*C-o*)***, 162.0 (*C-h*)***, 162.1 (*C-g*)***. MS (ES+): 553 (M + Na).

### General Procedure: Suzuki Reaction Used for the Production of Compounds **23**–**29**

Pd(PPh_3_)_4_ (90 mg, 0.08 mmol, 0.20 equiv) was added to a solution of **21** (200 mg, 0.38 mmol, 1.00 equiv) and K_2_CO_3_ (158 mg, 1.14 mmol, 3.00 equiv) in dioxane/water mixture (9:1 v/v). After stirring the resulting mixture under a nitrogen atmosphere for 1 h at rt, the corresponding boronic acid (0.760 mmol, 2.0 equiv) was added. The reaction mixture was then heated at reflux under a nitrogen atmosphere. After 2 h, an additional amount of boronic acid (0.38 mmol, 1.0 equiv) was added, and the reaction mixture was heated at reflux for another 2 h before it was cooled to room temperature and concentrated under reduced pressure. The crude material was taken-up with water (100 mL) and the mixture was extracted with EtOAc (3 × 60 mL). The combined organic layers were dried over Na_2_SO_4_ and concentrated under reduced pressure. The crude product was purified by flash chromatography (see the conditions below).

### 1-(4-Methoxybenzyl)-2-oxo-*N*-(2,3,5,6-tetrafluoro-4-(thiophen-2-yl)phenyl)-1,2-dihydropyrazolo[1,5-*a*]pyridine-3-carboxamide (**23**)

The crude product was purified by flash chromatography (eluent: petroleum ether/ EtOAc 1/1 v/v) giving a solid that was recrystallized from acetonitrile (8 mL) to give the title compound as a white solid (197.4–198.1 °C from acetonitrile). Yield: 72%. ^1^H NMR (600 MHz, chloroform-*d*) δ 3.79 (*s*, 3H, -OC*H*_3_), 5.41 (*s*, 2H, -NC*H*_2_Ar), 6.76 (*t*, 1H, *J* = 6.6 Hz, *H-b*), 6.90 (*d*, 2H, *J* = 8.5 Hz, *H-n*), 7.16–7.20 (*m*, 1H, *aromatic proton*), 7.21 (*d*, 2H, *J* = 8.5 Hz, *H-m*), 7.45 (*t*, 1H, *J* = 7.7 Hz, *H-c*), 7.54 (*d*, 1H, *J* = 5.0 Hz, *aromatic proton*), 7.59 (*d*, 1H, *J* = 3.2 Hz, *aromatic proton*), 7.75 (*d*, 1H, *J* = 6.9 Hz, *H-a*), 8.28 (*d*, 1H, *J* = 8.8 Hz, *H-d*), 10.00 (*s*, 1H, -N*H*); ^13^C NMR (151 MHz, chloroform-*d*) δ 45.2 (-N*C*H_2_Ar), 55.5 (-O*C*H_3_), 87.2 (*C-f*), 112.3 (*C-b*), 115.0 (*C-n*), 115.7 (*t*, *J* = 16.2 Hz, *C-p*)*, 118.4 (*C-d*), 123.1 (*C-a*), 124.3 (*C-l*), 127.3 (*thiophene carbon*), 127.9 (*thiophene carbon*), 128.1 (*t*, *J* = 3.3 Hz, *C-s*)*, 128.6 (*C-m*), 128.5 (*thiophene carbon*), 130.0 (*t*, *J* = 5.3 Hz, *thiophene carbon*), 131.7 (*C-c*), 142.6 (*C-e*), 142.9 (*dd*, *J* = 248.0, 15.8 Hz, *C-q*)**, 144.0 (*d*, *J* = 247.0, Hz, *C-r*)**, 160.0 (*C-o*)***, 161.6 (*C-h*)***, 162.2 (*C-g*)***. MS (ES+): 528.2 (M + 1).

### 1-(4-Methoxybenzyl)-2-oxo-*N*-(2,3,5,6-tetrafluoro-4-(pyridin-3-yl)phenyl)-1,2-dihydropyrazolo[1,5-*a*]pyridine-3-carboxamide (**24**)

The crude product was purified by flash chromatography (eluent: from petroleum ether/EtOAc 6/4 v/v to 3/7 v/v) giving a solid. This solid was then triturated with diisopropyl ether to give the title compound as a white solid (mp 202.6–203.8 °C from trituration with diisopropyl ether). Yield: 90%. ^1^H NMR (600 MHz, chloroform-*d*) δ 3.78 (*s*, 3H, -OC*H*_3_), 5.41 (*s*, 2H, -NC*H*_2_Ar), 6.77 (*t*, 1H, *J* = 6.7 Hz, *H-b*), 6.89 (*d*, 2H, *J* = 8.5 Hz, *H-n*), 7.21 (*d*, 2H, *J* = 8.5 Hz, *H-m*), 7.43–7.49 (*m*, 2H, *aromatic protons and H-c*), 7.77 (*d*, 1H, *J* = 6.9 Hz, H-*a*), 7.84 (*d*, 1H, *J* = 7.8 Hz, *aromatic proton*), 8.27 (*d*, 1H, *J* = 8.8 Hz, *H-d*), 8.69 (*d*, 1H, *J* = 2.9 Hz, *aromatic proton*), 8.76 (s, 1H, *aromatic proton*), 10.06 (*s*, 1H, -N*H*); ^13^C NMR (151 MHz, chloroform-*d*) δ 45.2 (-N*C*H_2_Ar), 55.5 (-O*C*H_3_), 87.1 (*C-f*), 112.9 (*C-b*), 114.0 (*t*, *J* = 16.9 Hz, *C-p*)*, 115.0 (*C-n*), 117.4 (*t*, *J* = 15.8 Hz, *C-s*)*, 118.3 (*C-d*), 123.1 (*C-a*), 123.7 (*pyridine carbon*), 124.2 (*C-l*), 124.3 (*pyridine carbon*), 128.6 (*C-m*), 131.8 (*C-c*), 137.9 (*pyridine carbon*), 142.6 (*C-e*), 142.9 (*d*, *J* = 252.7 Hz, *C-q*)**, 144.2 (*d*, *J* = 249.7 Hz, *C-r*)**, 149.9 (*pyridine carbon*), 150.5 (*pyridine carbon*), 160.0 (*C-o*)***, 161.6 (*C-h*)***, 162.2 (*C-g*)***. MS (ES+): 523 (M + 1).

### 1-(4-Methoxybenzyl)-2-oxo-*N*-(2,3,5,6-tetrafluoro-4-(2-(trifluoromethyl)pyridin-4-yl)phenyl)-1,2-dihydropyrazolo[1,5-*a*]pyridine-3-carboxamide (**25**)

The crude product was purified by flash chromatography (eluent: from petroleum ether/EtOAc 6/4 v/v to 3/7 v/v) giving a solid. This solid was then triturated with diisopropyl ether, and the title compound was obtained as a pale-yellow solid (mp 192.7–193.9 °C from trituration with diisopropyl ether). Yield: 70%. ^1^H NMR (600 MHz, chloroform-*d*) δ 3.79 (*s*, 3H, -OC*H*_3_), 5.42 (*s*, 2H, -NC*H*_2_Ar), 6.79 (*t*, 1H, *J* = 6.7 Hz, *H-b*), 6.90 (*d*, 2H, *J* = 8.5 Hz, *H-n*), 7.22 (*d*, 2H, *J* = 8.5 Hz, *H-m*), 7.48 (*t*, 1H, *J* = 7.9 Hz, *H-c*), 7.64 (*d*, 1H, *J* = 4.9 Hz, *pyridine proton*), 7.78 (*d*, 1H, *J* = 6.9 Hz, *H-a*), 7.84 (*s*, 1H, *pyridine proton*), 8.29 (*d*, 1H, *J* = 8.8 Hz, *H-d*), 8.88 (d, 1H, *J* = 4.9 Hz, *pyridine proton*), 10.15 (*s*, 1H, -N*H*); ^13^C NMR (151 MHz, chloroform-*d*) δ 45.3 (-N*C*H_2_Ar), 55.5 (-O*C*H_3_), 87.1 (*C-f*), 113.0 (*C-b*), 113.2 (*t*, *J* = 15.6 Hz, *C-p*)*, 115.0 (*C-n*), 118.4 (*C-d*), 118.9 (*t*, *J* = 14.7 Hz, *C-s*)*, 121.5 (*q*, *J* = 274.5 Hz, -CF_3_), 121.7 (*q*, *J* = 2.1 Hz, *pyridine carbon*), 123.1 (*C-a*), 124.2 (*C-l*), 127.6 (*pyridine carbon*), 128.6 (*C-m*), 131.9 (*C-c*), 137.9 (*pyridine carbon*), 142.6 (*C-e*), 142.8 (*d*, *J* = 247.0 Hz, *C-q*)**, 144.2 (*d*, *J* = 245.4 Hz, *C-r*)**, 149.0 (*q*, *J* = 34.9 Hz, *pyridine carbon*), 150.6 (*pyridine carbon*), 160.1 (*C-o*)***, 161.4 (*C-h*)***, 162.3 (*C-g*)***. MS (ES+): 591.

### 1-(4-Methoxybenzyl)-2-oxo-*N*-(2,3,5,6-tetrafluoro-3′-hydroxy-[1,1′-biphenyl]-4-yl)-1,2-dihydropyrazolo[1,5-*a*]pyridine-3-carboxamide (**26**)

The crude product was purified by flash chromatography (eluent: petroleum ether/EtOAc 1/2 v/v) giving a solid. This solid was then triturated with diisopropyl ether in order to remove traces of O=PPh_3_, giving the title compound as a white solid (236.9–237.4 °C from diisopropyl ether). Yield: 79%. ^1^H NMR (600 MHz, DMSO-*d*_6_) δ 3.71 (*s*, 3H, -OC*H*_3_), 5.49 (*s*, 2H, -NC*H*_2_Ar), 6.88–6.97 (*m*, 5H, *aromatic protons* and *H-n*), 7.07 (*t*, 1H, *J* = 7.1 Hz, *H-b*), 7.28 (*d*, 2H, *J* = 8.3 Hz, *H-m*), 7.35 (*t*, 1H, *J* = 8.1 Hz, *aromatic proton*), 7.67 (*t*, 1H, *J* = 7.9 Hz, *H-c*), 8.03 (*d*, 1H, *J* = 8.8 Hz, *H-d*), 8.57 (*d*, 1H, *J* = 6.9 Hz, *H-a*), 9.78 (*br s*, 1H, -O*H*), 10.07 (*s*, 1H, -N*H*); ^13^C NMR (151 MHz, DMSO-*d*_6_) δ 43.8 (-N*C*H_2_Ar), 55.1 (-O*C*H_3_), 85.5 (*C-f*), 113.2 (*C-b*), 114.4 (*C-n*), 116.2 (*C-d*), 116.3 (*aromatic carbon*), 116.6 (*t*, *J* = 14.6 Hz, *C-p*)*, 116.9 (*2 carbon, C-s partially overlapped with aromatic carbon*)*, 120.7 (*aromatic carbon*), 125.3 (*C-a*), 125.4 (*C-l*), 127.7 (*aromatic carbon*), 128.8 (*C-m*), 129.9 (*aromatic carbon*), 132.9 (*C-c*), 141.4 (*C-e*), 142.3 (*d*, *J* = 248.9 Hz, *C-q*)**, 143.3 (*d*, *J* = 251.3 Hz, *C-r*)**, 157.5 (*aromatic carbon*), 159.1 (*C-o*)***, 160.7 (*C-h*)***, 161.7 (*C-g*)***. MS (ES+): 538 (M + 1).

### 1-(4-Methoxybenzyl)-2-oxo-*N*-(2,3,5,6-tetrafluoro-3′-(trifluoromethoxy)-[1,1′-biphenyl]-4-yl)-1,2-dihydropyrazolo[1,5-*a*]pyridine-3-carboxamide (**27**)

The crude product was purified by flash chromatography (eluent: petroleum ether/DCM 2/1 v/v) giving a solid. This solid was then triturated with diisopropyl ether, and the title compound was obtained as a white solid (162.7–163.0 °C from diisopropyl ether). Yield: 82%. ^1^H NMR (600 MHz, chloroform-*d*) δ 3.80 (*s*, 3H, -OC*H*_3_), 5.42 (*s*, 2H, -NC*H*_2_Ar), 6.77 (*t*, 1H, *J* = 6.9 Hz, *H-b*), 6.91 (*d*, 2H, *J* = 8.5 Hz, *H-n*), 7.22 (*d*, 2H, *J* = 8.5 Hz, *H-m*), 7.32 (*d*, 1H, *J* = 8.1 Hz, *aromatic proton*), 7.37 (*s*, 1H, *aromatic proton*), 7.43 (*d*, 1H, *J* = 7.7 Hz, *aromatic proton*), 7.47 (*t*, 1H, *J* = 7.9 Hz, *H-c*), 7.53 (*t*, 1H, *J* = 8.0 Hz, *aromatic proton*), 7.76 (*d*, 1H, *J* = 6.9 Hz, *H-a*), 8.31 (*d*, 1H, *J* = 8.8 Hz, *H-d*), 10.03 (*s*, 1H, -N*H*); ^13^C NMR (151 MHz, chloroform-*d*) δ 45.2 (-N*C*H_2_Ar), 55.4 (-O*C*H_3_), 87.1 (*C-f*), 112.8 (*C-b*), 115.0 (*C-n*), 115.9 (*t*, *J* = 16.1 Hz, *C-p*)*, 117.0 (*t*, *J* = 15.6 Hz, *C-s*)*, 118.3 (*C-d*), 120.6 (*q*, *J* = 257.6 Hz, -O*C*F_3_), 121.5 (*aromatic carbon*), 123.0 (*aromatic carbon*), 123.1 (*C-a*), 124.2 (*C-l*), 128.5 (*C-m*), 128.8 (*aromatic carbon*), 129.4 (*aromatic carbon*), 130.1 (*aromatic carbon*), 131.7 (*C-c*),, 142.5 (*C-e*), 142.8 (*d*, *J* = 247.5 Hz, *C-q*)**, 144.0 (*d*, *J* = 249.5 Hz, *C-r*)**, 149.3 (*aromatic carbon*), 160.0 (*C-o*)***, 161.6 (*C-h*)***, 162.2 (*C-g*)***. MS (ES+): 606.6, 628.6.

### 1-(4-Methoxybenzyl)-2-oxo-*N*-(2,3,5,6-tetrafluoro-3′-methoxy-[1,1′-biphenyl]-4-yl)-1,2-dihydropyrazolo[1,5-*a*]pyridine-3-carboxamide (**28**)

The crude product was purified by flash chromatography (eluent: petroleum ether/EtOAc/dichloromethane 1.5/1/2 v/v/v) giving the title compound as a beige solid (172.9–173.8 °C from diisopropyl ether). Yield: 94%. ^1^H NMR (600 MHz, chloroform-*d*) δ 3.79 (*s*, 3H, -OC*H*_3_), 3.85 (*s*, 3H, -OC*H*_3_), 5.42 (*s*, 2H, -NC*H*_2_Ar), 6.76 (*t*, 1H, *J* = 6.9 Hz, *H-b*), 6.90 (*d*, 2H, *J* = 8.5 Hz, H-*n*), 6.97–7.03 (*m*, 2H, *aromatic protons*), 7.06 (*d*, 1H, *J* = 7.5 Hz, *aromatic proton*), 7.22 (*d*, 2H, *J* = 8.5 Hz, *H-m*), 7.41 (*t*, 1H, *J* = 7.9 Hz, *aromatic proton*), 7.45 (*t*, 1H, *J* = 7.9 Hz, *H-c*), 7.75 (*d*, 1H, *J* = 6.9 Hz, *H-a*), 8.29 (*d*, 1H, *J* = 8.8 Hz, *H-d*), 9.99 (*s*, 1H, -N*H*); ^13^C NMR (150 MHz, chloroform-*d*) δ 45.2 (-N*C*H_2_Ar), 55.5 (2 x -O*C*H_3_), 87.2 (*C-f*), 112.3 (*C-b*), 114.9 (*aromatic carbon*), 115.0 (*C-n*), 115.8 (*aromatic carbon*), 116.3 (*t*, *J* = 15.0 Hz, *C-p*)*, 117.6 (*t*, *J* = 19.6 Hz, *C-s*)*, 118.4 (*C-d*), 122.7 (*aromatic carbon*), 123.1 (*C-a*), 124.3 (*C-l*), 128.6 (*C-m*), 128.8 (*aromatic carbon*), 129.7 (*aromatic carbon*), 131.7 (*C-c*), 142.6 (*C-e*), 142.9 (*d*, *J* = 248.6 Hz, *C-q*)**, 144.2 (*d*, *J* = 244.2, Hz, *C-r*)**, 159.7 (*aromatic carbon*)***, 160.0 (*C-o*)***161.7 (*C-h*)****, 162.2 (*C-g*)****. MS (ES+): 552.5 (M + 1).

### 1-(4-Methoxybenzyl)-2-oxo-*N*-(2,3,5,6-tetrafluoro-3′-propoxy-[1,1′-biphenyl]-4-yl)-1,2-dihydropyrazolo[1,5-*a*]pyridine-3-carboxamide (**29**)

The crude product was purified by flash chromatography (eluent: petroleum ether/EtOAc 2/3 v/v) to give the title compound as a beige solid (168.8–169.9 °C from diisopropyl ether). Yield: 86%. ^1^H NMR (600 MHz, chloroform-*d*) δ 1.06 (*t*, 3H, *J* = 7.4 Hz, -OCH_2_CH_2_C*H*_3_), 1.84 (*h*, 2H, *J* = 7.3 Hz, -OCH_2_C*H*_2_CH_3_), 3.80 (*s*, 3H, -OC*H*_3_), 3.96 (*t*, 2H, *J* = 6.6 Hz, -OC*H*_2_CH_2_CH_3_), 5.42 (*s*, 2H, -NC*H*_2_Ar), 6.76 (*t*, 1H, *J* = 7.0 Hz, *H-b*), 6.91 (*d*, 2H, *J* = 8.6 Hz, *H-n*), 6.99 (*dd*, 1H, *J* = 8.4, 2.2 Hz, *aromatic proton*), 7.00 (*s*, 1H, *aromatic proton*), 7.04 (*d*, 1H, *J* = 7.6 Hz, *aromatic proton*), 7.22 (*d*, 2H, *J* = 8.6 Hz, *H-m*), 7.39 (*t*, 1H, *J* = 7.9 Hz, *aromatic proton*), 7.46 (*t*, 1H, *J* = 7.9 Hz, *H-c*), 7.75 (*d*, 1H, *J* = 7.0 Hz, *H-a*), 8.31 (*d*, 1H, *J* = 8.8 Hz, *H-d*), 9.98(*s*, 1H, -N*H*); ^13^C NMR (151 MHz, chloroform-*d*) δ 10.6 (-OCH_2_CH_2_*C*H_3_), 22.7 (-OCH_2_*C*H_2_CH_3_), 45.2 (-N*C*H_2_Ar), 55.5 (-O*C*H_3_), 69.8 (-O*C*H_2_CH_2_CH_3_), 87.2 (*C-f*), 112.8 (*C-b*), 115.0 (*C-n*), 115.5 (*aromatic carbon*), 116.2 (*t*, *J* = 15.6 Hz, *C-p*)*, 116.4 (*aromatic carbon*), 117.7 (*t*, *J* = 16.8 Hz, *C-s*)*, 118.3 (*C-d*), 122.5 (*aromatic carbon*), 123.1 (*C-a*), 124.3 (*C-l*), 128.6 (*C-m*), 128.7 (*aromatic carbon*), 129.7 (*aromatic carbon*), 131.7 (*C-c*), 142.5 (*C-e*), 142.8 (*dd*, *J* = 248.5, 15.3 Hz, *C-q*)**, 144.2 (*d*, *J* = 248.5 Hz, *C-r*)**, 159.2 (*aromatic carbon*)***, 160.0 (*C-o*)***161.7 (*C-h*)****, 162.2 (*C-g*)****. MS (ES+): 580 (M + 1).

### General Procedure for the Synthesis of Pyrazolo[1,5-*a*]pyridine Related Amides **32**–**35**

Oxalyl chloride (3.0 mmol) and dry DMF (1 drop) were added to a cooled (0 °C) solution of O-protected pyrazolo[1,5-*a*]pyridine acid (1.0 mmol) **30** in dry THF (20 mL), under a nitrogen atmosphere. The obtained solution was stirred at room temperature for 2 h. The solution was then concentrated under reduced pressure and the residue dissolved in dry THF (10 mL; this step was repeated three times to eliminate all gaseous residues). The resulting acyl chloride was immediately used without any further purification and was dissolved in 10 mL of dry toluene and transferred to the solution described below. Trimethylaluminum (2.0 M in hexane, 1.5 mmol) was added to a solution of the appropriate aniline (see Supporting Information for the synthesis, 1.5 mmol), in dry toluene (15 mL), under a nitrogen atmosphere. The resulting mixture was stirred for 2 h at room temperature producing a brown suspension, and then the solution of the previously described acyl chloride in dry toluene (30 mL) was quantitatively added. The mixture was heated overnight at 90 °C and then cooled to rt. The reaction was quenched with 1 M HCl. The layers were resolved, and the aqueous phase was exhaustively extracted using EtOAc. The combined organic layer was washed with 1 M NaOH and brine, dried, and the solvent was evaporated under reduced pressure. The crude product was purified by column chromatography.

### 1-Benzyl-2-oxo-*N*-(2,3,4′,5,6-pentafluoro-[1,1′-biphenyl]-4-yl)-1,2-dihydropyrazolo[1,5-*a*]pyridine-3-carboxamide (**32**)

2,3,4′,5,6-Pentafluoro-[1,1′-biphenyl]-4-aniline was used. Flash chromatography (eluent: petroleum ether/EtOAc from 80:20 v/v to 50:50 v/v) was performed. Gray solid (mp 234.2–235.4 °C from trituration with diisopropyl ether). Yield 31%. ^1^H NMR (600 MHz, chloroform-*d*) δ 5.48 (*s*, 2H, -NC*H*_2_Ph), 6.77 (*t*, 1H, *J* = 6.9 Hz, *H-b*), 7.19 (*t*, 2H, *J* = 8.6 Hz, *aromatic protons*), 7.28 (*d*, 2H, *J* = 7.4 Hz, *aromatic protons*), 7.32–750 (*m*, 6H, *aromatic protons*), 7.73 (*d*, 1H, *J* = 6.9, *H-a*), 8.30 (*d*, 1H, *J* = 8.8, *H-d*), 9.98 (*s*, 1H, -N*H*); ^13^C NMR (151 MHz, chloroform-*d*) δ 45.7 (-N*C*H_2_Ph), 87.2 (*C-f*), 112.9 (*C-b*), 115.9 (*d*, *J* = 21.9 Hz, *aromatic carbon*), 116.4 (*t*, *J* = 17.1 Hz, *C-p*)*, 116.7 (*d*, *J* = 16.7 Hz, *C-s*)*, 118.4 (*C-d*), 123.0 (*C-a*), 123.5 (*aromatic carbon*), 127.1 (*aromatic carbon*), 129.0 (*aromatic carbon*), 129.7 (*aromatic carbon*), 131.8 (*C-c*), 132.8 (*d*, *J* = 8.4 Hz, *aromatic carbon*), 132.5 (*aromatic carbon*), 142.6 (*C-e*), 142.8 (*d*, *J* = 244.1 Hz, *C-q*)**, 144.2 (*d*, *J* = 250.7 Hz, *C-r*)**, 161.7 (*C-g*)***, 162.2 (*C-h*)***, 163.1 (*d*, *J* = 249.5 Hz, -*C*F). MS (ESI) 510 (M + 1).

### 1-Benzyl-2-oxo-*N*-(2,3,3′,5,6-pentafluoro-[1,1′-biphenyl]-4-yl)-1,2-dihydropyrazolo[1,5-*a*]pyridine-3-carboxamide (**33**)

2,3,3′,5,6-Pentafluoro-[1,1′-biphenyl]-4-aniline was used. Flash chromatography (eluent: petroleum ether/EtOAc from 80:20 v/v to 50:50 v/v) was performed. Pale yellow solid (mp 195.4–196.3 °C from trituration with diisopropyl ether). Yield 40 *%*. ^1^H NMR (600 MHz, DMSO-*d*_6_) δ 5.57 (*s*, 2H, -NC*H*_2_Ph), 7.07 (*t*, 1H, *J* = 6.9 Hz, *H-b*), 7.29–7.45 (*m*, 7H, *aromatic protons*), 7.50 (*d*, 1H, *J* = 9.5 Hz, *aromatic proton*), 7.62 (*dd*, 1H, *J* = 14.3, 7.7 Hz, *aromatic proton*), 7.68 (*t*, 1H, *J* = 7.9 Hz, *H-c*), 8.05 (*d*, 1H, *J* = 8.7 Hz, *H-d*), 8.53 (*d*, 1H, *J* = 7.0 Hz, *H-a*), 10.12 (*s*, 1H, -N*H*); ^13^C NMR (151 MHz, DMSO-*d*_6_) δ 44.2 (-N*C*H_2_Ph), 85.4 (*C-f*), 113.3 (*C-b*), 115.4 (*t*, *J* = 17.3 Hz, *C-p*)*, 116.2 (*C-d*), 116.4 (*d*, *J* = 20.8 Hz, *aromatic carbon*), 117.0 (*C-s*)*, 117.2 (*d*, *J* = 23.1 Hz, *aromatic carbon*), 125.2 (*C-a*), 126.5 (*C-l*), 127.2 (*aromatic carbon*), 128.2 (*C-c*), 128.8 (*d*, *J* = 9.8 Hz, *aromatic carbon*), 129.1 (*aromatic carbon*), 130.9 (*d*, *J* = 8.4 Hz, *aromatic carbon*), 132.9 *(aromatic carbon)*, 133.7 *(aromatic carbon)*, 141.4 (*C-e*), 142.2 (*d*, *J* = 242.5 Hz, *C-q*)**, 143.3 (*d*, *J* = 245.8 Hz, *C-r*)**, 144.1, 160.6 (*C-g*)***, 161.2 (*C-h*)***, 162.0 (*d*, *J* = 244.3 Hz, *aromatic carbon*). MS (ESI) 508 (M – 1).

### 1-Benzyl-2-oxo-*N*-(2,3,5,6-tetrafluoro-4′-(trifluoromethyl)-[1,1′-biphenyl]-4-yl)-1,2-dihydropyrazolo[1,5-*a*]pyridine-3-carboxamide (**34**)

2,3,5,6-Tetrafluoro-4′-(trifluoromethyl)-[1,1′-biphenyl]-4-aniline was used. Flash chromatography eluent: from petroleum ether/EtOAc 80:20 v/v to 50:50 v/v. Pale yellow solid (mp 262.2–263.5 °C from trituration with diisopropyl ether). Yield 41 *%*. ^1^H NMR (600 MHz, chloroform-*d*) δ 5.49 (*s*, 2H, -NC*H*_2_Ph), 6.78 (*t*, 1H, *J* = 6.8 Hz, *H-b*), 7.29 (*d*, 2H, *J* = 7.4 Hz, *aromatic protons*), 7.33–7.43 (*m*, 3H, *aromatic protons*), 7.48 (*t*, 1H, *J* = 7.9 Hz, *H-c*), 7.62 (*d*, 2H, *J* = 7.9 Hz, *aromatic protons*), 7.73 (*d*, 1H, *J* = 6.9 Hz, *H-a*), 7.76 (*d*, 2H, *J* = 8.1 Hz, *aromatic protons*), 8.31 (*d*, 1H, *J* = 8.8 Hz, *H-d*), 10.04 (*s*, 1H, -N*H*); ^13^C NMR (151 MHz, chloroform-*d*) δ 45.7 (-N*C*H_2_Ph), 87.2 (*C-f*), 112.9 (*C-b*), 116.1 (*t*, *J* = 16.5 Hz, *C-p*)*, 117.2 (*t*, *J* = 15.6 Hz, *C-s*)*, 118.4 (*C-d*), 123.0 (*C-a*), 124.0 (*q*, *J* = 272.5 Hz, -*C*F_3_), 125.7 (*q*, *J* = 3.8 Hz, *aromatic carbon*), 127.1 (*aromatic carbon*), 129.0 (*C-c*), 129.7 (*aromatic carbon*), 130.8 (*aromatic carbon*), 131.1 (*q*, *J* = 32.8 Hz, *aromatic carbon*), 131.4 (*aromatic carbon*), 131.9 (*aromatic carbon*), 132.5 (*aromatic carbon*), 142.6 (*C-e*), 142.8 (*dd*, *J* = 251.8, 17.4 Hz, *C-q*)**, 144.1 (*dd*, *J* = 244.7, 15.4 Hz, *C-r*)**, 161.6 (*C-g*)***, 162.2 (*C-h*)***. MS (ESI) 560 (M + 1).

### 1-Benzyl-2-oxo-*N*-(2,3,5,6-tetrafluoro-3′-(trifluoromethyl)-[1,1′-biphenyl]-4-yl)-1,2-dihydropyrazolo[1,5-*a*]pyridine-3-carboxamide (**35**)

2,3,5,6-Tetrafluoro-3′-(trifluoromethyl)-[1,1′-biphenyl]-4-aniline was used. Flash chromatography eluent: from petroleum ether/EtOAc 80:20 v/v to 50:50 v/v. White solid (mp 190.9–191.8 °C from trituration with diisopropyl ether). Yield 40 *%*. ^1^H NMR (600 MHz, chloroform-*d*) δ 5.49 (*s*, 2H, -NC*H*_2_Ph), 6.78 (*t*, 1H, *J* = 6.8 Hz, *H-b*), 7.29 (*d*, 2H, *J* = 7.3 Hz, *aromatic protons*), 7.33–7.42 (*m*, 3H, *aromatic protons*), 7.48 (*t*, 1H, *J* = 7.9 Hz, *H-c*), 7.64 (*t*, 1H, *J* = 7.7 Hz, *aromatic proton*), 7.68 (*d*, 1H, *J* = 7.5 Hz, *aromatic proton*), 7.72 (*t*, 2H, *J* = 7.6 Hz, *aromatic protons*), 7.76 (*s*, 1H, *aromatic proton*), 8.31 (*d*, 1H, *J* = 8.8, *H-d*), 10.04 (*s*, 1H, -N*H*); ^13^C NMR (151 MHz, chloroform-*d*) δ 45.7 (-N*C*H_2_Ph), 87.2 (*C-f*), 112.9 (*C-b*), 116.4 (*t*, *J* = 16.2 Hz, *C-p*)*, 117.2 (*t*, *J* = 15.2 Hz, *C-s*)*, 118.5 (*C-d*), 123.0 (*C-a*), 124.0 (*q*, *J* = 272.1 Hz, -*C*F_3_), 125.9 (*q*, *J* = 3.9 Hz, *aromatic carbon*), 127.1 (*aromatic carbon*), 127.2 (*aromatic carbon*), 128.5 (*aromatic carbon*), 129.0 (*aromatic carbon*), 129.3 (*aromatic carbon*), 129.7 (*aromatic carbon*), 131.3 (*q*, *J* = 32.4 Hz), 131.8 (*C-c*), 132.5 (*aromatic carbon*), 133.7 (*aromatic carbon*), 142.7 (*C-e*), 142.8 (*d*, *J* = 252.0 Hz, *C-q*)**, 144.2 (*d*, *J* = 248.2 Hz, *C-r*)**, 161.6 (*C-g*)***, 162.2 (*C-h*)***. MS (ESI) 560 (M + 1).

### 2-Hydroxy-*N*-(2,3,5,6-tetrafluoro-[1,1′-biphenyl]-4-yl)-4,5,6,7-tetrahydropyrazolo[1,5-*a*]pyridine-3-carboxamide (**5**)

Palladium on carbon (Pd/C, 20% w/w) was added to a solution of compound **31** (1.0 mmol) in dry THF (10 mL). The resulting mixture was stirred under a hydrogen atmosphere of 40 bar, at a temperature of 65 °C for 3 h using a microwave SynthWAVE. The suspension was filtered through Celite, and the cake was washed with methanol. The filtrate was concentrated under reduced pressure. The obtained solid was further purified by flash chromatography (eluent: dichloromethane/EtOAc/HCOOH 80:20:1 v/v/v). White solid (mp 270.9–272.9 °C dec, from diisopropyl ether). Yield 40% ^1^H NMR (600 MHz, DMSO-*d*_6_) δ 1.70–1.80 (*m*, 2H, *H-b*), 1.89–1.98 (*m*, 2H, *H-c*), 2.92 (*t*, 2H, *J* = 6.1 Hz, *H-d*), 3.86 (*t*, 2H, *J* = 5.8 Hz, *H-a*), 7.49–7.55 (*m*, 5H, *aromatic protons*), 9.11 (*s*, 1H, -N*H*), 11.94 (v *br s*,1H, -O*H*). Exchangeable proton signals overlapped with the water signal; ^13^C NMR (151 MHz, DMSO-*d*_6_) δ 18.5 (*C-b*), 22.1 (*C-d*), 22.9 (*C-c*), 46.5 (*C-a*), 95.5 (*C-f*), 116.8 (*t*, *J* = 14.3 Hz, *C-s*)*, 117.0 (*t*, *J* = 17.4 Hz, *C-p*)*, 126.7 (*aromatic carbon*), 128.9 (*aromatic carbon*), 129.4 (*aromatic carbon*), 130.1 (*aromatic carbon*), 141.8 (*C-e*) 143.2 (*d*, *J* = 248.4, 21.8 Hz, *C-q*)**, 144.3 (*d*, *J* = 244.0 Hz, *C-r*)**, 159.7 (*C-g*)***, 160.7 (*C-h*)***. MS (ES−) 404 (M – 1). IR (KBr) v (cm^–1^): 3338, 2924, 2519, 1685, 1577, 1522, 1437, 1374, 1316, 1283, 1241, 1144, 992. ESI-HRMS (*m*/*z*): [M + H]^+^ calcd for C_20_H_16_F_4_N_3_O_2_, 406.1173; obsd, 406,1170.

### General Procedure: Removal of the 4-Methoxybenzyloxy Moiety To Give Final Compounds **6**–**9**, **15**–**17**

Thioanisole (220 μL, 1.87 mmol, from 5.0 equiv to 10 equiv) was added to a solution of the corresponding starting material (200 mg, 0.37 mmol, 1.0 equiv) in TFA (3 mL). The mixture was heated at 70 °C for 2 h and then cooled to rt. The mixture was partially concentrated, and the crude product was taken up with water to give a suspension that was filtered, and the solid was washed with an additional amount of cold water. The resulting solid was triturated with diisopropyl ether to produce the title compounds, often directly in pure form (see details above).

### 2-Hydroxy-*N*-(2,3,5,6-tetrafluoro-4-morpholinophenyl)pyrazolo[1,5-*a*]pyridine-3-carboxamide (**6**)

Compound **22** (200 mg, 0.38 mmol, 1.0 equiv) was dissolved in a solution of thioanisole (250 μL, 2.26 mmol, 6.0 equiv) in TFA (2 mL). The residue was triturated with hexane and diisopropyl ether and then purified by flash chromatography (eluent: petroleum ether/DCM/MeOH 5/4/0.4 v/v/v). The resulting solid was triturated with diisopropyl ether to give the title compound as a white solid (276.5–277.2 °C dec from diisopropyl ether). Yield: 42%. UHPLC retention time: 3.952 min. UHPLC purity: 96.49%. ^1^H NMR (600 MHz, DMSO-*d*_6_) δ 3.18–3.25 (*m*, 4H, -NC*H*_2_CH_2_O-), 3.68–3.75 (*m*, 4H, -NCH_2_C*H*_2_O-), 7.0 (*t*, 1H, *J* = 6.8 Hz, *H-b*), 7.48 (*t*, 1H, *J* = 7.8 Hz, *H-c*), 7.94 (*d*, 1H, *J* = 8.7 Hz, *H-d*), 8.58 (*d*, 1H, *J* = 6.7 Hz, *H-a*), 8.76 (*s*, 1H, -N*H*), 12.83 (v *br s*, 1H, -O*H*); ^13^C NMR (151 MHz, DMSO-*d*_6_) δ 51.0 (-N*C*H_2_CH_2_O-), 66.7 (-NCH_2_*C*H_2_O-), 88.2 (*C-f*), 111.5 (*t*, *J* = 15.1 Hz, *C-p*)*, 113.1 (*C-b*), 116.8 (*C-d*), 127.5 (*t*, *J* = 11.2 Hz, *C-s*)*, 128.2 (*C-a*), 129.1 (*C-c*), 141.7 (*C-e*), 142.2 (*dd*, *J* = 243.5, 6.2 Hz, *C-q*)**, 143.4 (*dd*, *J* = 244.6, 14.4 Hz, *C-r*)**, 160.8 (*C-h*)***, 162.7 (*C-g*)***. MS (ES−): 409 (M – 1). ESI-HRMS (*m*/*z*): [M – H]^−^ calcd for C_18_H_13_F_4_N_4_O_3_, 409.0929; obsd, 409.0925.

### 2-Hydroxy-*N*-(2,3,5,6-tetrafluoro-4-(thiophen-2-yl)phenyl)pyrazolo[1,5-*a*]pyridine-3-carboxamide (**7**)

The solid was crystallized three times from acetonitrile (20 mL) to give the title compound as a gray solid (278.4–279.9 °C dec from acetonitrile). Yield: 23%. UHPLC retention time: 5.863 min. UHPLC purity: 97.00%. ^1^H NMR (600 MHz, DMSO-*d*_6_) δ 7.03 (*t*, 1H, *J* = 6.6 Hz, *H-b*), 7.31 (*t*, 1H, *J* = 4.3 Hz, *aromatic proton*), 7.51 (*t*, 1H, *J* = 7.8 Hz, *H-c*), 7.64 (*d*, 1H, *J* = 2.8 Hz, *aromatic proton*), 7.92 (*d*, 1H, *J* = 5.0 Hz, *aromatic proton*), 7.97 (*d*, 1H, *J* = 8.8 Hz, *H-d*), 8.61 (*d*, 1H, *J* = 6.7 Hz, *H-a*), 8.95 (*s*, 1H, -N*H*), 12.90 (*br s*, 1H, -O*H*); ^13^C NMR (151 MHz, DMSO-*d*_6_) δ 88.2 (*C-f*), 110.9 (*t*, *J* = 15.6 Hz, *C-p*)*, 113.8 (*C-b*), 17.03 (*t*, *J* = 16.5 Hz, *C-s*)*, 116.8 (*C-d*), 126.2 (*thiophene carbon*), 127.8 (*thiophene carbon*), 128.4 (*C-a*), 129.2 (*thiophene carbon*), 129.5 (*C-c*), 130.7 (*t*, *J* = 4.0 Hz, *thiophene carbon*), 141.8 (*C-e*), 143.0 (*dd*, *J* = 246.5, 14.3 Hz, *C-q* and *C-r*), 160.4 (*C-h*)**, 162.7(*C-g*)**. MS (ES−): 406 (M – 1). ESI-HRMS (*m*/*z*): [M – H]^−^ calcd for C_18_H_8_F_4_N_3_O_2_S, 406.0279; obsd, 406.0275.

### 2-Hydroxy-*N*-(2,3,5,6-tetrafluoro-4-(pyridin-3-yl)phenyl)pyrazolo[1,5-*a*]pyridine-3-carboxamide (**8**)

Pale yellow solid (mp 283.4–286.7 °C dec from trituration with diisopropyl ether). Yield: 76%. UHPLC retention time: 3.377 min. UHPLC purity: 95.10%. ^1^H NMR (600 MHz, DMSO-*d*_6_) δ 6.98 (*t*, 1H, *J* = 6.7 Hz, *H-b*), 7.47 (*t*, 1H, *J* = 7.9 Hz, *H-c*), 7.61 (*dd*, 1H, *J* = 7.7, 5.0 Hz, *aromatic proton*), 7.95 (*d*, 1H, *J* = 8.6 Hz, *H-d*), 8.05 (*d*, 1H, *J* = 7.4 Hz, *aromatic proton*), 8.57 (*d*, 1H, *J* = 6.5 Hz, *H-a*), 8.71 (*d*, 1H, *J* = 4.1 Hz, *aromatic proton*), 8.78 (*s*, 1H, *aromatic proton*), 9.30 (*br s*, 1H, -N*H*); ^13^C NMR (151 MHz, DMSO-*d*_6_) δ 88.3 (*C-f*), 112.9 (*C-b*), 113.8 (*t*, *J* = 17.4 Hz, *C-p*)*, 116.6 (*C-d*), 117.9 (*t*, *J* = 14.7 Hz, *C-s*)*, 123.3 (*pyridine carbon*), 124.0 (*pyridine carbon*), 128.0 (*C-a*), 128.9 (*C-c*), 137.9 (*pyridine carbon*), 141.7 (*C-e*), 142.6 (*dd*, *J* = 245.2, 15.1 Hz, *C-q*)**, 143.4 (*dd*, *J* = 245.7, 17.2 Hz, *C-q*)**, 150.2 (*pyridine carbon*), 150.3 (*pyridine carbon*), 160.6 (*C-h*)***, 163.9 (*C-g*)***. MS (ES+): 403 (M + H). ESI-HRMS (*m*/*z*): [M + H]^+^ calcd for C_19_H_11_F_4_N_4_O_2_, 403.0813; obsd, 403.0810.

### 2-Hydroxy-*N*-(2,3,5,6-tetrafluoro-4-(2-(trifluoromethyl)pyridin-4-yl)phenyl)pyrazolo[1,5-*a*]pyridine-3-carboxamide (**9**)

Pale yellow solid (mp 258.4–259.2 °C dec from trituration with diisopropyl ether). Yield: 55%. UHPLC retention time: 8.010 min. UHPLC purity: 98.60%. ^1^H NMR (600 MHz, DMSO-*d*_6_) δ 7.01 (*t*, 1H, *J* = 6.6 Hz, *H-b*), 7.49 (*t*, 1H, *J* = 7.7 Hz, *H-c*), 7.96 (*d*, 1H, *J* = 8.6 Hz, *H-d*), 8.00 (*d*, 1H, *J* = 3.5 Hz, *aromatic proton*), 8.22 (*s*, 1H, *aromatic proton*), 8.59 (*d*, 1H, *J* = 6.6 Hz, *H-a*), 8.99 (*d*, *J* = 4.6 Hz, 1H, *aromatic proton*), 9.22 (*br s*, 1H, -N*H*); ^13^C NMR (151 MHz, DMSO-*d*_6_) δ 88.2 (*C-f*), 113.1 (C-p* *overlapped with C-b*), 116.7 (*C-d*), 118.9 (*t*, *J* = 13.5 Hz, *C-s*)*, 121.5 (*q*, *J* = 274,6 Hz, -*CF*_3_), 121.8 (*pyridine carbon*), 128.2 (*C-a*), 128.5 (*C-c*), 129.1 (*q*, *J* = 12.5 Hz, *pyridine carbon*), 137.3 (*pyridine carbon*), 141.7 (*C-e*), 142.6 (*d*, *J* = 246.8 Hz, *C-q*)**, 143.3 (*d*, *J* = 247.0 Hz, *C-r*)**, 147.1 (*q*, *J* = 34.1 Hz, *pyridine carbon*), 151.0 (*pyridine carbon*), 160.3 (*C-h*)***, 163.4 (*C-g*)***. MS (ES+): 470 (M + H). ESI-HRMS (*m*/*z*): [M + H]^+^ calcd for C_20_H_10_F_7_N_4_O_2_, 471.0686; obsd, 471.0684.

### 2-Hydroxy-*N*-(2,3,5,6-tetrafluoro-3′-hydroxy-[1,1′-biphenyl]-4-yl)pyrazolo[1,5-*a*]pyridine-3-carboxamide (**14**)

White solid (275.9–276.4 °C dec from diisopropyl ether). Yield: 58%. UHPLC retention time: 5.749 min. UHPLC purity: 95.3%. ^1^H NMR (600 MHz, DMSO-*d*_6_) δ 6.84–6.99 (*m*, 3H, *aromatic protons*), 7.03 (*t*, 1H, *J* = 6.8 Hz, *H-b*), 7.35 (*t*, 1H, *J* = 8.2 Hz, *aromatic proton*), 7.51 (*t*, 1H, *J* = 7.8 Hz, *H-c*), 7.98 (*d*, 1H, *J* = 7.4 Hz, *H-d*), 8.61 (*d*, 1H, *J* = 4.5 Hz, *H-a*), 8.90 (*s*, 1H, -Ar-O*H*), 9.78 (*s*, 1H, -N*H*), 12.86 (*s*, 1H, -O*H*); ^13^C NMR (151 MHz, DMSO-*d*_6_) δ 88.2 (*C-f*), 113.3 (*C-b*), 114.7 (*t*, *J* = 13.5 Hz, *C-p*)*, 116.3 (*C-d*), 116.8 (*aromatic carbon*), 117.4 (*t*, *J* = 16.3 Hz, *C-s*)*, 120.7 (*aromatic carbon*), 127.6 (*aromatic carbon*), 128.4 (*C-a*), 128.3 (*aromatic carbon*), 129.1 (*C-c*), 129.9 (*aromatic carbon*), 141.7 (*C-e*), 142.7 (*d*, *J* = 248.4 Hz, *C-q*)**, 143.2 (*d*, *J* = 244.8 Hz, *C-r*)**, 157.5 (*aromatic carbon*), 160.4 (*C-h*)***, 162.6 (*C-g*)***. MS (ES−): 416 (M – 1). ESI-HRMS (*m*/*z*): [M + H]^+^ calcd for C_20_H_12_F_4_N_3_O_3_, 418.0809; obsd, 418.0807.

### 2-Hydroxy-*N*-(2,3,5,6-tetrafluoro-3′-(trifluoromethoxy)-[1,1′-biphenyl]-4-yl)pyrazolo[1,5-*a*]pyridine-3-carboxamide (**15**)

The crude product was partially dissolved in methanol, filtered from the insoluble solid, concentrated, and precipitated via the addition of diisopropyl ether. Beige solid (228.1–229.5 °C dec from diisopropyl ether). Yield: 26%. UHPLC retention time: 6.892 min. UHPLC purity: 99.00%. ^1^H NMR (600 MHz, DMSO-*d*_6_) δ 7.03 (*t*, 1H, *J* = 6.7 Hz, *H-b*), 7.51 (*t*, 1H, *J* = 7.8 Hz, *H-c*), 7.55 (*d*, 1H, *J* = 8.0 Hz, *aromatic proton*), 7.60–7.67 (*m*, 2H, *aromatic protons*), 7.71 (*t*, 1H, *J* = 7.9 Hz, *aromatic proton*), 7.98 (*d*, 1H, *J* = 8.7 Hz, *H-d*), 8.61 (*d*, 1H, *J* = 6.7 Hz, *H-a*), 8.97 (*s*, 1H, -N*H*), 12.94 (*br s*, 1H, -O*H*); ^13^C NMR (151 MHz, DMSO-*d*_6_) δ 88.2 (*C-f*), 113.2 (*C-b*), 115.5 (*t*, *J* = 17.2 Hz, *C-p*)*, 116.8 (*C-d*), 117.6 (*t*, *J* = 15.1 Hz, *C-s*)*, 120.1 (*q*, *J* = 257.0 Hz, -O*C*F_3_), 122.1 (*aromatic carbon*), 123.0 (*aromatic carbon*), 128.4 (*C-a*), 128.8 (*aromatic carbon*), 129.2 (*C-c*), 129.5 (*aromatic carbon*), 130.9 (*aromatic carbon*), 141.7 (*C-e*), 142.7 (*dd*, *J* = 246.2, 13.6 Hz, *C-q*)**, 143.3 (*d*, *J* = 245.6 Hz, *C-r*)**, 148.4 (*aromatic carbon*), 160.3 (*C-h*)***, 162.7 (*C-g*)***. MS (ES−): 484 (M – 1). ESI-HRMS (*m*/*z*): [M + H]^+^ calcd for C_21_H_11_F_7_N_3_O_3_, 486.0683; obsd, 486.0681.

### 2-Hydroxy-*N*-(2,3,5,6-tetrafluoro-3′-methoxy-[1,1′-biphenyl]-4-yl)pyrazolo[1,5-*a*]pyridine-3-carboxamide (**16**)

The residue was treated with diethyl ether, the mixture was filtered, and the insoluble residue was purified by flash chromatography (eluent: DCM/MeOH 97/3 v/v then petroleum ether/dichloromethane/methanol 5/4/0.6 v/v/v) to obtain the title compound as a white solid (223.6–224.1 °C dec from diisopropyl ether). Yield: 47%. UHPLC retention time: 5.788 min. UHPLC purity: 95.41%. ^1^H NMR (600 MHz, DMSO-*d*_6_) δ 3.81 (*s*, 3H, -OC*H*_3_), 7.02 (*t*, 1H, *J* = 6.6 Hz, *H-b*), 7.08–7.16 (*m*, 3H, *aromatic protons*), 7.45–7.54 (*m*, 2H, *H-c* and *aromatic proton*), 7.98 (*d*, 1H, *J* = 8.6 Hz, *H-d*), 8.61 (*d*, 1H, *J* = 6.6 Hz, *H-a*), 8.99 (*br s*, 1H, -N*H*); ^13^C NMR (150 MHz, DMSO-*d*_6_) δ 55.4 (-O*C*H_3_), 88.2 (*C-f*), 113.2 (*C-b*), 115.0 (*C-d*), 115.8 (*aromatic carbon*), 116.8 (*aromatic carbon*), 117.0 (*t*, *J* = 16.1 Hz, *C-s and C-p*)*, 122.3 (*aromatic carbon*), 127.8 (*C-a*), 128.3 (*aromatic carbon*), 129.1 (*C-c*), 130.0 (*aromatic carbon*), 141.8 (*C-e*), 142.9 (*d*, *J* = 246.3 Hz, *C-q*)**, 143.4 (*d*, *J* = 252.5 Hz, *C-r*)**, 159.4 (*aromatic carbon*), 160.5 (*C-h*)****, 163.0 162.2 (*C-g*)****. MS (ES−): 430 (M – 1), MS (ES+) 432 (M + 1). ESI-HRMS (*m*/*z*): [M + H]^+^ calcd for C_21_H_14_F_4_N_3_O_3_, 432.0966; obsd, 432.0969.

### 2-Hydroxy-*N*-(2,3,5,6-tetrafluoro-3′-propoxy-[1,1′-biphenyl]-4-yl)pyrazolo[1,5-*a*]pyridine-3-carboxamide (**17**)

Beige solid (209.4–210.0 °C from diisopropyl ether). Yield: 69%. UHPLC retention time: 6.449 min. UHPLC purity: 98.03%. ^1^H NMR (600 MHz, DMSO-*d*_6_) δ 0.98 (*t*, 3H, *J* = 7.2 Hz, -OCH_2_CH_2_C*H*_3_), 1.68–1.81 (*m*, 2H, -OCH_2_C*H*_2_CH_3_), 3.98 (*t*, 2H, *J* = 6.1 Hz, -OC*H*_2_CH_2_CH_3_), 7.02 (*t*, 1H, *J* = 6.5 Hz, *H-b*), 7.05–7.14 (*m*, 3H, *aromatic protons*), 7.46 (*t*, 1H, *J* = 7.8 Hz, *aromatic proton* or *H-c*), 7.51 (*t*, 1H, *J* = 7.6 Hz, *aromatic proton* or *H-c*), 7.99 (*d*, 1H, *J* = 8.7 Hz, *H-d*), 8.61 (*d*, 1H, *J* = 6.5 Hz, *H-a*), 8.91 (*s*, 1H, -N*H*), 12.86 (*s*, 1H, -O*H*); ^13^C NMR (151 MHz, DMSO-*d*_6_) δ 10.4 (-OCH_2_CH_2_*C*H_3_), 22.0 (-OCH_2_*C*H_2_CH_3_), 69.2 (-O*C*H_2_CH_2_CH_3_), 88.2 (*C-f*), 113.3 (*C-b*), 115.5 (*C-d*), 116.2 (*aromatic carbon*), 116.8 (*aromatic carbon*), 116.9–117.7 (*m*, *C-s and C-p*), 122.2 (*aromatic carbon*), 127.8 (*aromatic carbon*), 128.4 (*C-a*), 129.2 (*C-c*), 129.9 (*aromatic carbon*), 141.7 (*C-e*), 142.8 (*dd*, *J* = 245.5, 21.8 Hz, *C-q*)**, 143.3 (*d*, *J* = 245.6 Hz, *C-r*)**, 158.8 (*aromatic carbon*)***, 160.4 (*C-h*)****, 162.6 (*C-g*)****. MS (ES−): 458 (M – 1), MS (ES+): 460 (M + 1). ESI-HRMS (*m*/*z*): [M + H]^+^ calcd for C_23_H_18_F_4_N_3_O_3_, 460.1279; obsd, 460.1280.

### General Hydrogenation Procedure for the Production of Target Compounds **10**–**13**

Palladium on carbon (Pd/C, 6% w/w) was added to a solution of the appropriate amide (compounds **32**–**35**, 1.0 mmol), in dry THF (15 mL), and 37% HCl (1.0 mmol). The resulting mixture was vigorously stirred under a hydrogen atmosphere for 6 h. The suspension was filtered through Celite, and the cake was then washed with methanol. The filtrate was concentrated under reduced pressure. When necessary, the obtained solid was further purified by flash chromatography (see details below).

### 2-Hydroxy-*N*-(2,3,4′,5,6-pentafluoro-[1,1′-biphenyl]-4-yl)pyrazolo[1,5-*a*]pyridine-3-carboxamide (**10**)

Obtained from **32**, flash chromatography (eluent: dichloromethane/EtOAc/HCOOH 80:20:1 v/v/v). White solid (mp 293.4–294.5 °C dec from trituration with diisopropyl ether). Yield 75 *%*. UHPLC retention time: 4.323 min. UHPLC purity: 97.40%. ^1^H NMR (600 MHz, DMSO-*d*_6_) δ 7.03 (*t*, 1H, *J* = 6.7 Hz, *H-b*), 7.42 (*t*, 2H, *J* = 8.7 Hz, *aromatic protons*), 7.51 (*t*, 1H, *J* = 7.8 Hz, *H-c*), 7.57–7.71 (*m*, 2H, *aromatic protons*), 7.98 (*d*, 1H, *J* = 8.7, *H-d*), 8.62 (*d*, 1H, *J* = 6.7, *H-a*), 8.93 (*s*, 1H, -N*H*), 12.88 (*br s*, 1H, -O*H*); ^13^C NMR (151 MHz, DMSO-*d*_6_) δ 88.2 (*C-f*), 113.3 (*C-b*), 116.0 (*d*, *J* = 22.0 Hz, *aromatic carbon*), 116.3 (*t*, *J* = 17.4 Hz, *C-p*)*, 116.8 (*C-d*), 117.1 (*t*, *J* = 14.1 Hz, *C-s*)*, 123.0 (*aromatic carbon*), 128.4 (*C-a*), 129.2 (*C-c*), 132.5 (*d*, *J* = 8.4 Hz, *aromatic carbon*), 141.7 (*C-e*), 142.8 (*d*, *J* = 246.4 Hz, *C-q*)**, 143.3 (*d*, *J* = 248.6 Hz, *C-r*)**, 160.4 (*C-g*)***, 162.6 (*d*, *J* = 247.1 Hz, -*C*F), 162.7 (*C-h*)***. MS (ESI) 420 (M + 1). ESI-HRMS (*m*/*z*): [M + H]^+^ calcd for C_20_H_11_F_5_N_3_O_2_, 420.0766; obsd, 420.0766.

### 2-Hydroxy-*N*-(2,3,3′,5,6-pentafluoro-[1,1′-biphenyl]-4-yl)pyrazolo[1,5-*a*]pyridine-3-carboxamide (**11**)

Obtained from **33**, flash chromatography eluent: dichloromethane/EtOAc/HCOOH 80:20:1 v/v/v. White solid (mp 255.4–256.2 °C dec from trituration with diisopropyl ether). Yield 75 *%*. UHPLC retention time: 5.033 min. UHPLC purity: 96.50%. ^1^H NMR (600 MHz, DMSO-*d*_6_) δ 7.03 (*t*, 1H, *J* = 6.8 Hz, *H-b*), 7.33–7.56 (*m*, 4H, *aromatic protons*), 7.62 (*dd*, 1H, *J* = 14.3, 7.7 Hz, *aromatic proton*), 7.99 (*d*, 1H, *J* = 8.7, *H-d*), 8.62 (*d*, 1H, *J* = 6.8, *H-a*), 8.94 (*s*, 1H, -N*H*), 12.83 (*br s*, 1H, -O*H*); ^13^C NMR (151 MHz, CDCl_3_) δ 88.2 (*C-f*), 113.3 (*C-b*), 115.9 (*t*, *J* = 17.4 Hz, *C-p*)*, 116.4 (*d*, *J* = 20.9 Hz, *aromatic carbon*), 116.9 (*C-d*), 117.2 (*d*, *J* = 23.0 Hz, *aromatic carbon*), 117.4 (*t*, *J* = 14.8 Hz, *C-s*)*, 126.5 (*aromatic carbon*), 128.4 (*C-a*), 128.7 (*d*, *J* = 9.6 Hz, *aromatic carbon*), 129.2 (*C-c*), 130.9 (*d*, *J* = 8.3 Hz, *aromatic carbon*), 141.7 (*C-e*), 142.8 (*d*, *J* = 243.6 Hz, *C-q*)**, 143.2 (*d*, *J* = 244.6 Hz, *C-r*)**, 160.4 (*C-g*)***, 162.0 (*d*, *J* = 244.4 Hz, -*C*F), 162.7 (*C-h*)***. MS (ESI) 418 (M – 1). ESI-HRMS (*m*/*z*): [M + H]^+^ calcd for C_20_H_11_F_5_N_3_O_2_, 420.0766; obsd, 420.0763.

### 2-Hydroxy-*N*-(2,3,5,6-tetrafluoro-4′-(trifluoromethyl)-[1,1′-biphenyl]-4-yl)pyrazolo[1,5-*a*]pyridine-3-carboxamide (**12**)

Obtained from **34**, flash chromatography eluent: dichloromethane/EtOAc/HCOOH 80:20:1 v/v/v. White solid (mp 286.1–286.8 °C dec from trituration with diisopropyl ether). Yield 95%. UHPLC retention time: 4.880 min. UHPLC purity: 96.51%. ^1^H NMR (600 MHz, DMSO-*d*_6_) δ 7.03 (*t*, 1H, *J* = 6.7 Hz, *H-b*), 7.51 (*t*, 1H, *J* = 7.8 Hz, *H-c*), 7.83 (*d*, 2H, *J* = 7.8 Hz, *aromatic protons*), 7.94 (*d*, 2H, *J* = 8.0 Hz, *aromatic protons*), 7.98 (*d*, 1H, *J* = 8.7 Hz, *H-d*), 8.61 (*d*, 1H, *J* = 6.7 Hz, *H-a*), 9.00 (*s*, 1H, -N*H*), 12.92 (br *s*, 1H, -O*H*); ^13^C NMR (151 MHz, DMSO-*d*_6_) δ 88.2 (*C-f*), 113.3 (*C-b*), 115.7 (*t*, *J* = 16.9 Hz, *C-p*)*, 116.8 (*C-d*), 117.8 (*t*, *J* = 13.1 Hz, *C-s*)*, 124.0 (*q*, *J* = 272.4 Hz, -*C*F_3_), 125.8 (*q*, *J* = 3.3 Hz, *aromatic carbon*), 128.4 (*C-a*), 129.2 (*C-c*), 129.7 (*q*, *J* = 32.3 Hz, *aromatic carbon*), 131.0 (*aromatic carbon*), 131.2 (*aromatic carbon*), 141.7 (*C-e*), 142.7 (*dd*, *J* = 246.0, 14.9 Hz, *C-q*)**, 143.2 (*dd*, *J* = 244.9, 19.5 Hz, *C-r*)**, 160.3 (*C-g*)***, 162.7 (*C-h*)***. MS (ESI) 468 (M – 1). ESI-HRMS (*m*/*z*): [M + H]^+^ calcd for C_21_H_11_F_7_N_3_O_2_, 470.0734; obsd, 470.0731.

### 2-Hydroxy-*N*-(2,3,5,6-tetrafluoro-3′-(trifluoromethyl)-[1,1′-biphenyl]-4-yl)pyrazolo[1,5-*a*]pyridine-3-carboxamide (**13**)

Obtained from **35**, flash chromatography eluent: dichloromethane/EtOAc/HCOOH 80:20:1 v/v/v. Pale pink solid (mp 220.3–220.7 °C dec from trituration with diisopropyl ether). Yield 98%. UHPLC retention time: 4.767 min. UHPLC purity: 97.01%. ^1^H NMR (600 MHz, DMSO-*d*_6_) δ 7.03 (*t*, 1H, *J* = 6.7 Hz, *H-b*), 7.51 (*t*, 1H, *J* = 7.8 Hz, *H-c*), 7.82 (*t*, 1H, *J* = 7.7 Hz, *aromatic proton*), 7.91 (*d*, 2H, *J* = 7.6 Hz, *aromatic protons*), 7.95–8.02 (m, 2H, *aromatic proton* and *H-d*), 8.62 (*d*, 1H, *J* = 6.7, *H-a*), 8.98 (*s*, 1H, -N*H*), 12.87 (*br s*, 1H, -O*H*); ^13^C NMR (151 MHz, DMSO-*d*_6_) δ 88.2 (*C-f*), 113.3 (*C-b*), 115.6 (*t*, *J* = 17.0 Hz, *C-p*)*, 116.8 (*C-d*), 117.6 (*t*, *J* = 15.6 Hz, *C-s*)*, 123.9 (*q*, *J* = 271.9 Hz, -*C*F_3_), 126.2 (*aromatic carbon*), 126.9 (*aromatic carbon*), 127.9 (*aromatic carbon*), 128.4 (*C-a*), 129.2 (*C-c*), 129.7 (*q*, *J* = 32.4 Hz, *aromatic carbon*), 130.1 (*aromatic carbon*), 134.4 (*aromatic carbon*), 141.7 (*C-e*), 143.8 (*d*, *J* = 246.0 Hz, *C-q*)**, 143.9 (*d*, *J* = 246.9 Hz, *C-r*)**, 160.3 (*C-g*)***, 162.7 (*C-h*)***. MS (ESI) 468 (M – 1). ESI-HRMS (*m*/*z*): [M + H]^+^ calcd for C_21_H_11_F_7_N_3_O_2_, 470.0734; obsd, 470.0735.

### Ethyl 2-((*tert*-Butoxycarbonyl)oxy)pyrazolo[1,5-*a*]pyridine-3-carboxylate (**40**)

Cs_2_CO_3_ (2.86 g, 8.74 mmol) and *tert*-butoxycarbonyl anhydride (0.699 g, 3.2 mmol) were added to a solution of **18** (0.600 g, 2.91 mmol) in dry THF (25 mL). The reaction mixture was stirred under reflux overnight and allowed to reach room temperature. The solvent was concentrated under reduced pressure, and the residue was dissolved in water (50 mL) and extracted with diethyl ether (3 × 50 mL). The combined organic layer was washed with brine, dried over anhydrous Na_2_SO_4_, and concentrated under reduced pressure. The residue was purified by flash chromatography (eluent: petroleum ether/ethyl acetate 80:20 v/v) to afford the title compound as a white solid (mp 95.4–96.4 °C from trituration with diisopropyl ether). Yield 93%. ^1^H NMR (600 MHz, chloroform-*d*_3_) δ 1.39 (*t*, 3H, *J* = 7.1 Hz, -OCH_2_C*H*_3_), 1.58 (s, 9H, -OC(*CH*_3_)_3_), 4.36 (*q*, 2H, *J* = 7.0 Hz, -OC*H*_2_CH_3_), 6.96 (*t*, 1H, *J* = 6.8 Hz, *H-b*), 7.42 (*t*, 1H, *J* = 7.9 Hz, *H-c*), 8.08 (*d*, 1H, *J* = 8.8 Hz, *H-d*), 8.38 (*d*, 1H, *J* = 6.6 Hz, *H-a*). ^13^C NMR (151 MHz, chloroform-*d*_3_) δ 14.6 (-OCH_2_*C*H_3_), 27.8 (-C(*C*H_3_)_3_), 60.2 (-O*C*H_2_CH_3_), 84.8 (*C-f)*, 93.4(-*C*(CH_3_)_3_), 114.1 (*C-b*), 119.2 (*C-d*), 128.0 (*C-a*), 129.3 (*C-c*), 142.3 (*C-e*), 150.2 (*C-h*), 158.2 (*C-*g)*, 162.1 (*C-i*)*. MS (ESI) 307 (M + 1).

### Ethyl 2-((*tert*-Butoxycarbonyl)oxy)-7-chloropyrazolo[1,5-*a*]pyridine-3-carboxylate (**41**)

LiHMDS (1.0 M THF solution: 0.980 mL, 0.98 mmol, 1.5 equiv) was added dropwise to a solution of **40** (0.400 g, 0.654 mmol) in dry THF (10 mL) and cooled to −78 °C. The mixture was stirred at −78 °C for 1 h, and a solution of hexachloroethane (0.170 g, 0.72 mmol, 1.1 equiv) in dry THF was then added at −78 °C, and the reaction mixture was stirred for 15 min at room temperature. Subsequently, the reaction was quenched with an aqueous saturated solution of NH_4_Cl (100 mL). The water phase was extracted with dichloromethane (4 × 100 mL). The combined organic phases were dried over Na_2_SO4, filtered, and evaporated to dryness under vacuum. The crude product was purified by flash chromatography (eluent: petroleum ether/ethyl acetate 80:20 v/v) to afford the title compound as a white solid (mp 104.6–106.0 °C from trituration with diisopropyl ether). Yield 81%. ^1^H NMR (600 MHz, chloroform-*d*_3_) δ 1.40 (*t*, 3H, *J* = 7.1 Hz, -OCH_2_C*H*_3_), 1.58 (s, 9H, -OC(*CH*_3_)_3_), 4.37 (*q*, 2H, *J* = 7.2 Hz, -OC*H*_2_CH_3_), 7.11 (*dd*, 1H, *J* = 7.5, 1.1 Hz, *H-b*), 7.39 (*dd*, 1H, *J* = 8.9, 7.5 Hz, *H-c*), 8.09 (*dd*, 1H, *J* = 8.9, 1.2 Hz, *H-d*). ^13^C NMR (151 MHz, chloroform-*d*_3_) δ 14.6 (-OCH_2_*C*H_3_), 27.8 (-C(*C*H_3_)_3_), 60.5 (-O*C*H_2_CH_3_), 85.0 (*C-f*) _3_, 95.2(-*C*(CH_3_)_3_), 114.4 (*C-b*), 117.5 (*C-d*), 128.2 (*C-c*), 131.0 (*C-a*), 143.9 (*C-e*), 150.0 (*C-h*), 158.0 (*C-*g)*, 161.8 (*C-i*)*. MS (ESI) 241 (M + 1, -Boc).

### Ethyl 7-Chloro-2-hydroxypyrazolo[1,5-*a*]pyridine-3-carboxylate (**42**)

Trifluoroacetic acid (10 mL) was added to a solution of **41** in dry dichloromethane (25 mL), and the reaction mixture was stirred at room temperature for 4 h. The mixture was quenched with water, and the layers were separated. The aqueous solution was further extracted with dichloromethane (3 × 25 mL). The combined organic phases were dried over Na_2_SO_4_, filtered, and evaporated to dryness under reduced pressure. The crude product was purified by flash chromatography (eluent: dichloromethane/methanol 98:2 v/v) to afford the title compound as a white solid (mp 134.0–135.8 °C from trituration with diisopropyl ether). Yield 94%. ^1^H NMR (600 MHz, chloroform-*d*_3_) δ 1.44 (*t*, 3H, *J* = 7.2 Hz, -OCH_2_C*H*_3_), 4.44 (*q*, 2H, *J* = 7.2 Hz, -OC*H*_2_CH_3_), 7.02 (*dd*, 1H, *J* = 7.5, 1.2 Hz, *H-b*), 7.35 (*dd*, 1H, *J* = 8.7, 7.5 Hz, *H-c*), 7.72 (*dd*, 1H, *J* = 8.7, 1.2 Hz, *H-d*), 9.09 (*s*, 1H, -O*H*). ^13^C NMR (151 MHz, chloroform-*d*_3_) δ 14.6 (-OCH_2_*C*H_3_), 60.9 (-O*C*H_2_CH_3_), 87.9 (*C-f*), 113.6 (*C-b*), 115.5 (*C-d*), 128.4 (*C-c*), 131.3 (*C-a*), 142.0 (*C-e*), 166.0 (*C-h*)*, 166.9 (*C-*g)*. MS (ESI) 241 (M + 1).

### Ethyl 2-(Benzyloxy)-7-chloropyrazolo[1,5-*a*]pyridine-3-carboxylate (**43**)

Benzyl bromide (645 mg, 3.20 mmol, 1.10 equiv) was added dropwise to a mixture of **42** (600 mg, 2.91 mmol) and Cs_2_CO_3_ (2.295 g, 7.04 mmol, 2.4 equiv) in dry DMF (15 mL). The reaction mixture was stirred overnight at room temperature, and water (100 mL) was then added. The mixture was extracted with EtOAc (4 × 70 mL), and the combined organic layer was dried under Na_2_SO_4_ and evaporated under reduced pressure to give a yellow oil. The mixture was separated using flash chromatography (eluent: petroleum ether/EtOAc 6/4 v/v) to afford the title compound as a pale yellow solid (mp 98.2–99.3 °C from trituration with diisopropyl ether). Yield 85% ^1^H NMR (600 MHz, chloroform-*d*_3_) δ 1.41 (*t*, 3H, *J* = 7.1 Hz, -OCH_2_C*H*_3_), 4.38 (*q*, 2H, *J* = 7.1 Hz, -OC*H*_2_CH_3_), 5.58 (*s*, 2H, -OC*H*_2_Ph), 6.96 (*dd*, 1H, *J* = 7.4, 1.2 Hz, *H-b*), 7.27–7.34 (*m*, 2H, *H-c and aromatic proton*), 7.39 (*t*, 2H, *J* = 7.5 Hz, *aromatic protons*), 7.59 (*d*, 2H, *J* = 7.4 Hz, *aromatic protons*), 7.99 (*dd*, 1H, *J* = 8.8, 1.2 Hz, *H-d*). ^13^C NMR (151 MHz, chloroform-*d*_3_) δ 14.6 (-OCH_2_*C*H_3_), 60.0 (-O*C*H_2_CH_3_), 71.1 (-O*C*H_2_Ph), 90.1 (*C-f*), 112.8 (*C-b*), 116.5 (*C-d*), 127.8 (*aromatic carbon*), 127.9 (*aromatic carbon*), 128.0 (*C-c*), 128.5 (*aromatic carbon*), 130.6 (*C-a*), 136.7 (*aromatic carbon*), 144.6 (*C-e*), 163.1 (*C-h*)*, 164.8 (*C-*g)*. MS (ESI) 331 (M + 1).

### 2-(Benzyloxy)-7-chloropyrazolo[1,5-*a*]pyridine-3-carboxylic Acid (**44**)

6 M NaOH (5.0 equiv) was added to a solution of compound **43** (785 mg, 2.40 mmol) in EtOH abs (20 mL). The mixture was stirred for 4 h at 75 °C, then neutralized with 6 M HCl and concentrated under reduced pressure. The mixture was cooled to 0 °C and then acidified with 2 M HCl until pH 2 was reached, giving a suspension. This suspension was filtered to give the title compound as a white solid (mp 178.4–179.8 °C dec with gas developed, from trituration with diisopropyl ether). Yield 84%. ^1^H NMR (600 MHz, DMSO-*d*_6_) δ 5.48 (*s*, 2H, -OC*H*_2_Ph), 7.31 (*dd*, 1H, *J* = 7.5, 1.1 Hz, *H-b*), 7.35 (*t*, 1H, *J* = 7.4 Hz, *aromatic proton*), 7.41 (*t*, 2H, *J* = 7.4 Hz, *aromatic protons*), 7.52 (*dd*, 1H, *J* = 8.7, 7.6 Hz, *H-c*), 7.55 (*d*, 2H, *J* = 7.5 Hz, *aromatic protons*), 7.95 (*dd*, 1H, *J* = 8.9, 1.1 Hz, *H-d*), 12.34 (*br s*, 1H, -COO*H*). ^13^C NMR (151 MHz, DMSO-*d*_6_) δ 70.5 (-O*C*H_2_Ph), 89.4 (*C-f*), 113.3 (*C-b*), 116.1 (*C-d*), 127.9 (*C-a*), 128.1 (*aromatic carbon*), 128.4 (*aromatic carbon*), 128.9 (*aromatic carbon*), 129.4 (*C-c*), 136.4 (*aromatic carbon*), 144.1 (*C-e*), 163.3 (*C-h*)*, 164.0 (*C-*g)*. MS (ESI) 301 (M – 1).

### 1-Benzyl-7-chloro-2-oxo-*N*-(2,3,5,6-tetrafluoro-[1,1′-biphenyl]-4-yl)-1,2-dihydropyrazolo[1,5-*a*]pyridine-3-carboxamide (**45**)

Oxalyl chloride (3.0 mmol) and dry DMF (1 drop) were added to a cooled (0 °C) solution of **44** (1.0 mmol) 1–3, in dry THF (20 mL), under a nitrogen atmosphere. The obtained solution was stirred at room temperature for 2 h. The solution was then concentrated under reduced pressure and the residue dissolved in dry THF (10 mL, this step was repeated three times). The resulting acyl chloride was immediately used without any further purification and was dissolved in 10 mL of dry toluene and transferred to the solution described below. Trimethylaluminum (2.0 M in hexane, 1.5 mmol) was added to a solution of 4-phenyl-2,3,5,6-tetrafluoroaniline (1.5 mmol) in dry toluene (15 mL), under a nitrogen atmosphere. The resulting mixture was stirred for 2 h at room temperature, giving a brown suspension, and the solution of the previously described acyl chloride in dry toluene (30 mL) was then quantitatively added. The mixture was heated overnight at 90 °C and then cooled to rt. The reaction was quenched with 1 M HCl, the layers were resolved, and the aqueous phase was exhaustively extracted using EtOAc. The combined organic layer was washed with 1 M NaOH and brine, dried, and the solvent was evaporated under reduced pressure. The crude product was purified by flash chromatography (eluent: from petroleum ether/EtOAc 8:2 v/v to petroleum ether/EtOAc 4:6 v/v) to afford the title compound as a white solid (mp 201.1–202.4 °C from trituration with diisopropyl ether). Yield 38%.^1^H NMR (600 MHz, chloroform-*d*_3_) δ 5.74 (*s*, 2H, -NC*H*_2_Ph), 6.73 (*dd*, 1H, *J* = 7.5, 1.2 Hz, *H-b*), 7.06 (*dd*, 2H, *J* = 7.4, 1.7 Hz, *aromatic protons*), 7.22–7.30 (*m*, 3H, *aromatic protons*), 7.35 (*dd*, 1H, *J* = 8.8, 7.6 Hz, *H-c*), 7.42–7.53 (*m*, 5H, *aromatic protons*), 8.28 (*dd*, 1H, *J* = 8.8, 1.2 Hz, *H-d*), 10.01 (*s*, 1H, -N*H*); ^13^C NMR (151 MHz, chloroform-*d*_3_) δ 52.6 (-N*C*H_2_Ph), 89.2 (*C-f*), 114.6 (*C-b*), 115.9 (*t*, *J* = 16.3 Hz, *C-p*)*, 116.7 (*C-d*), 118.0 (*t*, *J* = 17.2 Hz, *C-s*)*, 127.2 (*aromatic carbon*), 127.6 (*C-a*), 128.6 (*aromatic carbon*), 128.7 (*aromatic carbon*), 129.1 (*aromatic carbon*), 129.2 (*aromatic carbon*), 130.4 (*C-c*), 130.6 (*aromatic carbon*), 133.7 (*aromatic carbon*), 133.8 (*aromatic carbon*), 142.8 (*dd*, *J* = 248.8, 15.1 Hz, *C-q*)**, 144.2 (*d*, *J* = 248.3 Hz, *C-r*)**, 150.3 (*C-e*), 161.2 (*C-g*)***, 167.5 (*C-h*)***. MS (ESI) 526 (M – 1).

### 7-Chloro-2-hydroxy-*N*-(2,3,5,6-tetrafluoro-[1,1′-biphenyl]-4-yl)pyrazolo[1,5-*a*]pyridine-3-carboxamide (**4**)

Thioanisole (240 μL, 1.90 mmol, 10.0 equiv) was added to a solution of **45** (100 mg, 0.19 mmol, 1.0 equiv) in TFA (2 mL). The mixture was heated at 70 °C for 3 h then cooled to rt. The mixture was partially concentrated, and the crude product was taken up with water giving a suspension that was filtered. The solid was washed with an additional amount of cold water. The resulting solid was triturated with diisopropyl ether to afford the title compound, in pure form, as a white solid (mp 259.3–260.4 °C dec from trituration with diisopropyl ether). Yield 64%. ^1^H NMR (600 MHz, DMSO-*d*_6_) δ 7.32 (*dd*, 1H, *J* = 7.5, 1.1 Hz, *H-b*), 7.53 (*dd*, 1H, *J* = 8.7, 7.5 Hz, *H-c*), 7.53–7.60 (*m*, 5H, *aromatic protons*), 8.02 (*dd*, 1H, *J* = 8.8, 1.1 Hz, *H-d*), 8.99 (*s*, 1H, -N*H*), 13.33 (*br s*, 1H, -O*H*). ^13^C NMR (151 MHz, DMSO-*d*_6_) δ 89.9 (*C-f*), 113.5 (*C-b*), 115.6 (*C-d*), 116.7 (*t*, *J* = 17.8 Hz, *C-p*)*, 117.4 (*t*, *J* = 17.7 Hz, *C-s*)*, 126.6 (*C-a*), 128.8 (*aromatic carbon*), 128.9 (*aromatic carbon*), 128.9 (*C-c*), 129.4 (*aromatic carbon*), 130.1 (*aromatic carbon*), 142.9 (*d*, *J* = 246.6 Hz, *C-q*)**, 143.2 (*d*, *J* = 241.3 Hz, *C-r*)**, 143.5 (*C-e*), 160.2 (*C-g*)***, 162.6 (*C-h*)***. MS (ESI) 436 (M + 1). ESI-HRMS (*m*/*z*): [M + H]+ calcd for C_20_H_11_ClF_4_N_3_O_2_, 436.0470; obsd, 436.0472.

### Molecular Modeling

#### Docking

The predicted binding modes shown herein were carried out using the Glide XP Docking Protocol.^48−50^ The protein structure of **1** in complex with *h*DHODH (PDB code 6FMD)^18^ was retrieved from the Protein Data Bank^51^ and prepared for the docking procedure. The crystal structure of the protein underwent a preparation process that was performed using the Protein Preparation Wizard tool,^52^ implemented in Maestro GUI.^53−55^ Missing hydrogen atoms were added, missing bonds or atoms were fixed, bond orders were assigned, and water molecules were removed. The prediction of protonation states for the protein was accomplished using PROPKA, with the pH set at 7.4.^56−59^ The docking procedure was validated by performing a self-docking protocol and verifying the correct positing of the cocrystallized ligand. The coordinates of the bound crystallographic ligand were used as the centroid of the grid box.

### Enzymatic Assays

#### Protein Expression and Purification

BL21DE3 PyrD *E. coli* cells were transformed using the plasmid construct pFN2A–*h*DHODH (kindly given by Department of Drug Science and Technology, University of Turin, Turin, Italy). The vector produces *h*DHODH as an N-terminal GST-fusion protein. Cells were grown at 37 °C in LB medium supplemented with 0.1 mM flavin mononucleotide (Cayman Chemical). After 20 h of growth, cells were induced with 0.8 mM isopropyl-d-thiogalactopyranoside at an OD_600_ of 0.5–0.7 at 28 °C for an additional 6 h. A cell pellet from 250 mL of culture was lysed in 20 mL of PBS (50 mM Na_2_HPO_4_, 50 mM NaH_2_PO_4_, 500 mM NaCl), which had been supplemented with 24 mg of lysozyme and 0.2% v/v protease inhibitor cocktail, incubated for 30 min over ice, and disrupted by sonication (total sonication time: 8 min with on/off cycles of 10″/50″). Triton X-100 was added to the lysate, to a final concentration of 1%, before centrifugation at 14 000*g* for 40 min at 4 °C. The clarified supernatant was incubated with DNase I for 30 min at room temperature, supplemented with 2 mM dithiothreitol (DTT), and filtered through a 0.45 μm syringe filter as previously described by Sainas et al.^18^ The GST-fused enzyme was purified from the bacterial lysate using affinity chromatography on immobilized glutathione-Sepharose columns (GE- HiTrap Protein G HP 1 mL). The GST tag was not cleaved for further analysis. All the reagents used in the protein expression and purification were supplied by Merck/Sigma-Aldrich if not otherwise specified.

#### *h*DHODH Inhibition Assay

The enzymatic inhibition assay was optimized for being performed on a 96-well plate and to achieve higher throughput. For each well of the plate a total volume of 200 μL was used: 5 μL of purified GST-*h*DHODH; 60 μL of 2,6-dichloroindophenol (DCIP) 50 μM; 20 μL of coenzyme Q10 enzyme 100 μM; 20 μL of dihydroorotate (DHO) 500 μM; Tris-HCl, pH 8, up to a final volume of 200 μL. Inhibitory activity was assessed by monitoring the reduction of DCIP, which is associated with the oxidation of dihydroorotate as catalyzed by the DHODH enzyme. The enzyme was preincubated for 5 min at 37 °C in Tris-HCl, pH 8, with coenzyme Q10, with DCIP (50 μM), and with the compounds to be tested used at different concentrations (final DMSO concentration 0.1% v/v). The reaction was initiated by the addition of DHO (500 μM), and the absorbance kinetic reduction was monitored at λ = 650 nm using a multiplate reader (Tecan, M1000Pro). In order to assess the minimum and maximum absorbance values of the enzymatic reaction, a Min control value was obtained by measuring the absorbance without DHO. Similarly, a Max value was obtained by measuring the absorbance with DHO but no inhibitor. A blank reduction calculation was also performed by measuring the absorbance values using 180 μL of Tris-HCl and 20 μL of coenzyme Q10. The instrument was set to read the absorbance values every 10 s for a total read time of 10 min at 37 °C. The initial rate was measured in the first 5 min (ε = 10 400 M^–1^ cm^–1^), and an IC_50_ value was calculated,^60^ using GraphPad Prism 7 software. Values are the mean ± SE of three independent experiments.

### Cell-Based Assay

#### Cell Lines and Drug Treatment

Human cells, THP1 (acute monocytic leukemia), U937 (acute monocytic leukemia), and Jurkat (T cell leukemia) were cultured in complete RPMI 1640 (Invitrogen Life Technologies, Gaithersburg, MD), supplemented with 10% heat-inactivated fetal bovine serum (FBS) and 1% penicillin/streptomycin (GIBCO, Invitrogen, Milan, Italy). Each compound was solubilized in DMSO (Sigma-Aldrich, Milan, Italy) at a final concentration of 10 mM, which was used as the stock solution for all experiments. Final dilutions were made in culture medium.

#### CFSE-Based Cytotoxic Activity Assay

Briefly, the Jurkat cell line was incubated with 1 μM carboxyfluorescein diacetate succinimidyl ester dye (CFSE, Vybrant CFDA SE cell tracer kit; Molecular Probes, Invitrogen Carlsbad, CA), at 10^7^/mL for 20 min at 37 °C. At the end of the labeling process, cells were resuspended and washed in RPMI-1640 supplemented with 1% fetal bovine serum. Cells were then resuspended in RPMI 1640 supplemented with 10% FBS and incubated for 20 min at 37 °C. Cells were centrifuged and plated (1 × 10^4^ in 200 μL of medium), with increasing concentrations of the DHODH inhibitors (1 μM to 100 μM), for 3 days. Cells were harvested, and 1 μg/mL of propidium iodide was added to assign the ratio of cell death. The percentage of specific lysis was calculated as described and in accordance with the following equation: [dead targets in sample (%) – spontaneously dead targets (%))/(100 – spontaneously dead targets (%))] × 100. Spontaneous release was obtained by incubating cell lines in medium supplemented with the corresponding percentage of DMSO used for the dilution of compounds. Values represent the concentration that induces significant cytotoxic effects (≥30%).

#### Annexin Assay

For compound screening, 1 × 10^4^ THP1 cells were plated in 96-well round-bottom plates and *h*DHODH inhibitors were added, at 0.1 μM or 1.0 μM, in a volume of 200 μL of medium. For the determination of EC_50_, 1 × 10^4^ THP1 or U937 cells were plated in 96-well round-bottom plates and treated with increasing doses of DHODH inhibitors from 0.001 μM to 10 μM. After 3 days of culture, the apoptotic assay was performed using the annexin V-FITC kit (Miltenyi Biotec, Italy), according to the manufacturer’s instructions. The apoptotic cells were acquired on FacsVerse and analyzed using Kaluza software version 1.2 (Beckman Coulter, Fullerton, CA). The annexin assay was also performed in the presence of uridine 100 μM (Sigma-Aldrich, Milan, Italy).^61^

#### Differentiation Assay

For compound screening, 1 × 10^4^ THP1 cells were plated in 96-well round-bottom plates and the *h*DHODH inhibitors were added, at 0.1 μM or 1.0 μM, in a 200 μL volume of medium. For the determination of EC_50_, 1 × 10^4^ THP1 or U937 cells were plated in 96-well round-bottom plates and treated with increasing doses of the *h*DHODH inhibitors from 0.001 μM to 10 μM. After 3 days of culture, the differentiation pathway was monitored by analyzing the expression of CD14 (THP1) and CD11b (U937) via flow cytometry analysis. Cells were washed and resuspended in staining buffer (phosphate buffered saline, 2% bovine serum albumin, 1 mM EDTA) and incubated with CD14-PE (Beckam Coulter, CA, USA) or CD11b-PE (BD Bioscience, San Jose, CA, USA) at 4 °C for 45 min. Samples were acquired on a FACSVerse (BD-Biosciences, San Jose, CA), and dead cells were excluded from the analyses, according to the use of propidium iodide (Sigma-Aldrich, Milan, Italy). Data were processed using Kaluza software version 1.2 (Beckman Coulter, Fullerton, CA). The differentiation assay was also performed in the presence of uridine 100 μM.

#### Statistical Analysis

Statistical analyses were performed on Prism software, version 5.0 (GraphPad Software, San Diego, CA). Data are reported as the mean ± SD. Two-tail paired Student’s *t* tests were calculated to assess the differences between mean values, and *P* < 0.05 was considered significant. For the determination of EC_50_, a nonlinear regression model was applied.

### Preliminary ADME and Chemophysical Profiling

#### Solubility Assay at pH 7.4

Solubility was assayed in phosphate buffered saline (PBS: 12 mM with NaCl 137 mM and KCl 2.7 mM, pH 7.4). Each solid compound (1 mg) was added to 1 mL of PBS. The samples were shaken in an orbital shaker at 25 °C for 24 h. These suspensions were filtered through a PTFE 0.45 μm filter (VWR), and the solutions were chromatographically analyzed using a PerkinElmer ultrahigh performance liquid chromatography (UHPLC) instrument, equipped with a reverse-phase (RP) C18 Phenomenex column (2.1 mm × 100 mm, 1.7 μm particle size). Gradient elution: the ratio of eluents A and B (0.1% trifluoroacetic acid in water and 0.1% trifluoroacetic acid in acetonitrile, respectively) changed linearly from 60% A–40% B to 0% A–100% B in 12 min, followed by 5 min in isocratic elution at 100% of eluent B and then 4 min in equilibration elution to reset the starting conditions. The flow rate was 0.5 mL/min. Standard injection volumes were either 2 or 4 μL for poorly soluble compounds. The detection system was a PerkinElmer diode array detector. The wavelengths that were monitored for each compound were defined according to the compound’s own absorption spectrum. Solubility, expressed as μM concentration of the saturated solution, was calculated via interpolation with external calibration curves that were obtained with solutions of each compound in acetonitrile.

#### ClogP and log *D* (pH 7.4)

ClogP values were calculated using the Bio-Loom program for Windows, version 1.5 (BioByte). The partition coefficients between *n*-octanol and PBS at pH 7.4 (LogD^7.4^) were obtained using the shake-flask technique at room temperature. In the shake-flask experiments, 50 mM phosphate buffered saline, pH 7.4, was used as the aqueous phase. The organic (*n*-octanol) and aqueous phases were mutually saturated by shaking for 4 h. The compounds were solubilized in the buffered aqueous phase at the highest concentration compatible with solubility, and appropriate amounts of *n*-octanol were added. The two phases were shaken for about 20 min, by which time the partitioning equilibrium of solutes had been reached, and then centrifuged (10 000 rpm, 10 min). The concentration of the solutes was measured in the aqueous phase using a UV spectrophotometer (Varian Cary 50BIO); absorbance values (recorded for each compound at the wavelength of maximum absorption) were interpolated in calibration curves obtained using standard solutions of the compounds (*r*^2^ > 0.99). Each log *D* value is an average of at least six measurements.

#### Protein Binding *in Vitro*

This was achieved via ultrafiltration using commercially available membrane systems (Centrifree ultrafiltration devices with ultracel YM-T membrane, Merck). A solution of the selected compound in DMSO was added to human serum (sterile-filtered from human male AB plasma, Sigma-Aldrich) to give the final concentration of 50 μM with 2% of cosolvent. 1 mL of the solution obtained in the sample reservoir of the ultrafiltration device was gently shaken in an orbital shaker at 37 °C for 1 h. The tube was then centrifuged at 1000*g* for 15 min. The concentrations of the compounds in the ultrafiltrate and filtrate were determined using reverse-phase UHPLC, and the chromatographic conditions were those described above with different injection volumes: 20 μL for ultrafiltrate samples and 2 μL for filtrate samples. The quantitation of the compounds in the filtrate and in ultrafiltrate was performed using two different calibration curves of compound standard solutions (linearity determined in concentration ranges of 0.5–25 μM with injection volume of 20 μL for ultrafiltrate and 10–100 μM with injection volume of 2 μL for filtrate; *r*^2^ > 0.99). The recovery of the ultrafiltration process was calculated in order to discover whether any compound was lost during ultrafiltration, in view of the limited solubility of tested compounds.
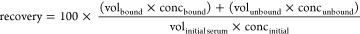
vol_bound_ is calculated by dividing the weight of the bound fraction (difference between the weights of the sample reservoir after ultrafiltration and empty) by its density (0.991 g/mL assessed by weighing five replicates of a known volume of the bound fraction). vol_unbound_ is calculated by dividing the weight of the unbound fraction (difference between the weights of the ultrafiltrate cup after and before ultrafiltration) by its density (0.999 g/mL assessed by weighing five replicates of a known volume of the unbound fraction). conc_bound_ is calculated using the RP-HPLC method. conc_unbound_ is calculated using the RP-HPLC method (calibration with standard additions). Average recovery was 90% for all tested compounds.

#### *In Vivo* Toxicity Assays

All procedures have been described previously.^62^ Briefly, 12 female Balb/c mice (4–5 weeks old, 16–22 g of weight) were randomly assigned to 3 groups (4 mice in each group) and animals were given access to food and water *ad libitum*. The control group was intraperitoneally inoculated with the vehicle (every 3 days, ip), for 35 days). The other two groups of mice received 10 and 25 mg/kg of **1** (every 3 days, ip, for 35 days) and were weighed before treatment. Compound **1** was dissolved in 50% PBS, 25% Cremophor (Kolliphor EL) and 25% ethanol (vehicle). At the end point of the study, we examined the following parameters: mortality, clinical signs, body weight, food consumption (every week), hematology, and serum biochemistry parameters. The organs (liver, heart, lung, kidney) were collected at sacrifice and fixed in 4% buffered formaldehyde for histological analyses. At the time of sacrifice, 1 mL of blood from each mouse was collected from the heart, using a hypodermic syringe, stored in a tube containing heparin, and hematological and biochemical analyses were performed. All results were compared to standard parameters for normal mouse blood. These analyses were performed at the Veterinary Analysis Laboratory (Turin, Italy). All animal procedures were approved by the Ethics Committee at the University of Turin and by the Italian Ministry of Health (MoH), in compliance with international laws and policies (MoH Approved Project No. 337/216-PR).

### *In Vitro* Metabolic Behavior

#### Incubation Conditions of Rat Microsomes and Sample Preparation

Rat-liver microsomes (Sprague-Dawley, male, Sigma-Aldrich; 20 mg/mL protein concentration) were incubated with the candidate compound solution (5 μM final concentration, with 1% DMSO) and Tris buffer (0.1 M, pH = 7.4). The regenerating system, which slowly generated coenzyme units over the incubation time, leading to a better reproduction of *in vivo* behavior, was composed of MgCl_2_ (3.3 mM), NADP^+^ (1.3 mM), Glu6P (3.5 mM), and Glu6P dehydrogenase (0.5 U/mL). In addition to the compound sample (“C”) that was incubated with active microsomes and regenerating system, drug-free matrix blank sample (B) and two other series of specimens were used to provide more information for the interpretation of experimental results:

- In the “C1” control sample, the tested drug was incubated with heat-inactivated microsomes (inactivation via a 10 min heating cycle at 90 °C).
- In the “C2” control sample, there was no regenerating system in the incubation medium.

The incubation time started with the addition of the microsomes suspension (0.5 mg/mL). Time point *t*_0_ was immediately collected, and the following samples were collected at 15, 30, 60, and 120 min in order to evaluate short-term stability and longer-term stability.

Metabolic reactions were stopped by adding 100 μL of cooled acetonitrile to the 100 μL sample of the incubation mixture. Samples were centrifuged to provoke protein precipitation, and the supernatants were immediately stocked at −80 °C, until analysis, to prevent the potential degradation of unstable products.

#### Identification of Metabolites Using High-Resolution Mass Spectrometry

The products of *in vitro* metabolism were identified using a high-resolution mass spectrometer (Q-Exactive Orbitrap, Thermo Scientific) coupled to an HPLC instrument (1200 system Agilent). All analytes were separated on an Ascentis C18 column (150 mm × 2.1 mm, 5 μm particle size) maintained at 35 °C. The elution mixture was composed of solvent A (0.1% formic acid in water for positive ionization mode and 0.05% acetic acid for negative ionization mode) and solvent B (acetonitrile). The elution gradient was from 1% to 99% of solvent B in 24 min; held at 99% for 4 min and re-equilibrated for 6 min at 1% of solvent B. The injection volume and flow rate were 4 and 180 μL/min, respectively. Mass spectrometric analyses were performed in positive- and negative-ion mode using a HESI II source under the following conditions: heated capillary temperature 320 °C, spray voltage 3 kV (positive ions) or −2.2 kV (negative ions), auxiliary gas temperature 160 °C, flow rate of 6 (arbitrary units), sheath-gas flow rate 32 (arbitrary units), sweep-gas flow rate 2 (arbitrary units). Accurate mass measurements were obtained using full-scan mass spectra (resolving power *R* = 70 000; mass range *m*/*z* 105–850 Da) and with data dependent MS2 acquisition, in which the four most abundant ions of the previous full-scan spectrum were selected for fragmentation. After a first explorative analysis, samples were reanalyzed using a data-independent (DIA) method, in which the molecular ions that corresponded to the metabolites that were found were always selected for MS2 fragmentation. This allowed the acquisition of MS2 spectra even for metabolites present in very low concentrations. MS2-DIA scans were acquired at *R* = 17 500 and with variable collision energies ranging from 30 to 45, depending on the *m*/*z* of the parent ion. Results from the DIA analyses allowed the main characteristic fragments for each metabolite to be identified.

## Supporting Information Available

Molecular formula strings and biological data (CSV)
Synthesis of compounds **36**–**39** and characterization, MS2 spectra of hydroxylated metabolites of **1**, a table with docking scores of compounds **4**–**17**, *in vivo* toxicity figures, and ^1^H NMR spectra, ^13^C NMR spectra, and HRMS of representative compounds (PDF)

## Abbreviations Used

CFSE: carboxyfluorescein diacetate succinimidyl ester
DCIP: dichloroindophenol
DHO: dihydroorotate
DTT: dithiothreitol
ETC: electron transport chain
FEP: free energy perturbation
FMN: flavin mononucleotide
*h*DHODH: human dihydroorotate dehydrogenase
MD: molecular dynamics
TFA: trifluoroacetic acid;
