# Supplementary material for: Targeting Acute Myelogenous Leukemia Using Potent Human Dihydroorotate Dehydrogenase Inhibitors Based on the 2-Hydroxypyrazolo[1,5-a]pyridine Scaffold: SAR of the Biphenyl Moiety
Source: J Med Chem. 2021 Apr 12;64(9):5404–28. doi: 10.1021/acs.jmedchem.0c01549 (PMC8279415; doi:10.1021/acs.jmedchem.0c01549)

Supporting Information

Targeting Acute Myelogenous Leukemia using potent *human* dihydroorotate dehydrogenase inhibitors based on the 2-hydroxypyrazolo[1,5-*a*]pyridine scaffold: SAR of the biphenyl moiety

Stefano Sainas,[†, #] Marta Giorgis,[†,#] Paola Circosta,[‡, §] Valentina Gaidano[‡, ‖] Davide Bonanni,[†] Agnese C. Pippione,[†] Renzo Bagnati,[⊥] Alice Passoni,[⊥] Yaqi Qiu,[∇, ○] Carina Florina Cojocaru,[∇] Barbara Canepa,[■] Alessandro Bona,[□] Barbara Rolando,[†] Mariia Mishina,[†] Cristina Ramondetti,[¥] Barbara Buccinnà,[¥] Marco Piccinini,[¥] Mohammad Houshmand,[‡, §] Alessandro Cignetti,[œ] Enrico Giraudo,[†, ∇] Salam Al-Karadaghi,[¡] Donatella Boschi,[†] Giuseppe Saglio[‡, œ] and Marco L. Lolli[†*]

† Department of Drug Science and Technology, University of Turin, Via P. Giuria 9, 10125 - Turin, Italy;
‡ Department of Clinical and Biological Sciences, University of Turin, Regione Gonzole 10 - 10043 Orbassano (Turin), Italy;
§ Molecular Biotechnology Center, University of Turin, Via Nizza 52, 10126 - Turin, Italy;
∥ Division of Hematology, AO SS Antonio e Biagio e Cesare Arrigo, Via Venezia 16, 15121 - Alessandria, Italy;
⊥ Department of Environmental Health Sciences, Istituto di Ricerche Farmacologiche Mario Negri IRCCS, Via Mario Negri 2 - 20156 Milano, Italy;
∇ Laboratory of Tumor Microenvironment, Candiolo Cancer Institute – FPO, IRCCS, Candiolo, Strada Provinciale, 142 - KM 3.95, 10060 - Candiolo (Turin), Italy;
○ Higher Education Mega Center, Institutes for Life Sciences, South China University of Technology, Guangzhou, China;
■ Gem Forlab srl, Via Ribes, 5, 10010 - Colleretto Giacosa (Turin), Italy;
Gem Chimica srl, Via Maestri del Lavoro, 25, 12022 - Busca (Cuneo), Italy;
¥ Department of Oncology, University of Turin, Via Michelangelo 27/B, 10125 - Turin, Italy;
¡ Department of Biochemistry and Structural Biology, Lund University, Naturvetarvägen 14, Box 124, 221 00 Lund, Sweden;
œ Division of Hematology and Cell Therapy, AO Ordine Mauriziano, Largo Filippo Turati, 62, 10128 - Turin, Italy.
# contributed equally

Table of Content

Page S2	Synthesis and characterization of anilines **36 - 39**
Page S3	MS2 spectra of hydroxylated metabolites of **1**
Page S3	Table of docking scores
Page S4 – S5	In vivo Toxicity
Page S6 – S97	^{1}H-NMR, ^{13}C-NMR spectra, HRMS and UHPLC of representative compounds

*General procedure for the synthesis of aniline **36 - 39.***

$Pd(PPh_3)_4$ (90 mg, 0.08 mmol, 0.20 eq) was added to a solution of 4-bromo-2,3,5,6-tetrafluoroaniline (200 mg, 0.38 mmol, 1.00 eq) and K_2CO_3 (158 mg, 1.14 mmol, 3.00 eq) in 1,2-dimethoxyethane (35 mL). After stirring the resulting mixture under atmosphere of nitrogen for 1 h at r.t. the corresponding boronic acid (0.760 mmol, 2.0 eq) was added; the reaction mixture was then heated at reflux under atmosphere of nitrogen. After 2 h, an additional amount of boronic acid (0.38 mmol, 1.0 eq) was added, the reaction mixture was heated at reflux for another 2h before cooling to room temperature and concentrating under reduced pressure. The crude material was *taken-up* with water (100 mL) and the mixture was extracted with EtOAc (3 x 60 mL), the combined organic layers were dried over Na_2SO_4 and concentrated under reduced pressure. The crude product was purified by flash chromatography (see below the conditions).

2,3,4',5,6-pentafluoro-[1,1'-biphenyl]-4-aniline (**36**). Flash chromatography (eluent: *petroleum ether* / EtOAc from 95:5 v/v). White solid (m.p. 134.1 – 134.7 °C from trituration with diisopropyl ether). Yield 74%. ^{1}H NMR (600 MHz, Chloroform-*d*): δ 4.05 (*s*, 2H, -NH_2), 7.11 – 7.17 (*m*, 2H, *aromatic protons*), 7.37 – 7.42 (*m*, 2H, *aromatic protons*); ^{13}C NMR (151 MHz, $CDCl_3$): δ 107.3 (*t*, J = 16.8 Hz), 115.7 (*d*, J = 21.7 Hz), 124.0 (*d*, J = 1.7 Hz), 125.6 (*t*, J = 14.1 Hz), 132.2 (*d*, J = 7.7 Hz), 136.9 (*d*, J = 238.0 Hz), 144.1 (*d*, J = 243.5 Hz), 162.7 (*d*, J = 248.3 Hz). MS (ES-) 258 (M-1).

2,3,3',5,6-pentafluoro-[1,1'-biphenyl]-4-aniline (**37**). Flash chromatography (eluent: *petroleum ether* / EtOAc from 95:5 v/v). White solid (m.p. 108.8 – 109.7 °C from trituration with diisopropyl ether). Yield 90%. ^{1}H NMR (600 MHz, Chloroform-*d*): δ 4.08 (*s*, 2H, -NH_2), 7.10 (*t*, 1H, J = 8.4 Hz *aromatic proton*), 7.15 (*d*, 1H, J = 9.7 Hz, *aromatic protons*), 7.21 (*d*, 1H, J = 7.5 Hz, *aromatic proton*), 7.38 – 7.44 (*m*, 1H, *aromatic proton*); ^{13}C NMR (151 MHz, $CDCl_3$): δ 107.0 (*t*, J = 17.6 Hz), 115.3 (*d*, J = 20.8 Hz), 117.5 (*d*, J = 22.3 Hz), 125.9 (*t*, J = 14.2 Hz), 126.2, 130.0 (*d*, J = 8.3 Hz), 130.1, 136.8 (*dd*, J = 238.4, 16.2 Hz), 144.1 (*d*, J = 243.9 Hz), 162.7 (d, J = 245.9 Hz). MS (ES-) 258 (M-1).

2,3,5,6-tetrafluoro-4'-(trifluoromethyl)-[1,1'-biphenyl]-4-aniline (**38**). Flash chromatography (eluent: *petroleum ether* / EtOAc from 95:5 v/v). White solid (m.p. 163.9 – 164.8 °C from trituration with diisopropyl ether). Yield 60%. ^{1}H NMR (600 MHz, Chloroform-*d*): δ 4.12 (*s*, 2H, -NH_2), 7.56 (*d*, 2H, J = 8.0 Hz, *aromatic protons*), 7.71 (*d*, 2H, J = 8.2 Hz, *aromatic protons*); ^{13}C NMR (151 MHz, $CDCl_3$): δ 106.8 (*t*, J = 15.1 Hz), 124.1 (*q*, J = 271.7 Hz, -*C*F_3), 125.5 (*q*, J = 6.7 Hz), 126.4 (*t*, J = 3.8 Hz), 130.4 (*q*, J = 32.7 Hz), 130.8, 132.0, 136.9 (*d*, J = 238.7 Hz), 144.2 (*d*, J = 244.2 Hz). MS (ES-) 308 (M-1).

2,3,5,6-tetrafluoro-3'-(trifluoromethyl)-[1,1'-biphenyl]-4-aniline (**39**). Flash chromatography (eluent: *petroleum ether* / EtOAc from 95:5 v/v). Pale yellow solid (m.p. 46.6 – 47.0 °C from trituration with diisopropyl ether). Yield 92%. ^{1}H NMR (600 MHz, Chloroform-*d*): δ 4.11 (*s*, 2H, -NH_2), 7.55 -7.60 (*m*, 1H, *aromatic proton*), 7.61 (*d*, 1H, J = 7.2 Hz, *aromatic proton*), 7.65 (*d*, 1H, J = 7.4 Hz, *aromatic proton*), 7.70 (*s*, 1H, *aromatic proton*); ^{13}C NMR (151 MHz, $CDCl_3$): δ 106.7 (*t*, J = 16.6 Hz), 124.1 (*q*, J = 272.7 Hz, -*C*F_3), 125.2 (*q*, J = 3.8 Hz), 126.3 (*t*, J = 14.3 Hz), 127.3, 129.0, 129.1, 131.5 (*q*, J = 32.7 Hz), 133.8, 136.9 (*dd*, J = 239.4, 16.5 Hz), 144.2 (*d*, J = 244.2 Hz). MS (ES-) 308 (M-1).

Hydroxy-derivates of **1**

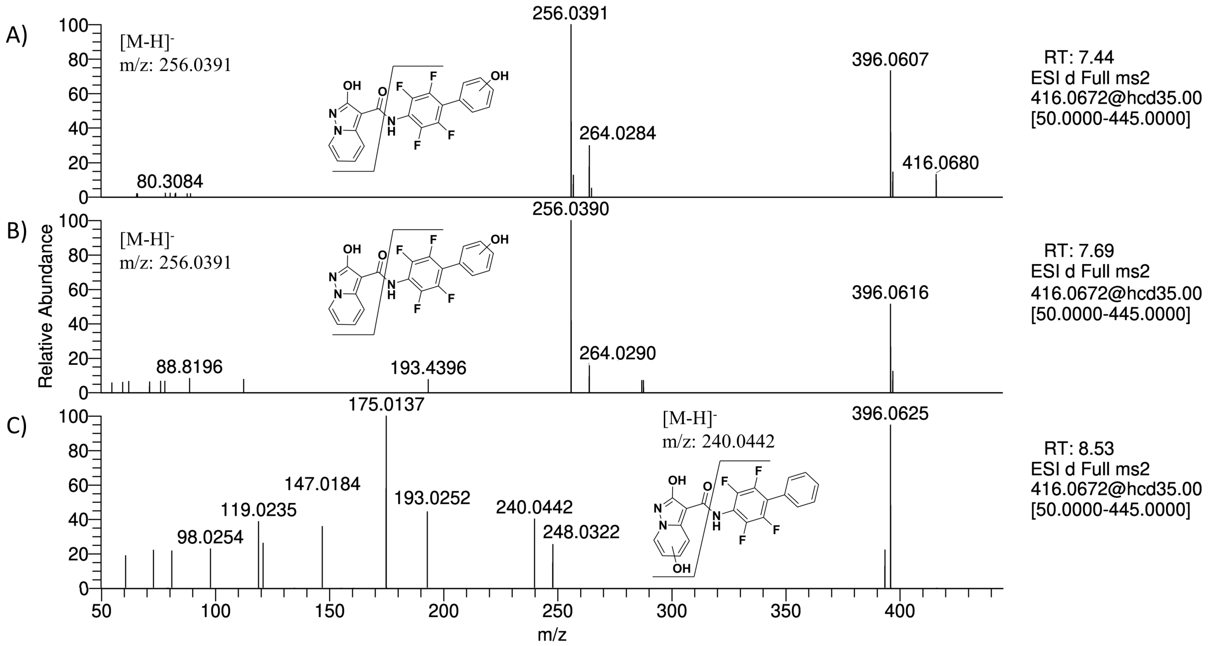

Figure S1. MS2 spectra (CID 35 eV, resolving power 17,500) of hydroxylated metabolites of **1** acquired in sample C, incubated with microsomes and regenerating system for 6 hours. Panel A and B show the product ion spectrum of hydroxyl-derivate metabolite of **1** at retention time 7.44 and 7.69 minutes, respectively. Panel C show the product ion spectrum of hydroxyl-derivate metabolite of 1 at retention time 8.61 minutes.

Compound	Docking score
**1**	-14,677
**5** (#500)	-13.716
**6** (#609)	-10.915
**7** (#607)	-13,873
**8** (#604)	-11,977
**9** (#610)	-12,47
**10** (#602)	-14,474
**11** (#601)	-14,668
**12** (#603)	-14,473
**13** (#600)	-14,743
**14** (#614)	-15,327
**15** (#608)	-14,855
**16** (#606)	-14,814
**17** (#613)	-15,559

Table S1: docking scores

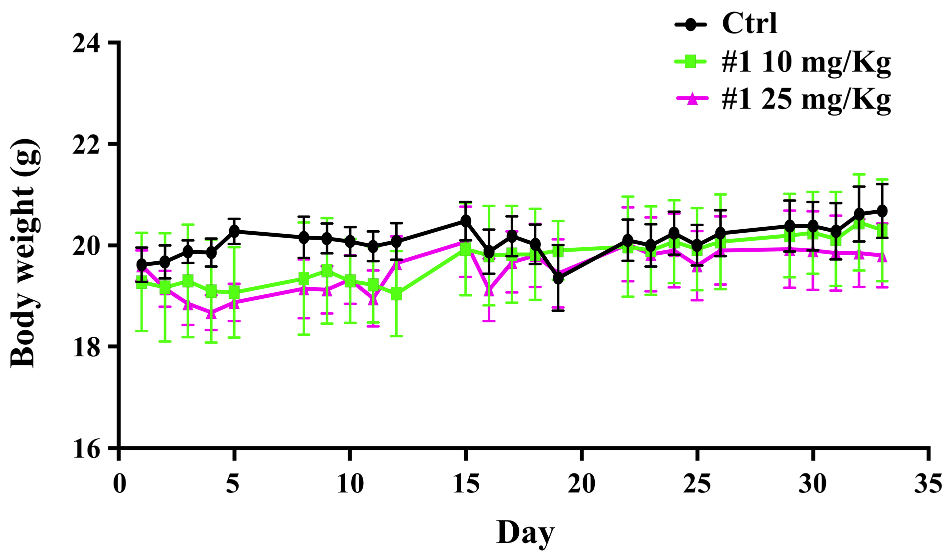

Figure S2. Body weight analysis of compound **1**–treated mice. Mean body weights at different time-point after 35 days of intraperitoneal injection of 10 and 25 mg/kg of compound **1** (every 3 days, i.p, n = 4/group) in healthy Balb/c mice.

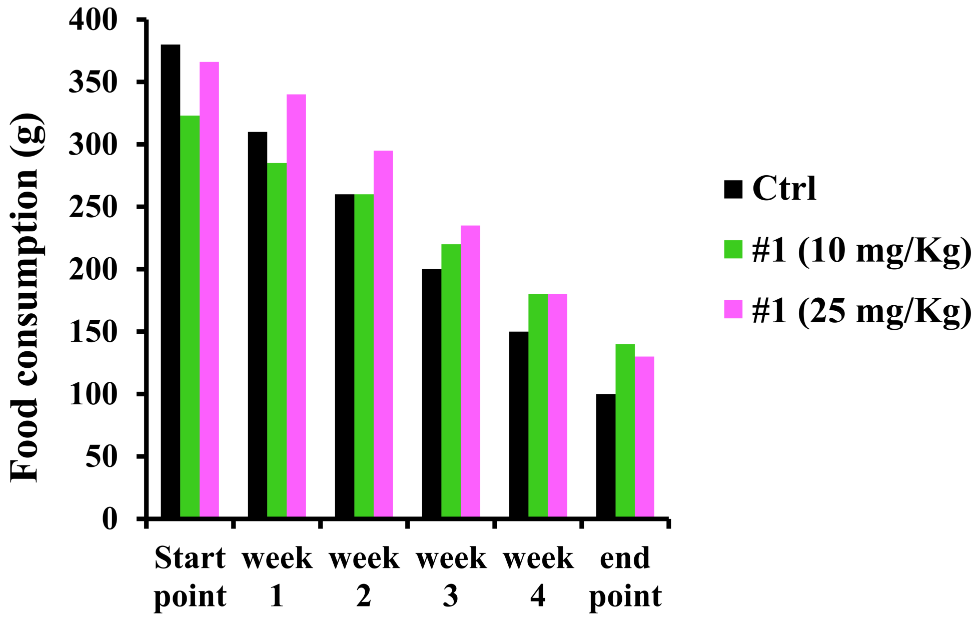

Figure S3. Food uptake of compound **1**–treated mice. Food consumption evaluated every week. Balb/c were treated with 10 and 25 mg/kg of compound **1** (every 3 days, i.p, n = 4/group). Control mice were treated with vehicle (every 3 days, i.p, n = 4/group).

	Control (vehicle)	1 (10 mg/kg)	1 (25 mg/kg)	UdM
**RBC**	10.23	10,10	10,34	M/uL
**HCT**	48.8	4.,5	48.9	%
**HGB**	15.2	15.0	14.8	g/dL
**MCV**	47.7	49.0	47.3	fL
**MCH**	14.9	14.9	14.3	pg
**MCHC**	31.1	30.3	30.3	g/dL
**RDW**	26.3	25.6	26.2	%
**RET%**	4.3	3.8	3.4	%
**RET**	438.9	380.8	350.5	K/uL
**WBC**	4.04	4.83	5.28	K/uL
**NEU%**	25.1	25.5	20.4	%
**LYM%**	64.6	65.8	71.4	%
**MON%**	7.4	6.8	5.9	%
**EOS%**	2.2	1.9	2.1	%
**BAS%**	0.7	0.0	0.2	%
**NEU**	1.01	1.23	1.08	K/uL
**LYM**	2.61	3.18	3.77	K/uL
**MON**	0.30	0.33	0.31	K/uL
**EOS**	0.09	0.09	0.11	K/uL
**BAS**	0.03	0.00	0.01	K/uL
**PLT**	258	262	323	K/uL
**BUN**	29	34	37	mg/dL
**Creatinine**	0.43	0.48	0.47	mg/dL
**ALT**	28	24	30	U/L

Table S2. Hematological profiling and biochemical analysis of renal and hepatic function parameters. Analysis of the blood samples of compound **1**–treated mice (10 and 25 mg/Kg, every 3 days, i.p, n = 4/group) compared with the controls. RBC, red blood cells; HGB, hemoglobin; HCT, hematocrit; MCV, mean corpuscular volume; RDW, red cell distribution width; RET, reticulocytes; WBC, white blood cells; NEU Neutrophil; LYM, lymphocyte; MON, monocyte; EOS, eosinophil; BAS, basophil; PLT, platelets; MCH, mean corpuscular hemoglobin; MCHC, mean corpuscular hemoglobin concentration. BUN, blood urea nitrogen (parameter of kidney and liver function); Creatinine (parameter of kidney function); ALT, alanine aminotransferase, parameter of liver function

^{1}H-NMR, ^{13}C-NMR spectra, HRMS and UHPLC of representative compounds

Ethyl 2-((4-methoxybenzyl)oxy)pyrazolo[1,5-a]pyridine-3-carboxylate (**19a**)

^{1}H NMR Spectrum (CDCl$_3$)

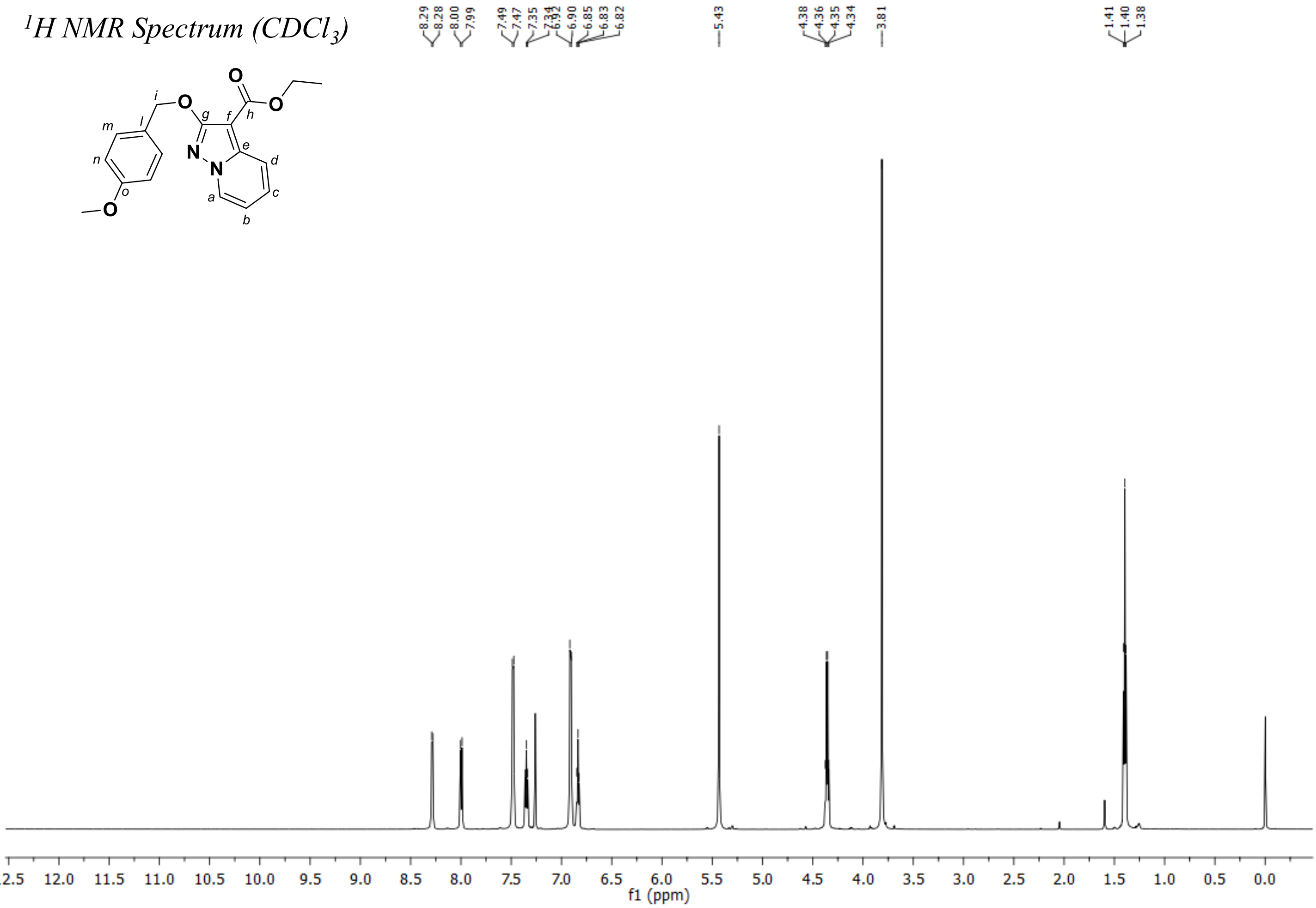

^{13}C NMR Spectrum (CDCl$_3$)

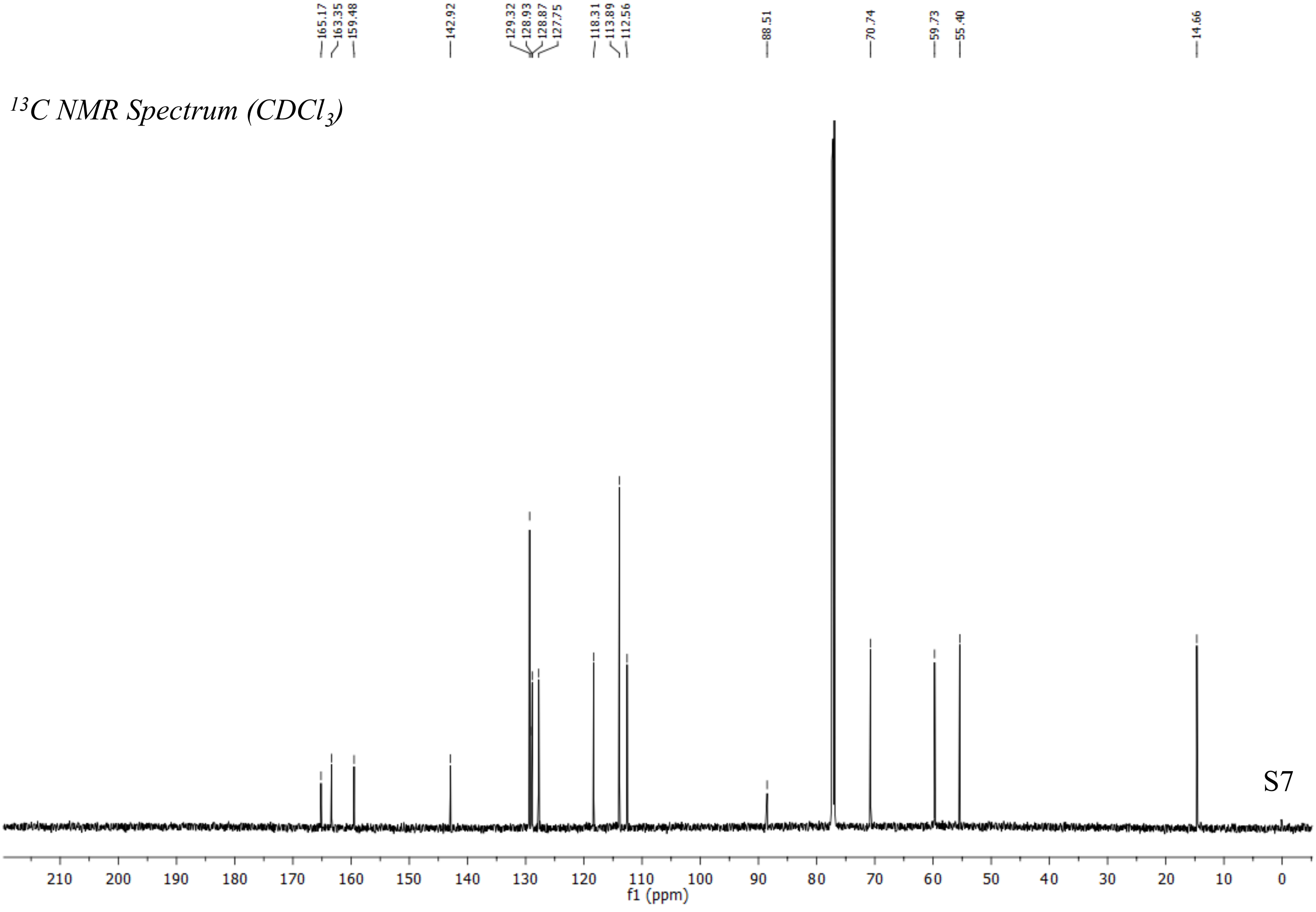

Ethyl N-(4-methoxybenzyl)-2-oxo-pyrazolo[1,5-a]pyridine-3-carboxylate (**19b**)

^{1}H NMR Spectrum (DMSO)

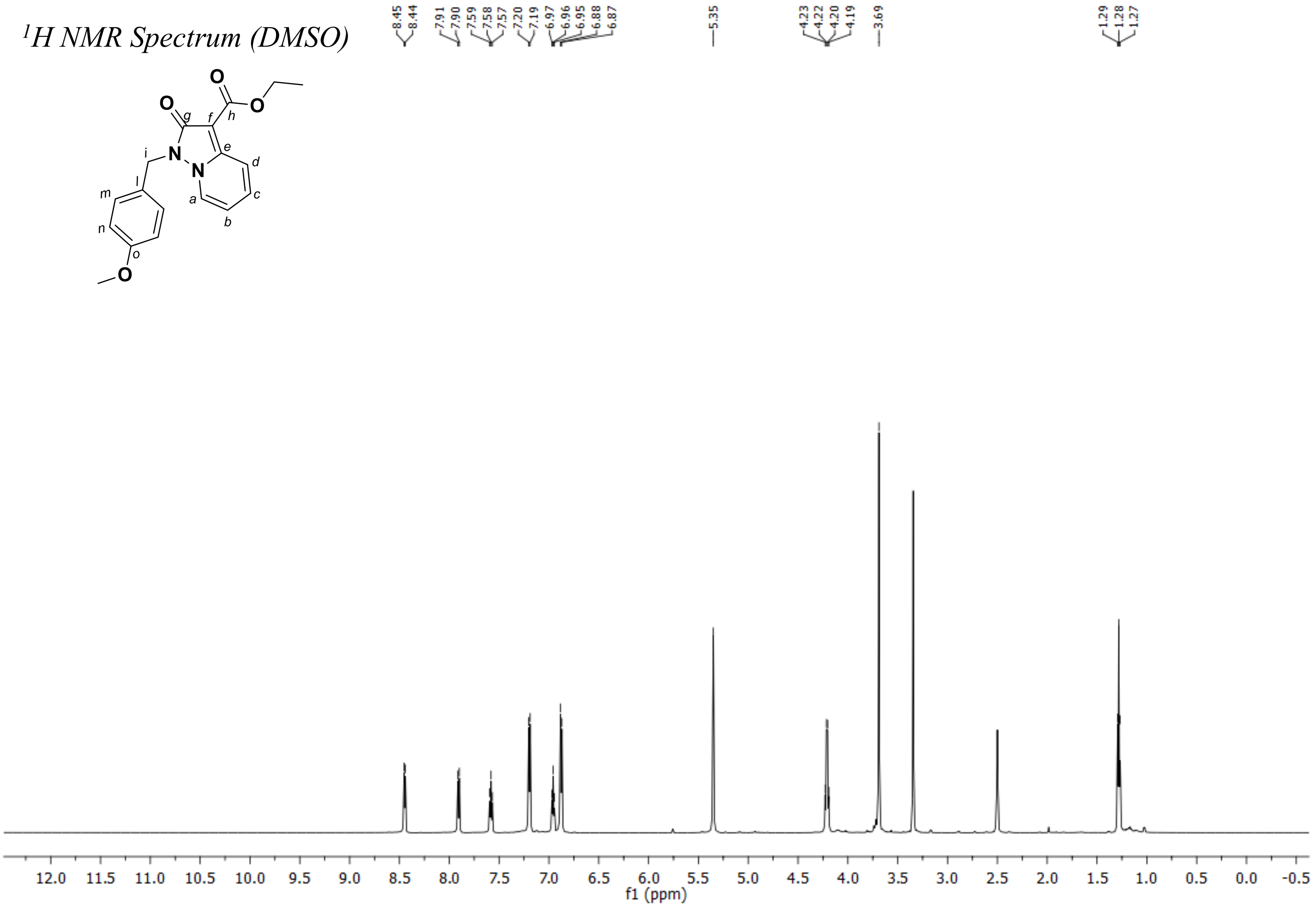

^{13}C NMR Spectrum (DMSO)

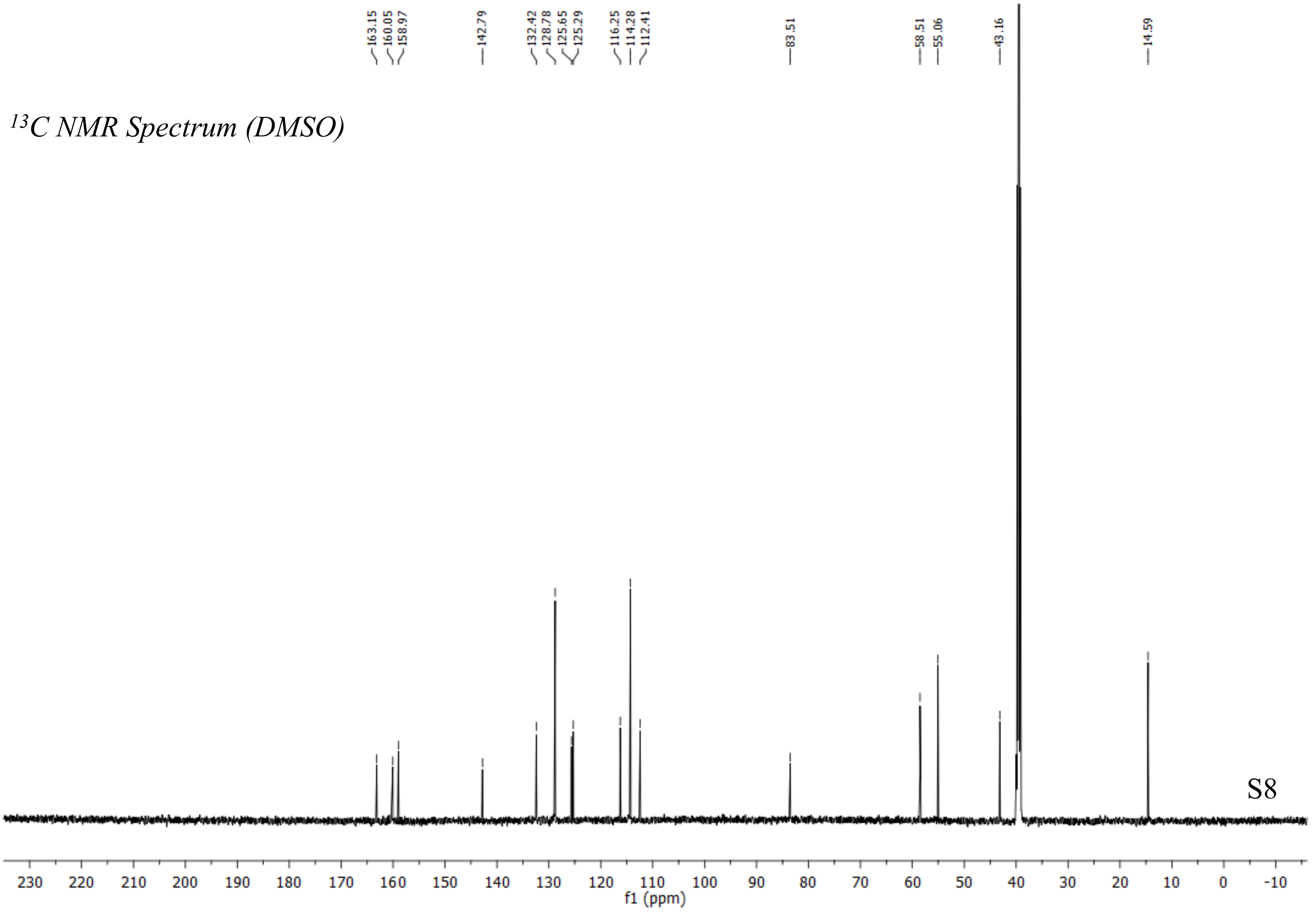

2-((4-Methoxybenzyl)oxy)pyrazolo[1,5-a]pyridine-3-carboxylic acid (**20**)

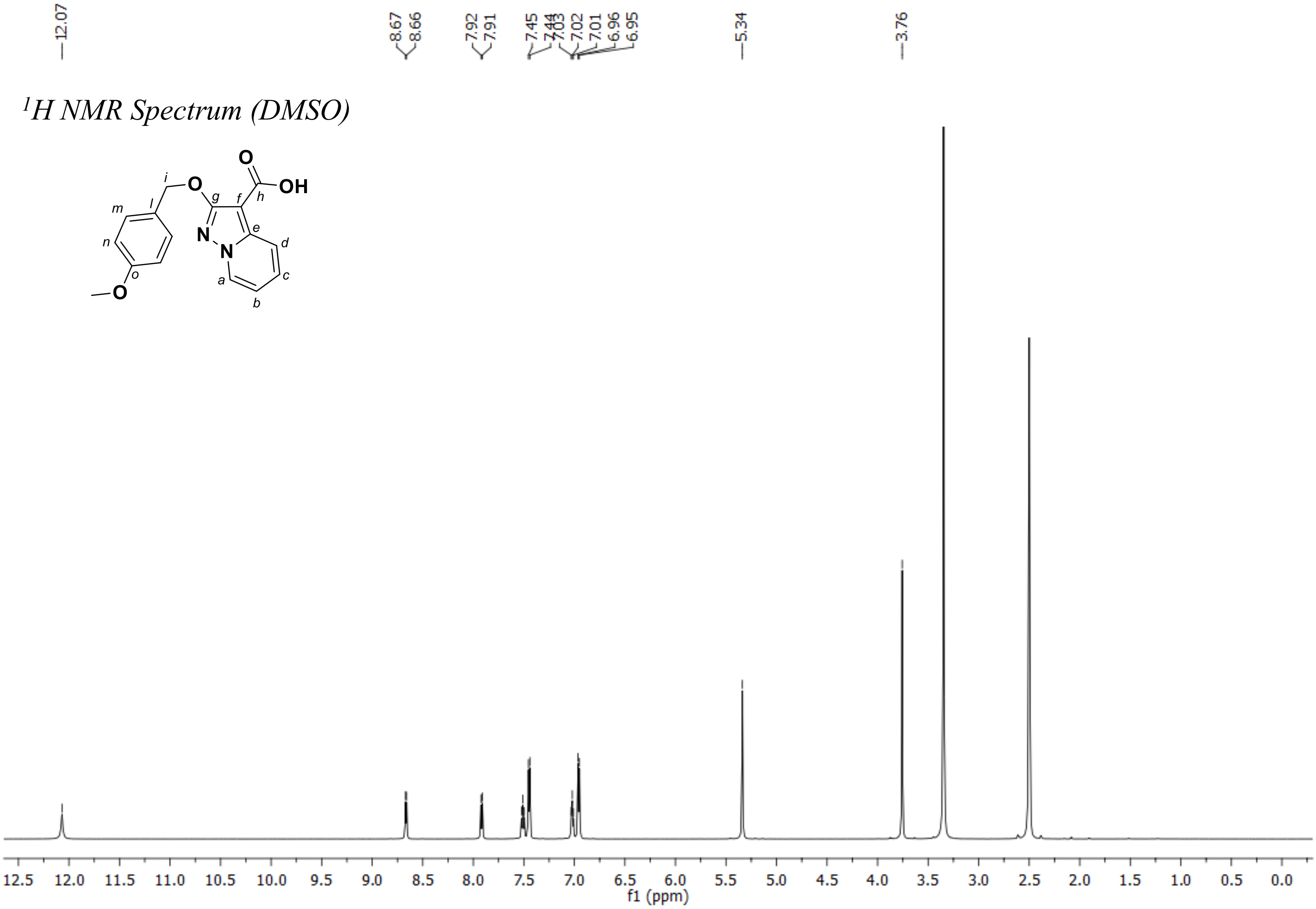

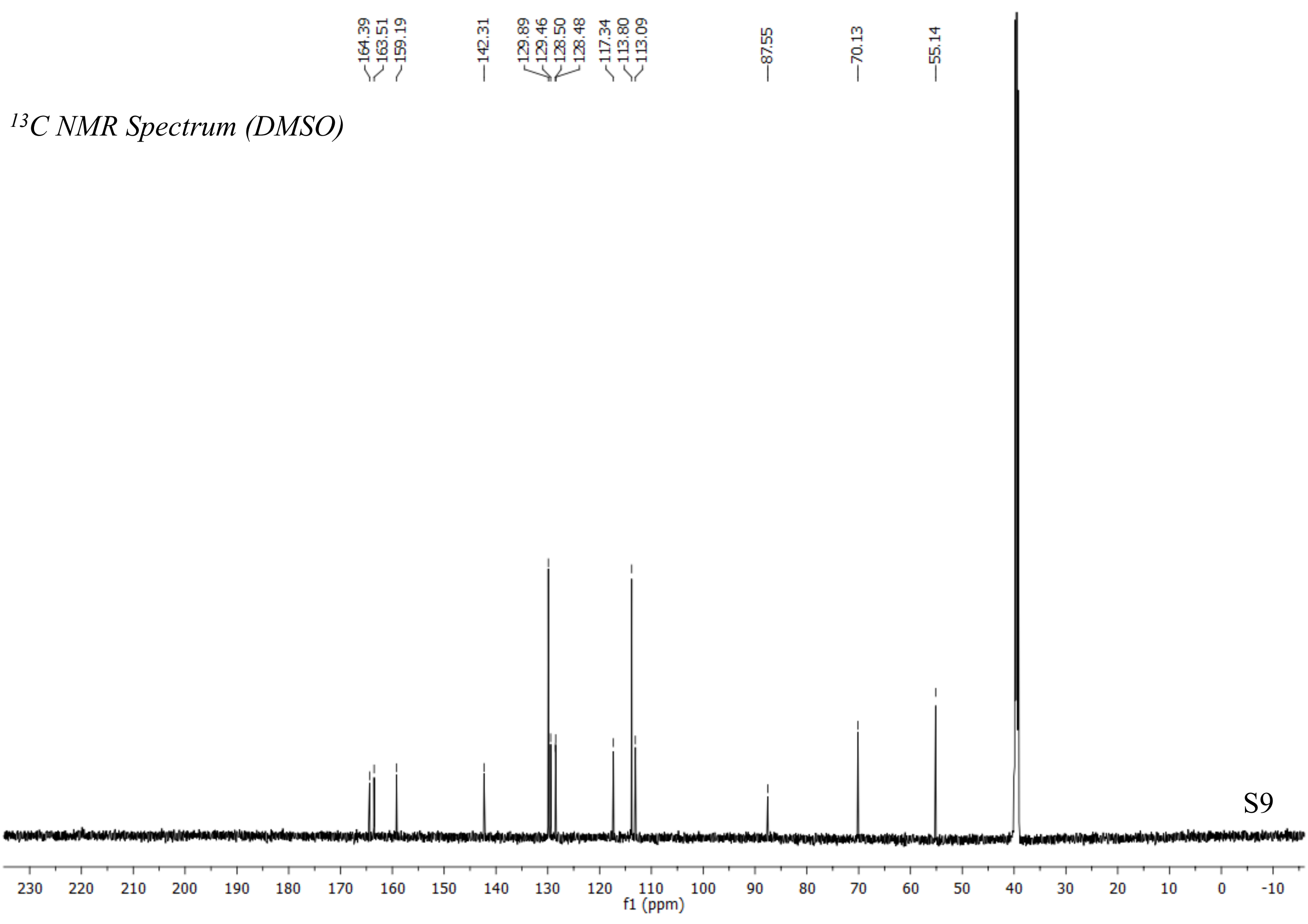

N-(4-bromo-2,3,5,6-tetrafluorophenyl)-1-(4-methoxybenzyl)-2-oxo-1,2-dihydropyrazolo[1,5-a]pyridine-3-carboxamide (**20**)

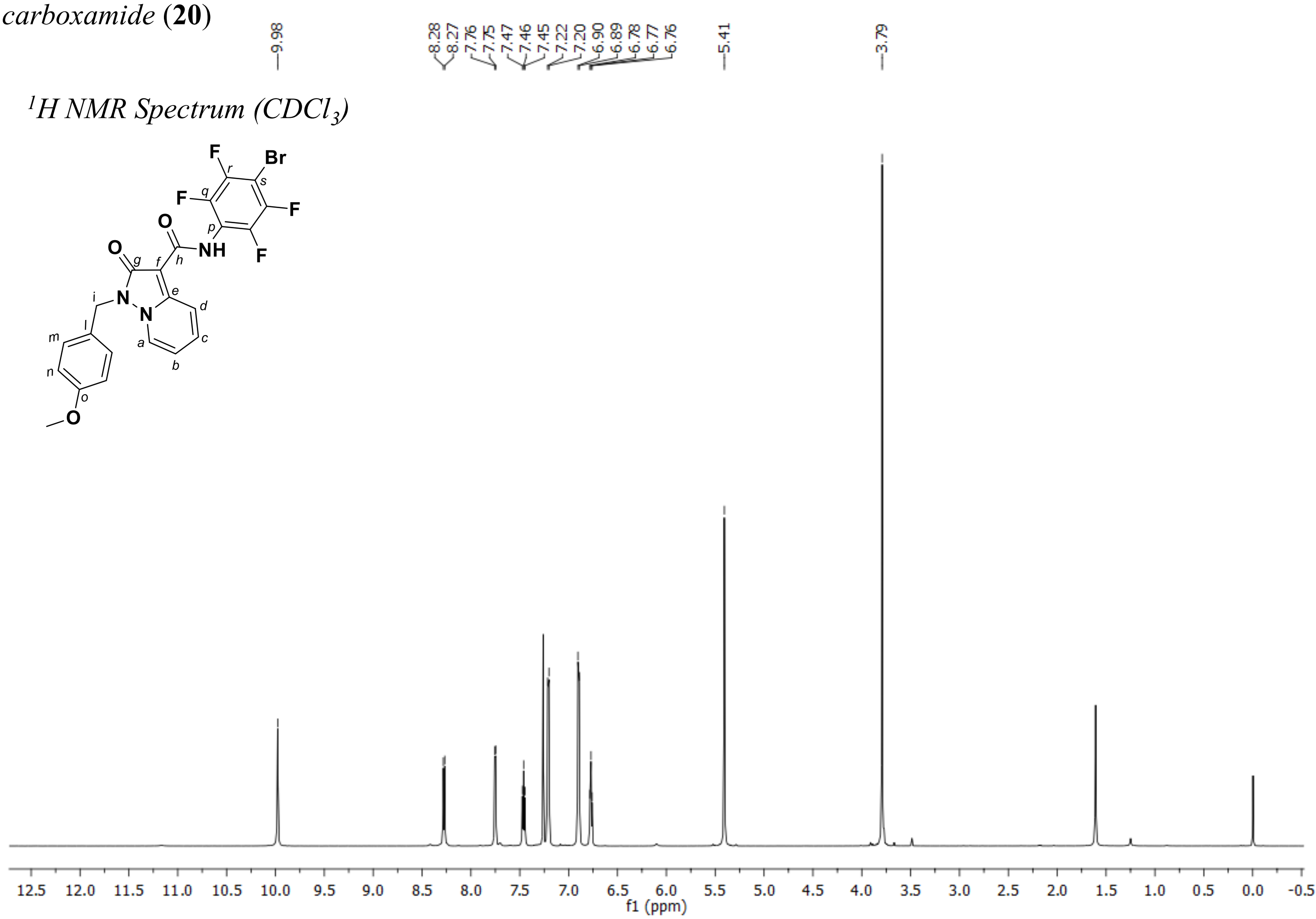

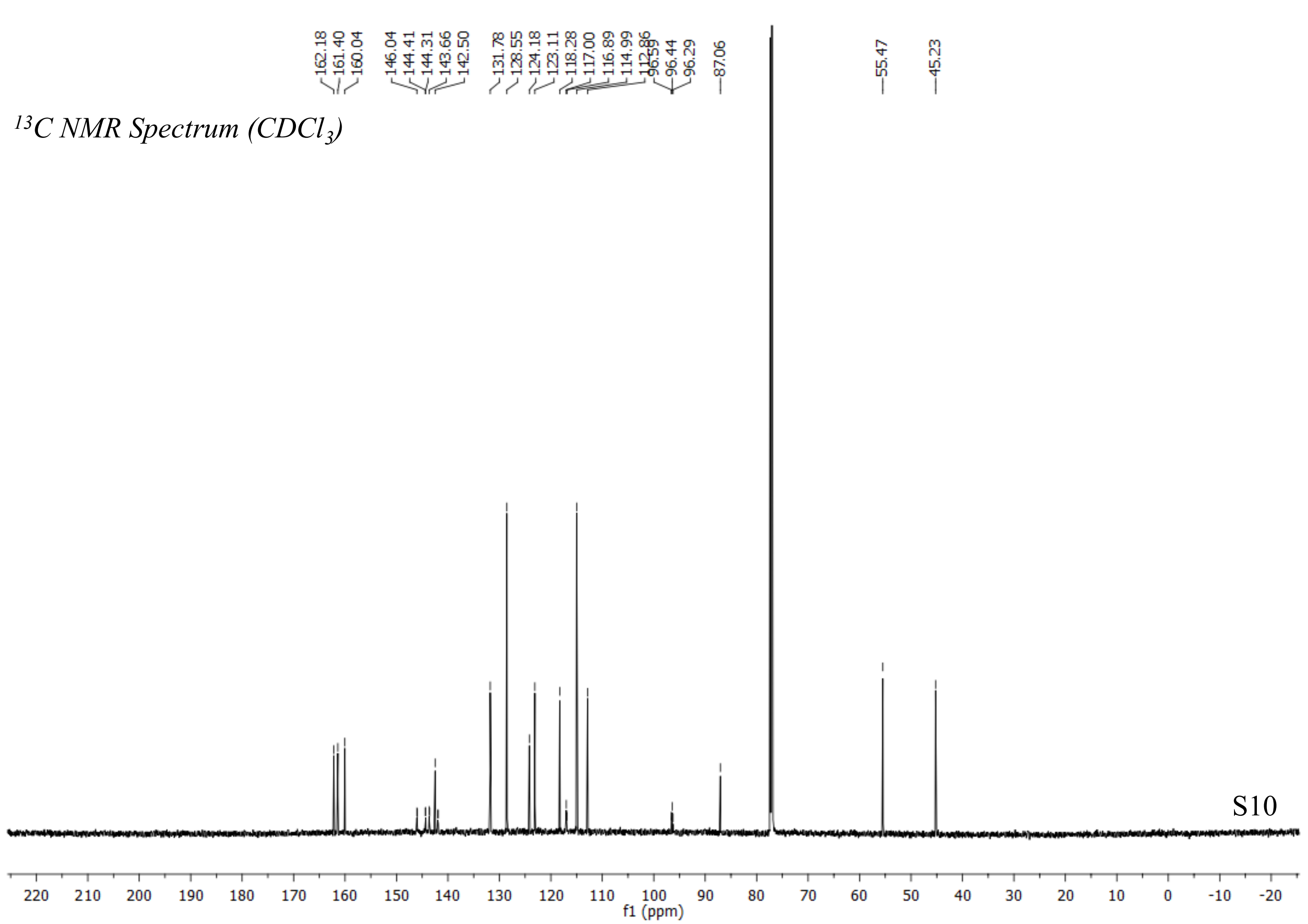

1-(4-Methoxybenzyl)-2-oxo-N-(2,3,5,6-tetrafluoro-4-morpholinophenyl)-1,2-dihydropyrazolo[1,5-a]pyridine-3-carboxamide (**22**)

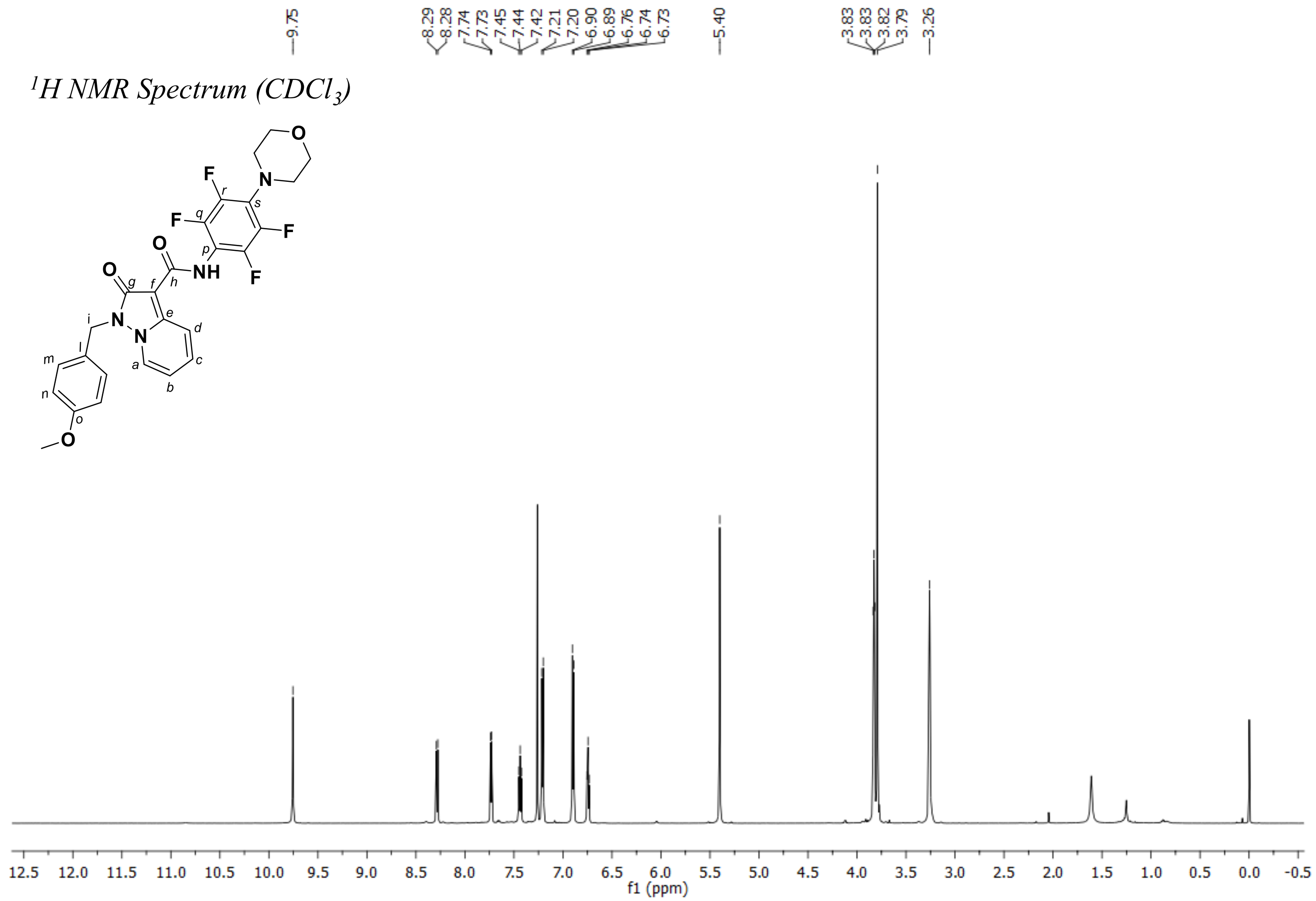

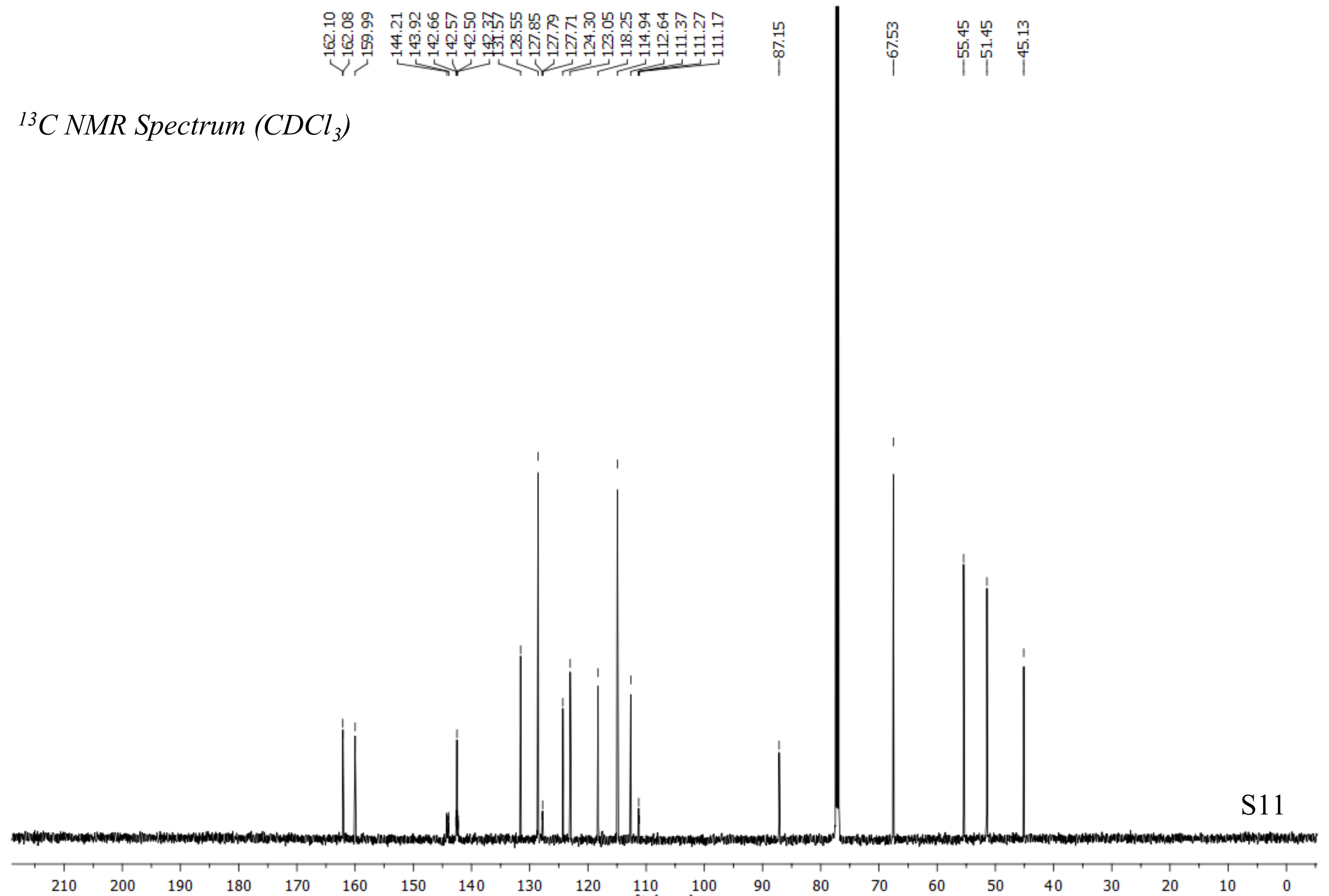

1-(4-Methoxybenzyl)-2-oxo-N-(2,3,5,6-tetrafluoro-4-(thiophen-2-yl)phenyl)-1,2-dihydropyrazolo[1,5-a]pyridine-3-carboxamide (**23**)

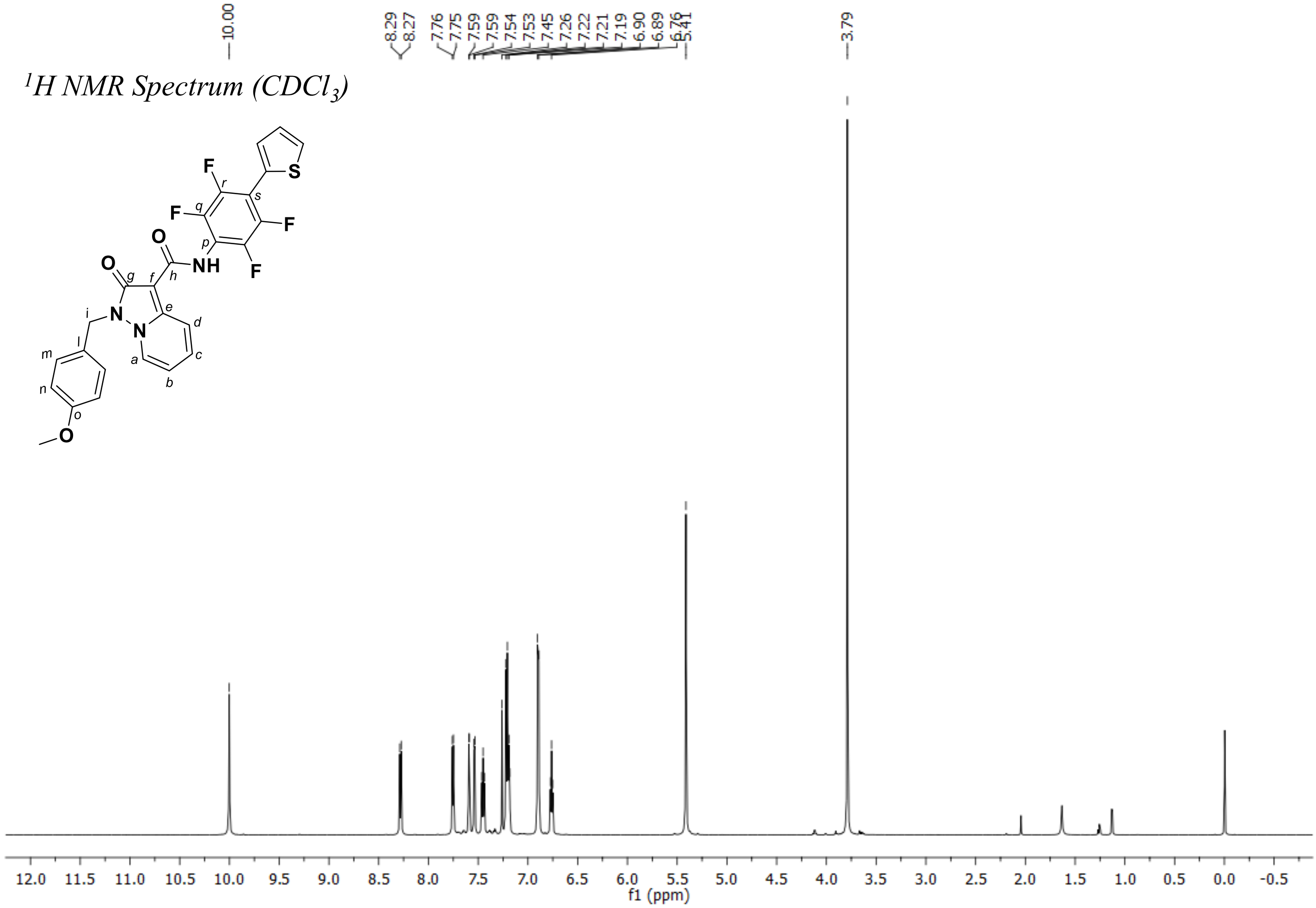

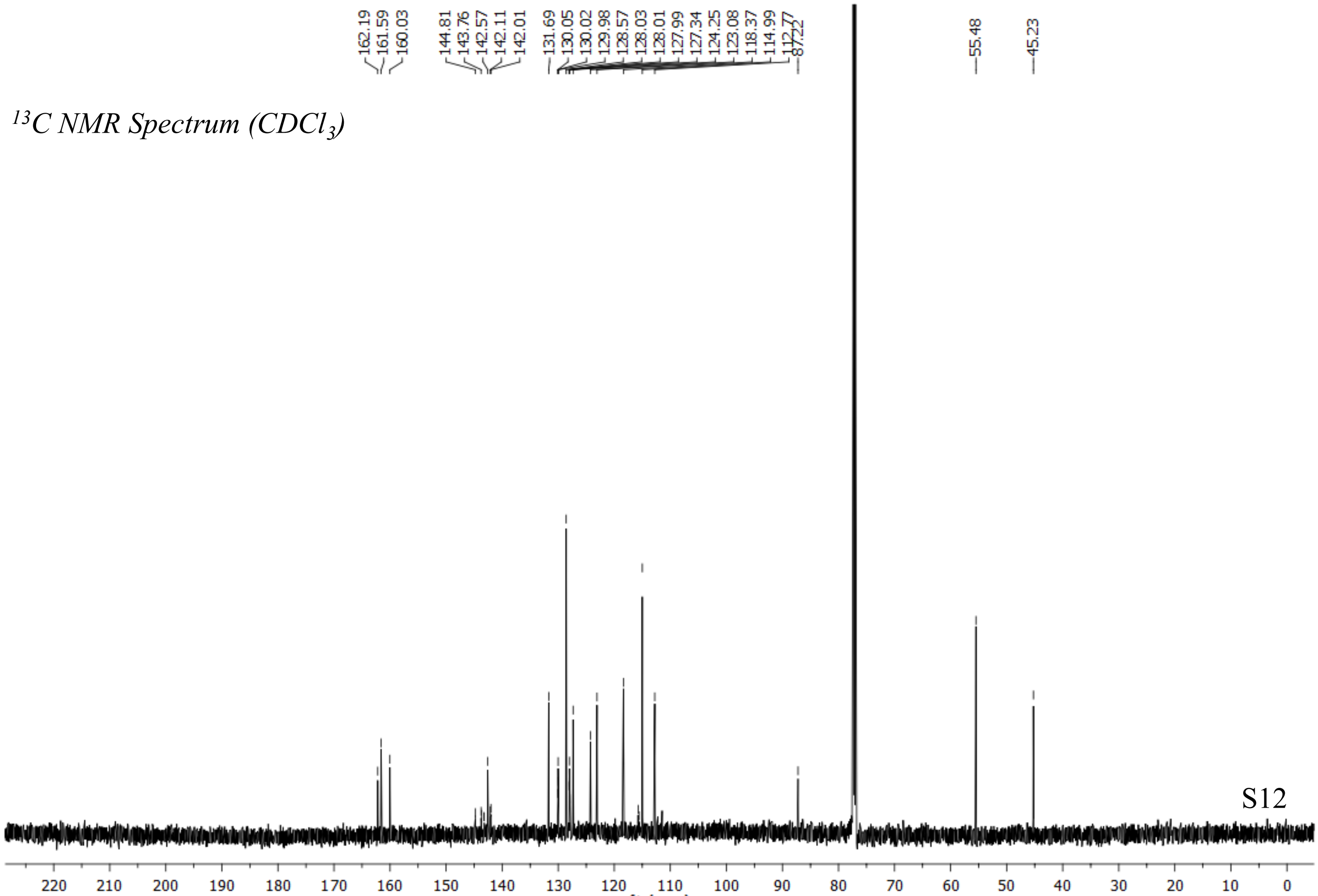

1-(4-Methoxybenzyl)-2-oxo-N-(2,3,5,6-tetrafluoro-4-(pyridin-3-yl)phenyl)-1,2-dihydropyrazolo[1,5-a]pyridine-3-carboxamide (**24**)

^{1}H NMR Spectrum (CDCl$_3$)

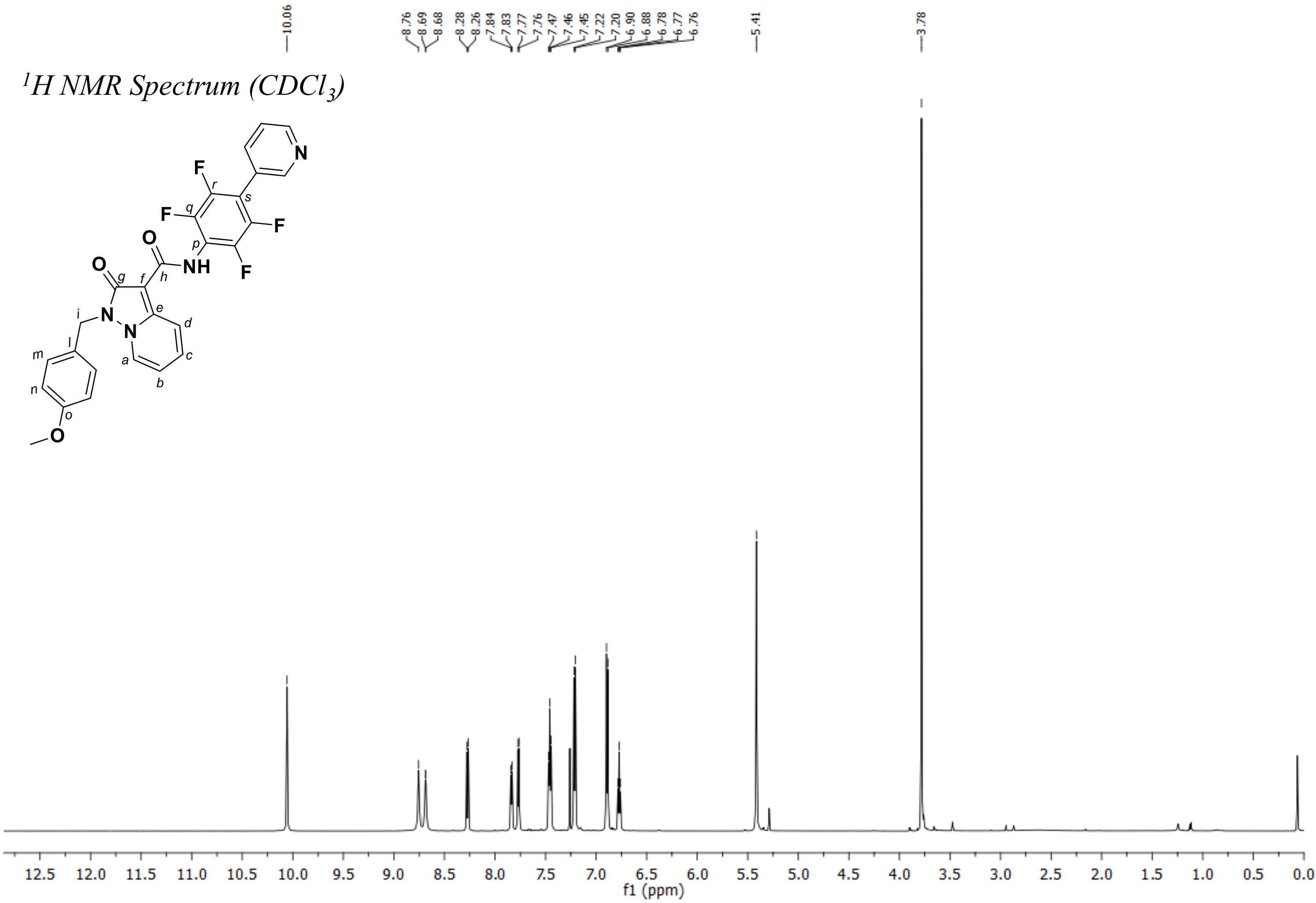

^{13}C NMR Spectrum (CDCl$_3$)

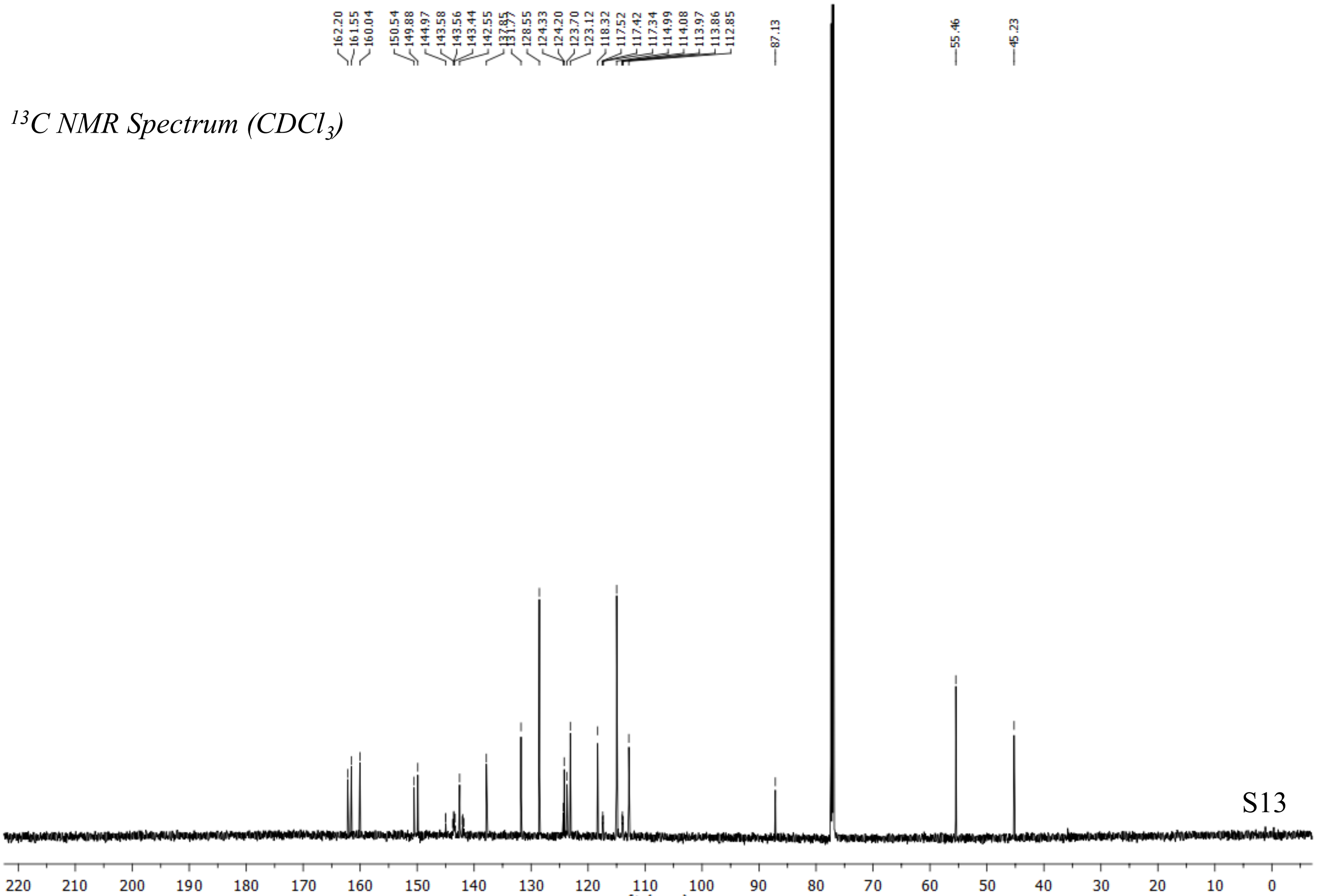

1-(4-Methoxybenzyl)-2-oxo-N-(2,3,5,6-tetrafluoro-4-(2-(trifluoromethyl)pyridin-4-yl)phenyl)-1,2-dihydropyrazolo[1,5-a]pyridine-3-carboxamide (**25**)

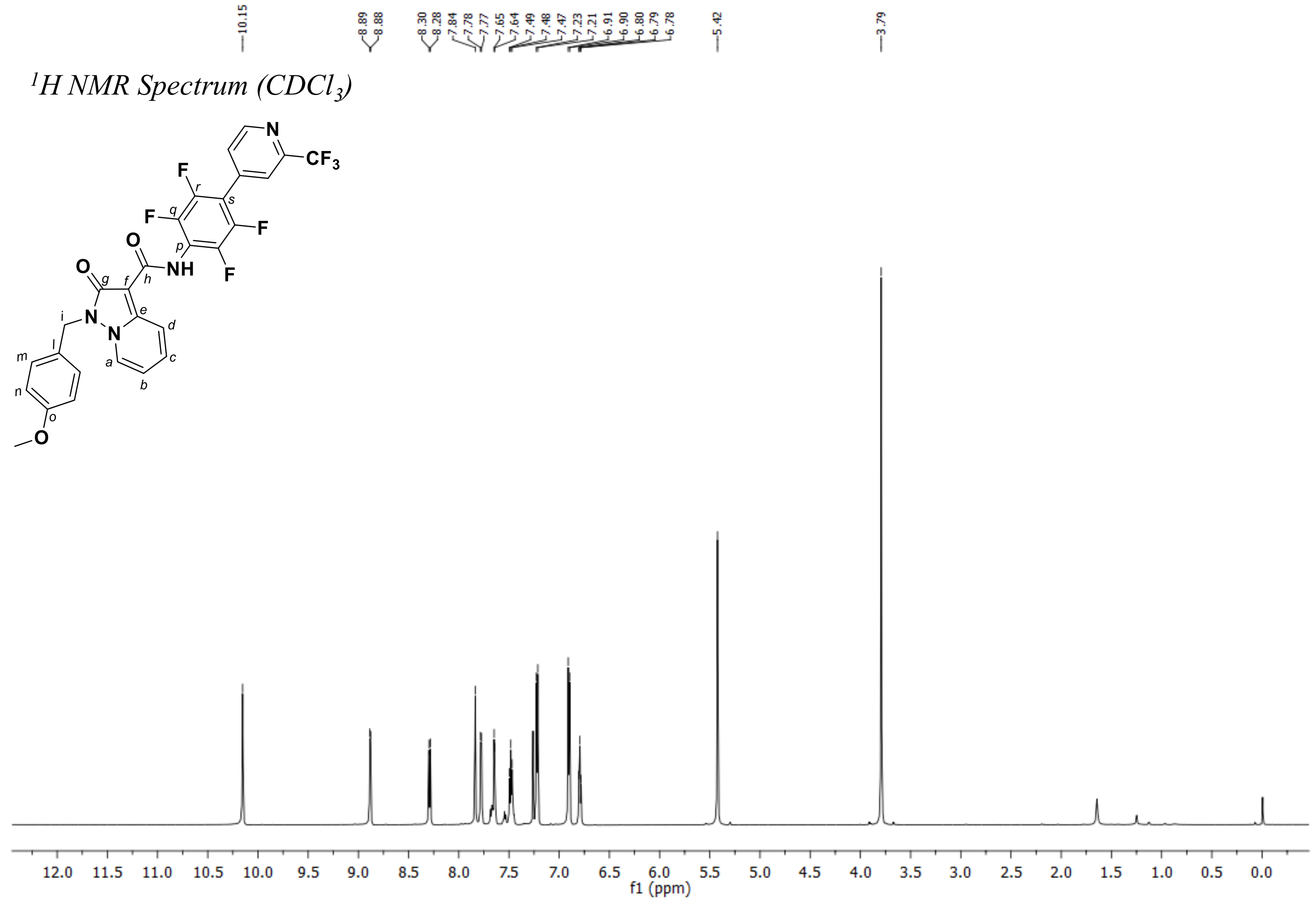

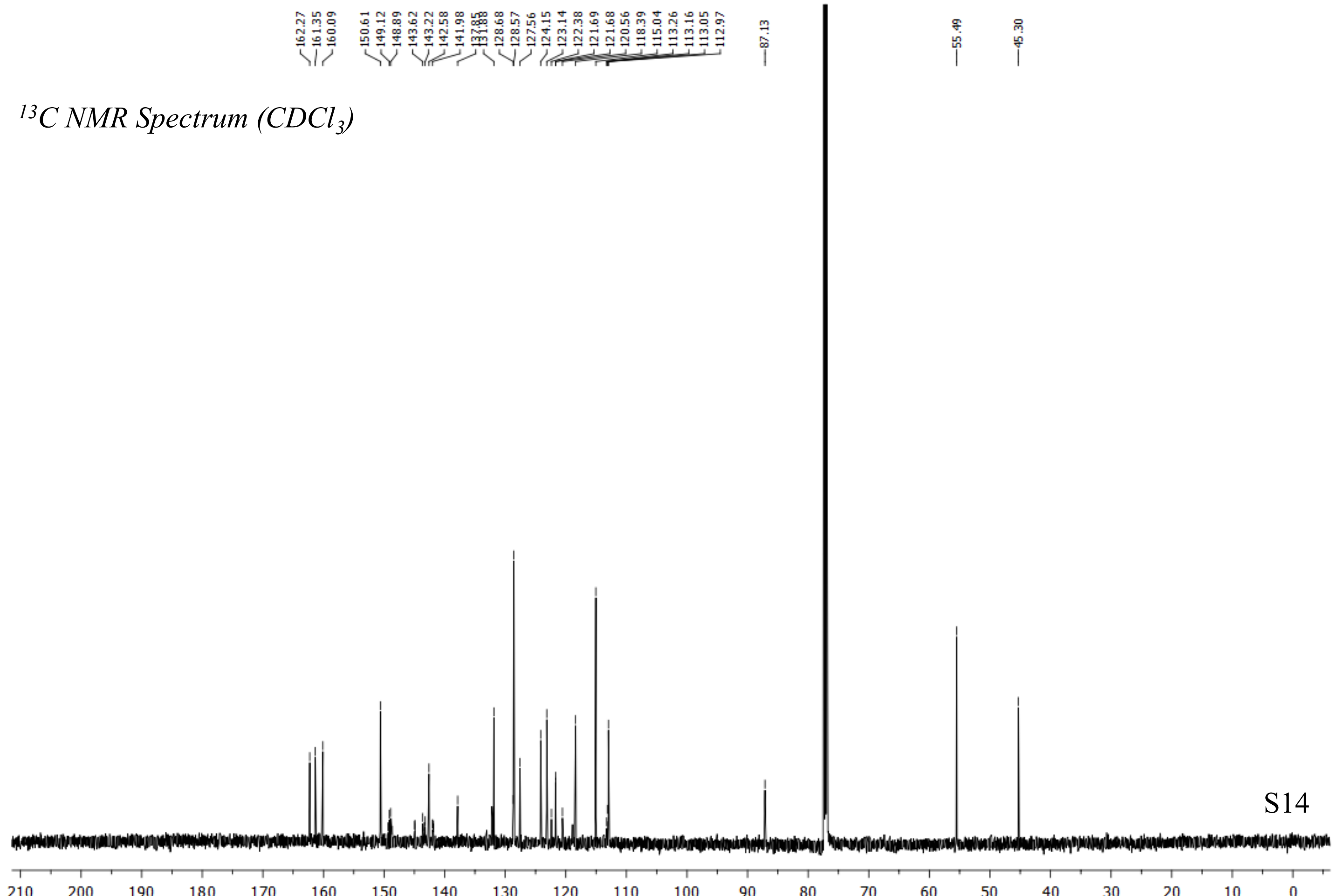

1-(4-Methoxybenzyl)-2-oxo-N-(2,3,5,6-tetrafluoro-3'-hydroxy-[1,1'-biphenyl]-4-yl)-1,2-dihydropyrazolo[1,5-a]pyridine-3-carboxamide (**26**)

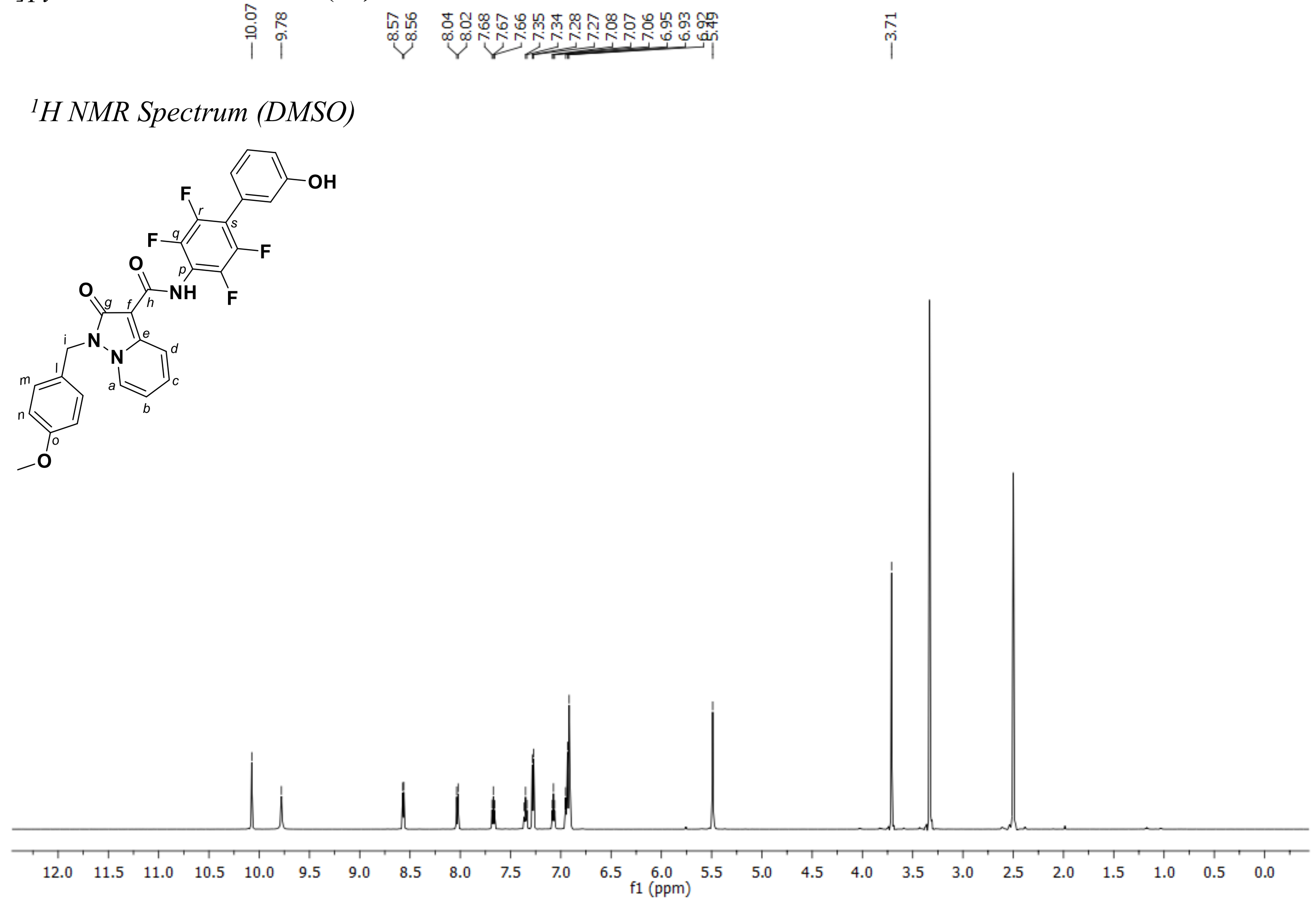

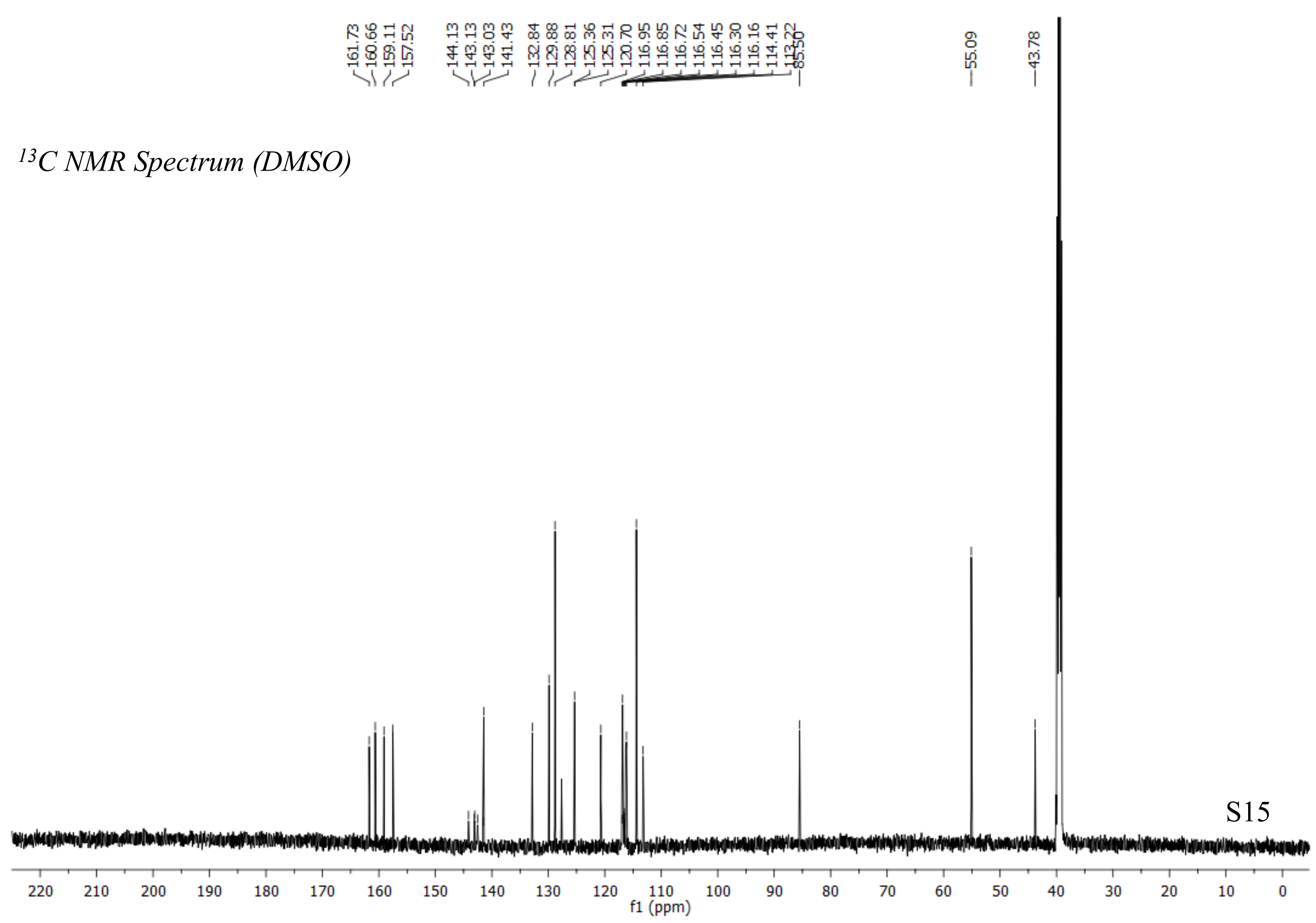

1-(4-Methoxybenzyl)-2-oxo-N-(2,3,5,6-tetrafluoro-3'-(trifluoromethoxy)-[1,1'-biphenyl]-4-yl)-1,2-dihydropyrazolo[1,5-a]pyridine-3-carboxamide (**27**)

^{1}H NMR Spectrum ($CDCl_3$)

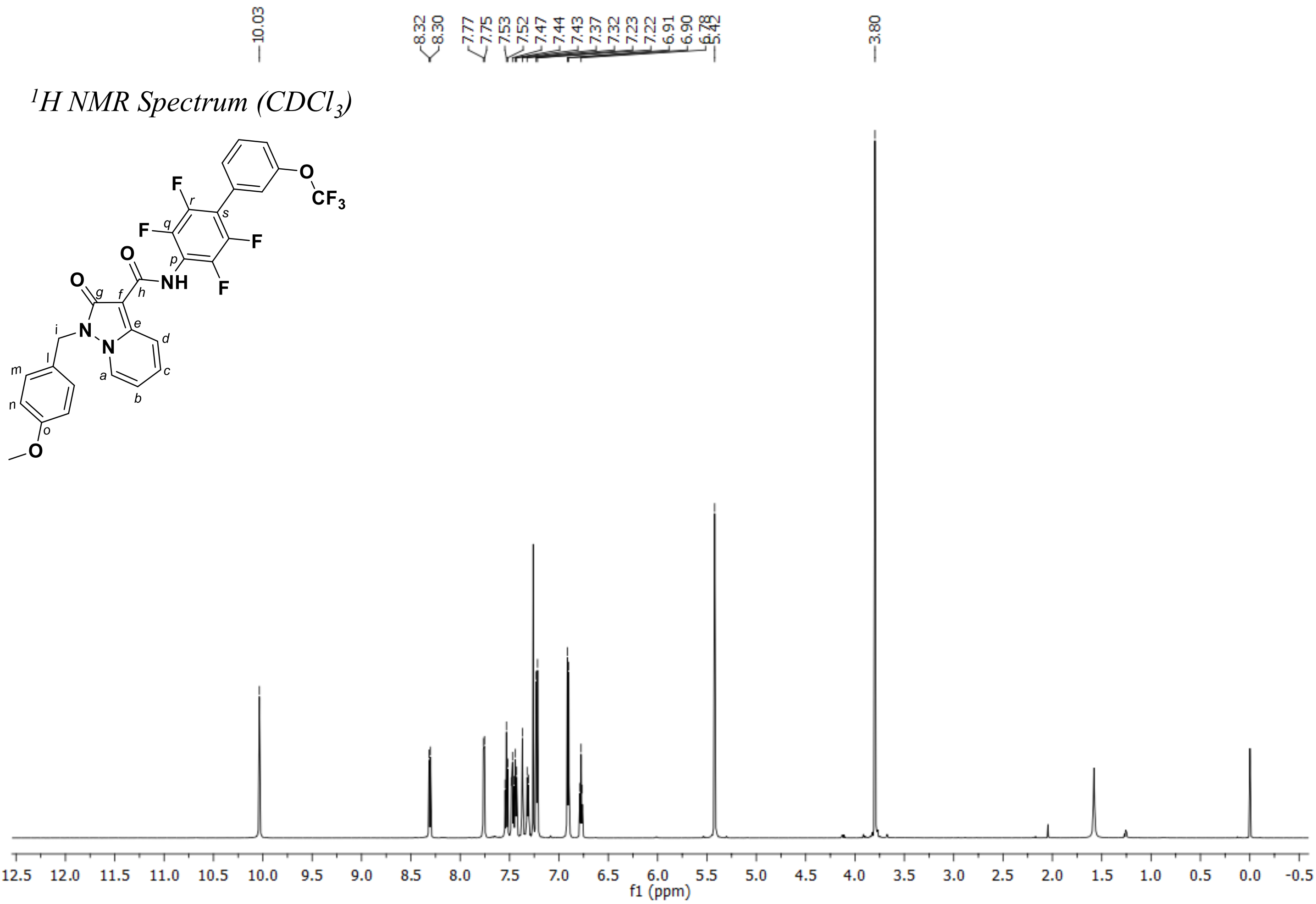

^{13}C NMR Spectrum ($CDCl_3$)

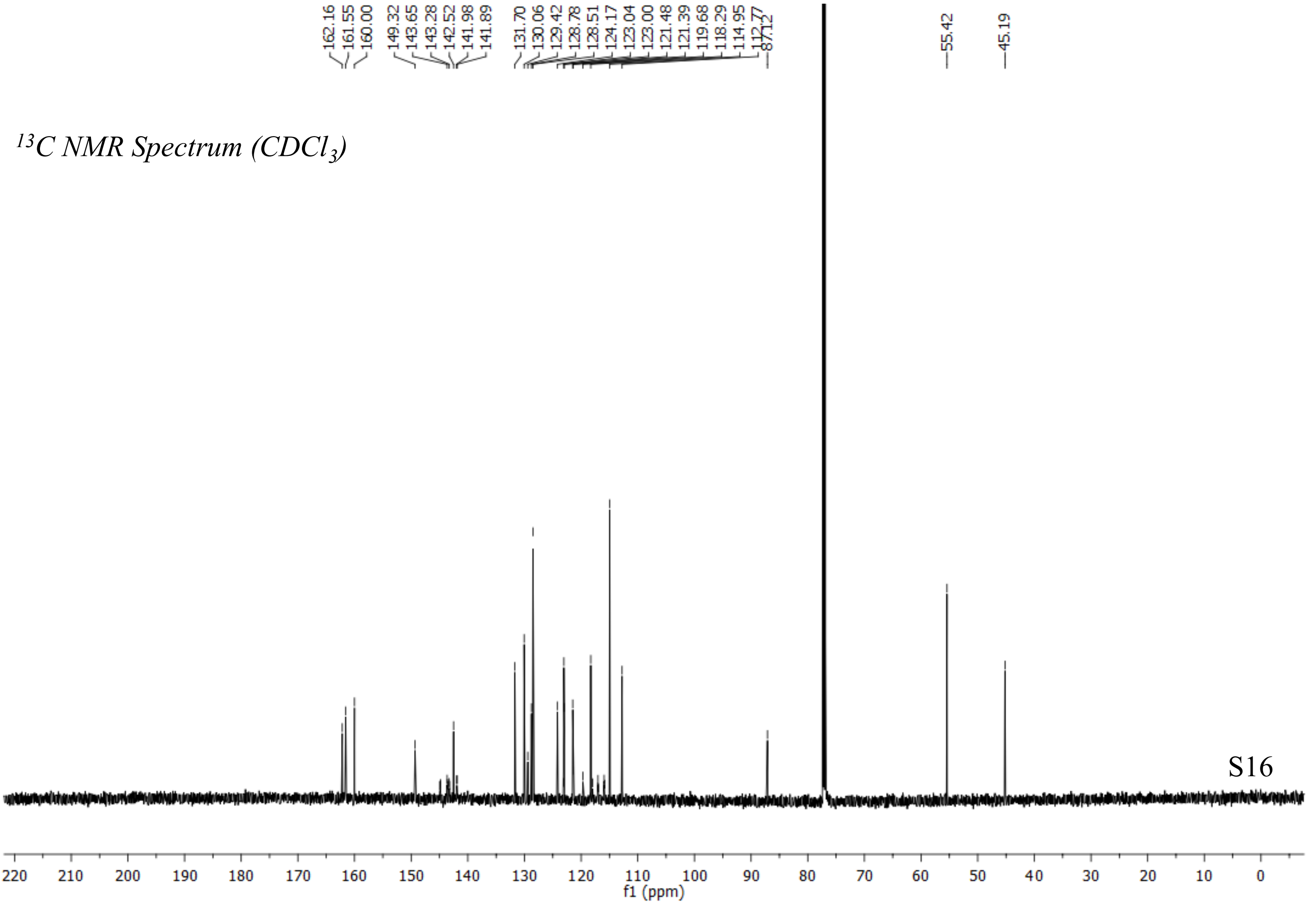

1-(4-Methoxybenzyl)-2-oxo-N-(2,3,5,6-tetrafluoro-3'-methoxy-[1,1'-biphenyl]-4-yl)-1,2-dihydropyrazolo[1,5-a]pyridine-3-carboxamide (**28**)

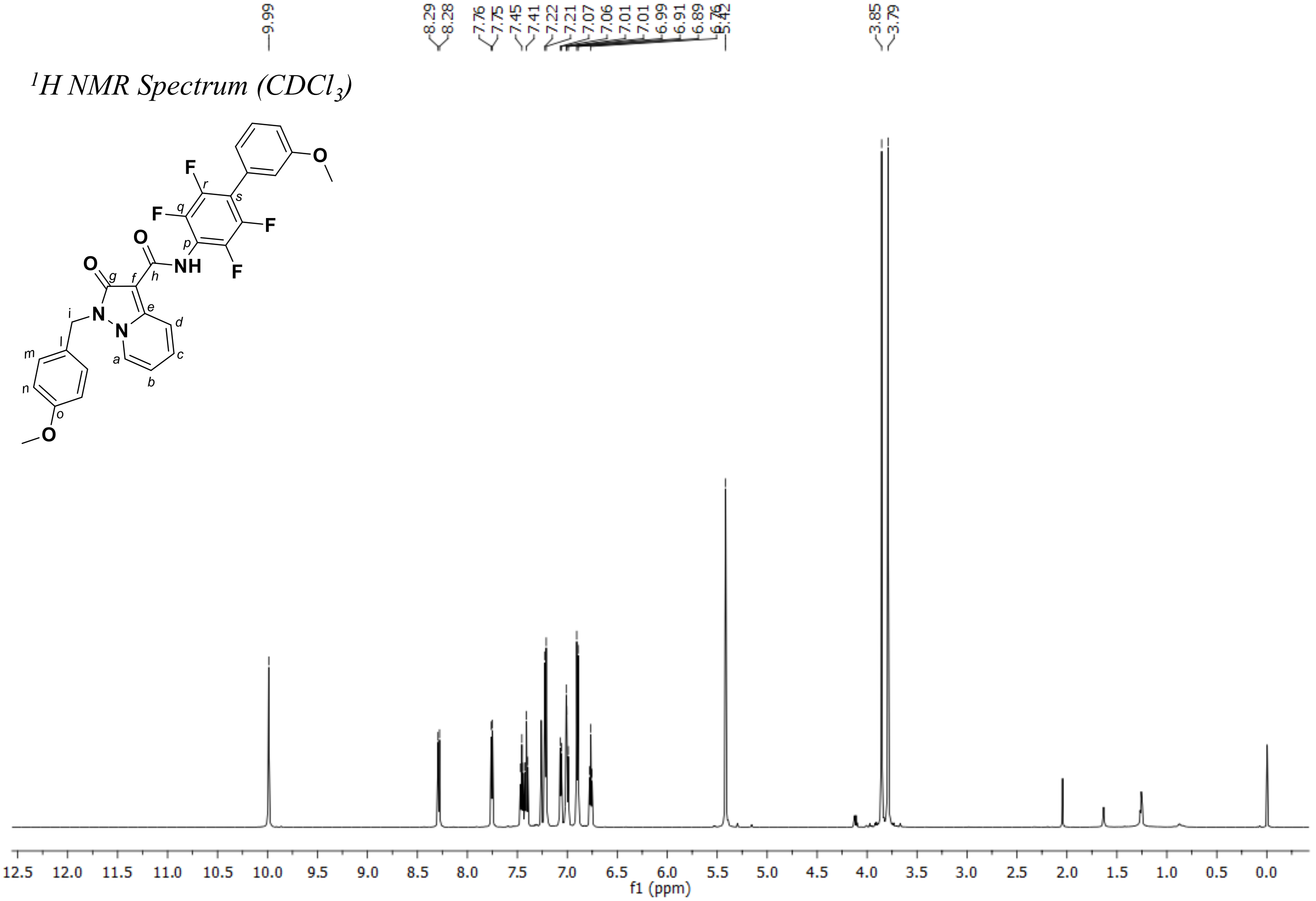

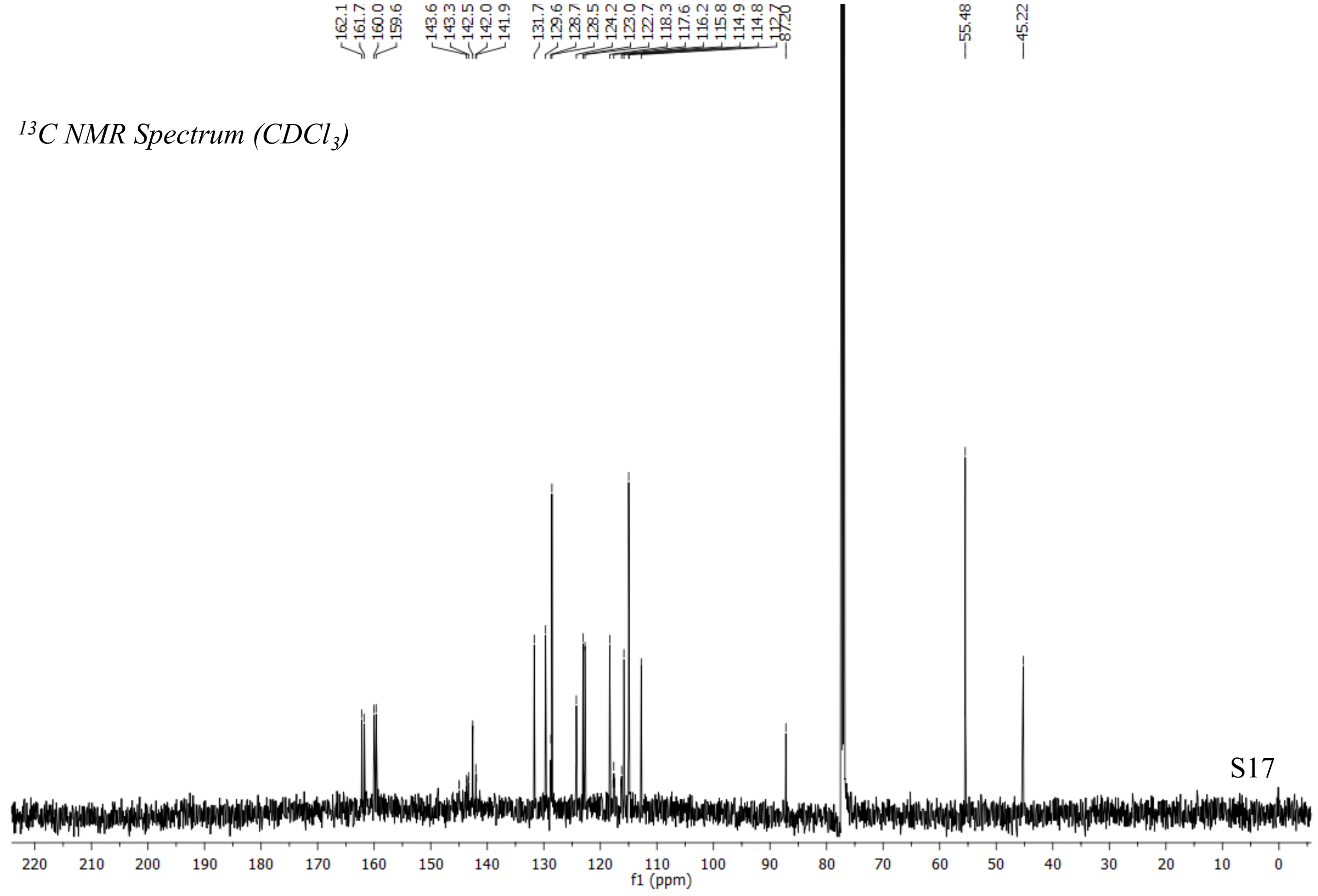

1-(4-Methoxybenzyl)-2-oxo-N-(2,3,5,6-tetrafluoro-3'-propoxy-[1,1'-biphenyl]-4-yl)-1,2-dihydropyrazolo[1,5-a]pyridine-3-carboxamide (**29**)

^{1}H NMR Spectrum (CDCl$_3$)

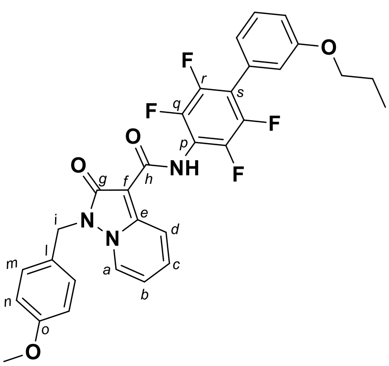

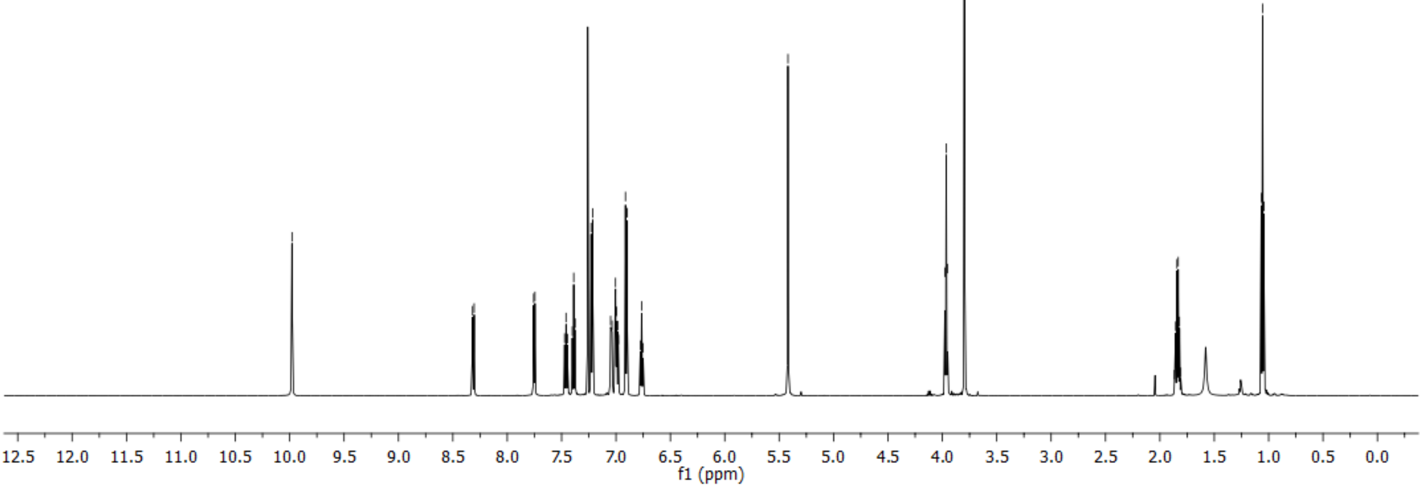

^{13}C NMR Spectrum (CDCl$_3$)

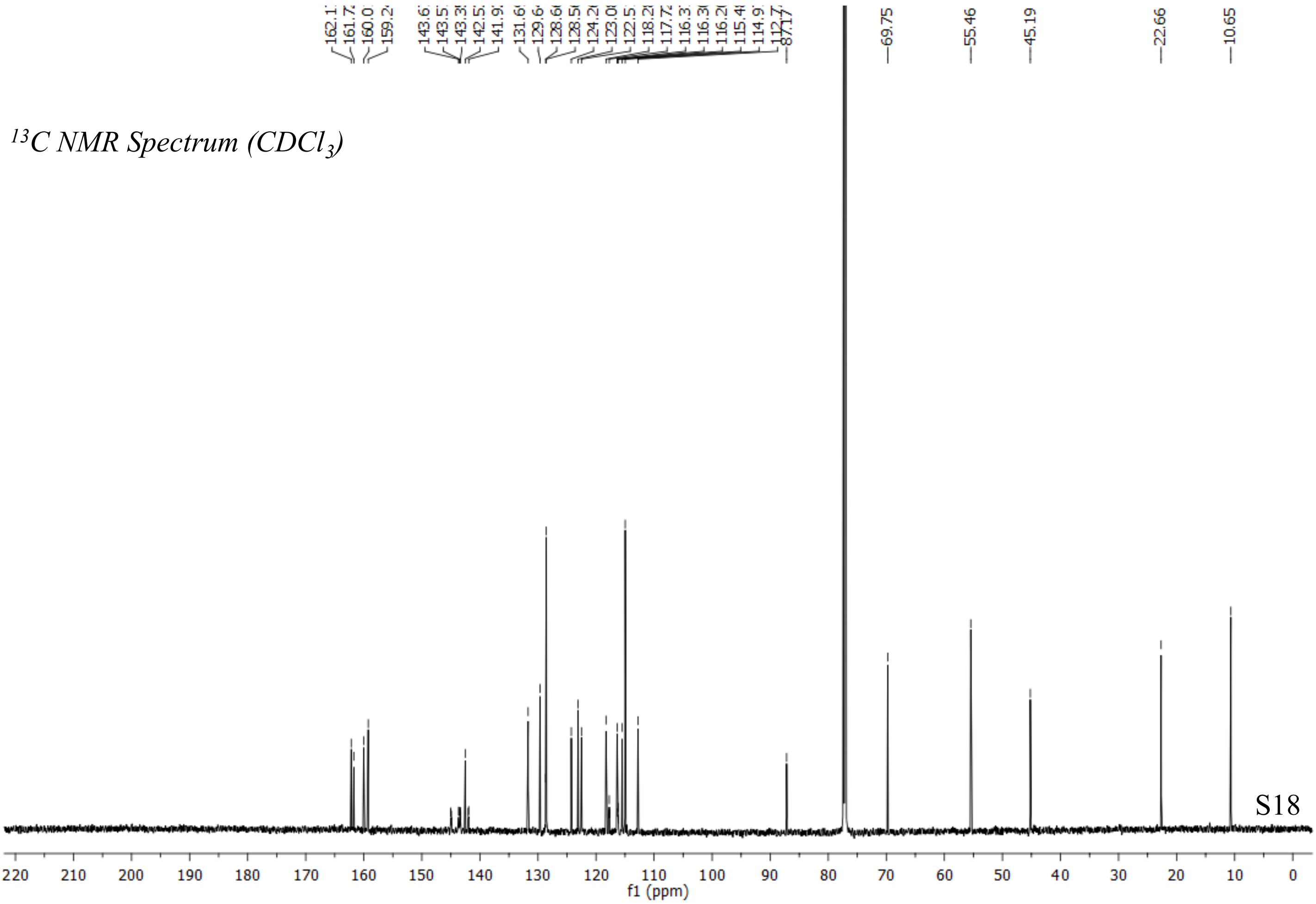

1-Benzyl-2-oxo-N-(2,3,4',5,6-pentafluoro-[1,1'-biphenyl]-4-yl)-1,2-dihydropyrazolo[1,5-a]pyridine-3-carboxamide (**32**)

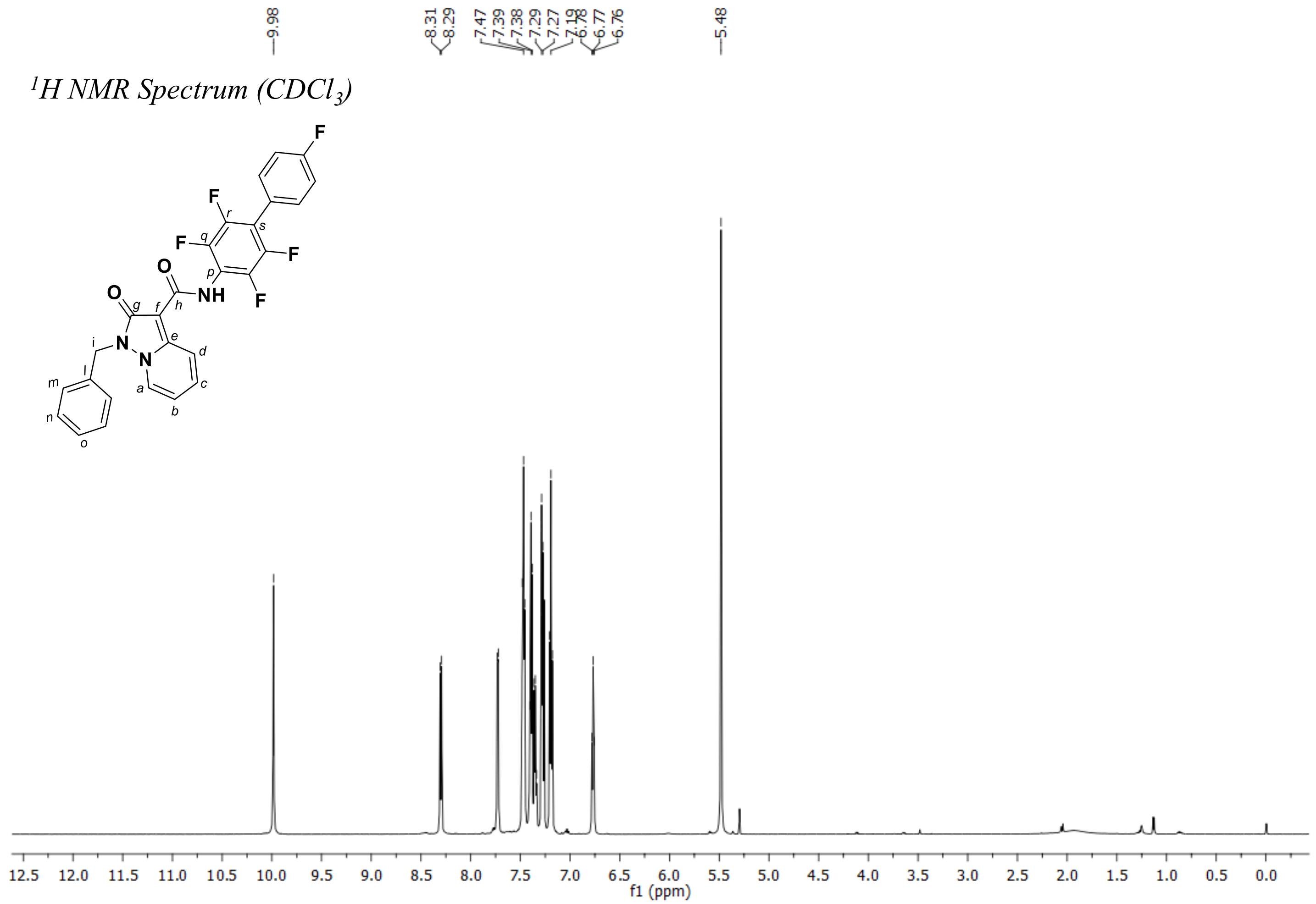

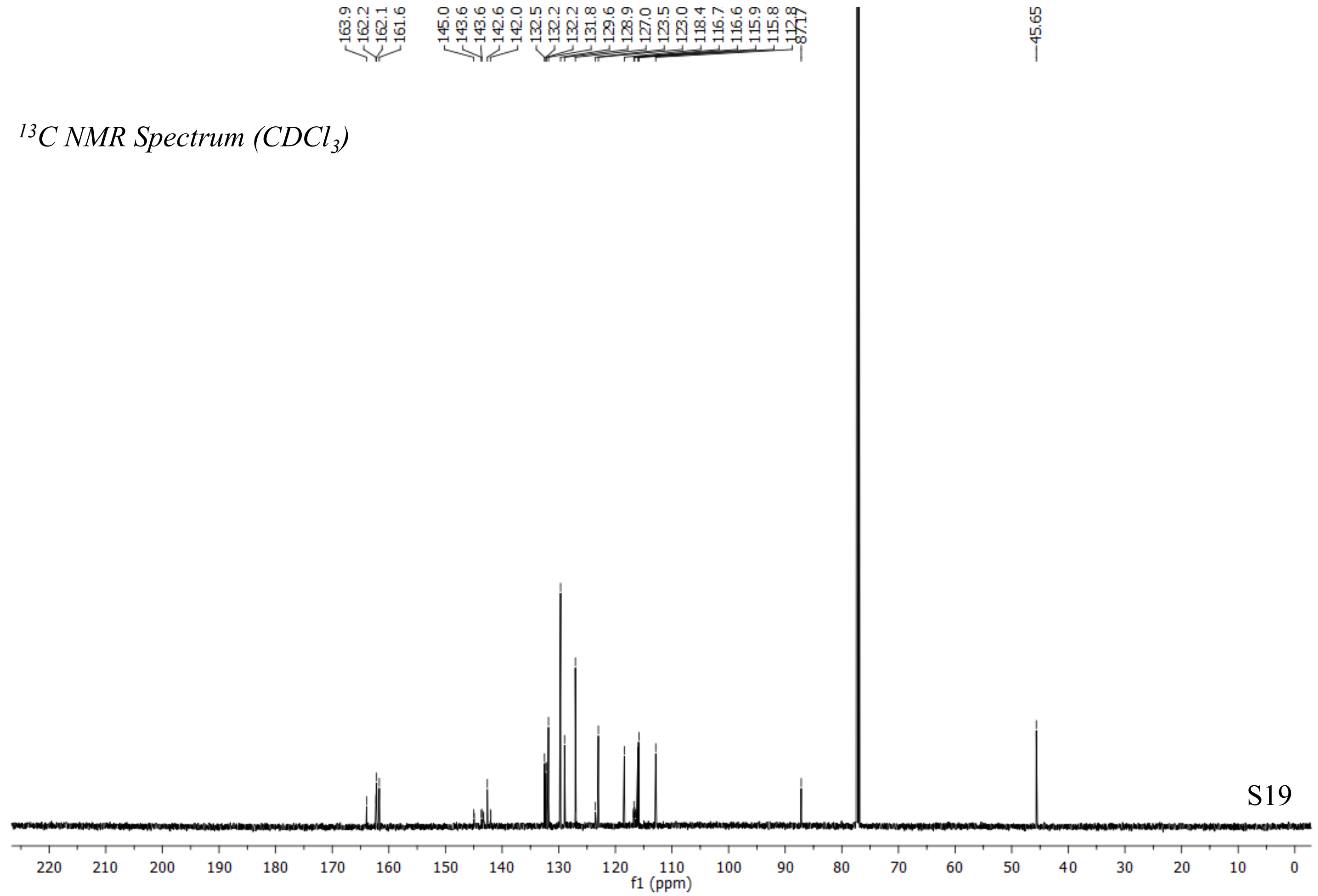

1-Benzyl-2-oxo-N-(2,3,3',5,6-pentafluoro-[1,1'-biphenyl]-4-yl)-1,2-dihydropyrazolo[1,5-a]pyridine-3-carboxamide (**33**)

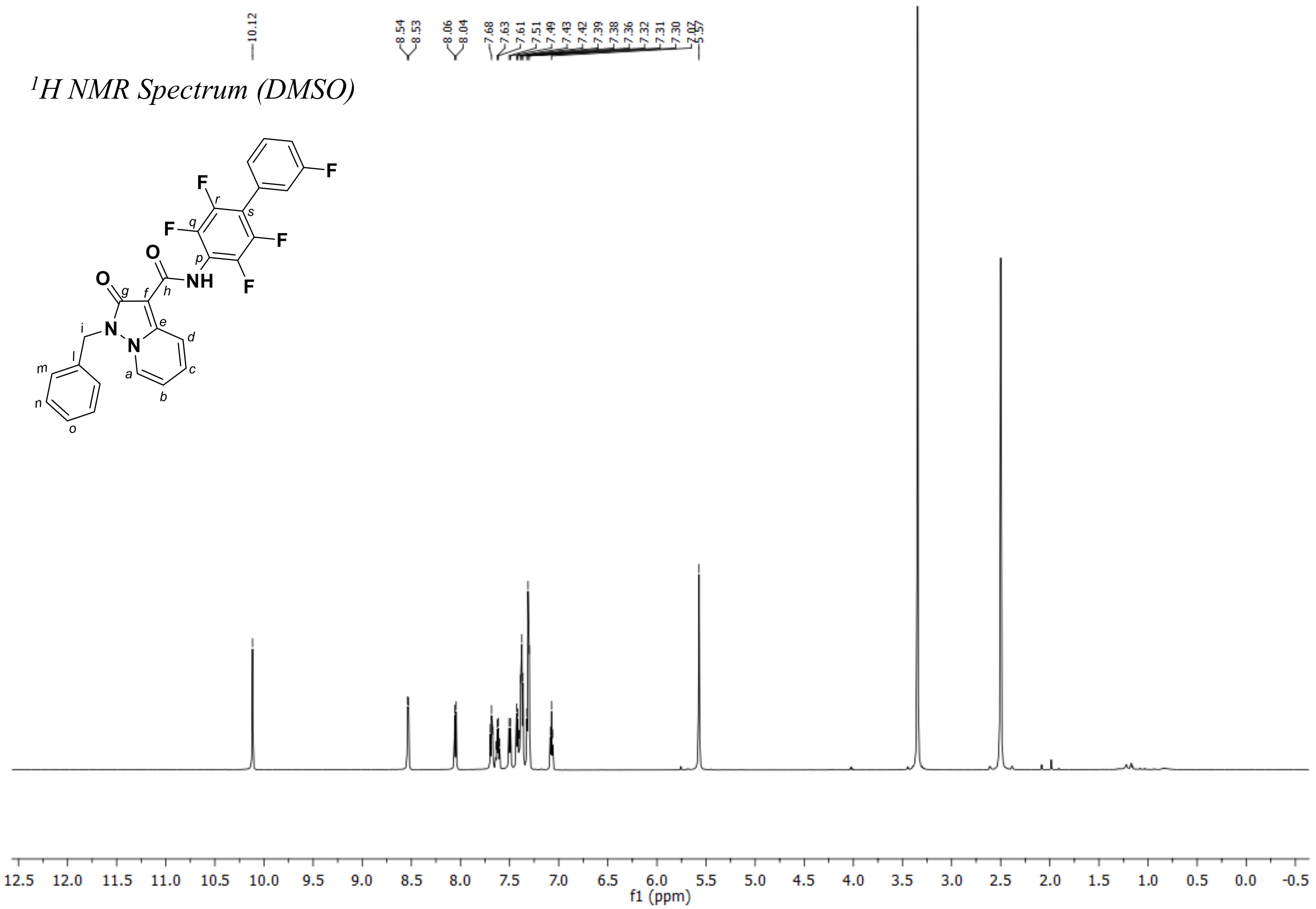

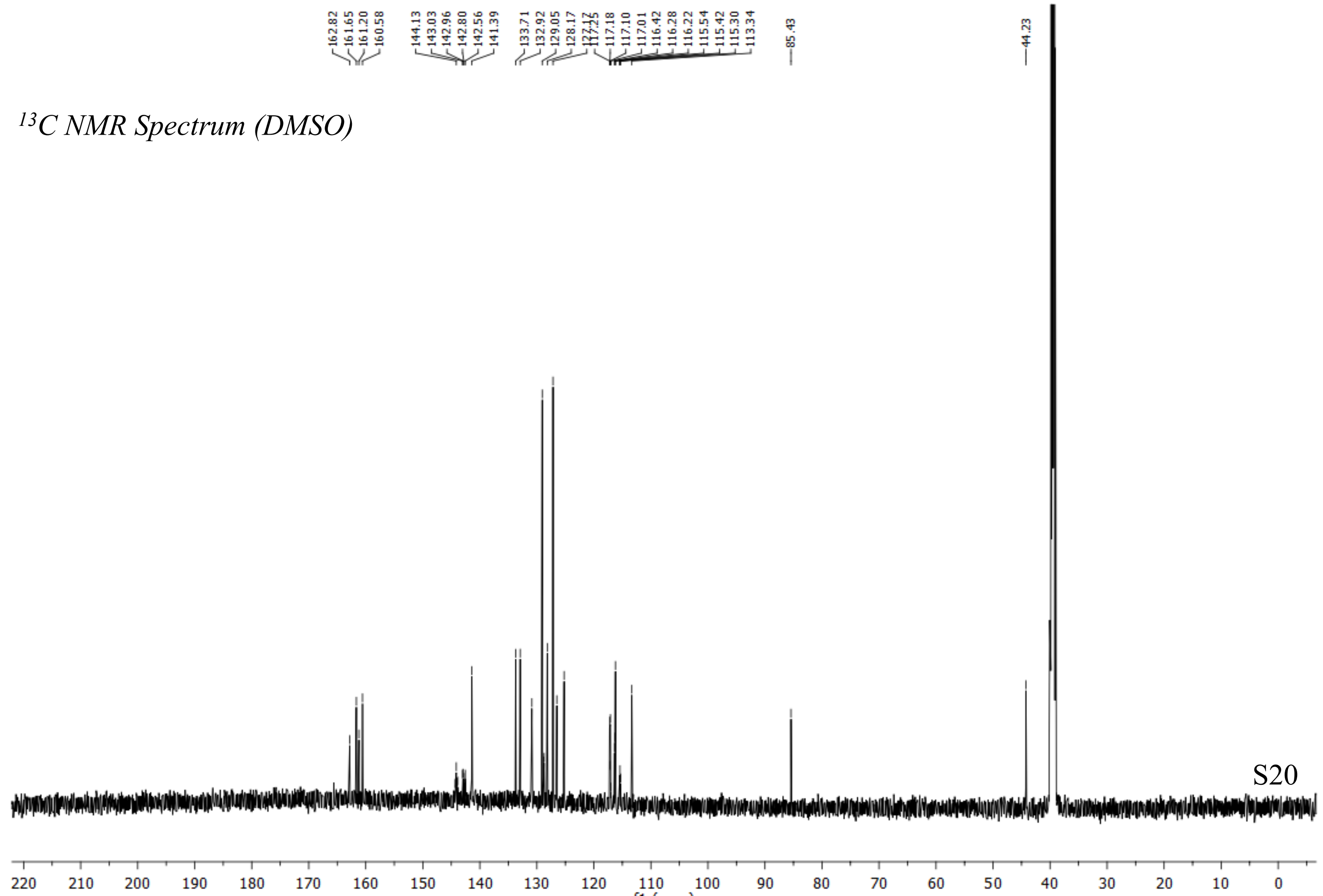

1-Benzyl-2-oxo-N-(2,3,5,6-tetrafluoro-4'-(trifluoromethyl)-[1,1'-biphenyl]-4-yl)-1,2-dihydropyrazolo[1,5-a]pyridine-3-carboxamide (**33**)

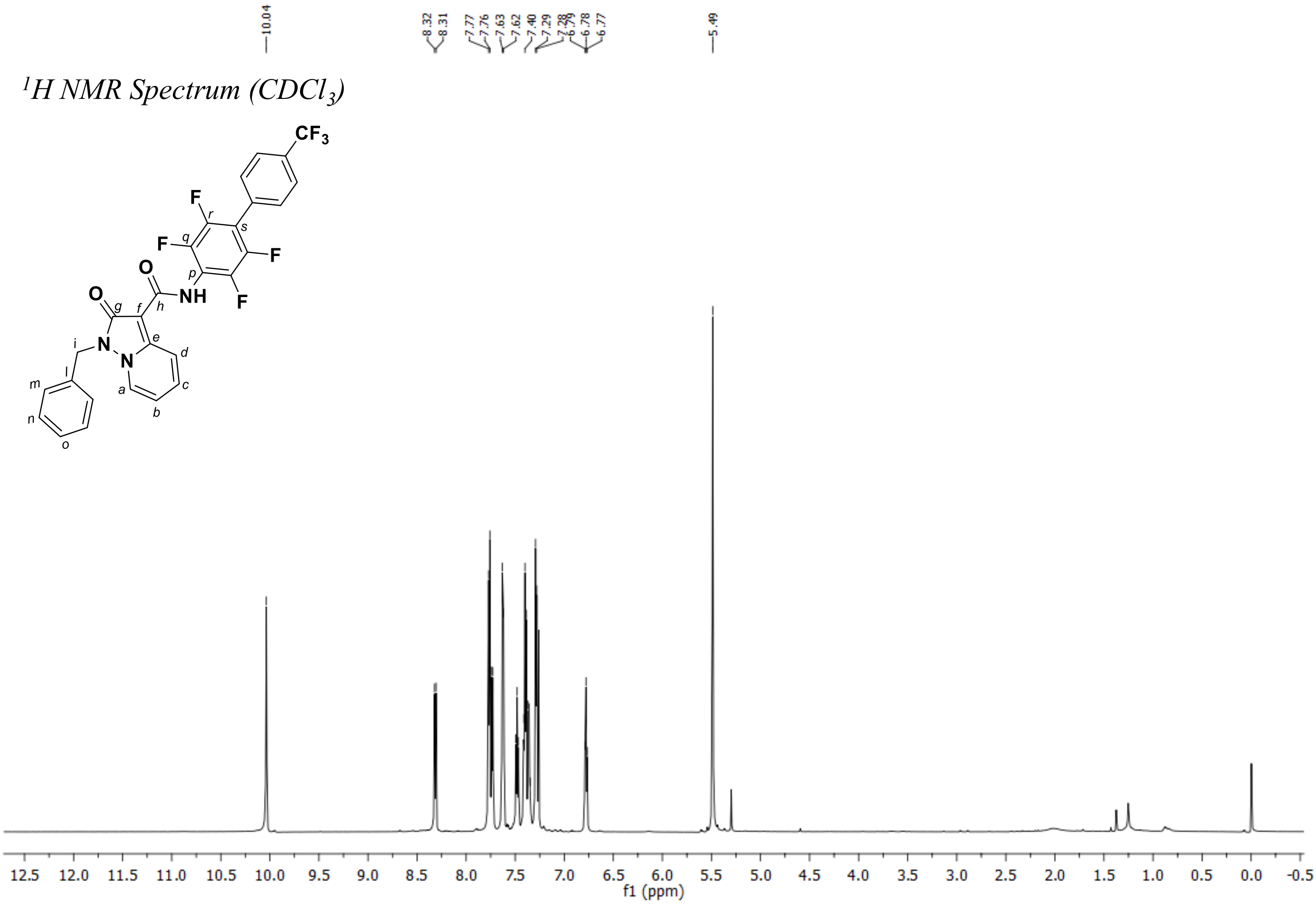

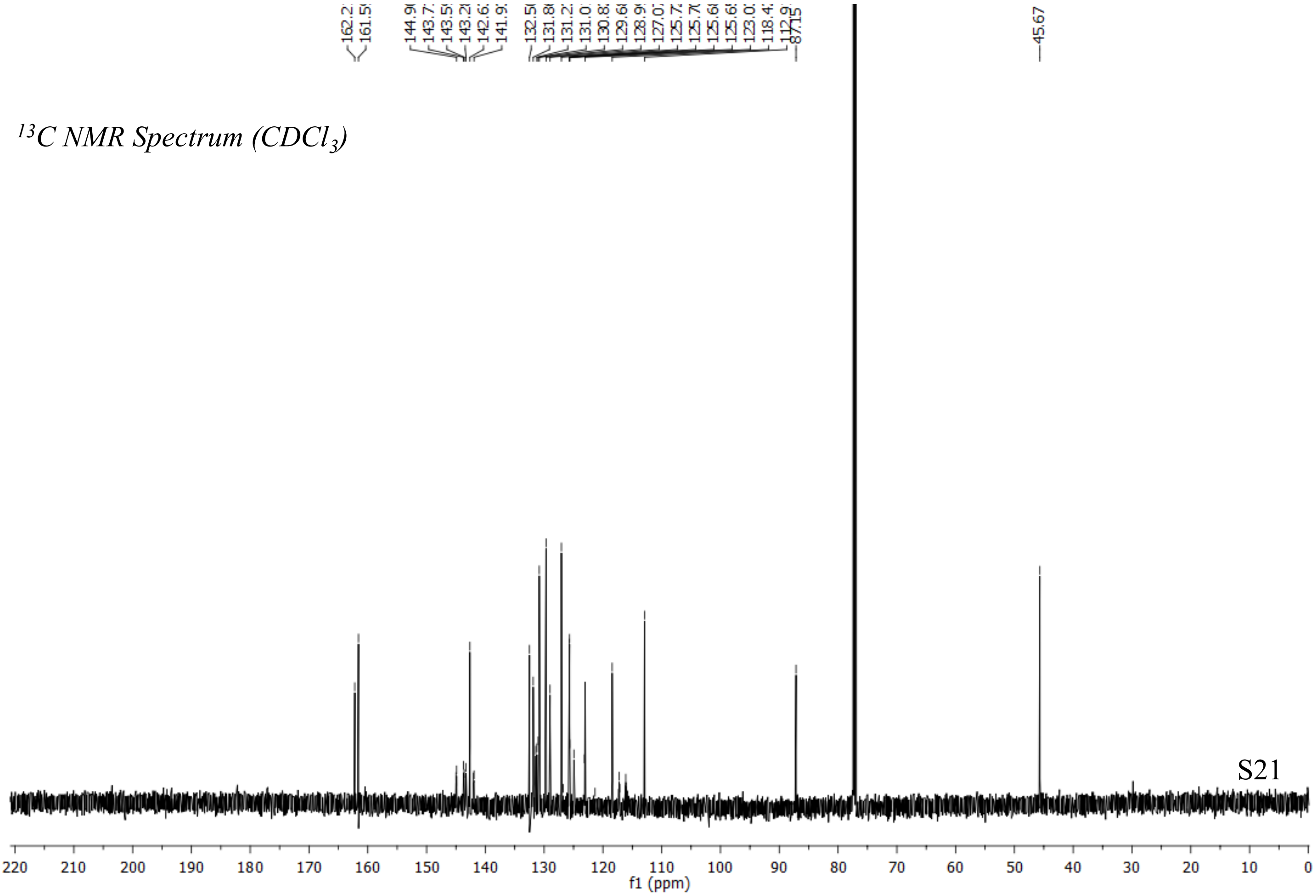

1-Benzyl-2-oxo-N-(2,3,5,6-tetrafluoro-3'-(trifluoromethyl)-[1,1'-biphenyl]-4-yl)-1,2-dihydropyrazolo[1,5-a]pyridine-3-carboxamide (**35**)

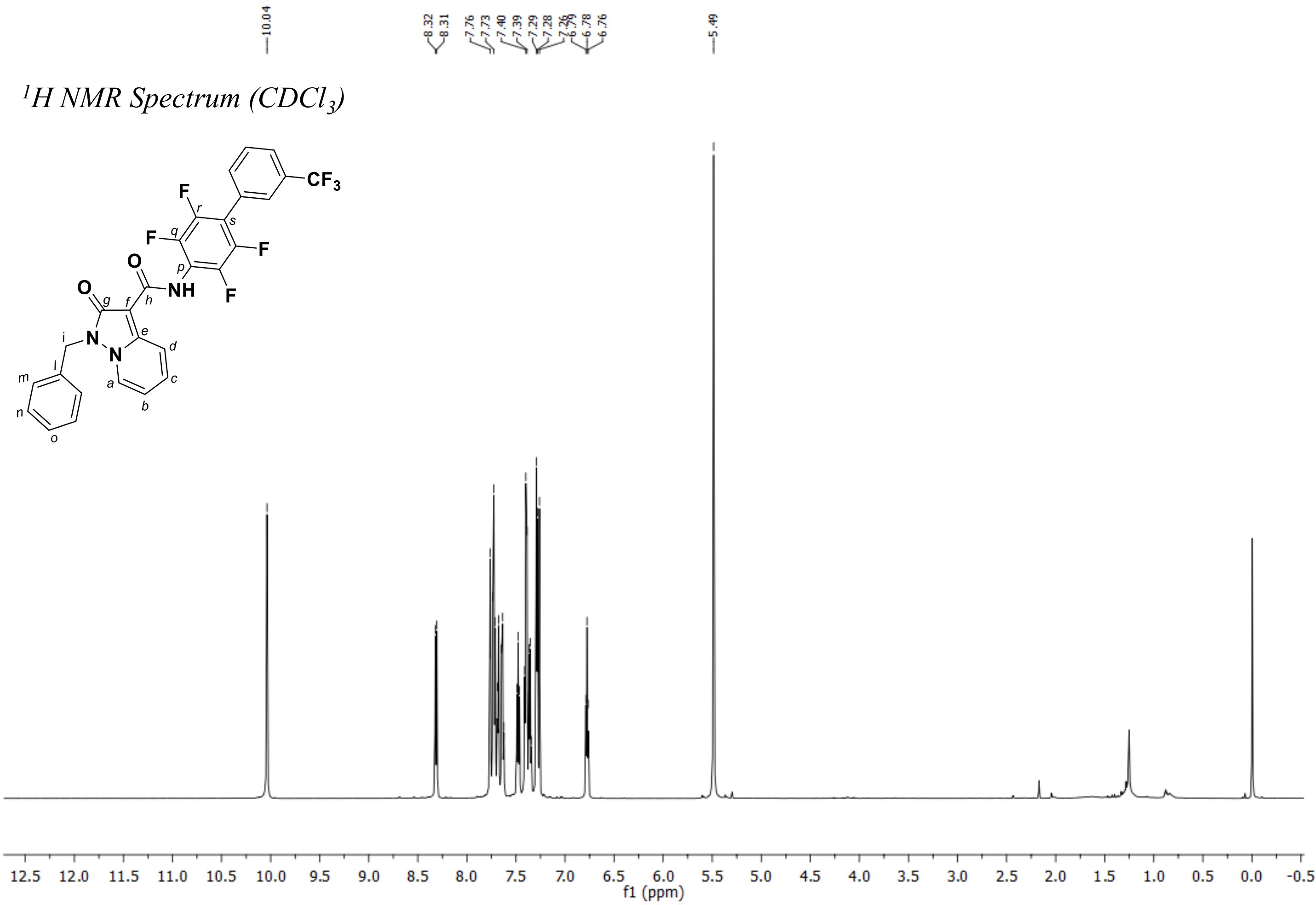

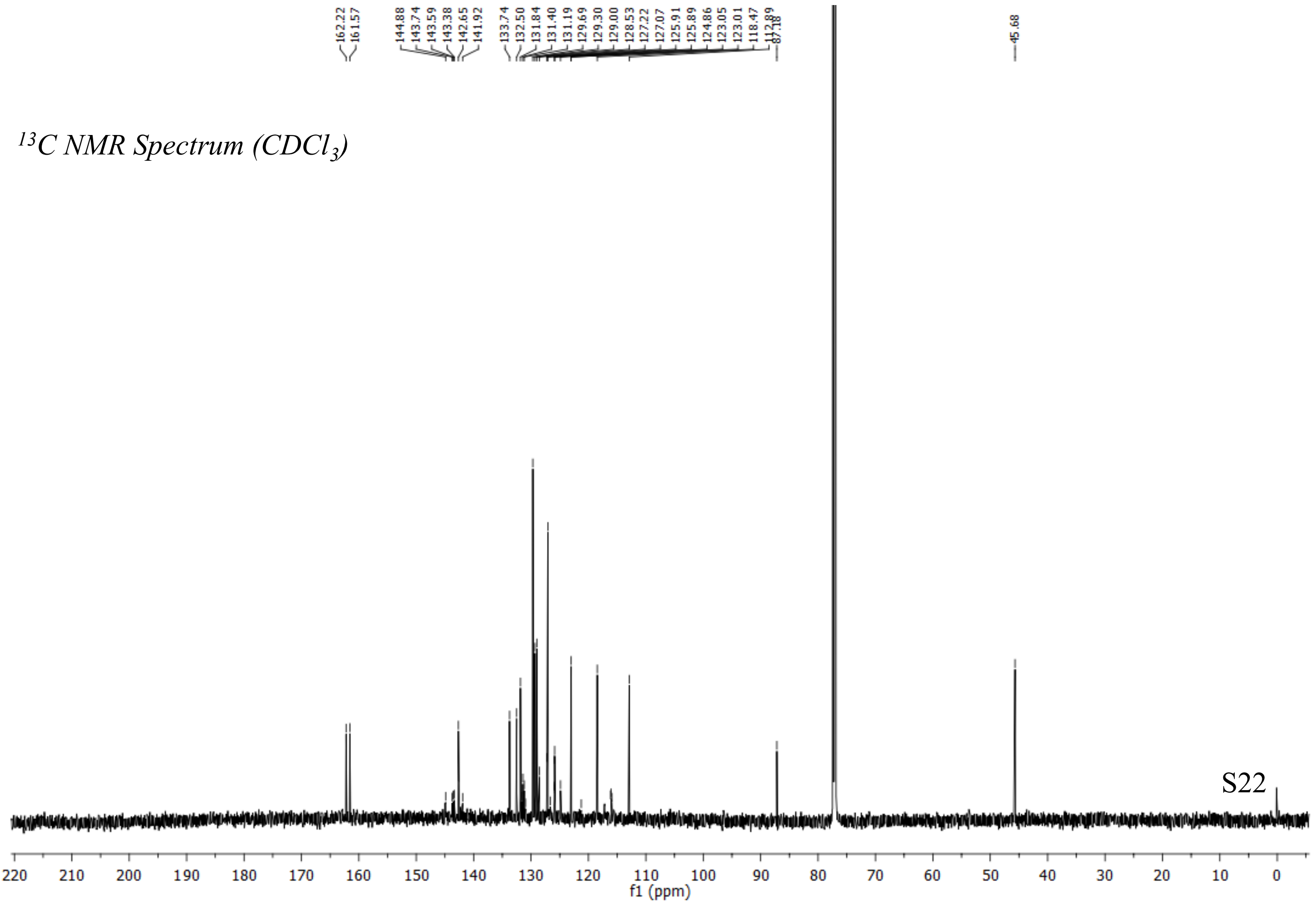

2,3,4',5,6-pentafluoro-[1,1'-biphenyl]-4-aniline (**36**)

^{1}H NMR Spectrum ($CDCl_3$)

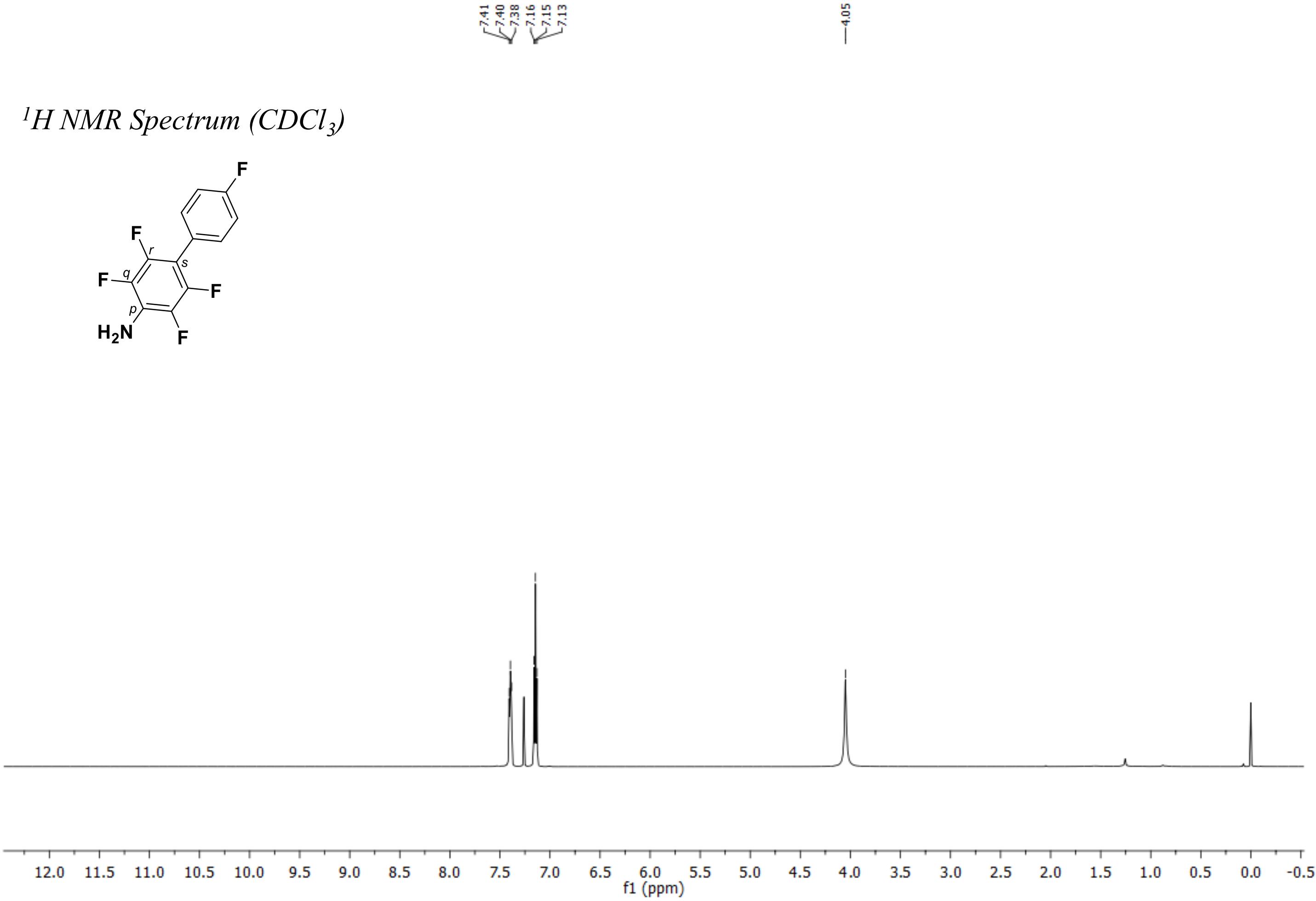

^{13}C NMR Spectrum ($CDCl_3$)

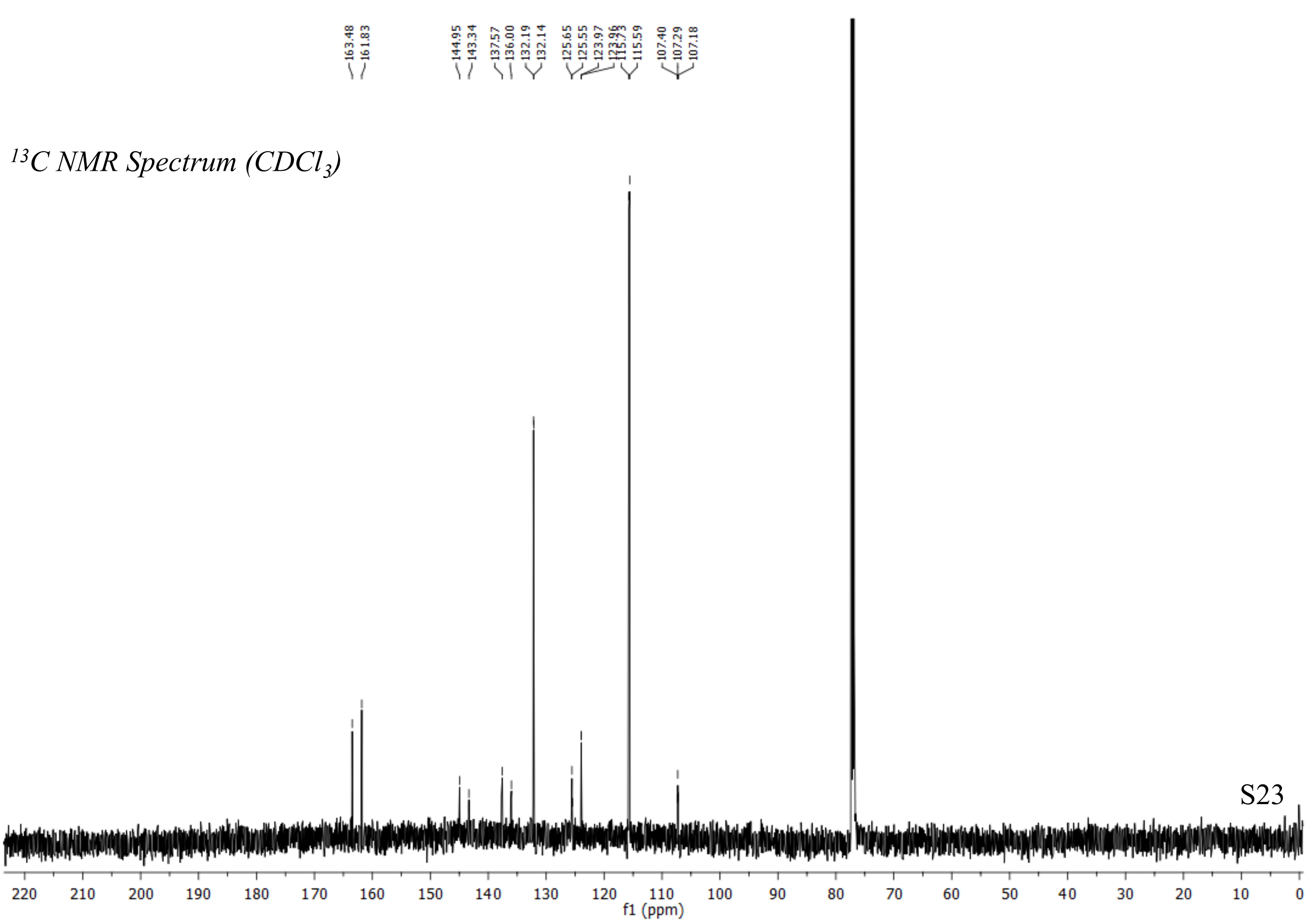

2,3,3',5,6-pentafluoro-[1,1'-biphenyl]-4-aniline (**37**)

^{1}H NMR Spectrum (CDCl$_3$)

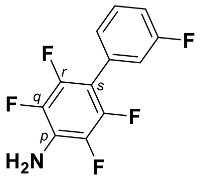

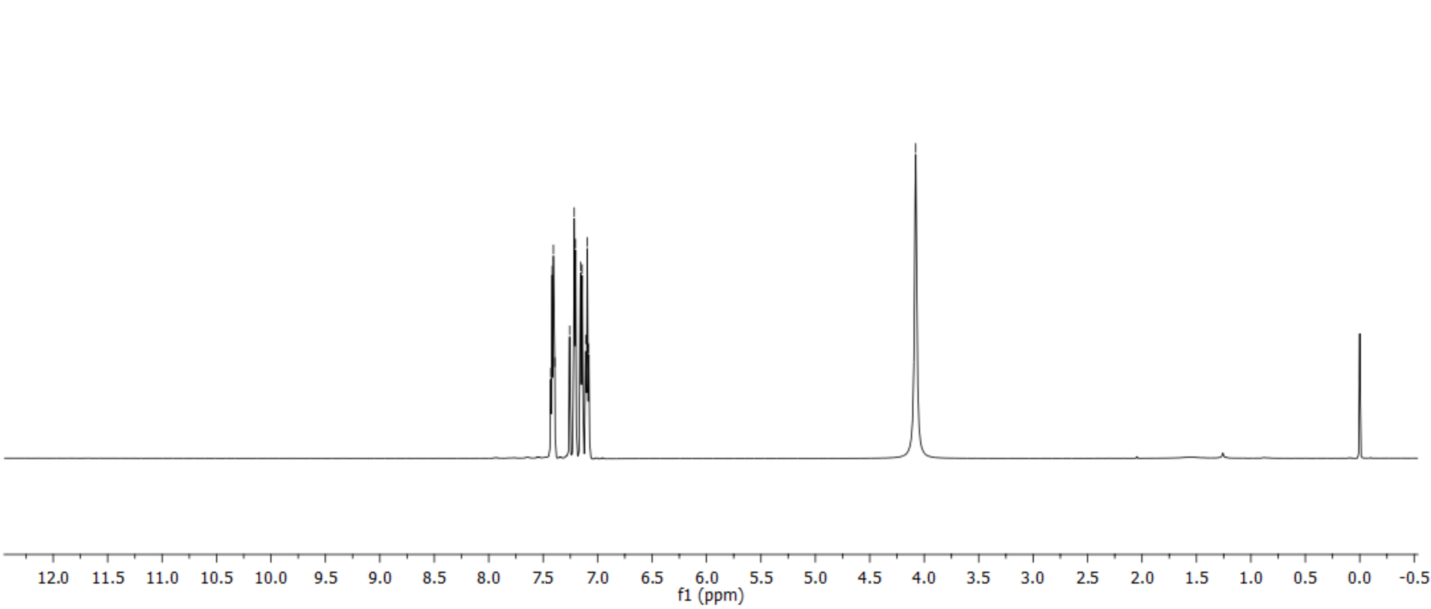

^{13}C NMR Spectrum (CDCl$_3$)

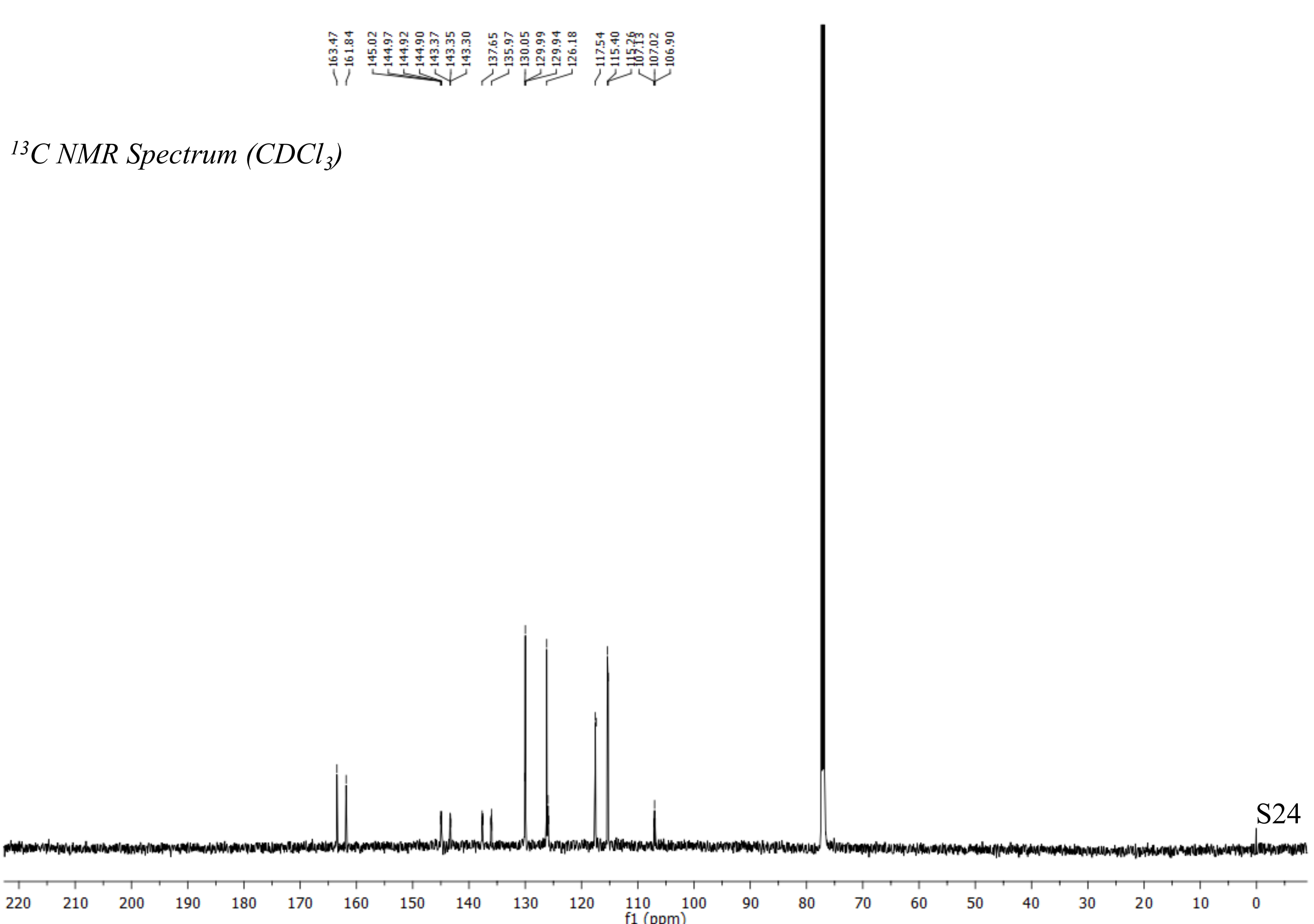

2,3,5,6-tetrafluoro-4'-(trifluoromethyl)-[1,1'-biphenyl]-4-aniline (**38**)

^{1}H NMR Spectrum ($CDCl_3$)

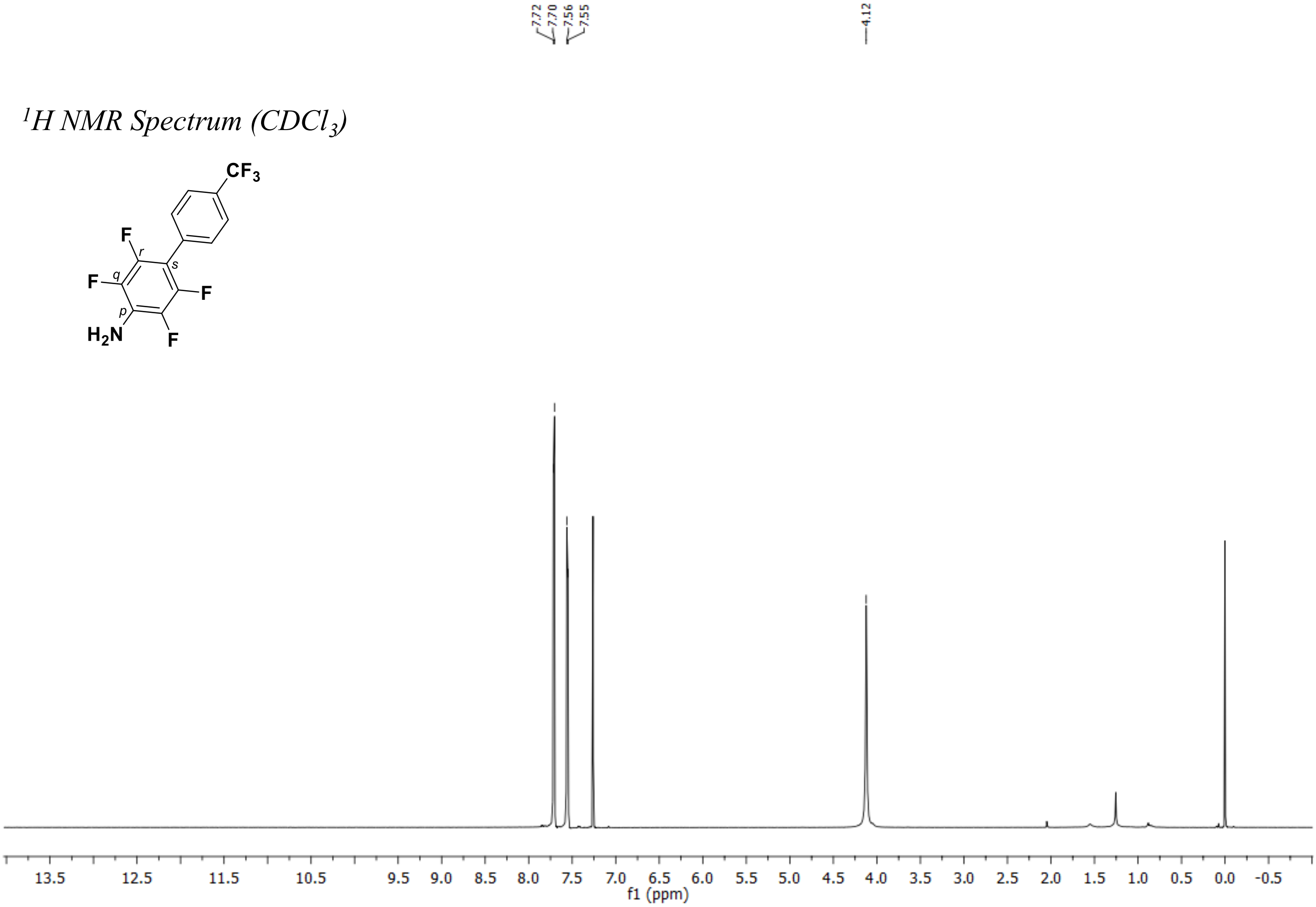

^{13}C NMR Spectrum ($CDCl_3$)

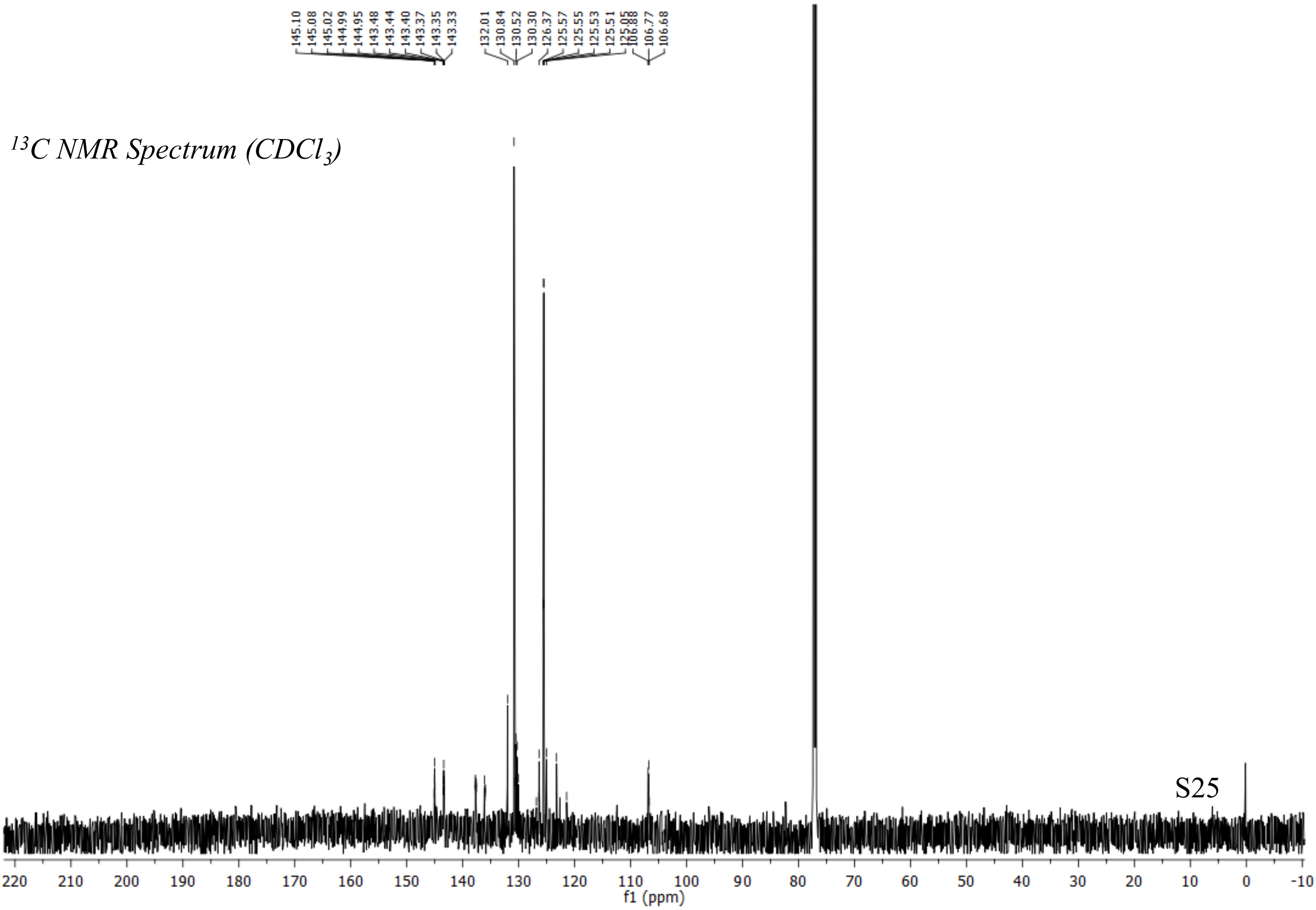

2,3,5,6-tetrafluoro-3'-(trifluoromethyl)-[1,1'-biphenyl]-4-aniline (**39**)

^{1}H NMR Spectrum ($CDCl_3$)

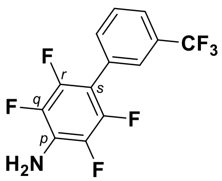

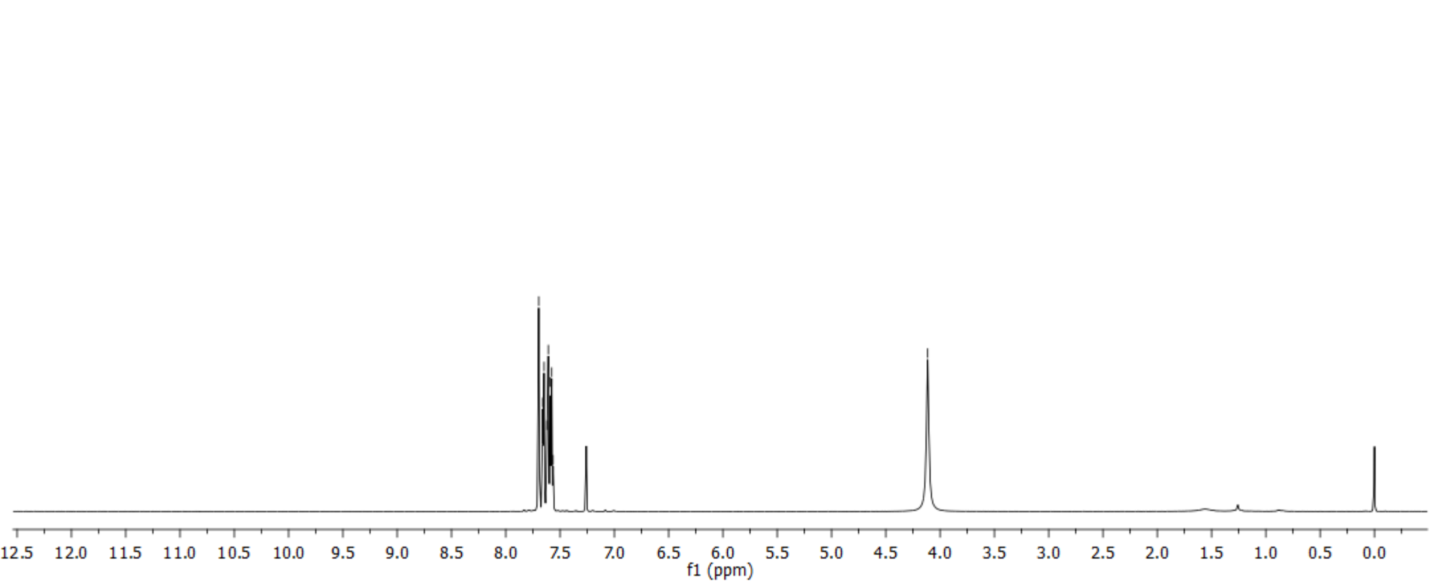

^{13}C NMR Spectrum ($CDCl_3$)

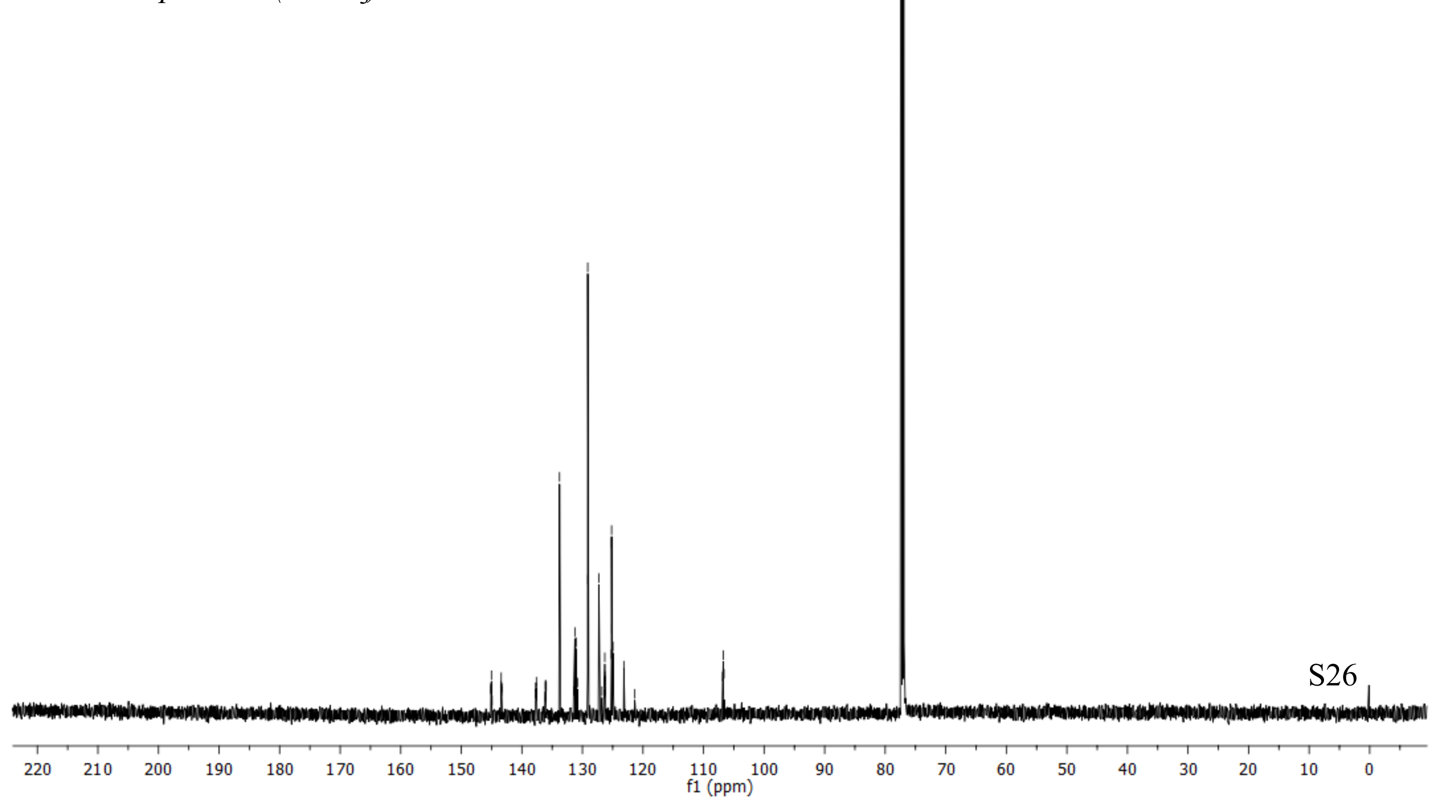

2-Hydroxy-N-(2,3,5,6-tetrafluoro-4-morpholinophenyl)pyrazolo[1,5-a]pyridine-3-carboxamide (**6**)

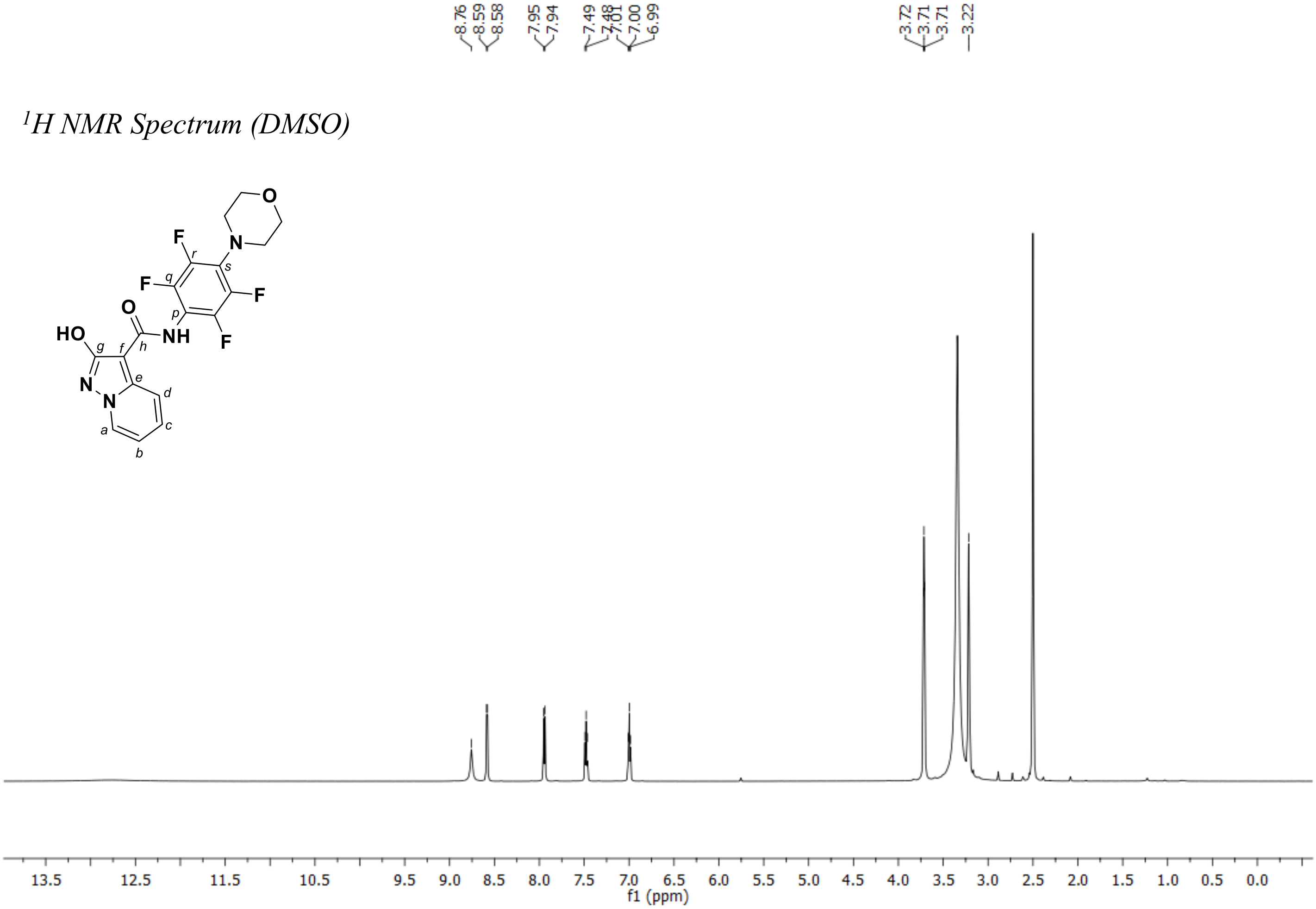

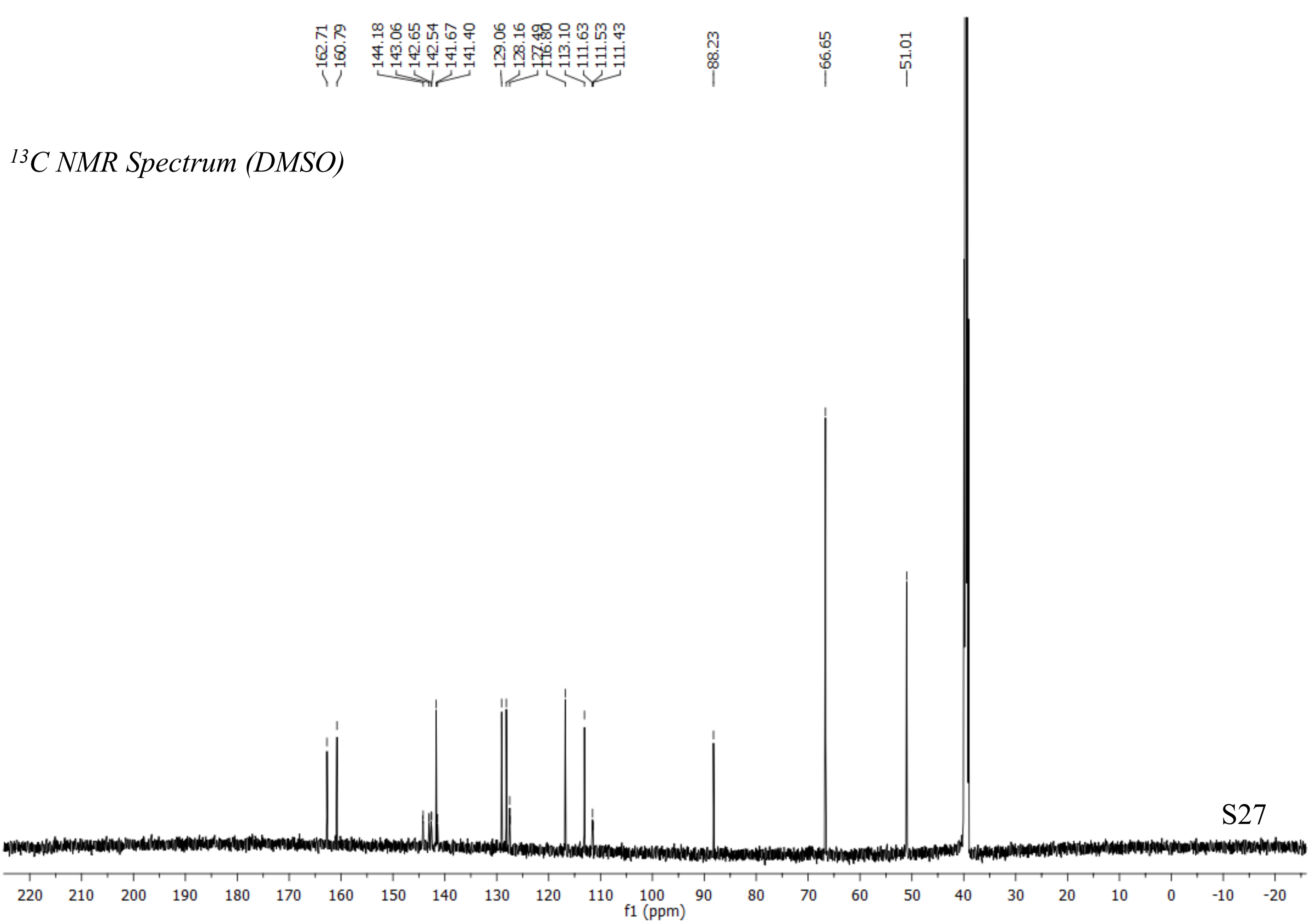

2-Hydroxy-N-(2,3,5,6-tetrafluoro-4-morpholinophenyl)pyrazolo[1,5-a]pyridine-3-carboxamide (**6**)

meds-609__10ng-ul_inf_ms-neg 01/23/20 16:04:35 MEDS-609__10ng-ul_inf_MS-neg

meds-609 10ng-ul inf ms-neg #1-20 RT: 0.01-0.29 AV: 20 NL: 9.56E6
T: FTMS - p ESI Full ms [403.00-430.00]

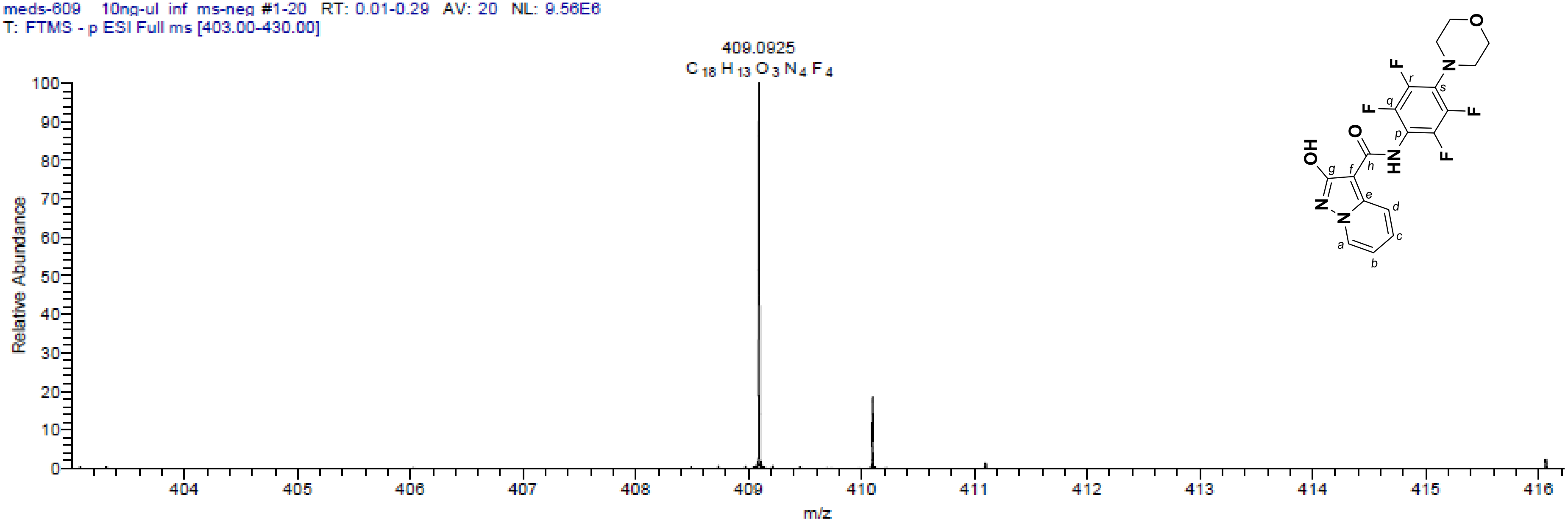

meds-609__10ng-ul_inf_ms2-hcd-70-neg #1-20 RT: 0.01-0.37 AV: 20 NL: 1.46E6
T: FTMS - p ESI Full ms2 409.00@hcd70.00 [50.00-430.00]

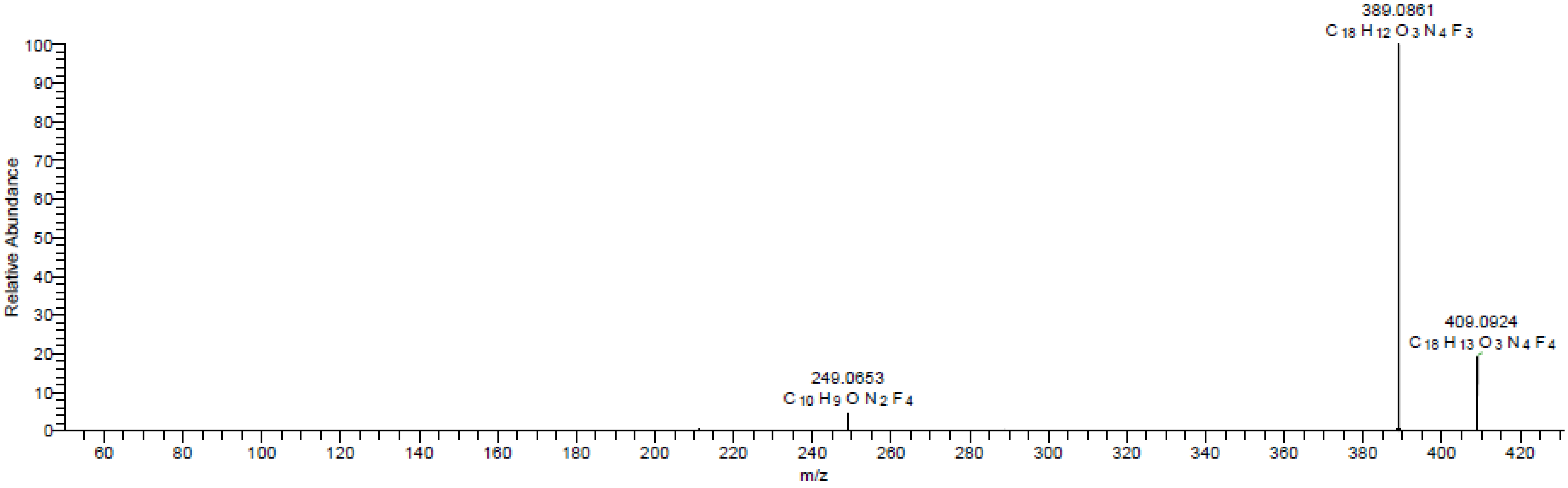

Report UHPLC

Acquisition Date/Time	6/17/2019 3:44:31 PM	Sample Name	MMS334
Acquisition Method	B2 C18_95_ACN	Injection Volume	2
Instrument Name	UHPLC		

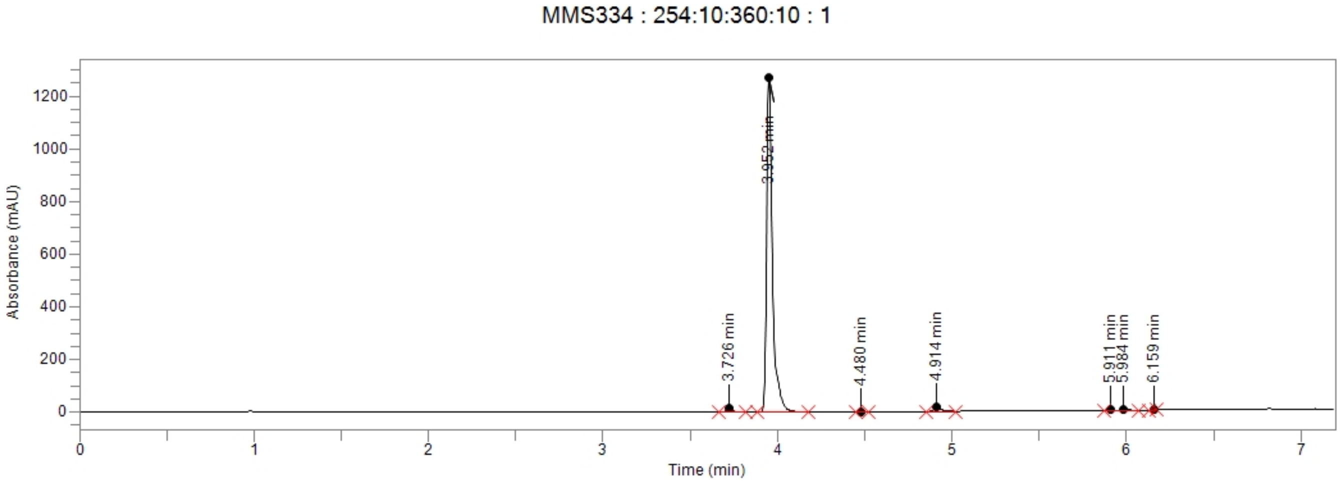

Channel Name **254:10:360:10**

Component Name	Time	Height	Area	Area %	Peak Purity	Wavelength Maximum
	3.726	13,836.6	32,085.9	1.07	1.96Fail	201
	3.952	1,276,726.6	2,880,293.3	96.49	1.22Pass	223
	4.480	545.1	1,029.3	0.03	1.27Pass	224
	4.914	18,989.5	54,643.7	1.83	1.40Pass	224
	5.911	1,444.6	2,843.4	0.10	1.44Pass	228
	5.984	5,735.5	13,721.6	0.46	1.23Pass	228
	6.159	309.6	573.9	0.02	1.12Pass	228
Total			2,985,191.1	100.00		

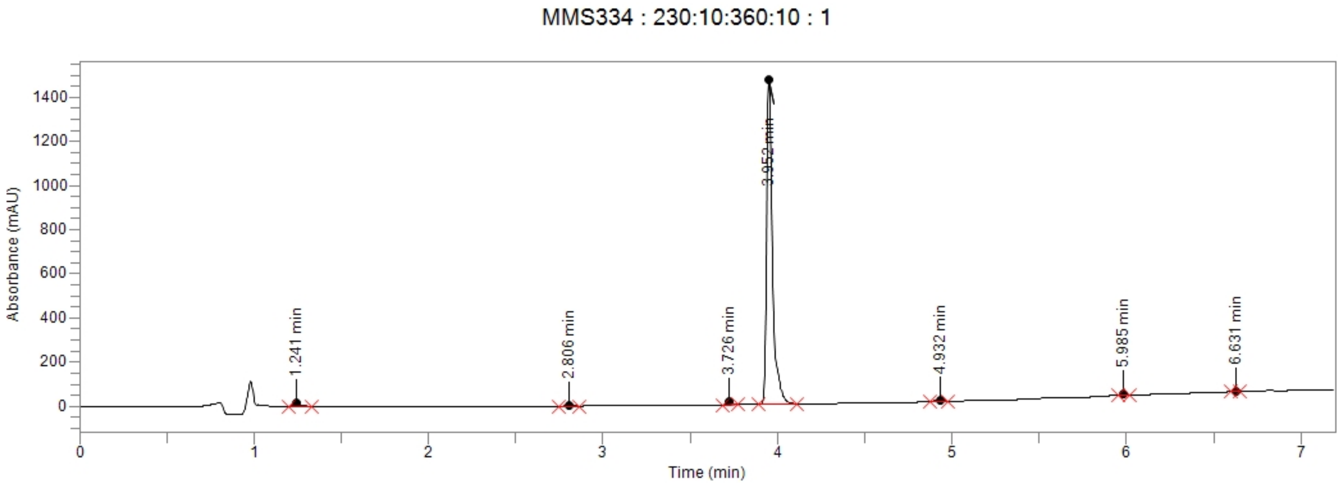

Channel Name **230:10:360:10**

Component Name	Time	Height	Area	Area %	Peak Purity	Wavelength Maximum
	1.241	16,235.0	43,141.3	1.23	4.25Fail	231
	2.806	4,024.1	14,546.7	0.41	34.58Fail	201
	3.726	16,492.6	33,945.4	0.97	2.21Fail	201
	3.952	1,479,467.9	3,376,630.5	96.28	1.22Pass	223
	4.932	7,795.9	25,818.8	0.74	1.82Fail	225
	5.985	5,320.7	10,025.1	0.29	1.12Pass	228
	6.631	1,896.8	3,097.1	0.09	1.07Pass	228
Total			3,507,205.0	100.00		

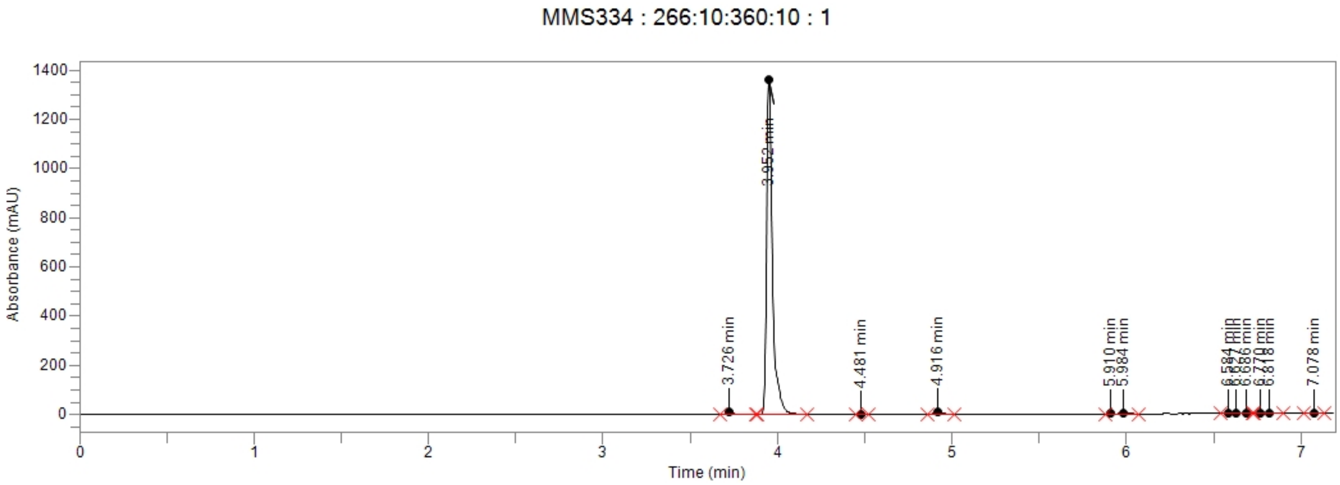

Channel Name **266:10:360:10**

Component Name	Time	Height	Area	Area %	Peak Purity	Wavelength Maximum
	3.726	11,698.0	27,951.3	0.87	1.62Fail	201
	3.952	1,367,831.1	3,104,230.7	97.13	1.22Pass	223
	4.481	548.2	1,015.9	0.03	1.27Pass	224
	4.916	9,676.2	31,058.8	0.97	1.46Pass	224
	5.910	816.8	1,554.7	0.05	1.40Pass	228
	5.984	5,224.0	12,233.9	0.38	1.23Pass	228
	6.584	851.9	1,895.0	0.06	1.12Pass	228
	6.627	991.3	2,230.4	0.07	1.08Pass	228
	6.686	946.4	1,888.5	0.06	1.06Pass	228
	6.770	1,062.4	1,872.8	0.06	1.09Pass	228
	6.818	2,059.5	6,032.7	0.19	1.08Pass	228
	7.078	1,153.1	3,904.8	0.12	1.06Pass	228
Total			3,195,869.7	100.00		

2-Hydroxy-N-(2,3,5,6-tetrafluoro-4-(thiophen-2-yl)phenyl)pyrazolo[1,5-a]pyridine-3-carboxamide (**7**)

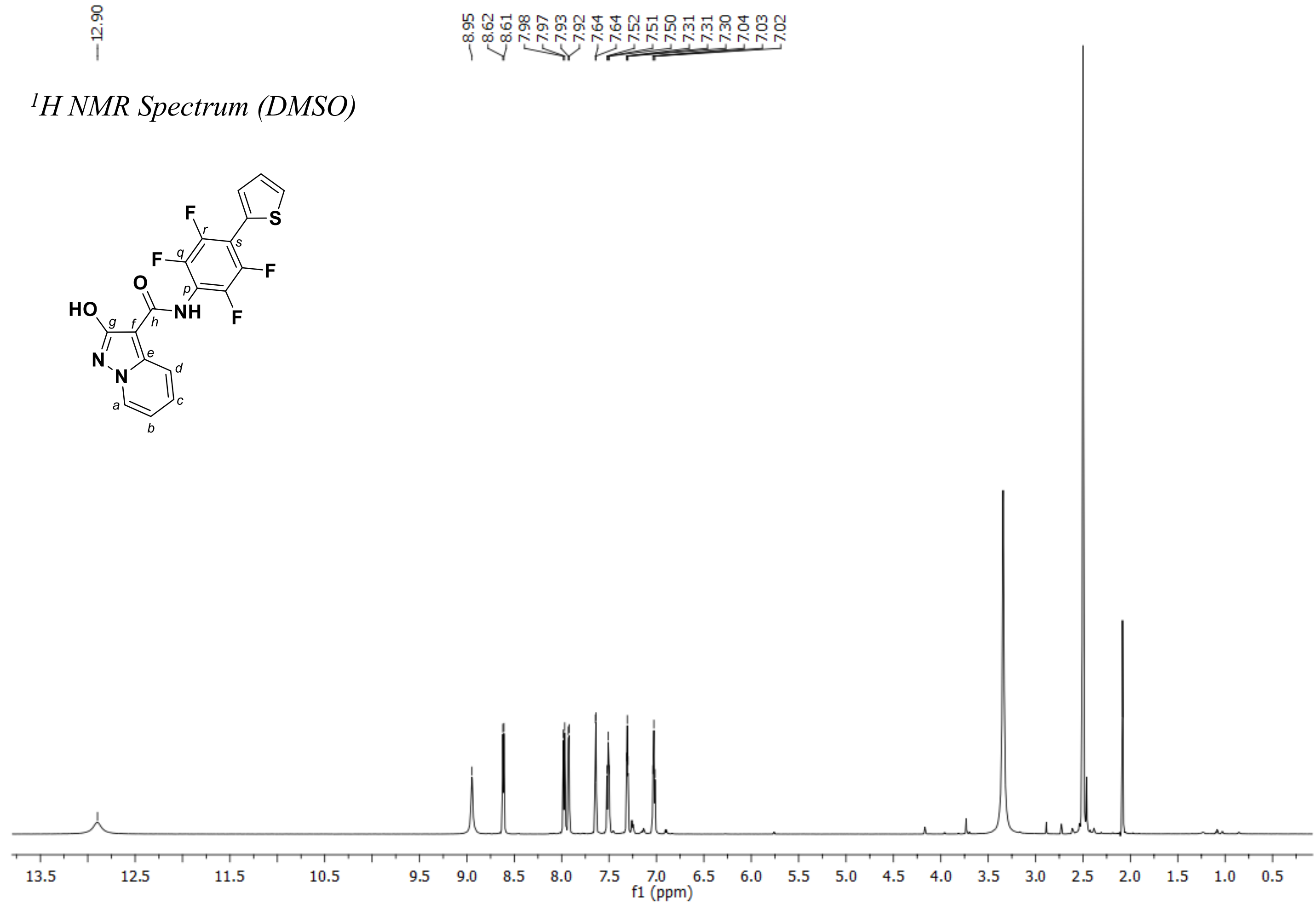

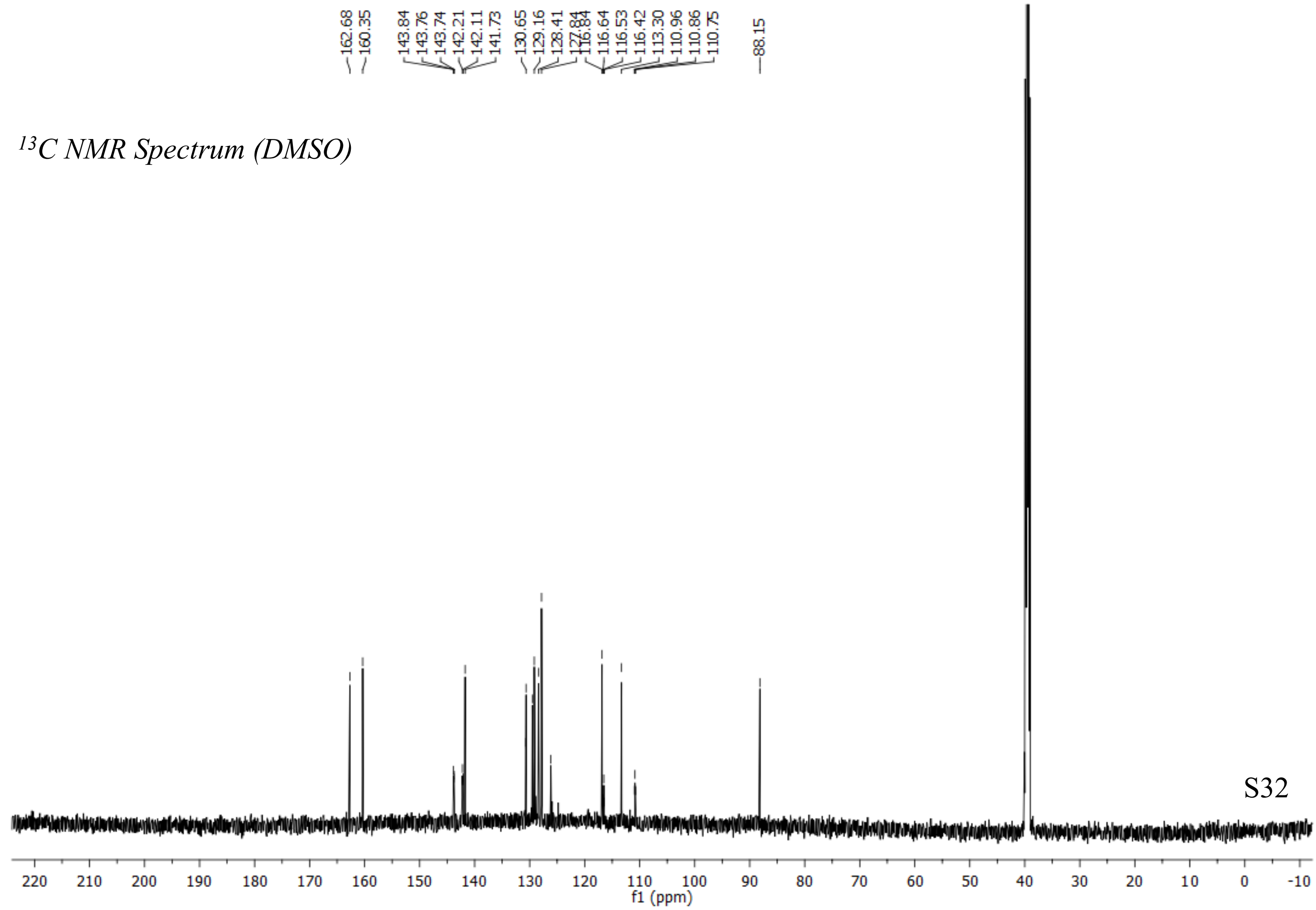

2-Hydroxy-N-(2,3,5,6-tetrafluoro-4-(thiophen-2-yl)phenyl)pyrazolo[1,5-a]pyridine-3-carboxamide (**7**)

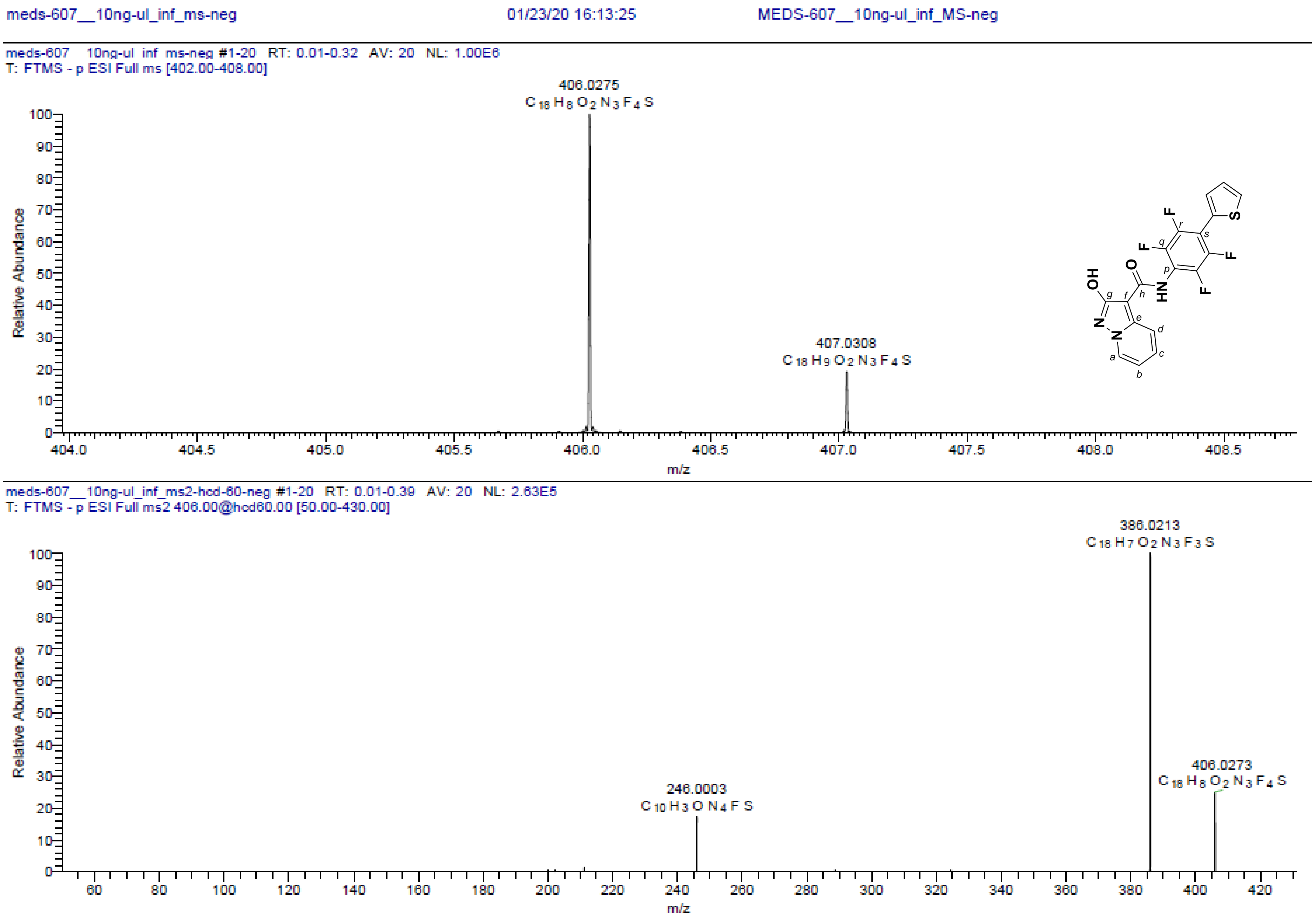

Report UHPLC

Acquisition Date/Time	5/16/2019 4:08:20 PM	Sample Name	MMS266G
Acquisition Method	DHODH screening	Injection Volume	2
Instrument Name	UHPLC		

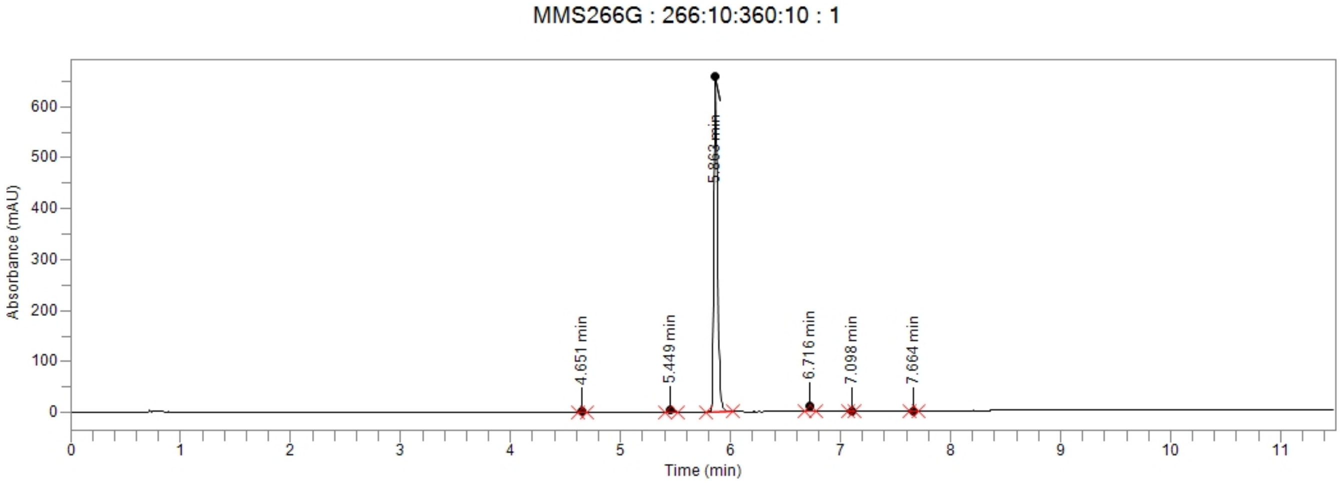

Channel Name **266:10:360:10**

Component Name	Time	Height	Area	Area %	Peak Purity	Wavelength Maximum
	4.651	1,416.5	2,881.3	0.18	1.87Fail	200
	5.449	3,889.6	10,473.4	0.65	2.48Fail	200
	5.863	658,435.6	1,566,752.1	97.79	1.46Pass	223
	6.716	10,393.4	20,214.4	1.26	1.14Pass	225
	7.098	466.9	726.4	0.05	1.08Pass	227
	7.664	599.4	1,107.4	0.07	1.05Pass	228
Total			1,602,155.0	100.00		

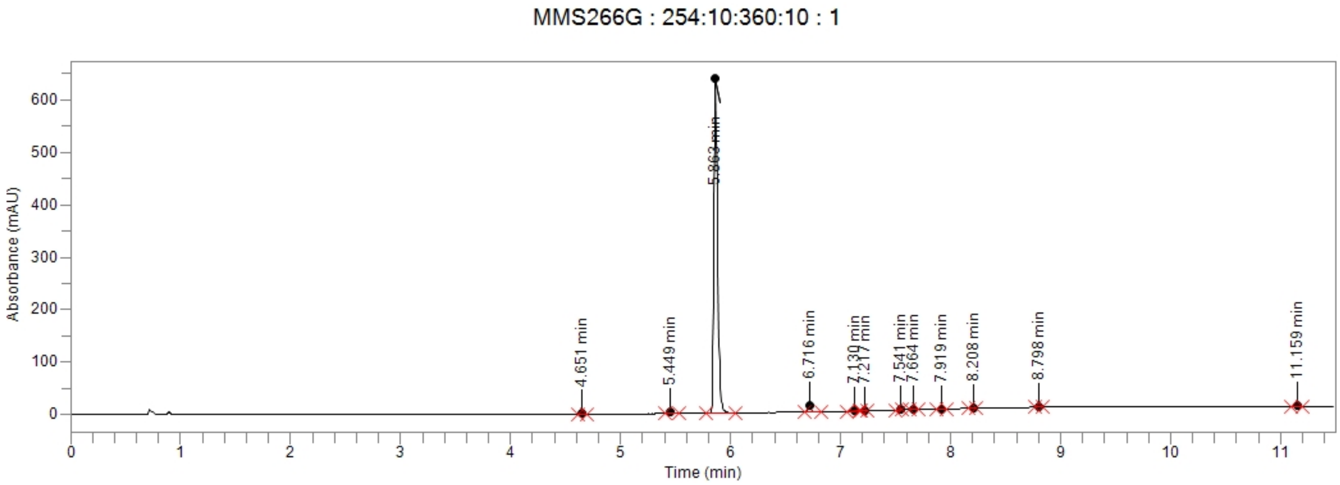

Channel Name **254:10:360:10**

Component Name	Time	Height	Area	Area %	Peak Purity	Wavelength Maximum
	4.651	1,029.8	1,983.5	0.13	1.28Pass	200
	5.449	4,049.7	11,593.9	0.73	2.52Fail	200
	5.863	638,393.7	1,530,054.6	96.95	1.46Pass	223
	6.716	11,547.1	23,465.6	1.49	1.07Pass	225
	7.130	850.4	3,369.5	0.21	1.08Pass	227
	7.217	442.6	704.7	0.04	1.03Pass	227
	7.541	449.6	912.3	0.06	1.05Pass	228
	7.664	593.2	1,073.7	0.07	1.04Pass	228
	7.919	340.8	1,352.6	0.09	1.11Pass	228
	8.208	351.6	940.7	0.06	1.13Pass	228
	8.798	537.8	1,013.6	0.06	1.06Pass	229
	11.159	511.5	1,793.4	0.11	1.04Pass	229
Total			1,578,258.0	100.00		

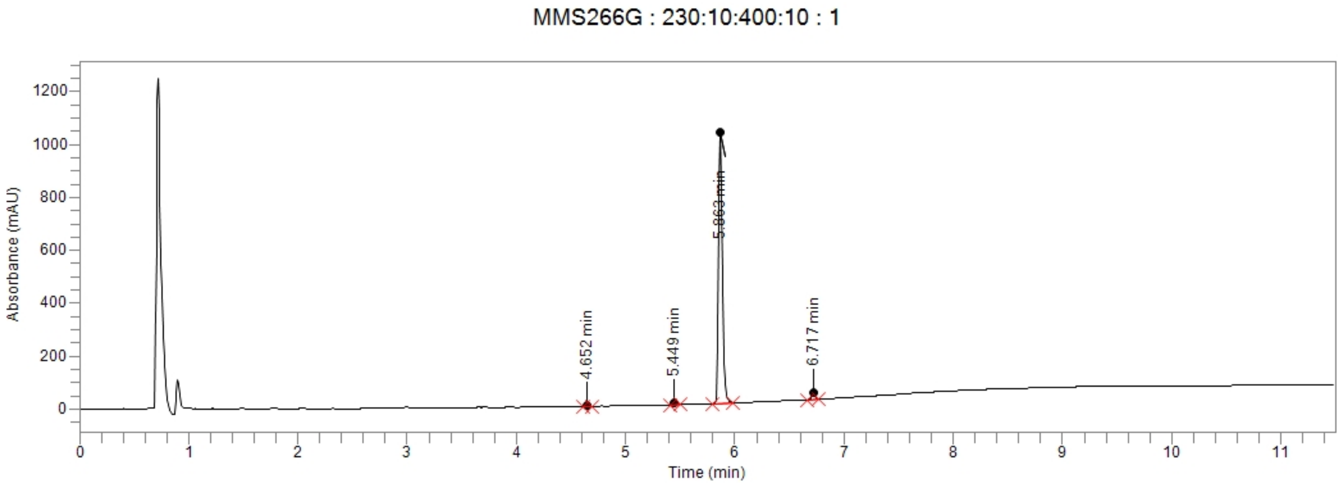

Channel Name **230:10:400:10**

Component Name	Time	Height	Area	Area %	Peak Purity	Wavelength Maximum
	4.652	2,758.6	5,515.4	0.22	1.65Fail	200
	5.449	5,418.1	13,437.9	0.54	6.07Fail	200
	5.863	1,028,041.2	2,433,425.2	97.37	1.46Pass	223
	6.717	23,723.7	46,825.5	1.87	1.14Pass	225
Total			2,499,204.1	100.00		

2-Hydroxy-N-(2,3,5,6-tetrafluoro-4-(pyridin-3-yl)phenyl)pyrazolo[1,5-a]pyridine-3-carboxamide (**8**)

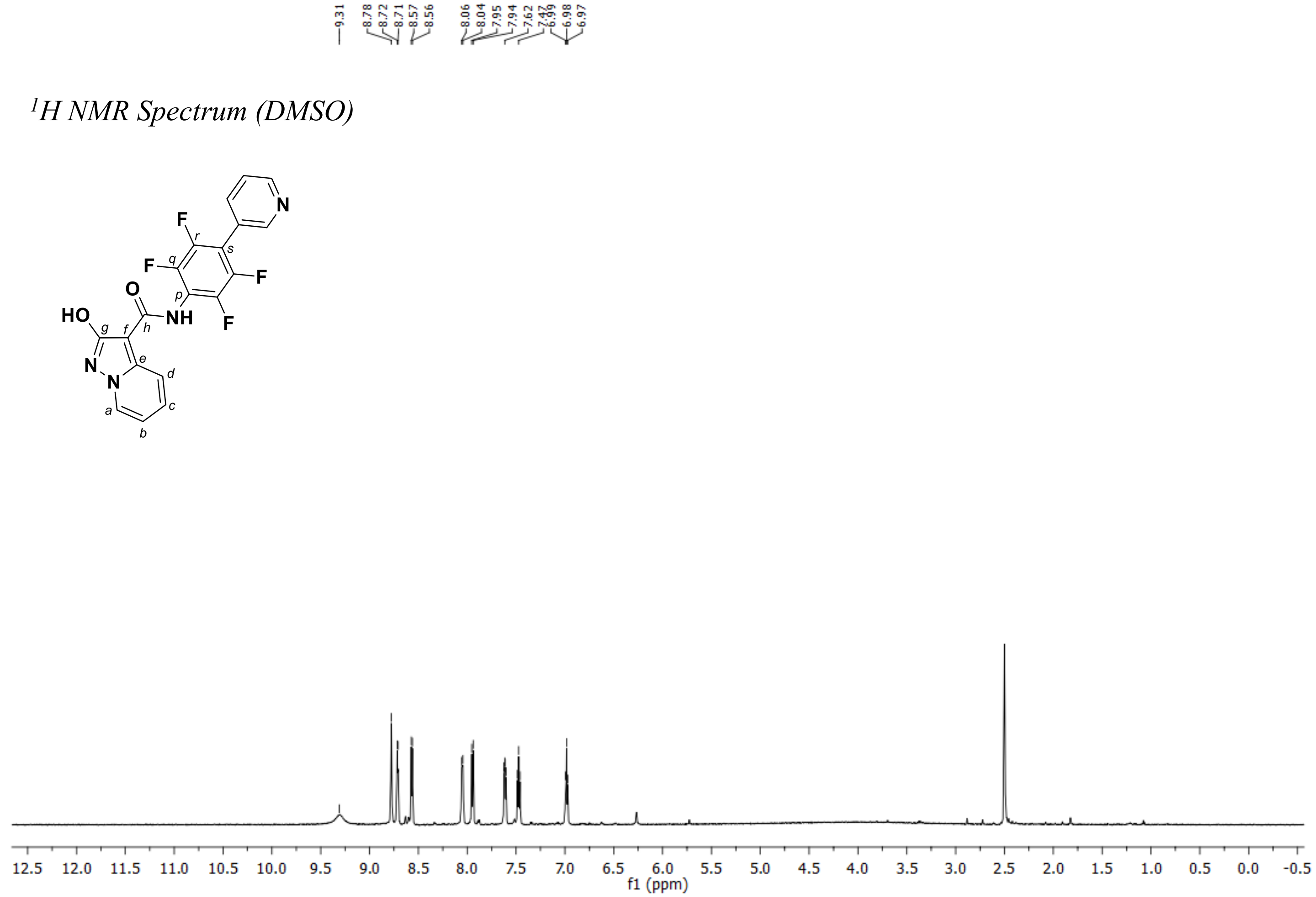

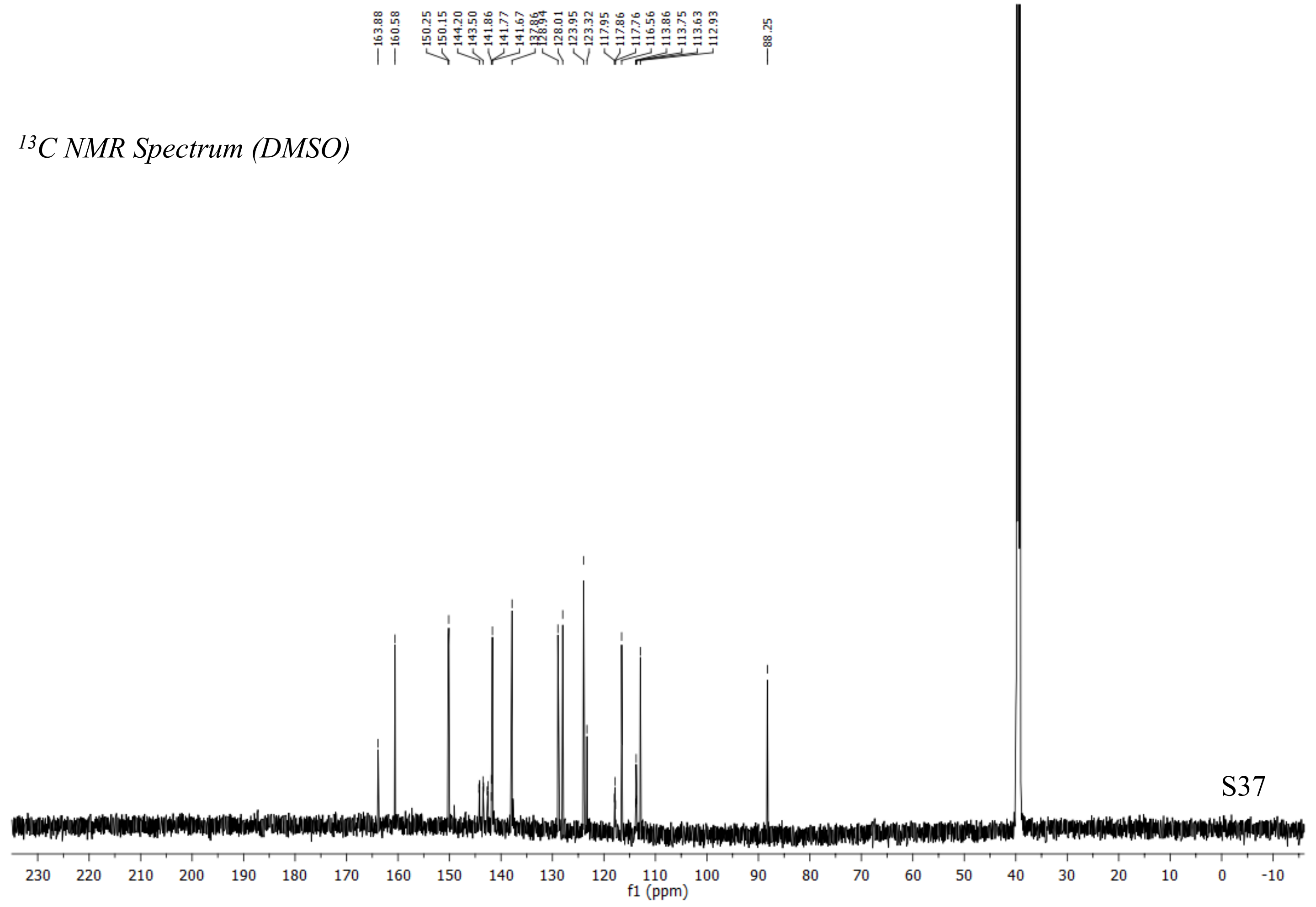

2-Hydroxy-N-(2,3,5,6-tetrafluoro-4-(pyridin-3-yl)phenyl)pyrazolo[1,5-a]pyridine-3-carboxamide (**8**)

meds-604__10ng-ul_inf_ms-pos 01/23/20 12:12:46 MEDS-604__10ng-ul_inf_MS-pos

meds-604 10ng-ul inf ms-pos #1-20 RT: 0.00-0.28 AV: 20 NL: 2.79E7
T: FTMS + p ESI Full ms [399.00-440.00]

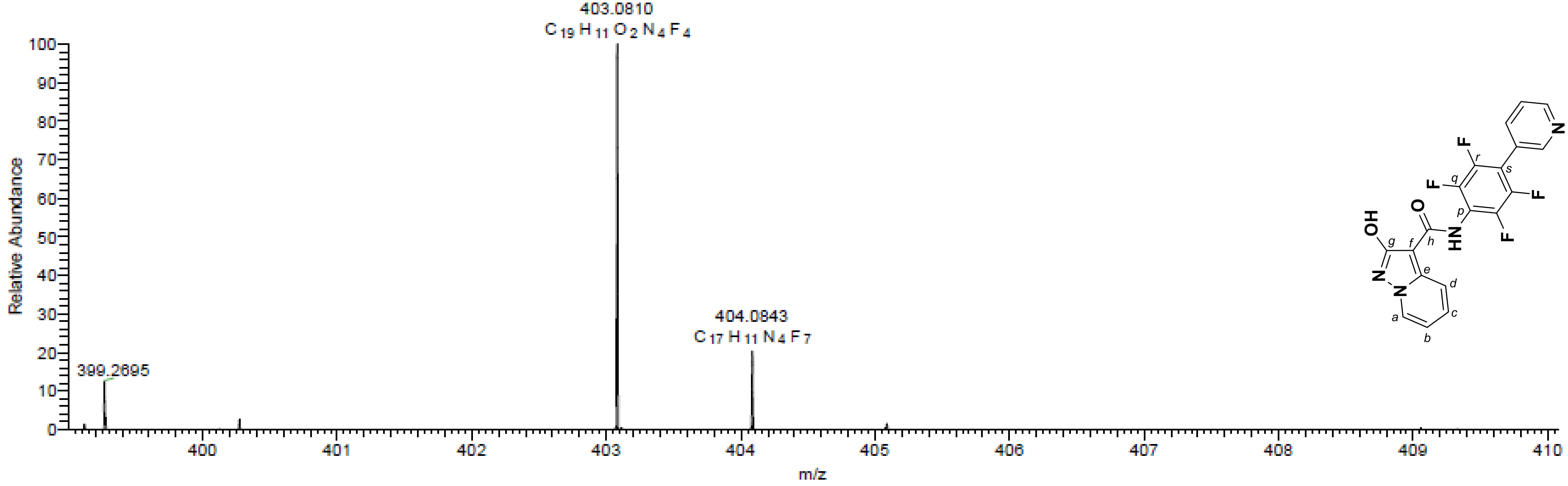

meds-604__10ng-ul_inf_ms2-hcd-23-pos #1-20 RT: 0.01-0.33 AV: 20 NL: 8.31E6
T: FTMS + p ESI Full ms2 403.00@hcd23.00 [50.00-450.00]

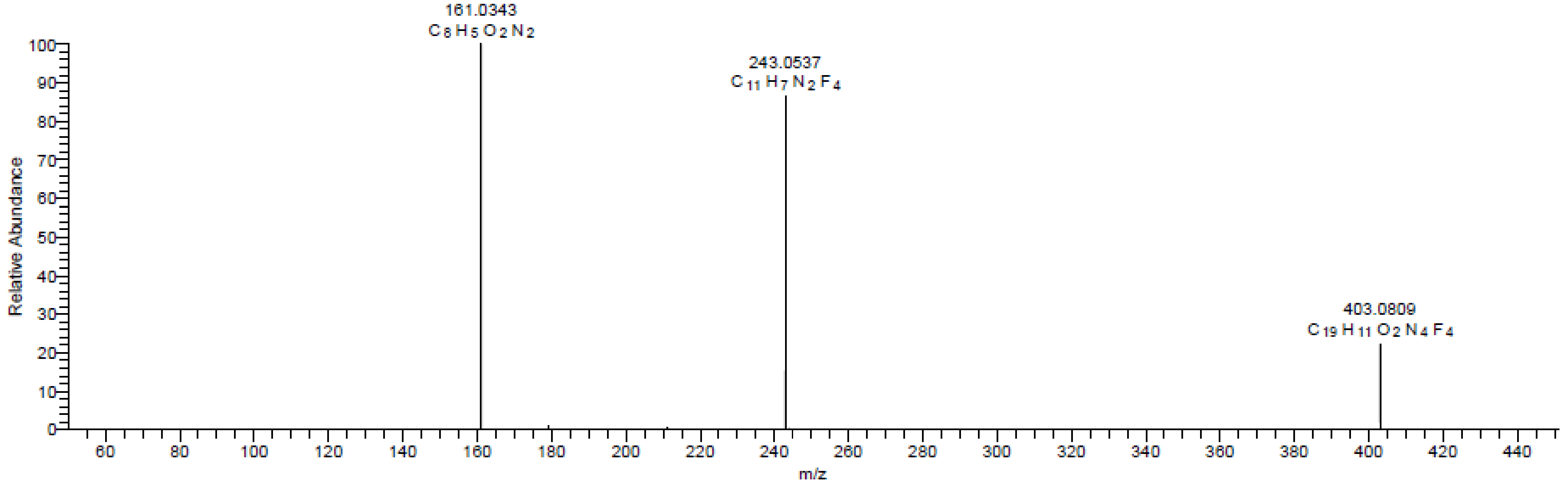

Report UHPLC

Acquisition Date/Time	7/9/2020 5:30:40 PM	Sample Name	M604
Acquisition Method	C18_03_Polar2	Injection Volume	2
Instrument Name	UHPLC		

M604 : 280:10:360:10 : 1

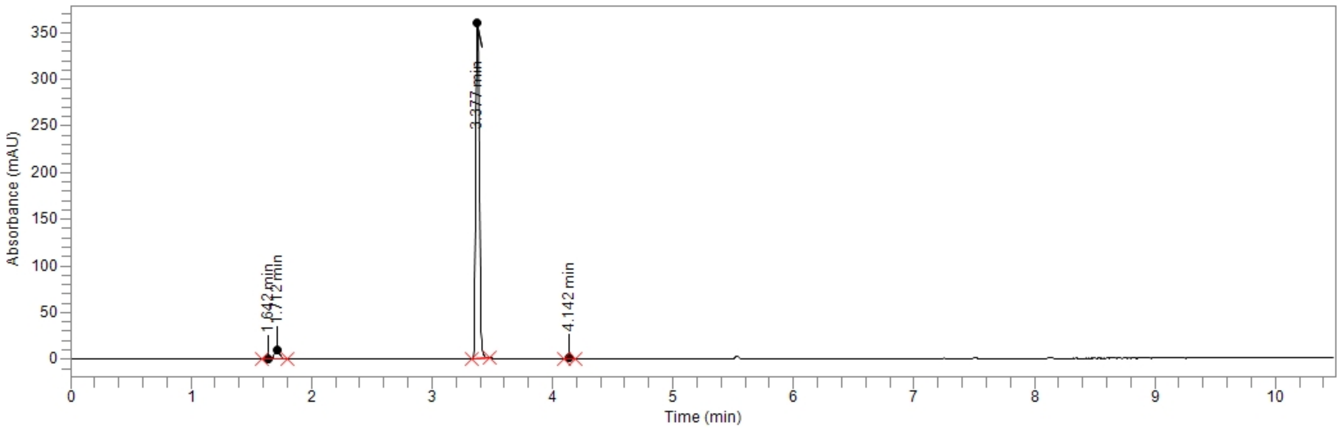

Channel Name **280:10:360:10**

Component Name	Time	Height	Area	Area %	Peak Purity	Wavelength Maximum
	1.642	779.5	2,077.1	0.28		
	1.712	10,118.1	31,757.7	4.26		
	3.377	359,213.2	708,334.0	95.11		
	4.142	1,221.3	2,613.6	0.35		
Total			744,782.4	100.00		

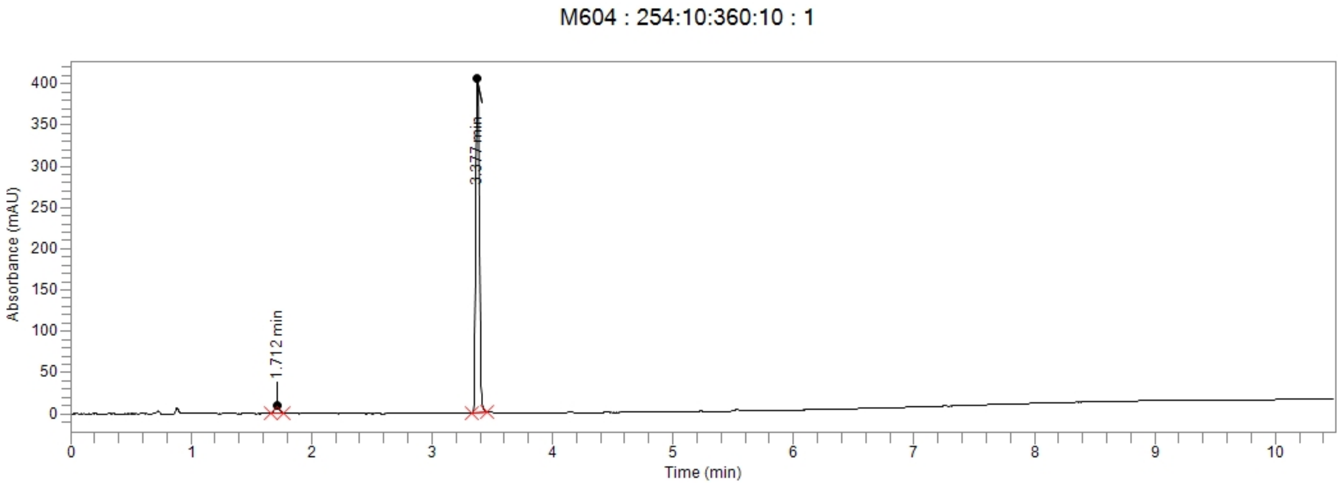

Channel Name **254:10:360:10**

Component Name	Time	Height	Area	Area %	Peak Purity	Wavelength Maximum
	1.712	8,904.0	26,285.5	3.19		
	3.377	404,450.3	796,440.9	96.81		
Total			822,726.4	100.00		

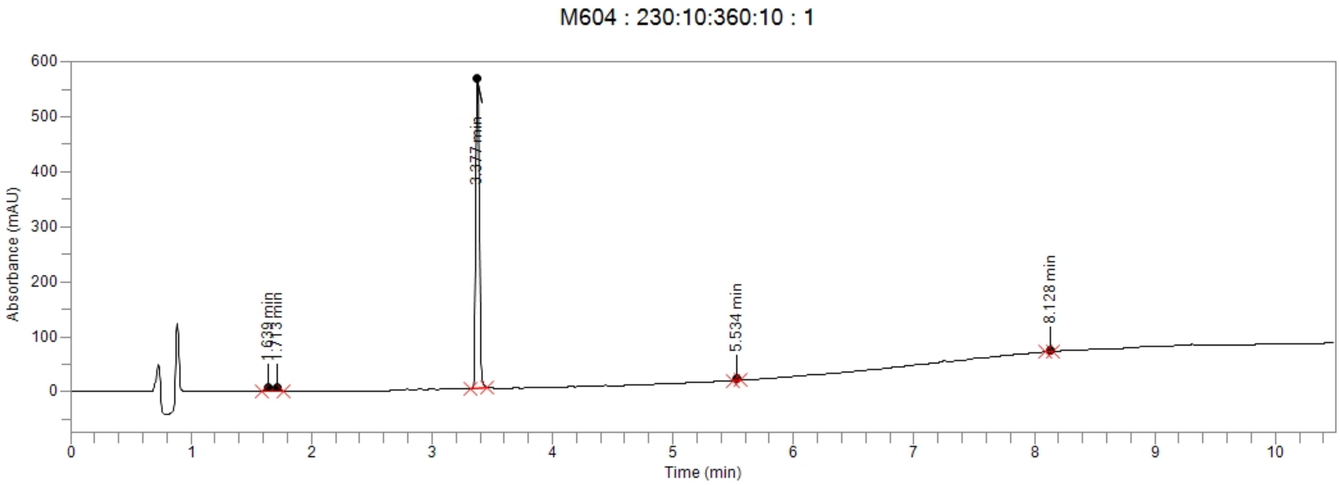

Channel Name **230:10:360:10**

Component Name	Time	Height	Area	Area %	Peak Purity	Wavelength Maximum
	1.639	7,291.2	21,023.3	1.80		
	1.713	7,349.7	22,921.7	1.96		
	3.377	562,943.0	1,114,187.5	95.24		
	5.534	2,951.2	5,585.3	0.48		
	8.128	3,357.1	6,099.8	0.52		
Total			1,169,817.6	100.00		

M604 : 262:10:360:10 : 1

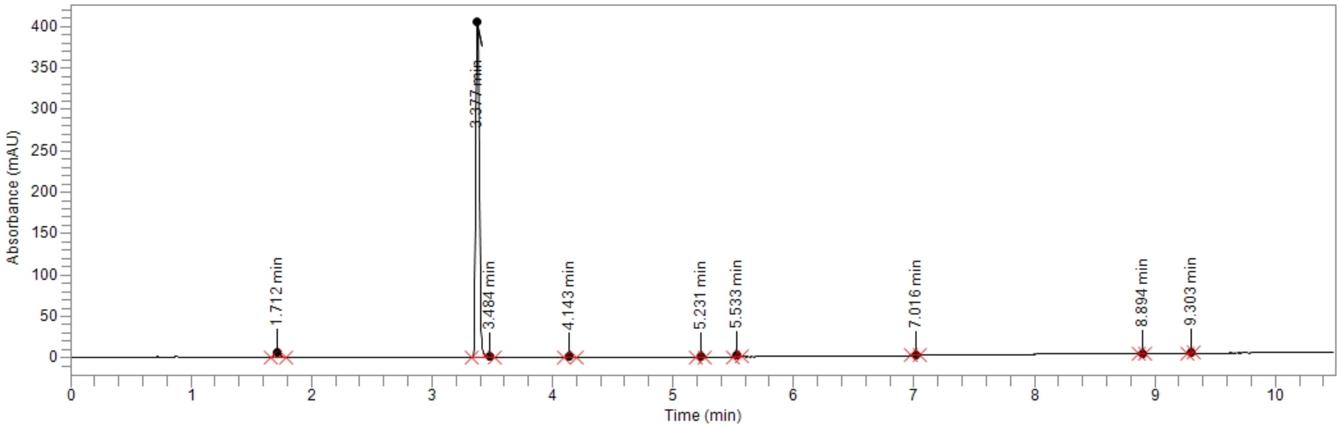

Channel Name **262:10:360:10**

Component Name	Time	Height	Area	Area %	Peak Purity	Wavelength Maximum
	1.712	6,887.3	20,727.3	2.48		
	3.377	404,396.3	799,230.3	95.71		
	3.484	1,151.0	3,402.4	0.41		
	4.143	1,840.1	4,030.6	0.48		
	5.231	692.1	1,204.5	0.14		
	5.533	2,267.4	4,631.6	0.55		
	7.016	540.8	1,038.2	0.12		
	8.894	289.5	497.0	0.06		
	9.303	216.6	302.8	0.04		
Total			835,064.8	100.00		

2-Hydroxy-N-(2,3,5,6-tetrafluoro-4-(2-(trifluoromethyl)pyridin-4-yl)phenyl)pyrazolo[1,5-a]pyridine-3-carboxamide (**9**)

^{1}H NMR Spectrum (DMSO)

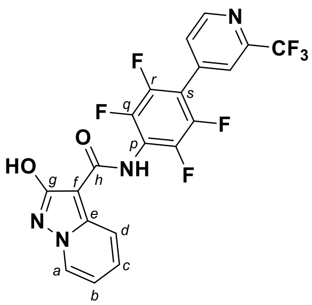

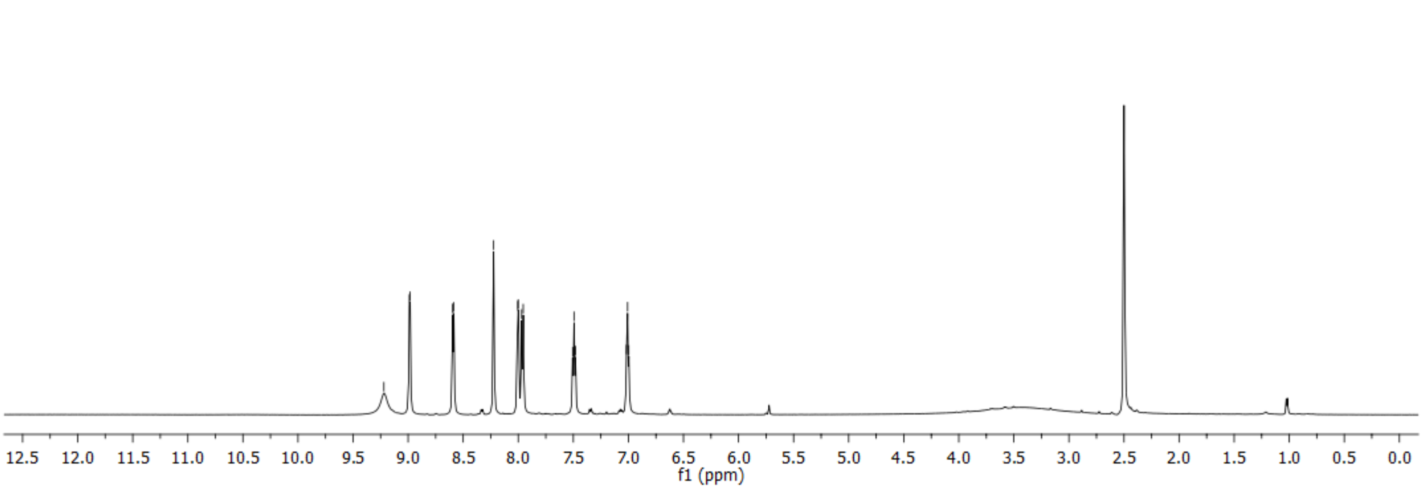

^{13}C NMR Spectrum (DMSO)

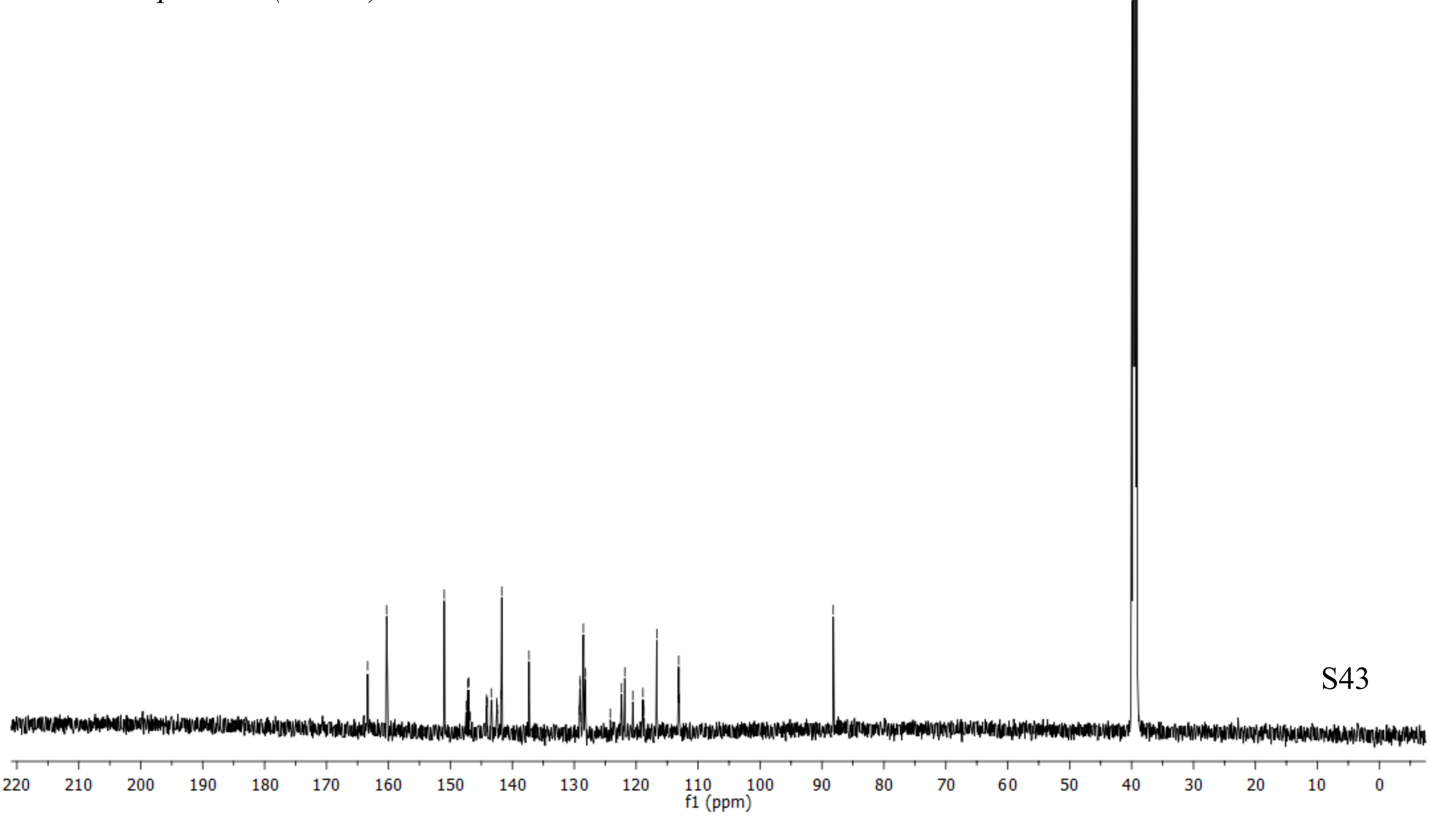

2-Hydroxy-N-(2,3,5,6-tetrafluoro-4-(2-(trifluoromethyl)pyridin-4-yl)phenyl)pyrazolo[1,5-a]pyridine-3-carboxamide (**9**)

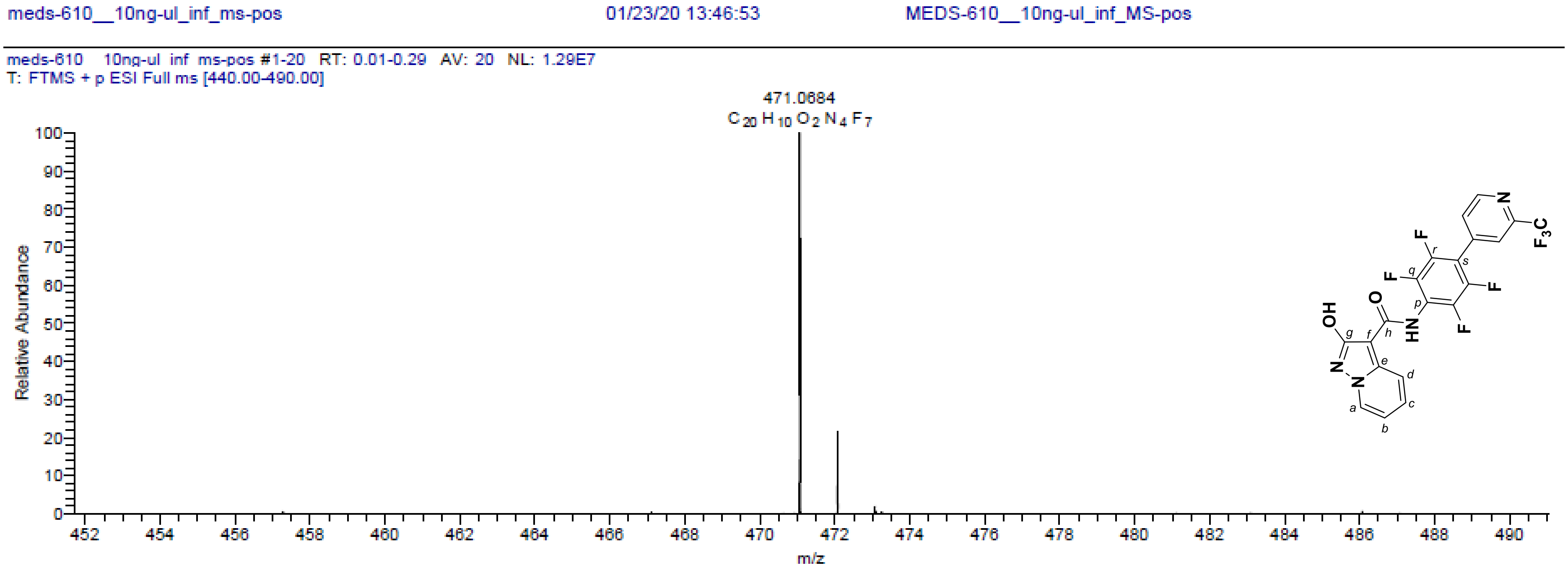

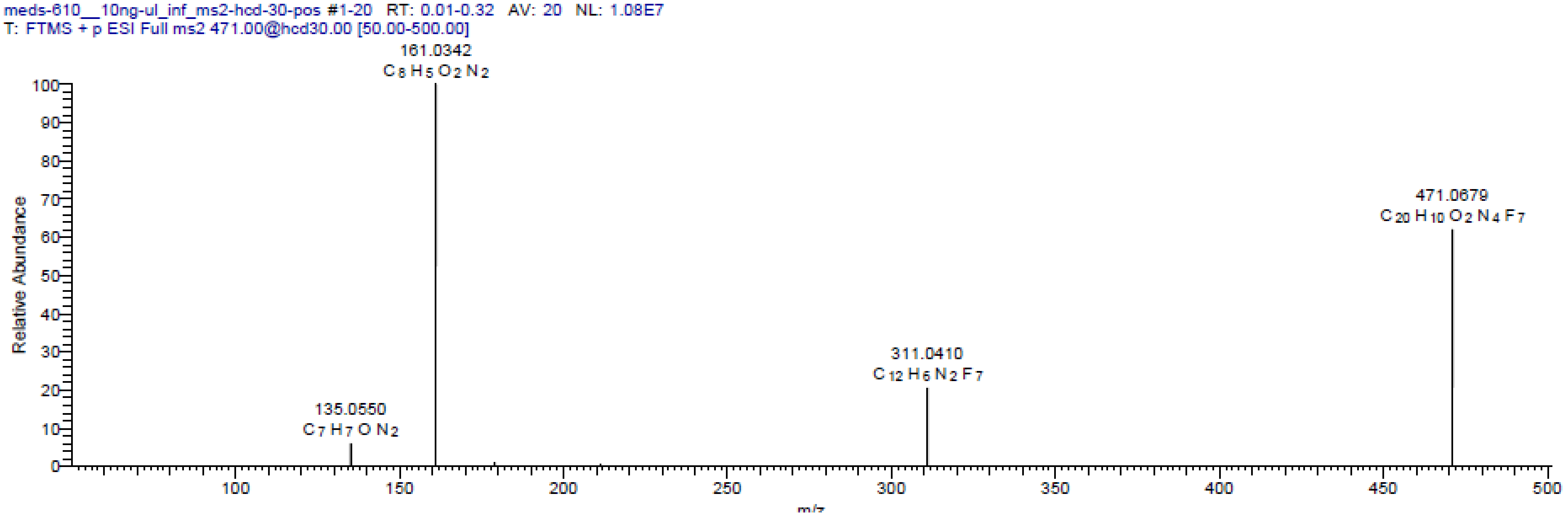

Report UHPLC

Acquisition Date/Time	7/9/2019 4:31:09 PM	Sample Name	SS1772D
Acquisition Method	FLUORO FEN. 1	Injection Volume	1
Instrument Name	UHPLC		

SS1772D : 262:10:360:10 : 1

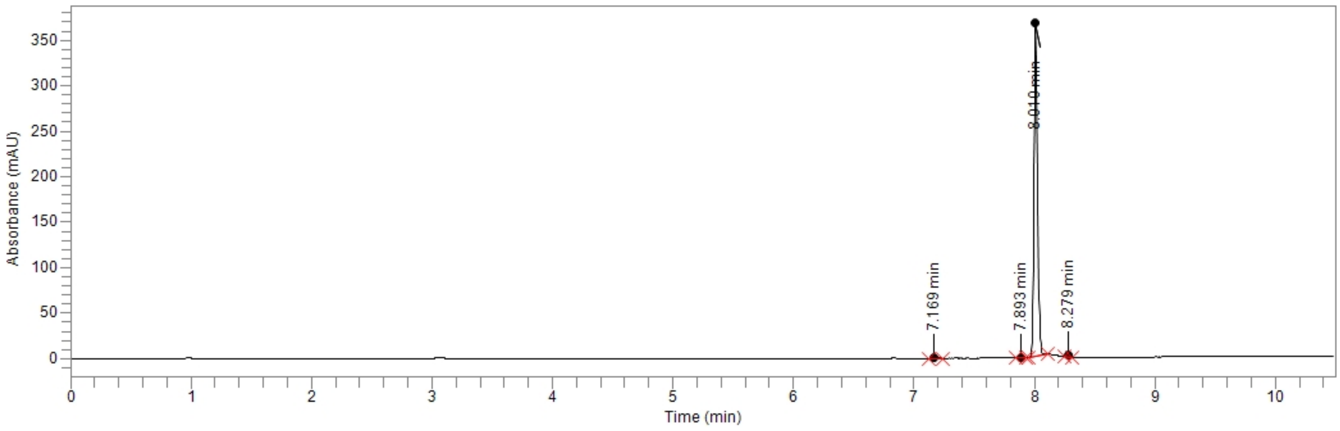

Channel Name **262:10:360:10**

Component Name	Time	Height	Area	Area %	Peak Purity	Wavelength Maximum
	7.169	808.4	2,172.3	0.28	1.94Fail	201
	7.893	577.9	1,135.8	0.15	1.97Fail	229
	8.010	365,742.4	771,705.2	99.27	1.78Fail	223
	8.279	1,305.8	2,354.3	0.30	1.69Fail	229
Total			777,367.6	100.00		

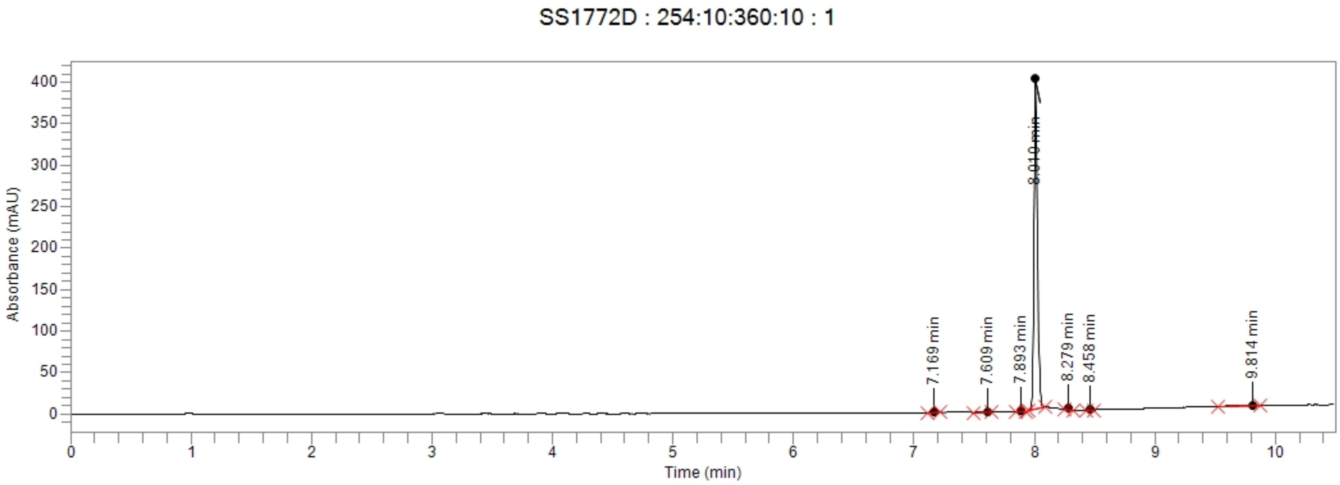

Channel Name **254:10:360:10**

Component Name	Time	Height	Area	Area %	Peak Purity	Wavelength Maximum
	7.169	754.3	1,830.4	0.22	2.32Fail	201
	7.609	221.1	1,761.8	0.21	1.59Fail	228
	7.893	421.0	781.0	0.09	2.11Fail	229
	8.010	398,285.6	837,287.6	98.66	1.78Fail	223
	8.279	1,565.1	2,827.7	0.33	1.69Fail	229
	8.458	414.3	788.4	0.09	1.14Pass	229
	9.814	189.8	3,348.5	0.39	1.26Pass	230
Total			848,625.3	100.00		

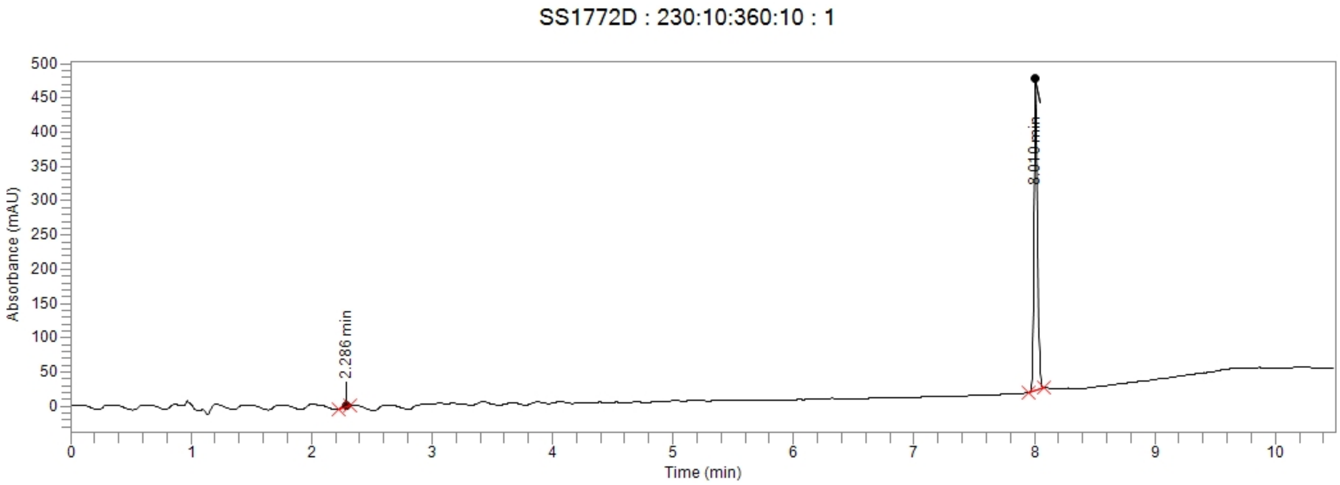

Channel Name **230:10:360:10**

Component Name	Time	Height	Area	Area %	Peak Purity	Wavelength Maximum
	2.286	2,537.5	9,094.6	0.94	3.11Fail	201
	8.010	455,065.7	957,742.8	99.06	1.78Fail	223
Total			966,837.4	100.00		

2-Hydroxy-N-(2,3,5,6-tetrafluoro-3'-hydroxy-[1,1'-biphenyl]-4-yl)pyrazolo[1,5-a]pyridine-3-carboxamide (**14**)

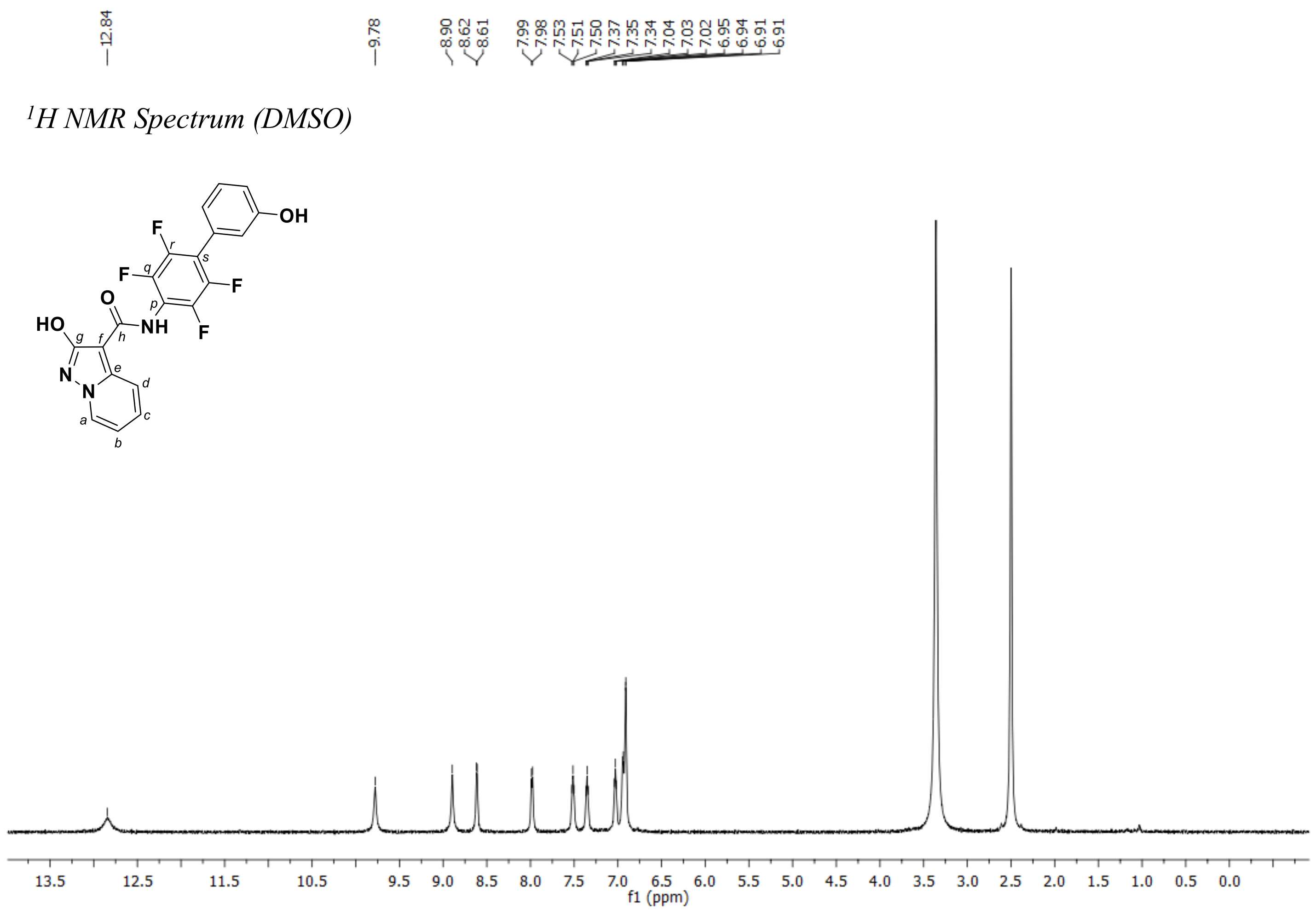

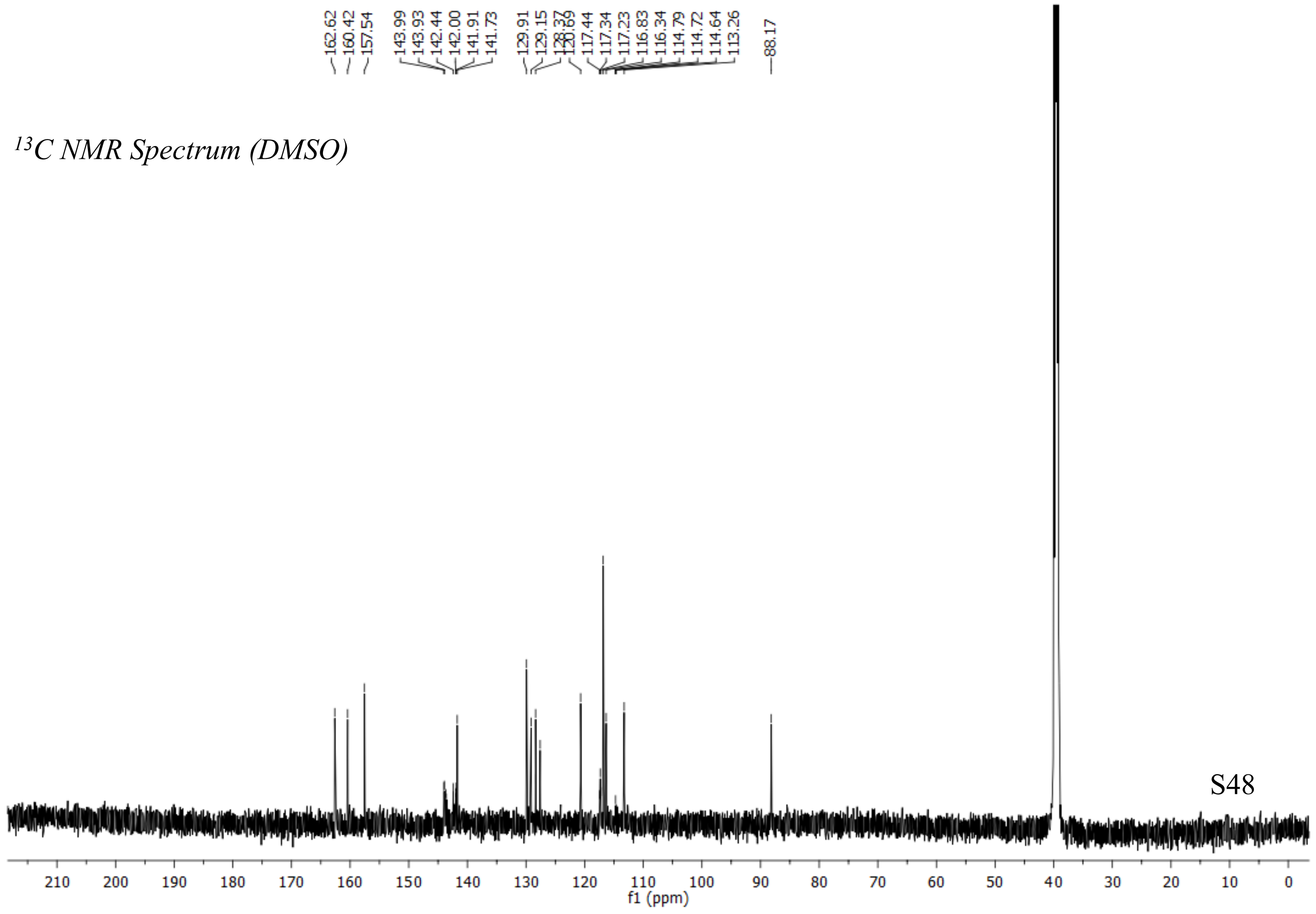

2-Hydroxy-N-(2,3,5,6-tetrafluoro-3'-hydroxy-[1,1'-biphenyl]-4-yl)pyrazolo[1,5-a]pyridine-3-carboxamide (**14**)

meds-614__10ng-ul_inf_ms-pos 01/23/20 14:17:23 MEDS-614__10ng-ul_inf_MS-pos

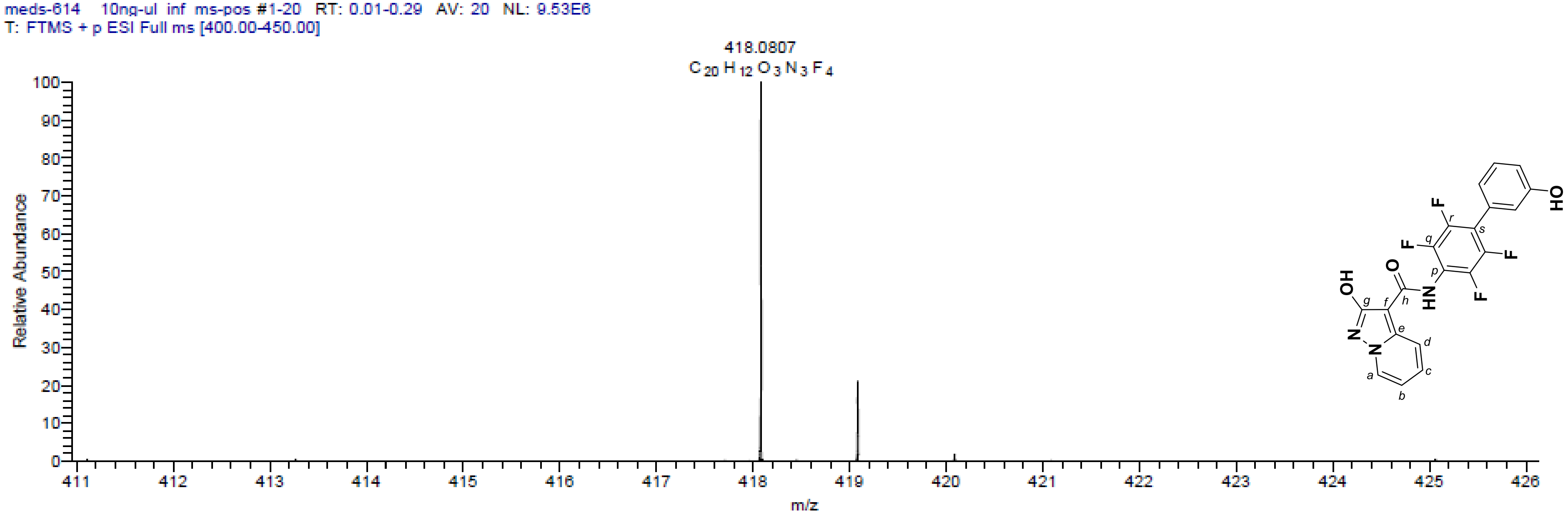

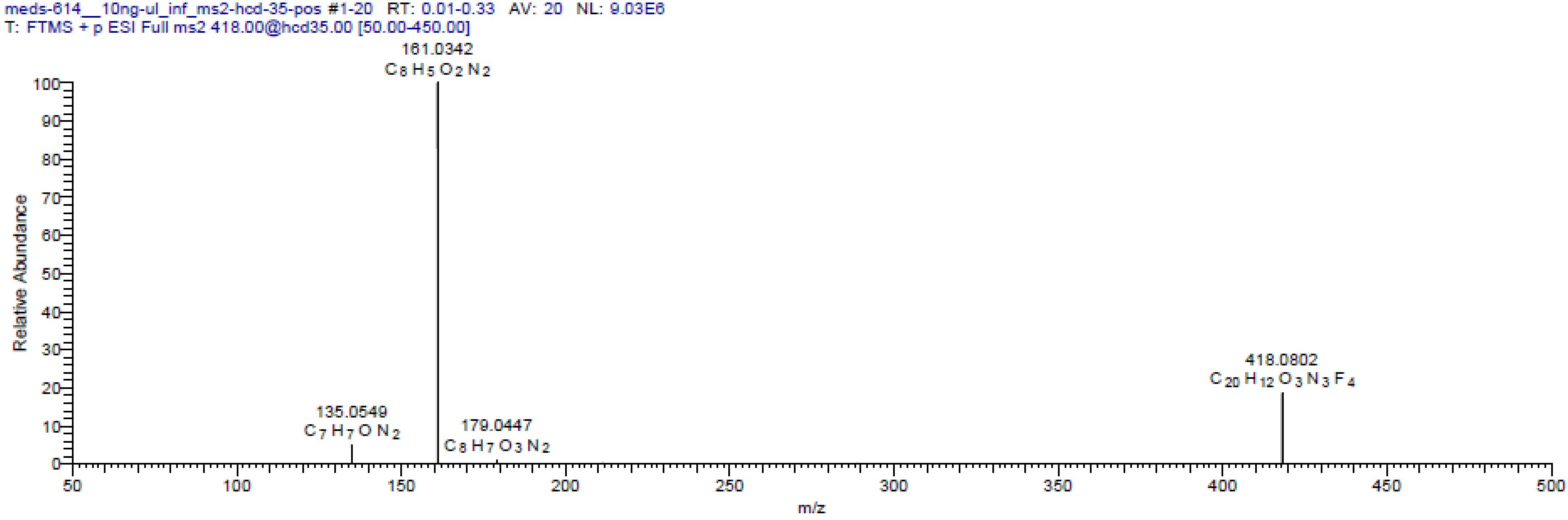

Report UHPLC

Acquisition Date/Time	7/25/2019 5:44:59 PM	Sample Name	MMS382
Acquisition Method	FLUO	Injection Volume	1
Instrument Name	UHPLC		

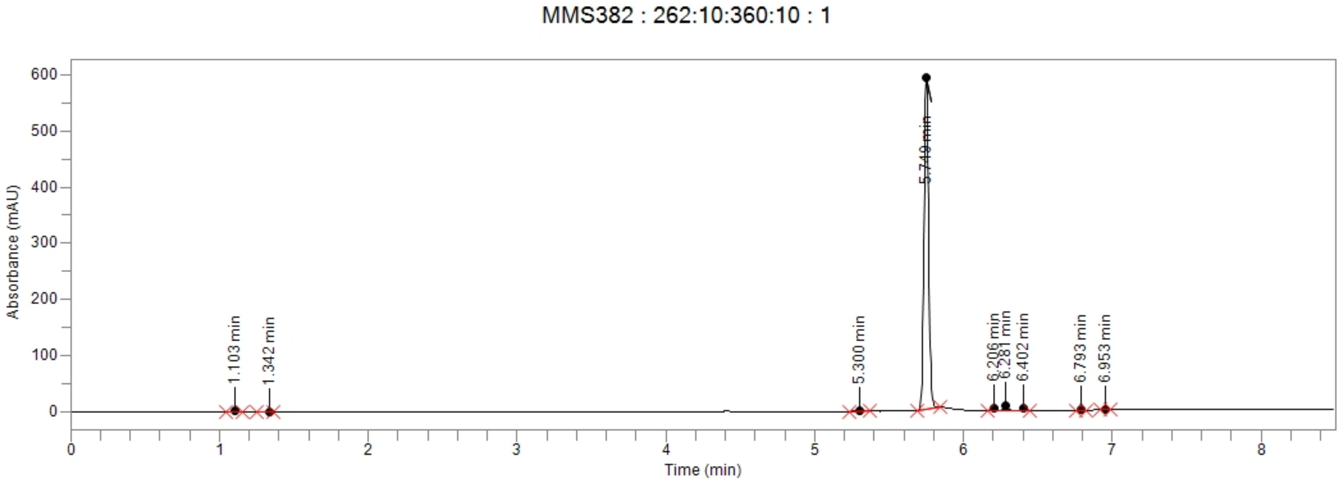

Channel Name **262:10:360:10**

Component Name	Time	Height	Area	Area %	Peak Purity	Wavelength Maximum
	1.103	1,101.7	3,750.0	0.28	Not Enough Valid Points	201
	1.342	330.8	2,019.3	0.15	8.69Fail	200
	5.300	1,695.0	6,489.9	0.49	4.05Fail	201
	5.749	593,508.1	1,276,458.0	95.95	1.23Pass	222
	6.206	5,669.2	11,481.5	0.86	1.29Pass	227
	6.281	9,029.7	20,079.7	1.51	1.49Pass	225
	6.402	3,663.9	7,251.7	0.55	1.42Pass	229
	6.793	426.2	959.7	0.07	1.25Pass	230
	6.953	814.0	1,833.6	0.14	1.18Pass	230
Total			1,330,323.5	100.00		

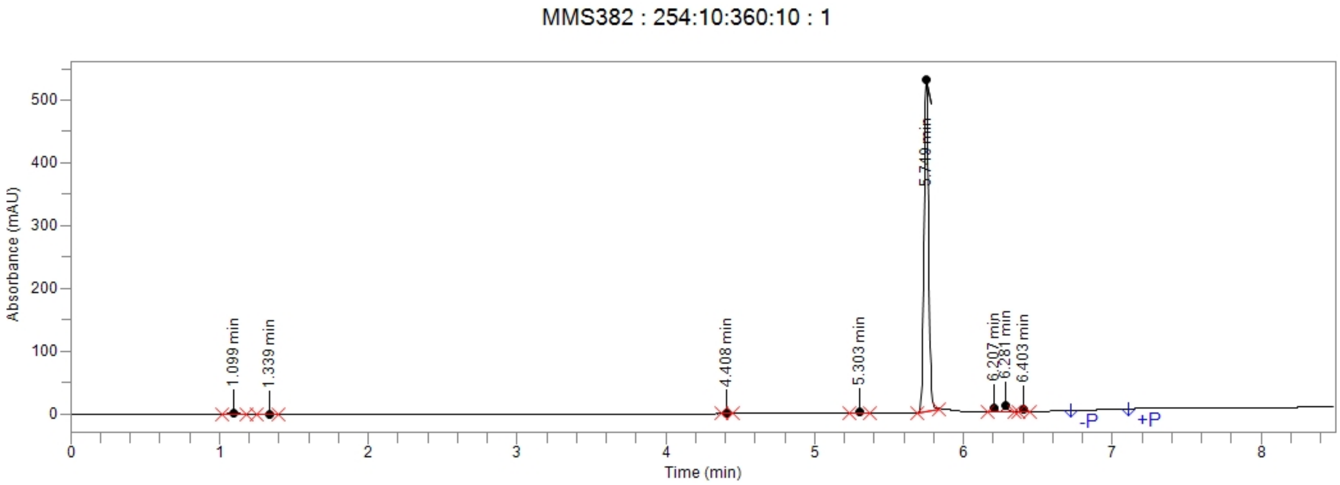

Channel Name **254:10:360:10**

Component Name	Time	Height	Area	Area %	Peak Purity	Wavelength Maximum
	1.099	1,498.1	6,766.6	0.57	Not Enough Valid Points	201
	1.339	583.6	3,224.2	0.27	2.66Fail	200
	4.408	563.1	1,468.6	0.12	1.39Pass	201
	5.303	2,101.0	7,841.8	0.66	3.55Fail	201
	5.749	530,130.3	1,137,447.6	95.33	1.23Pass	222
	6.207	5,260.6	10,404.6	0.87	1.43Pass	227
	6.281	9,623.0	20,123.3	1.69	1.34Pass	225
	6.403	3,095.2	5,837.4	0.49	1.42Pass	229
Total			1,193,114.1	100.00		

MMS382 : 230:10:360:10 : 1

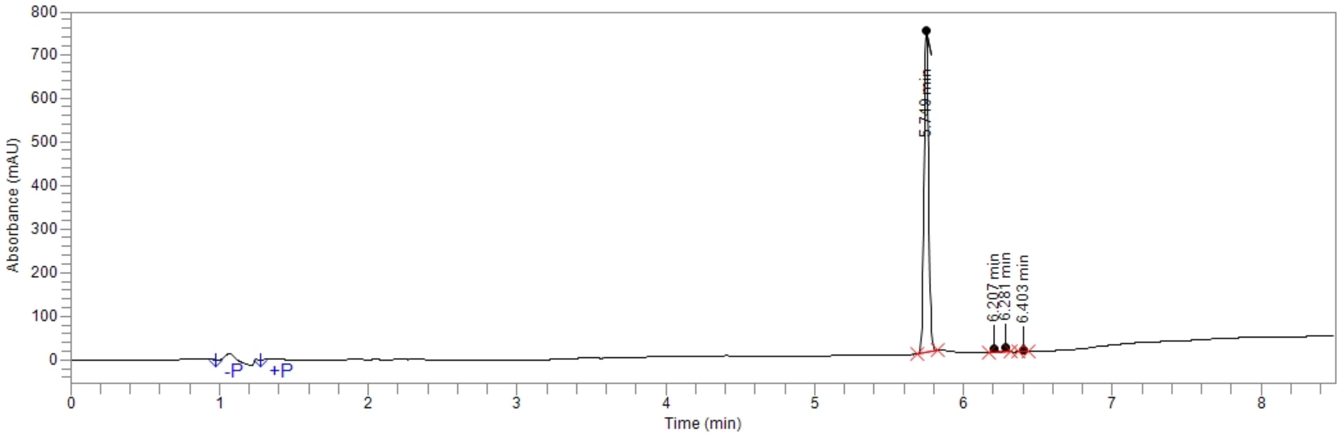

Channel Name **230:10:360:10**

Component Name	Time	Height	Area	Area %	Peak Purity	Wavelength Maximum
	5.749	741,009.4	1,591,497.4	97.19	1.23Pass	222
	6.207	9,810.5	19,061.9	1.16	1.28Pass	227
	6.281	9,209.5	17,745.5	1.08	1.52Fail	225
	6.403	5,062.4	9,180.2	0.56	1.54Fail	229
Total			1,637,484.9	100.00		

2-Hydroxy-N-(2,3,5,6-tetrafluoro-3'-(trifluoromethoxy)-[1,1'-biphenyl]-4-yl)pyrazolo[1,5-a]pyridine-3-carboxamide (**15**)

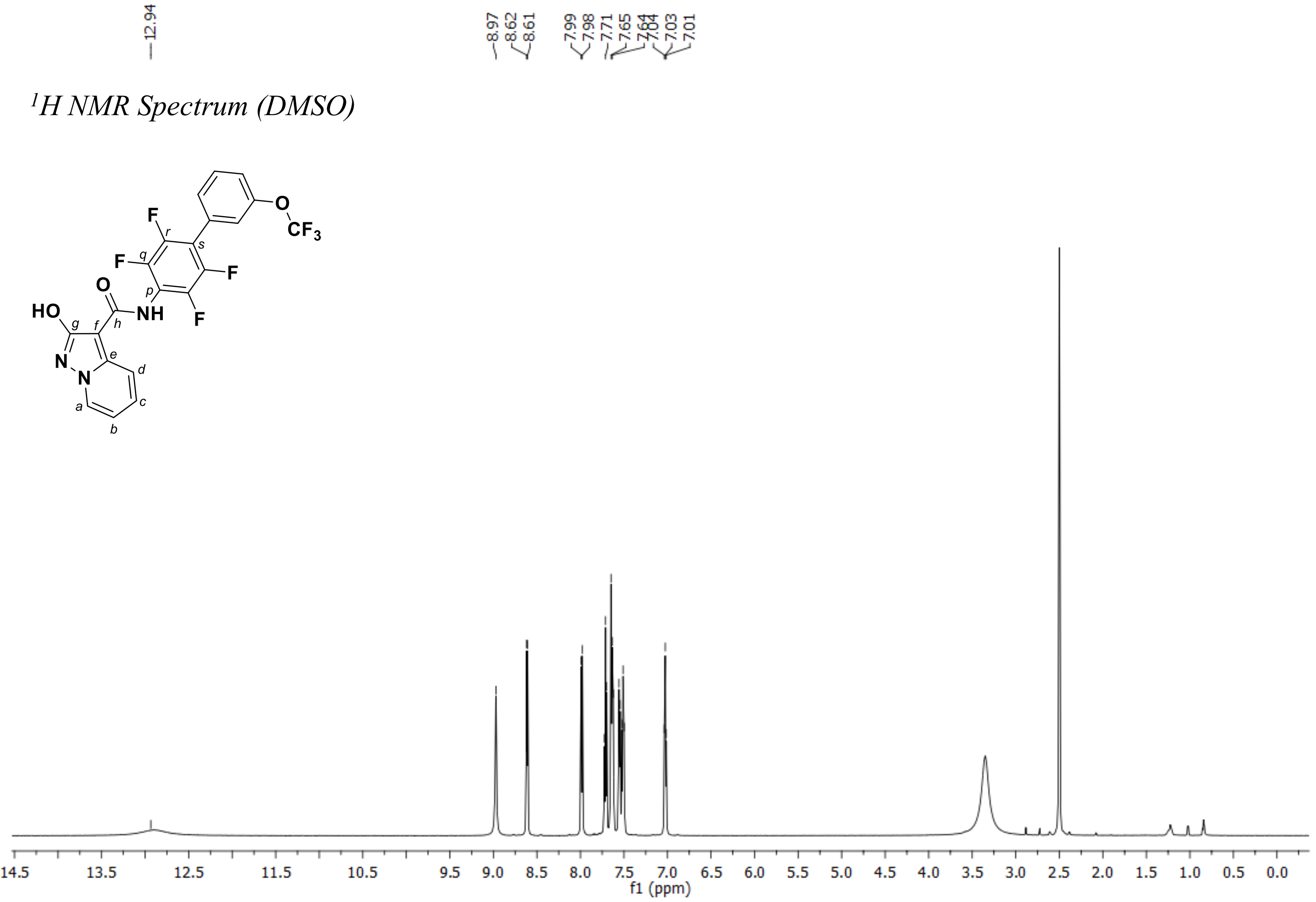

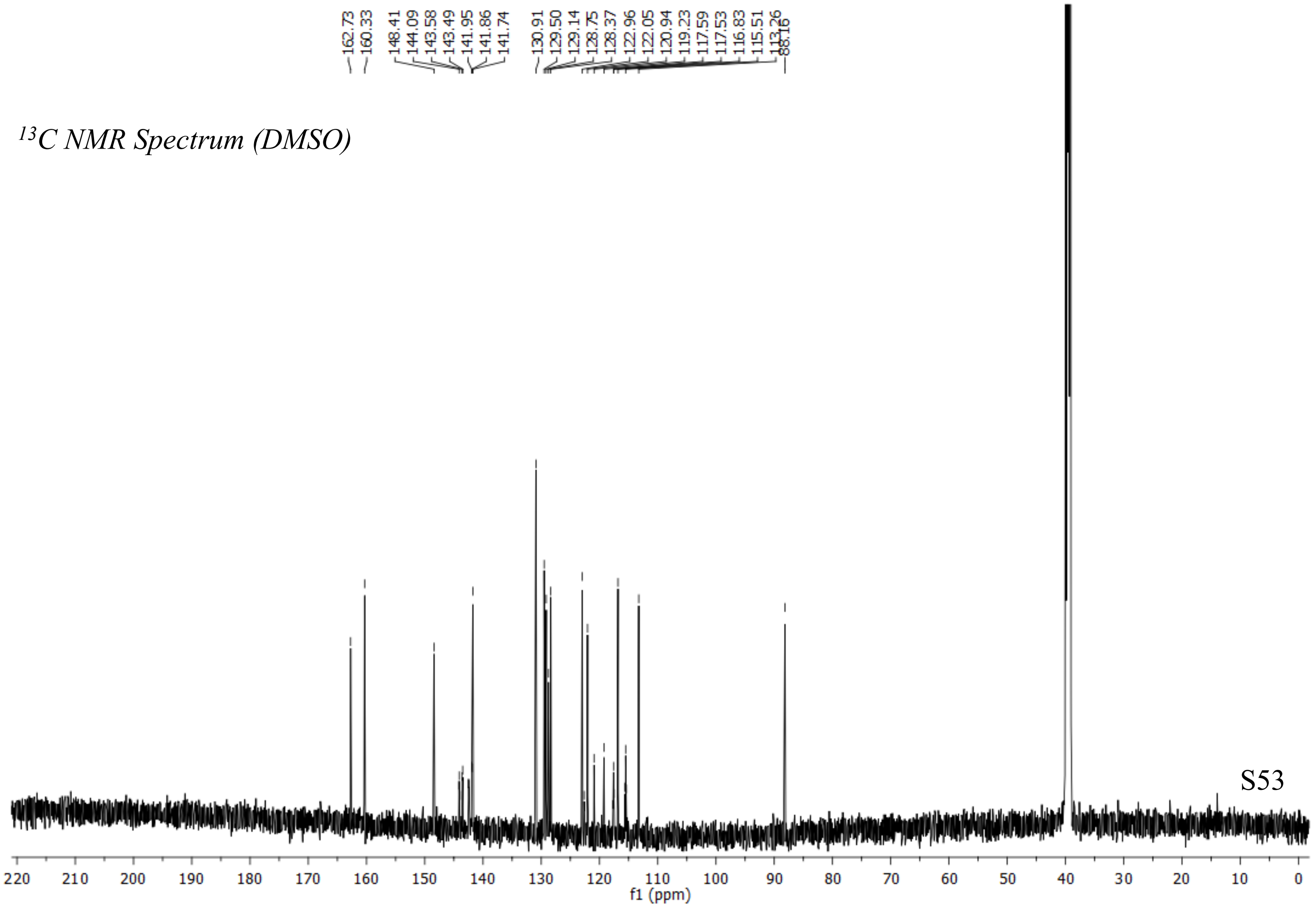

2-Hydroxy-N-(2,3,5,6-tetrafluoro-3'-(trifluoromethoxy)-[1,1'-biphenyl]-4-yl)pyrazolo[1,5-a]pyridine-3-carboxamide (**15**)

meds-608__10ng-ul_inf_ms2-hcd-35-pos 01/23/20 13:18:23 MEDS-608__10ng-ul_inf_MS2-HCD-35-pos

meds-608 10ng-ul inf ms-pos #1-20 RT: 0.01-0.29 AV: 20 NL: 2.00E6
T: FTMS + p ESI Full ms [450.00-500.00]

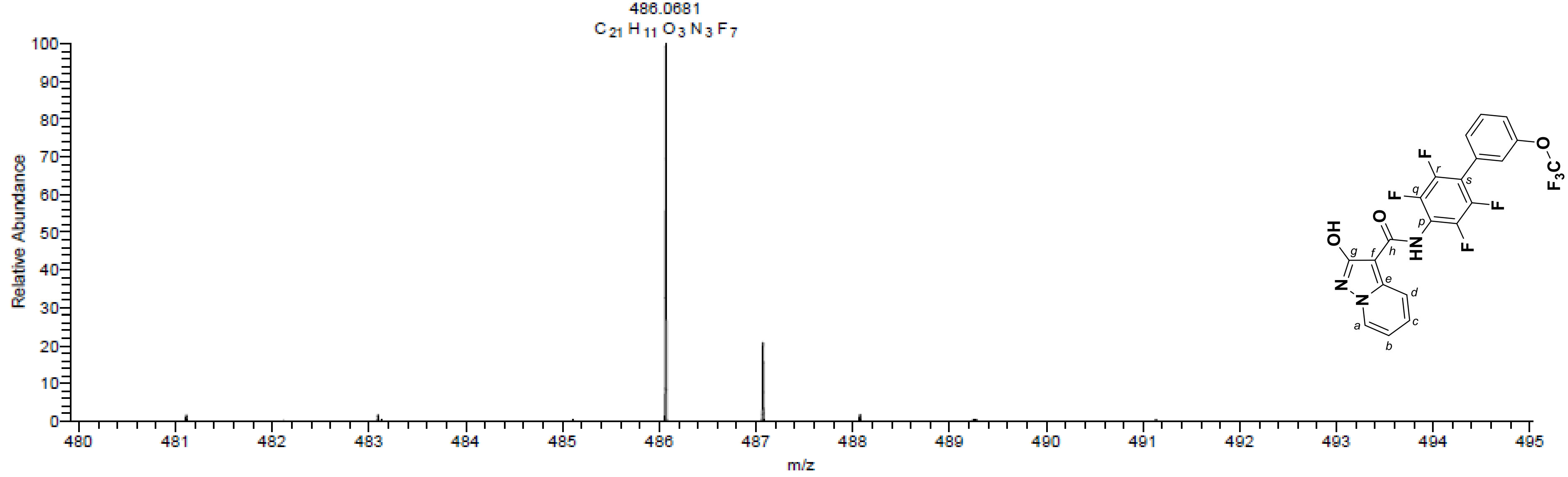

meds-608__10ng-ul_inf_ms2-hcd-35-pos #1-20 RT: 0.01-0.39 AV: 20 NL: 8.76E5
T: FTMS + p ESI Full ms2 486.00@hcd35.00 [50.00-500.00]

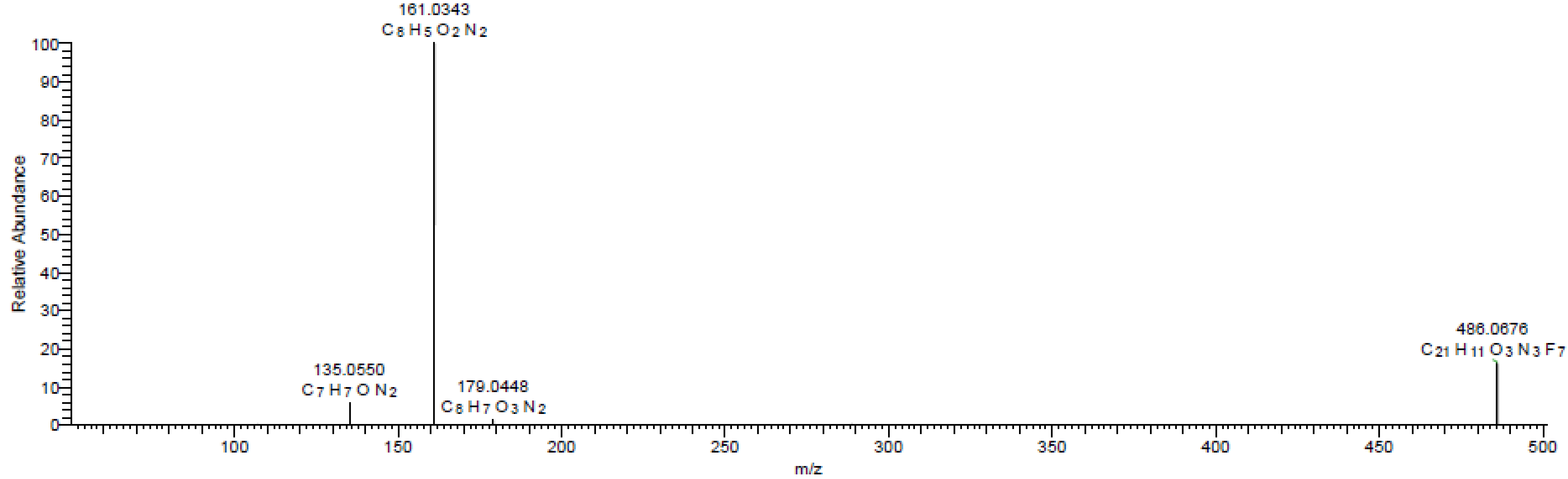

Report UHPLC

Acquisition Date/Time	6/12/2019 9:58:04 AM	Sample Name	MMS318
Acquisition Method	DHODH screening	Injection Volume	2
Instrument Name	UHPLC		

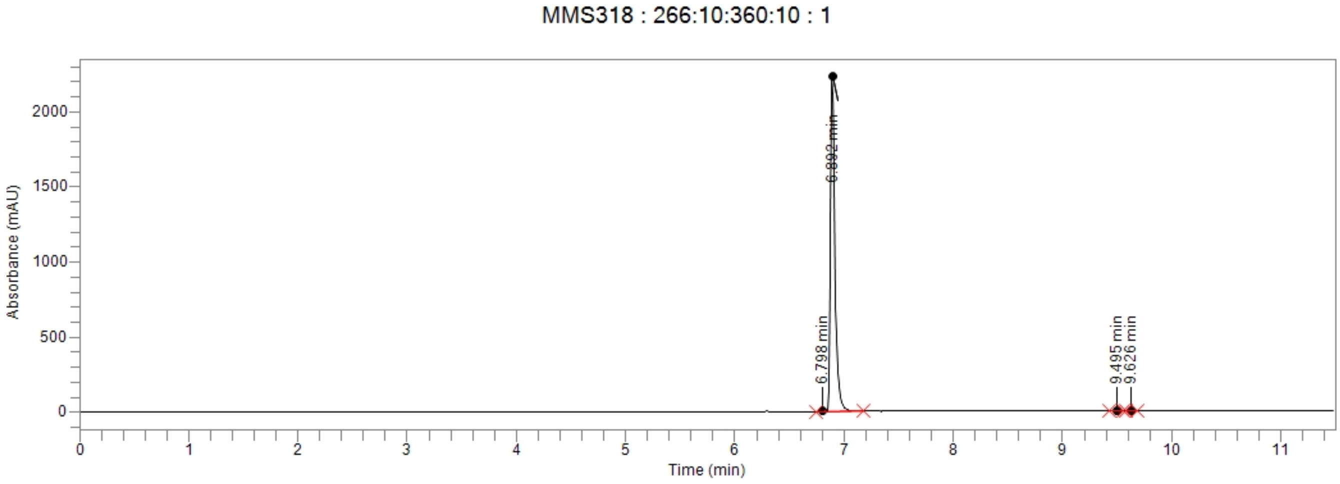

Channel Name **266:10:360:10**

Component Name	Time	Height	Area	Area %	Peak Purity	Wavelength Maximum
	6.798	1,209.1	2,911.1	0.05	1.13Pass	225
	6.892	2,249,915.8	6,259,179.3	99.80	1.15Pass	225
	9.495	1,655.5	6,194.2	0.10	1.04Pass	228
	9.626	1,294.6	3,154.1	0.05	1.05Pass	228
Total			6,271,438.7	100.00		

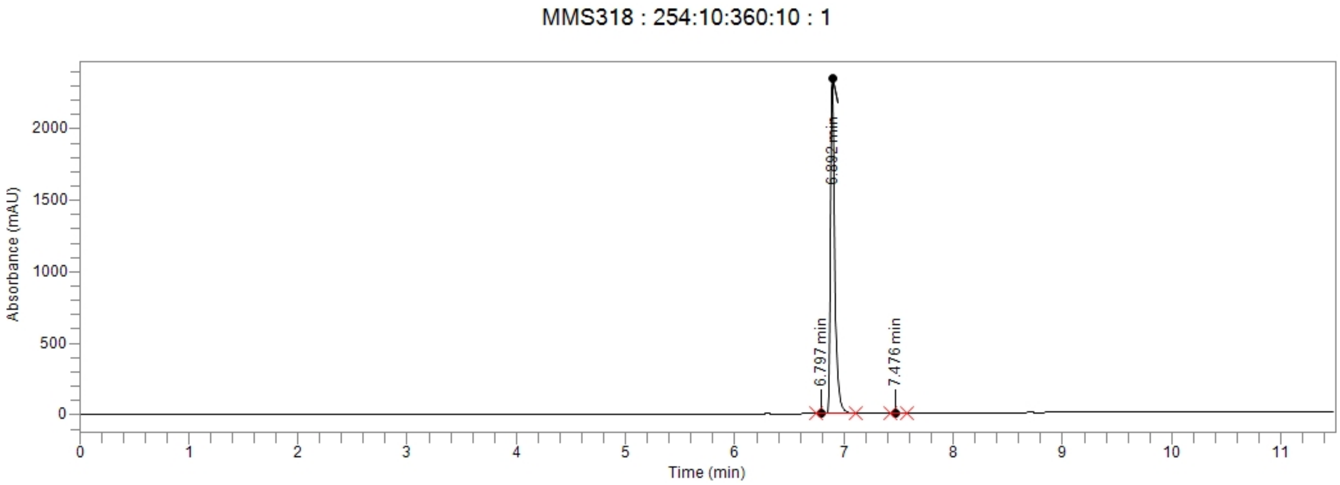

Channel Name **254:10:360:10**

Component Name	Time	Height	Area	Area %	Peak Purity	Wavelength Maximum
	6.797	976.6	2,088.7	0.03	1.13Pass	225
	6.892	2,357,660.2	6,245,168.8	99.80	1.15Pass	225
	7.476	3,405.9	10,355.7	0.17	1.11Pass	228
Total			6,257,613.2	100.00		

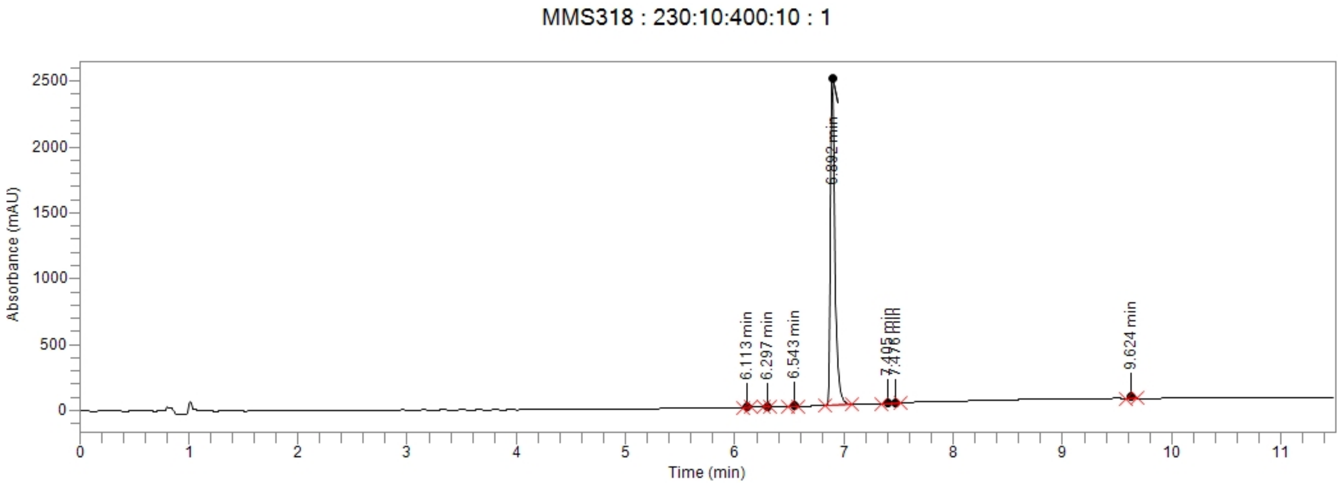

Channel Name **230:10:400:10**

Component Name	Time	Height	Area	Area %	Peak Purity	Wavelength Maximum
	6.113	1,983.2	3,761.9	0.05	10.21Fail	200
	6.297	1,994.6	3,959.9	0.06	3.38Fail	224
	6.543	4,070.3	11,828.1	0.17	2.30Fail	225
	6.892	2,501,378.2	7,002,826.7	99.00	1.15Pass	225
	7.405	3,316.5	9,726.1	0.14	1.11Pass	227
	7.476	6,347.1	15,040.4	0.21	1.12Pass	228
	9.624	10,670.3	26,625.7	0.38	1.05Pass	228
Total			7,073,768.8	100.00		

2-Hydroxy-N-(2,3,5,6-tetrafluoro-3'-methoxy-[1,1'-biphenyl]-4-yl)pyrazolo[1,5-a]pyridine-3-carboxamide (**16**)

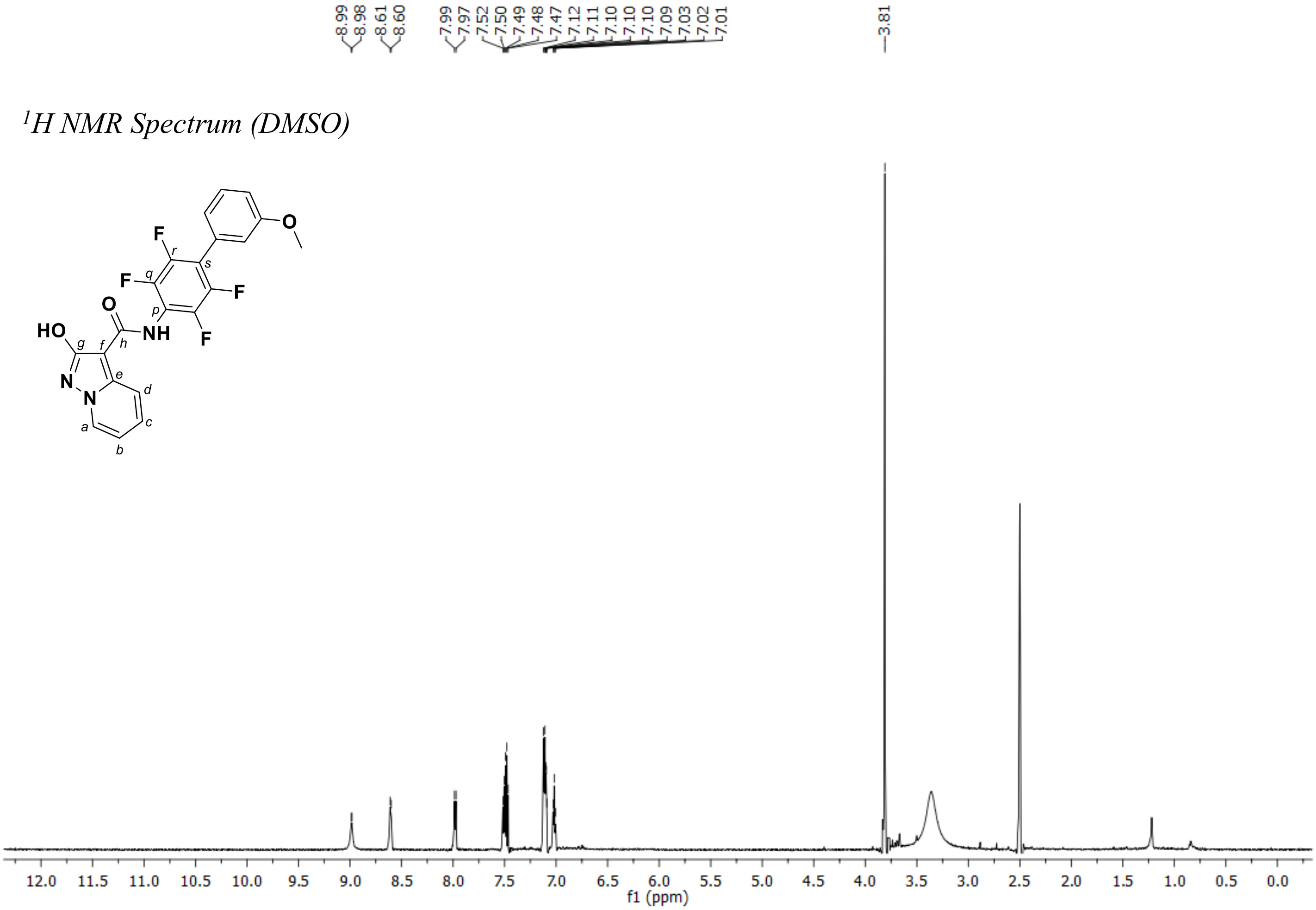

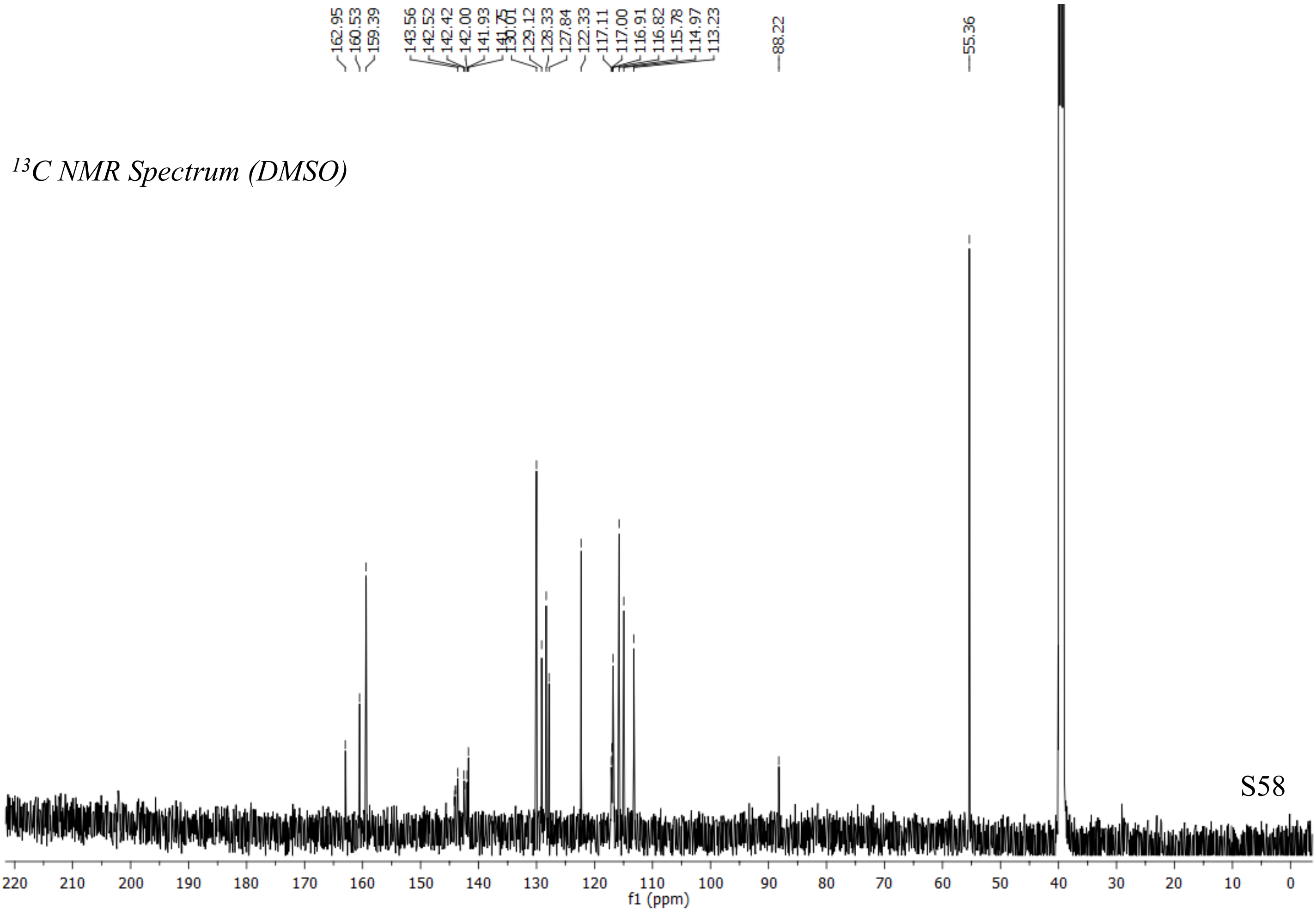

P:\Renzo\...\28__M606_10ng-uL__pos 07/15/20 05:15:31

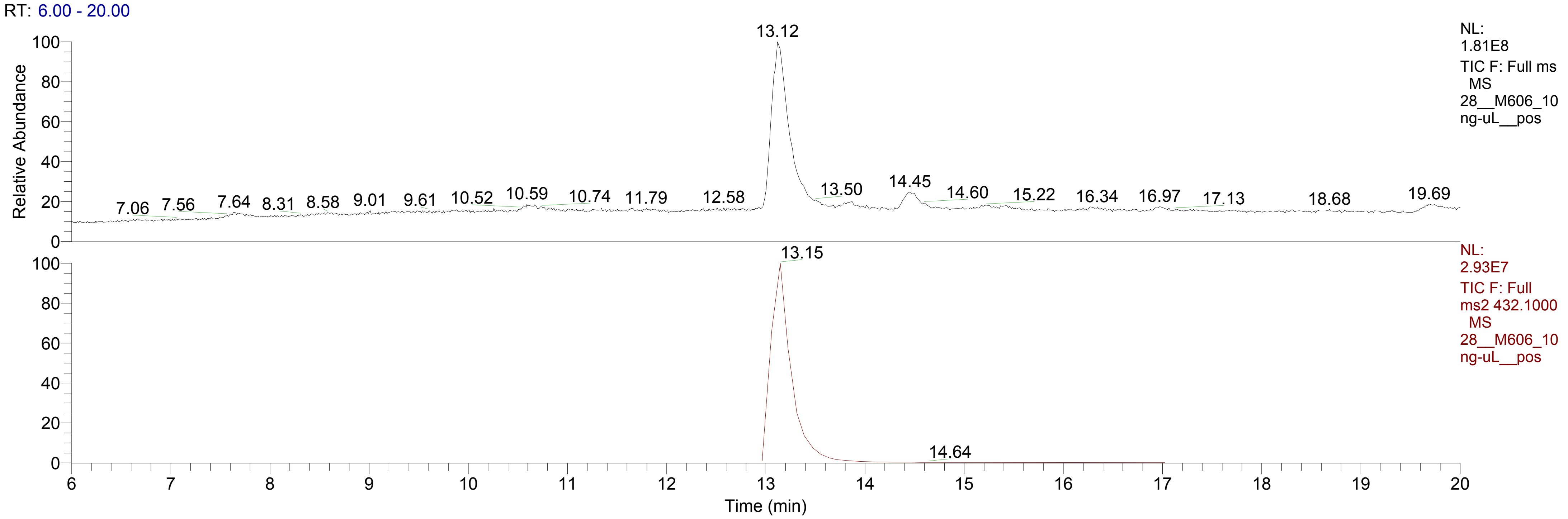

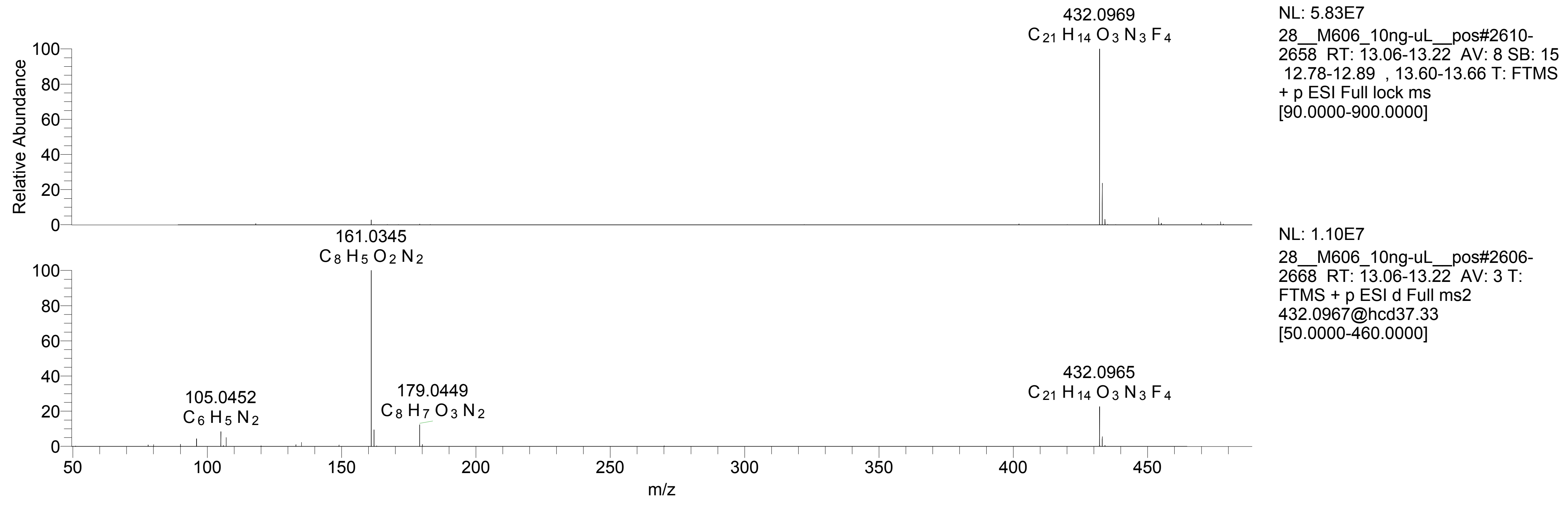

Report UHPLC

Acquisition Date/Time	4/16/2019 10:15:46 AM	Sample Name	MMS254.2
Acquisition Method	DHODH screening	Injection Volume	2
Instrument Name	UHPLC		

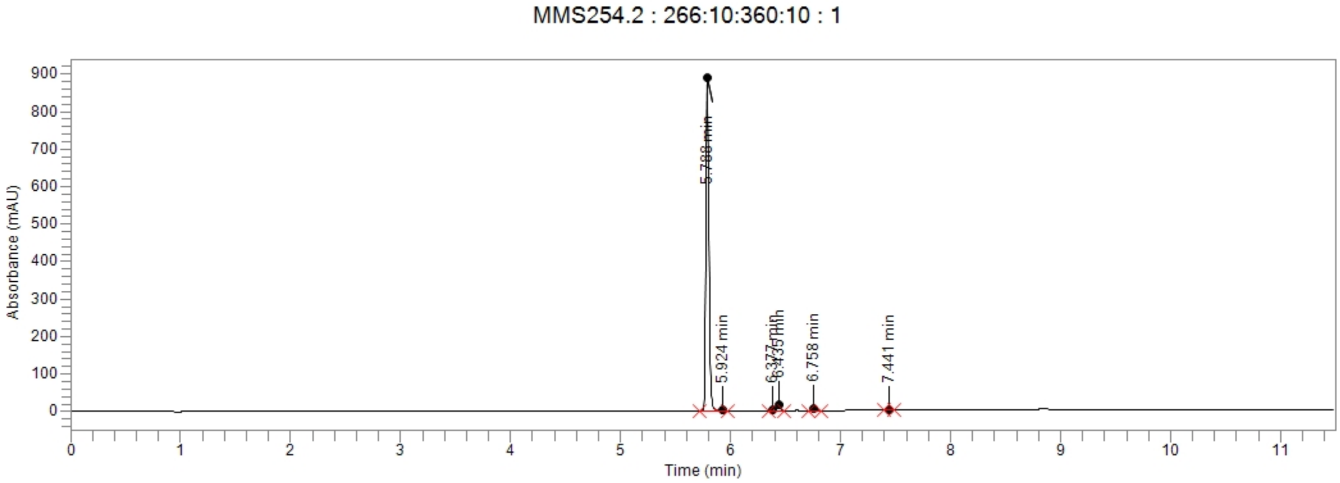

Channel Name **266:10:360:10**

Component Name	Time	Height	Area	Area %	Peak Purity	Wavelength Maximum
	5.788	894,699.6	1,700,575.6	96.86	1.20Pass	222
	5.924	1,605.9	6,107.6	0.35	2.06Fail	224
	6.377	728.7	1,300.7	0.07	1.09Pass	227
	6.435	15,365.3	29,733.9	1.69	1.36Pass	224
	6.758	6,651.5	12,768.7	0.73	1.09Pass	225
	7.441	2,860.2	5,190.0	0.30	1.11Pass	227
Total			1,755,676.4	100.00		

MMS254.2 : 254:10:360:10 : 1

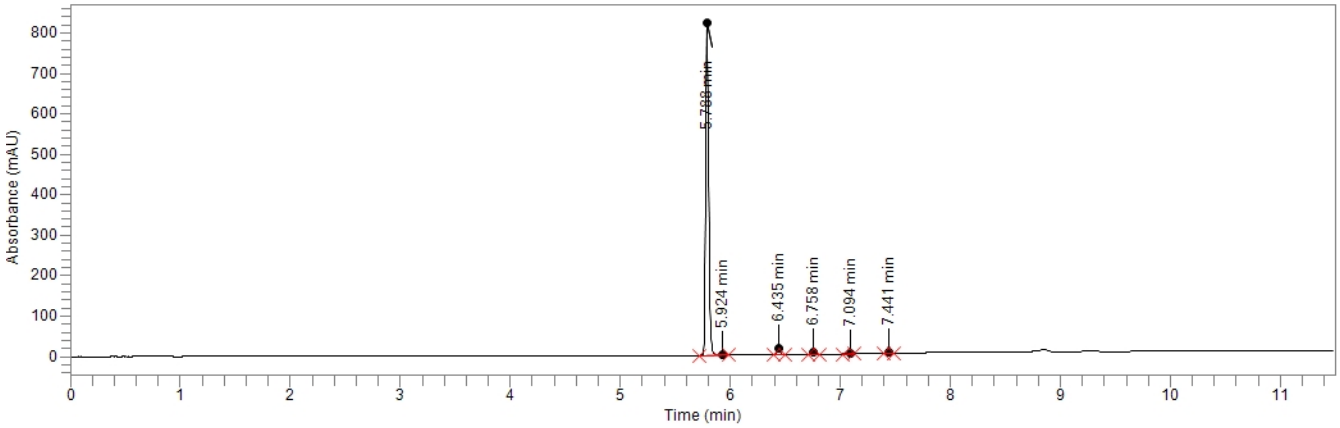

Channel Name **254:10:360:10**

Component Name	Time	Height	Area	Area %	Peak Purity	Wavelength Maximum
	5.788	827,536.5	1,570,958.1	96.73	1.20Pass	222
	5.924	1,883.8	6,834.7	0.42	3.75Fail	224
	6.435	15,750.6	29,840.8	1.84	1.10Pass	224
	6.758	5,578.2	10,477.9	0.65	1.09Pass	225
	7.094	524.8	1,674.6	0.10	1.07Pass	227
	7.441	2,386.6	4,227.8	0.26	1.11Pass	227
Total			1,624,013.9	100.00		

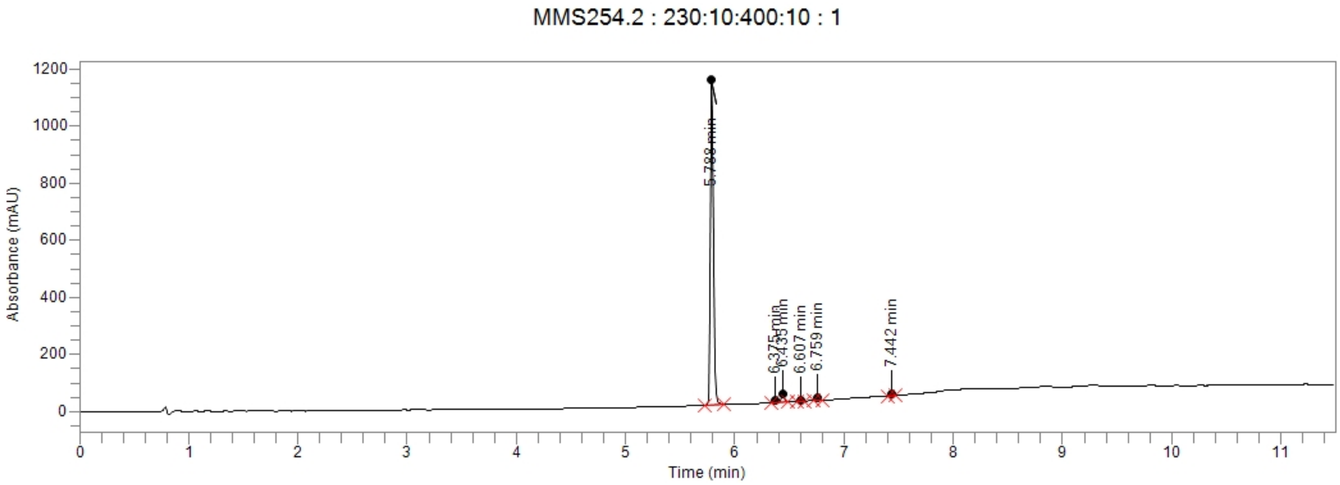

Channel Name **230:10:400:10**

Component Name	Time	Height	Area	Area %	Peak Purity	Wavelength Maximum
	5.788	1,148,224.4	2,184,838.3	95.41	1.20Pass	222
	6.375	7,259.3	13,689.9	0.60	1.25Pass	227
	6.435	30,171.7	57,345.6	2.50	1.10Pass	224
	6.607	4,791.8	9,341.6	0.41	1.04Pass	225
	6.759	9,028.5	16,477.9	0.72	1.09Pass	225
	7.442	4,634.6	8,203.2	0.36	1.11Pass	227
Total			2,289,896.6	100.00		

2-Hydroxy-N-(2,3,5,6-tetrafluoro-3'-propoxy-[1,1'-biphenyl]-4-yl)pyrazolo[1,5-a]pyridine-3-carboxamide **(17)**

^{1}H NMR Spectrum (DMSO)

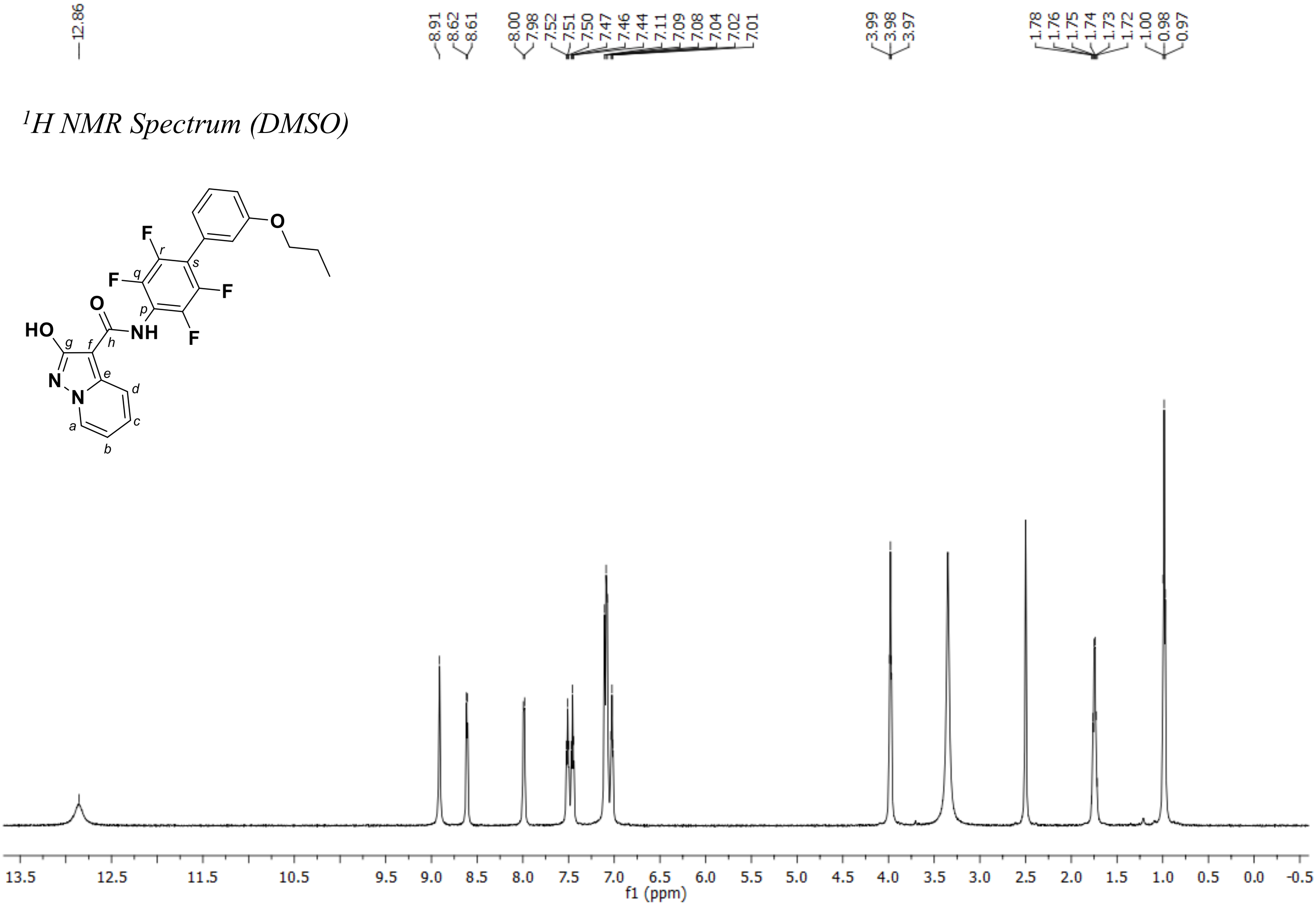

^{13}C NMR Spectrum (DMSO)

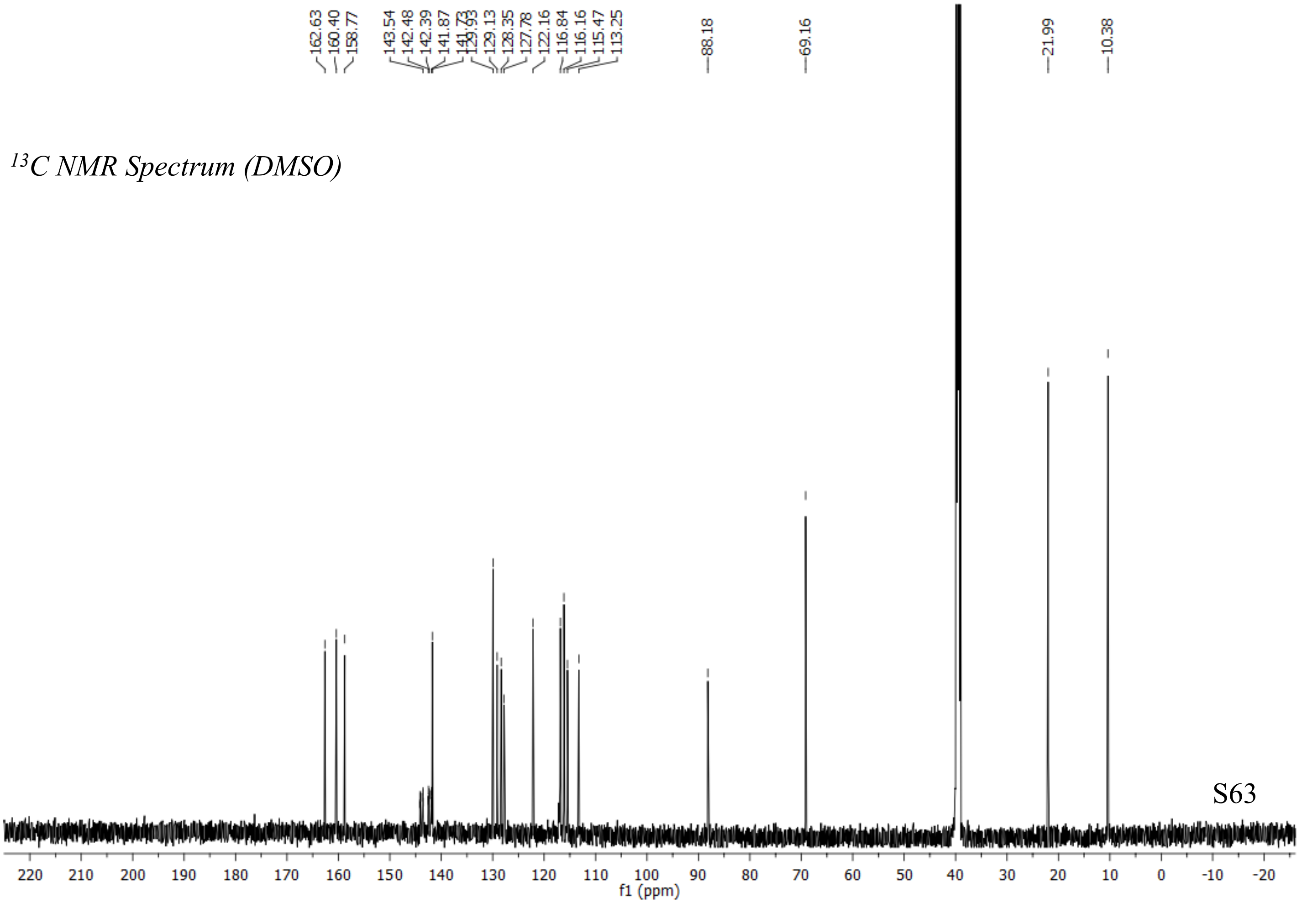

2-Hydroxy-N-(2,3,5,6-tetrafluoro-3'-propoxy-[1,1'-biphenyl]-4-yl)pyrazolo[1,5-a]pyridine-3-carboxamide (**17**)

meds-613__10ng-ul_inf_ms-pos 01/23/20 14:06:55 MEDS-613__10ng-ul_inf_MS-pos

meds-613 10ng-ul inf ms-pos #1-20 RT: 0.00-0.29 AV: 20 NL: 4.51E6
T: FTMS + p ESI Full ms [450.00-470.00]

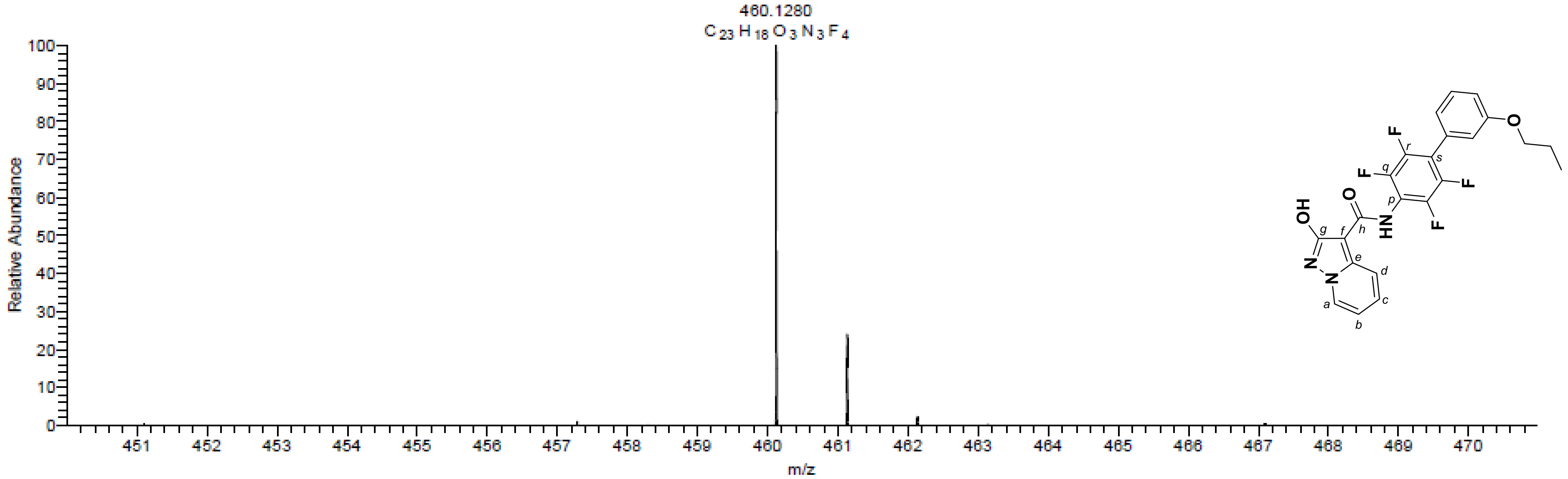

meds-613__10ng-ul_inf_ms2-hcd-37-pos #1-20 RT: 0.01-0.35 AV: 20 NL: 2.78E6
T: FTMS + p ESI Full ms2 460.00@hcd37.00 [50.00-500.00]

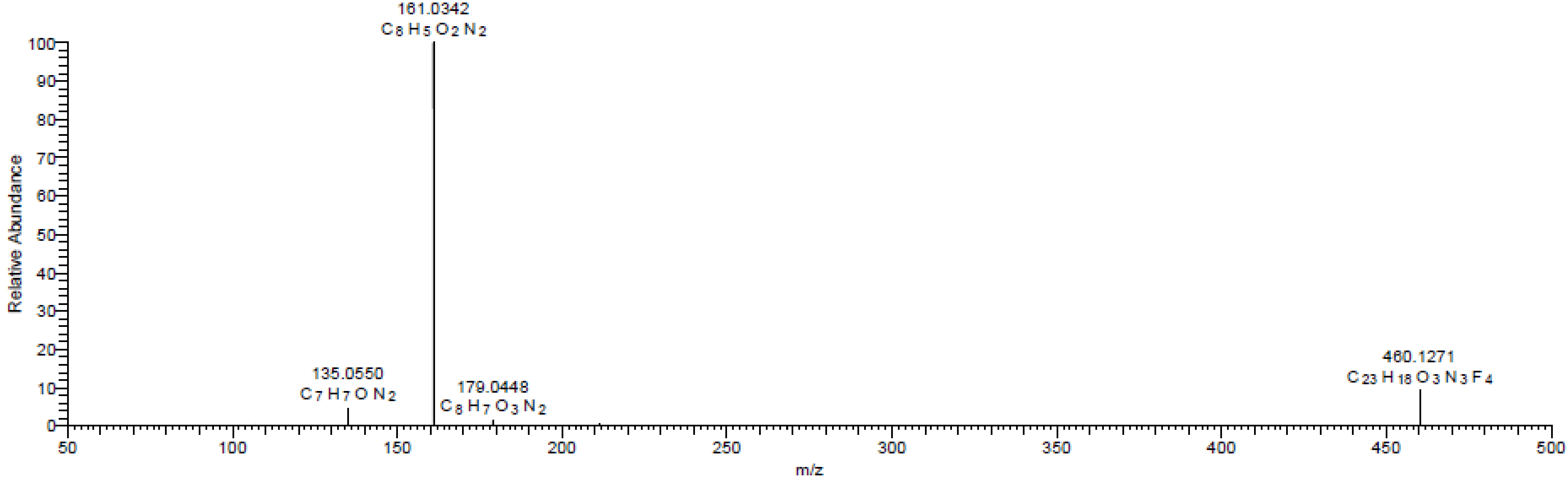

Report UHPLC

Acquisition Date/Time	7/24/2019 3:12:55 PM	Sample Name	MMS376C
Acquisition Method	FLUO	Injection Volume	1
Instrument Name	UHPLC		

MMS376C : 262:10:360:10 : 1

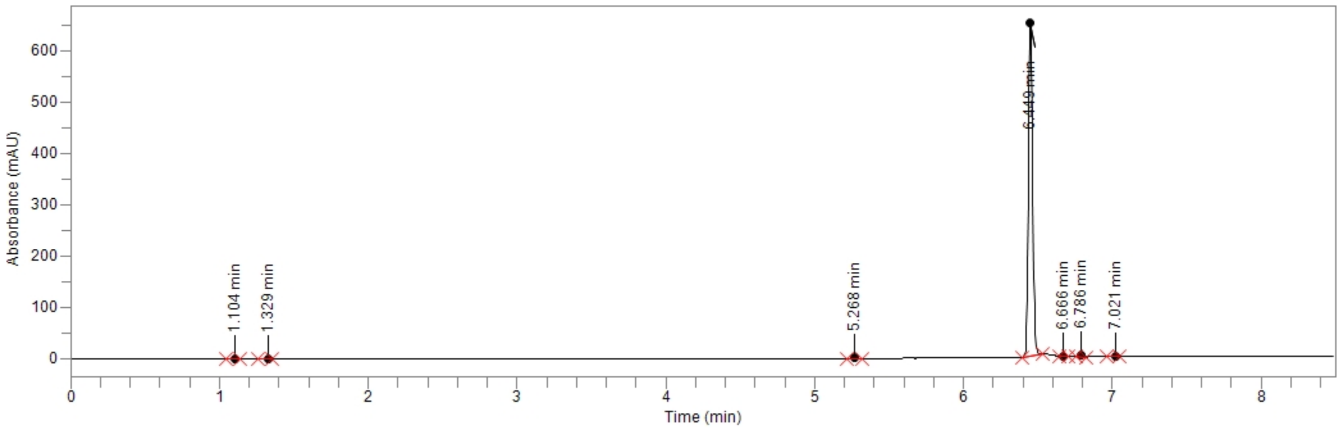

Channel Name **262:10:360:10**

Component Name	Time	Height	Area	Area %	Peak Purity	Wavelength Maximum
	1.104	693.0	2,287.2	0.17	Not Enough Valid Points	201
	1.329	296.2	1,558.9	0.12	Not Enough Valid Points	202
	5.268	1,139.9	2,920.7	0.22	3.98Fail	202
	6.449	652,901.4	1,302,165.2	98.94	2.10Fail	223
	6.666	895.9	1,532.3	0.12	1.33Pass	230
	6.786	2,725.2	4,691.0	0.36	1.21Pass	230
	7.021	495.8	1,001.3	0.08	1.09Pass	231
Total			1,316,156.5	100.00		

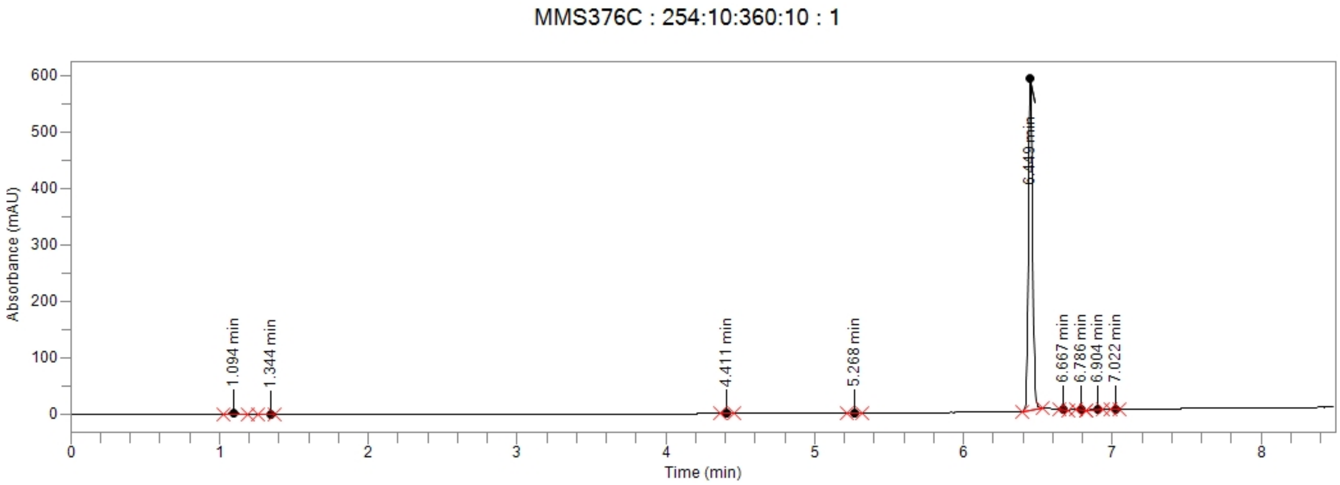

Channel Name **254:10:360:10**

Component Name	Time	Height	Area	Area %	Peak Purity	Wavelength Maximum
	1.094	1,598.0	7,525.7	0.63	Not Enough Valid Points	201
	1.344	302.9	2,360.7	0.20	Not Enough Valid Points	202
	4.411	679.4	2,023.6	0.17	2.65Fail	201
	5.268	1,133.6	2,896.6	0.24	2.54Fail	202
	6.449	589,951.2	1,174,858.4	98.03	2.10Fail	223
	6.667	913.8	1,555.3	0.13	1.33Pass	230
	6.786	2,190.4	3,587.5	0.30	1.21Pass	230
	6.904	807.1	2,822.8	0.24	1.29Pass	230
	7.022	473.2	894.5	0.07	1.16Pass	230
Total			1,198,525.0	100.00		

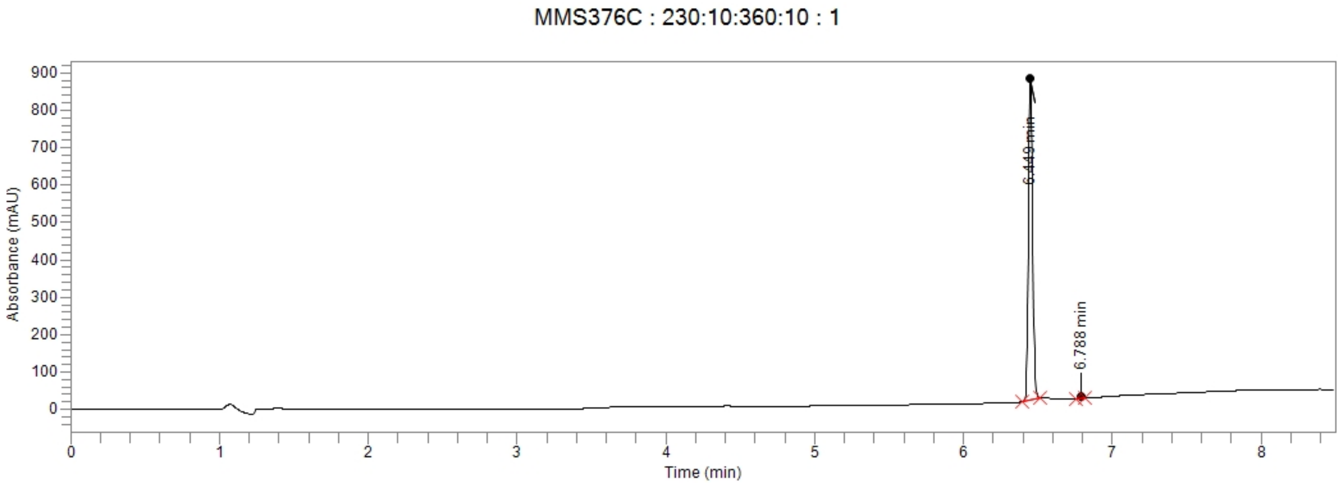

Channel Name **230:10:360:10**

Component Name	Time	Height	Area	Area %	Peak Purity	Wavelength Maximum
	6.449	864,514.0	1,725,257.1	99.59	2.10Fail	223
	6.788	4,232.3	7,153.7	0.41	1.21Pass	230
Total			1,732,410.8	100.00		

2-Hydroxy-N-(2,3,5,6-tetrafluoro-[1,1'-biphenyl]-4-yl)-4,5,6,7-tetrahydropyrazolo[1,5-a]pyridine-3-carboxamide (**5**)

^{1}H NMR Spectrum (DMSO)

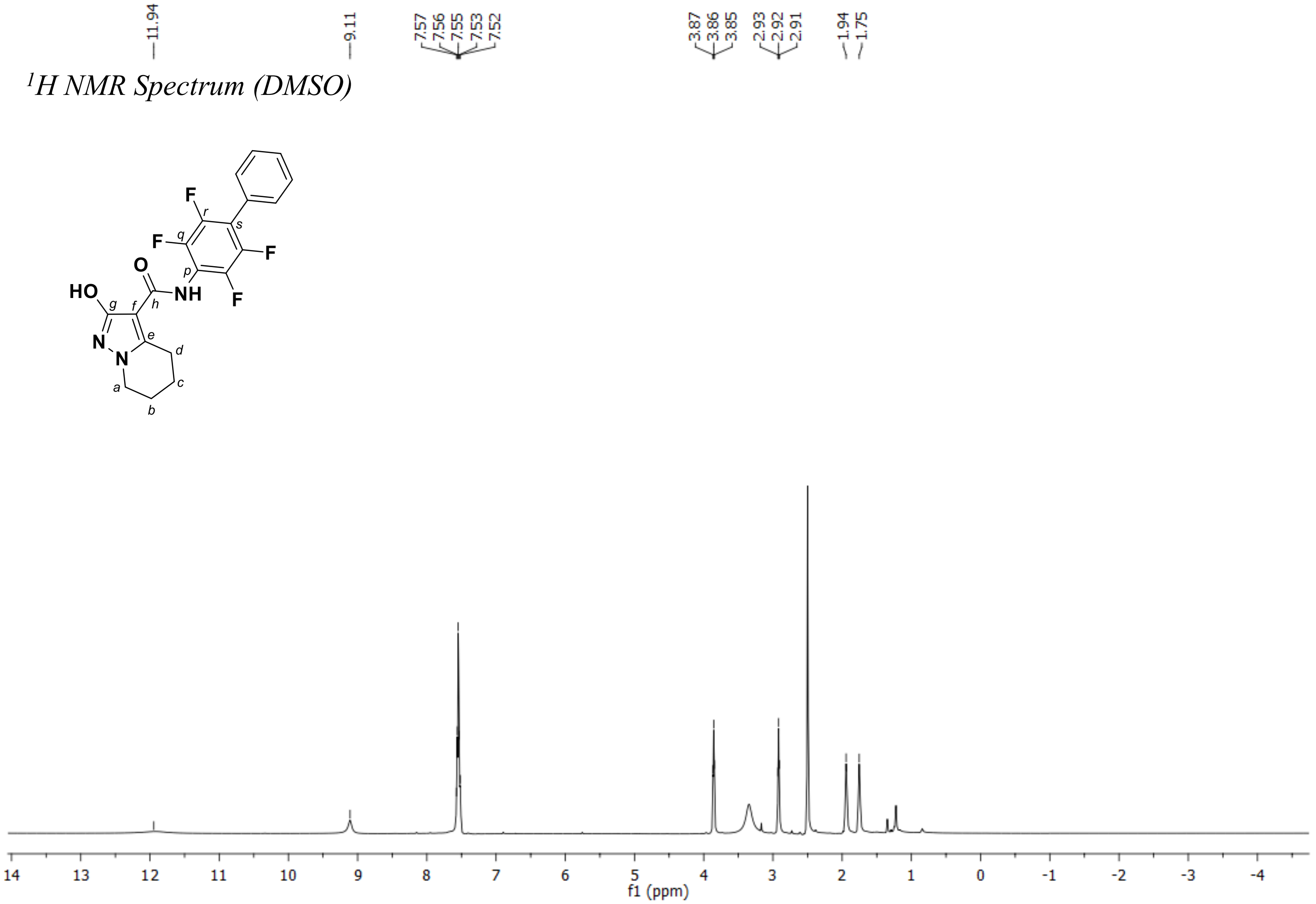

^{13}C NMR Spectrum (DMSO)

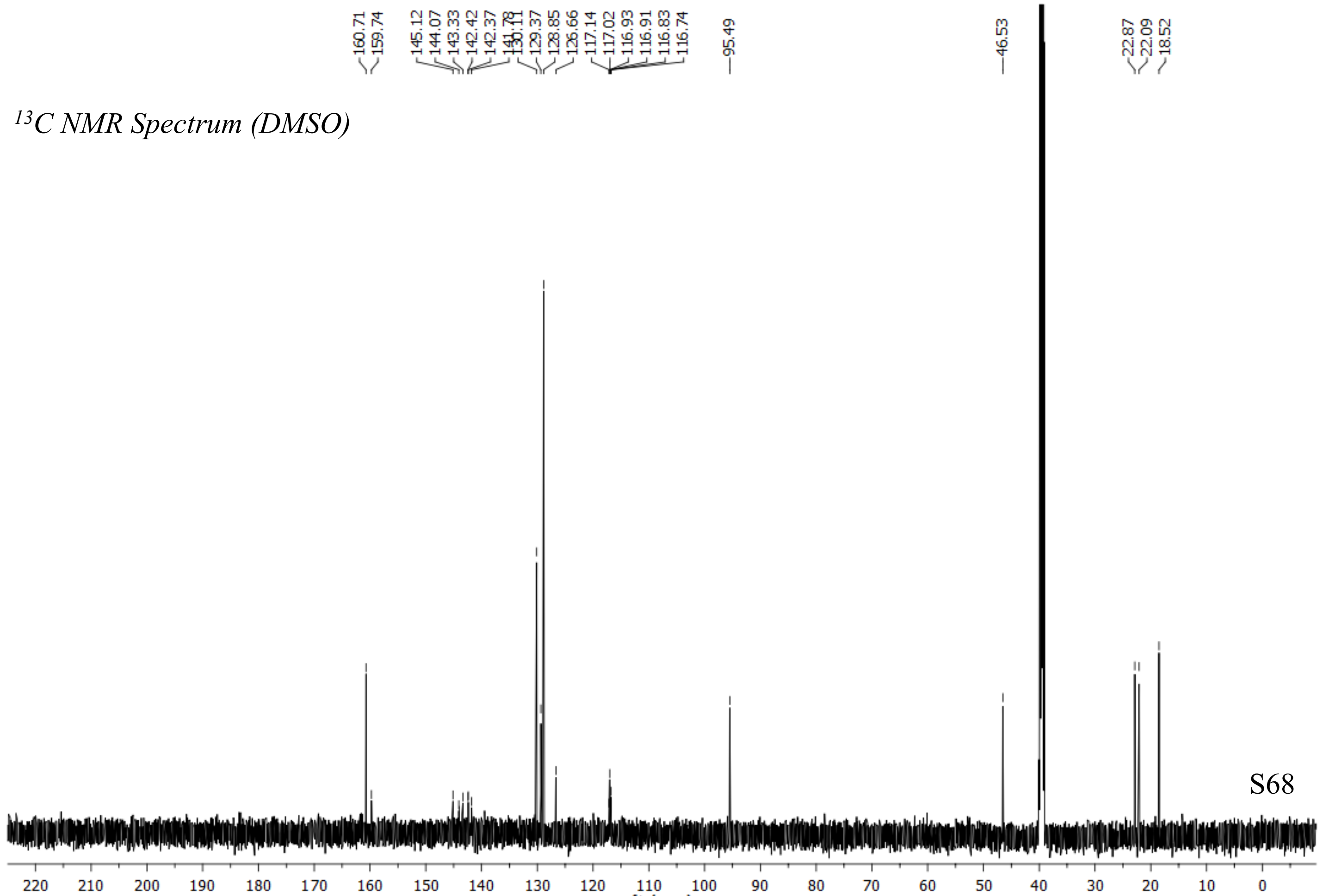

2-Hydroxy-N-(2,3,5,6-tetrafluoro-[1,1'-biphenyl]-4-yl)-4,5,6,7-tetrahydropyrazolo[1,5-a]pyridine-3-carboxamide (**5**)

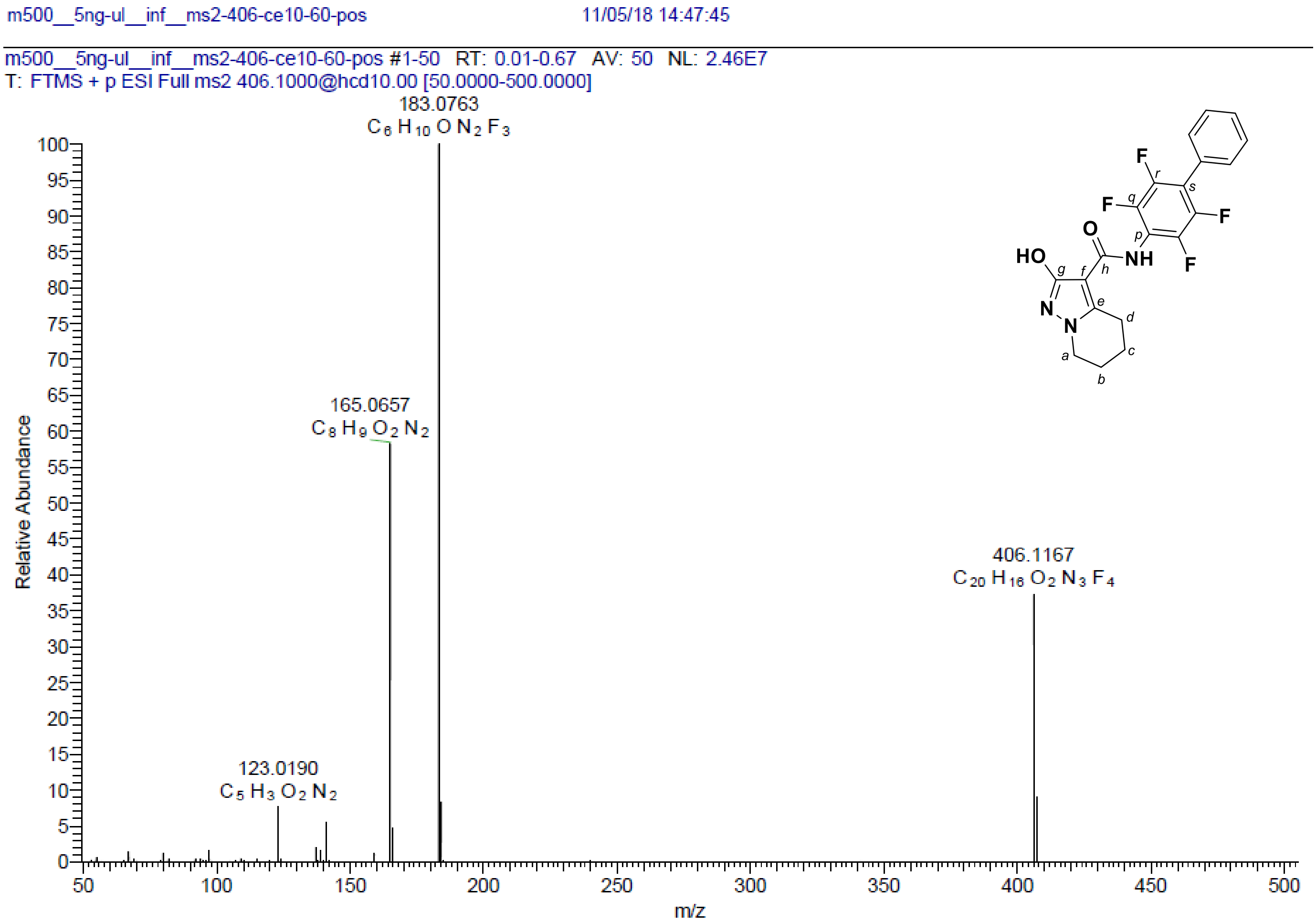

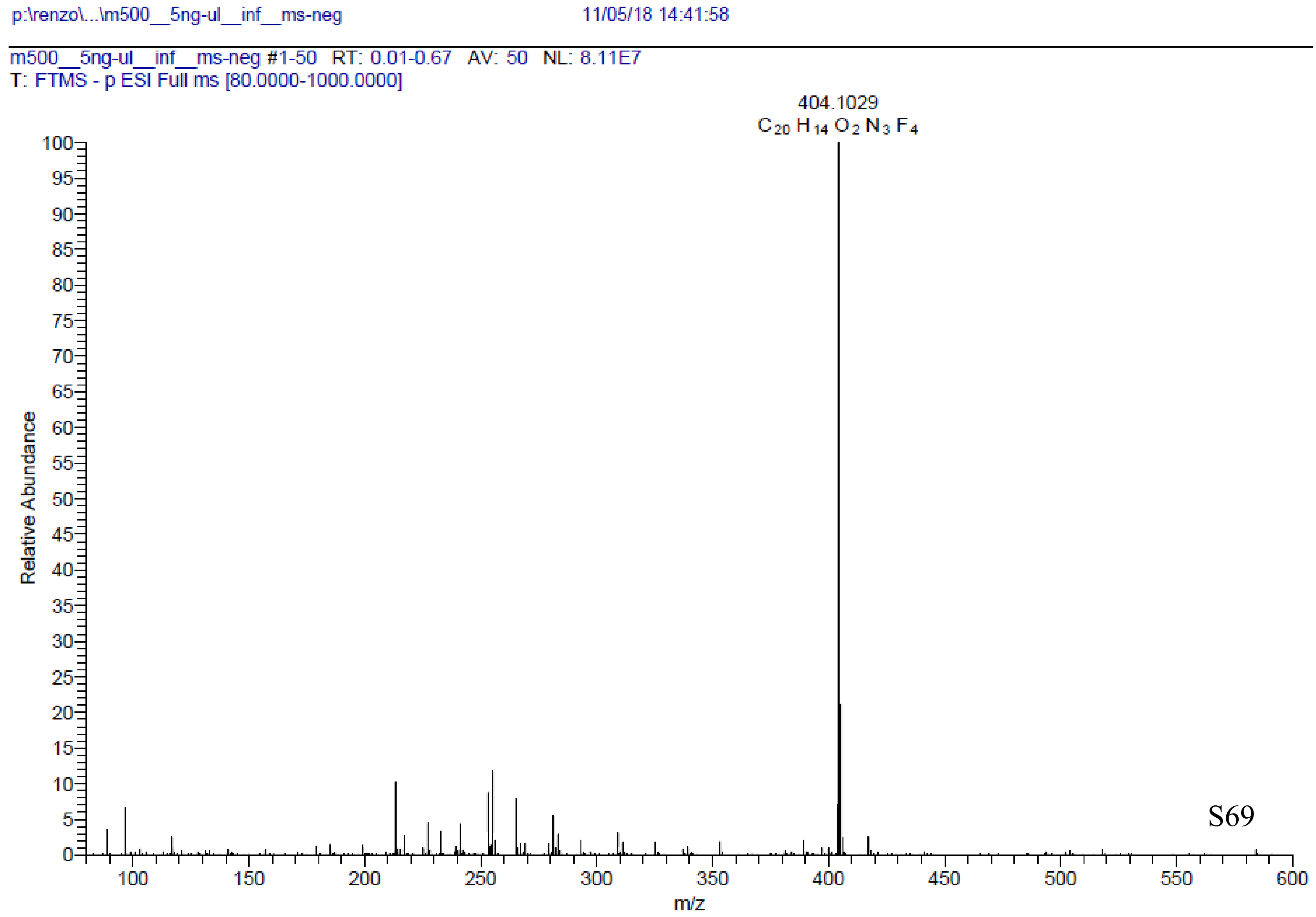

2-Hydroxy-N-(2,3,4',5,6-pentafluoro-[1,1'-biphenyl]-4-yl)pyrazolo[1,5-a]pyridine-3-carboxamide (**10**)

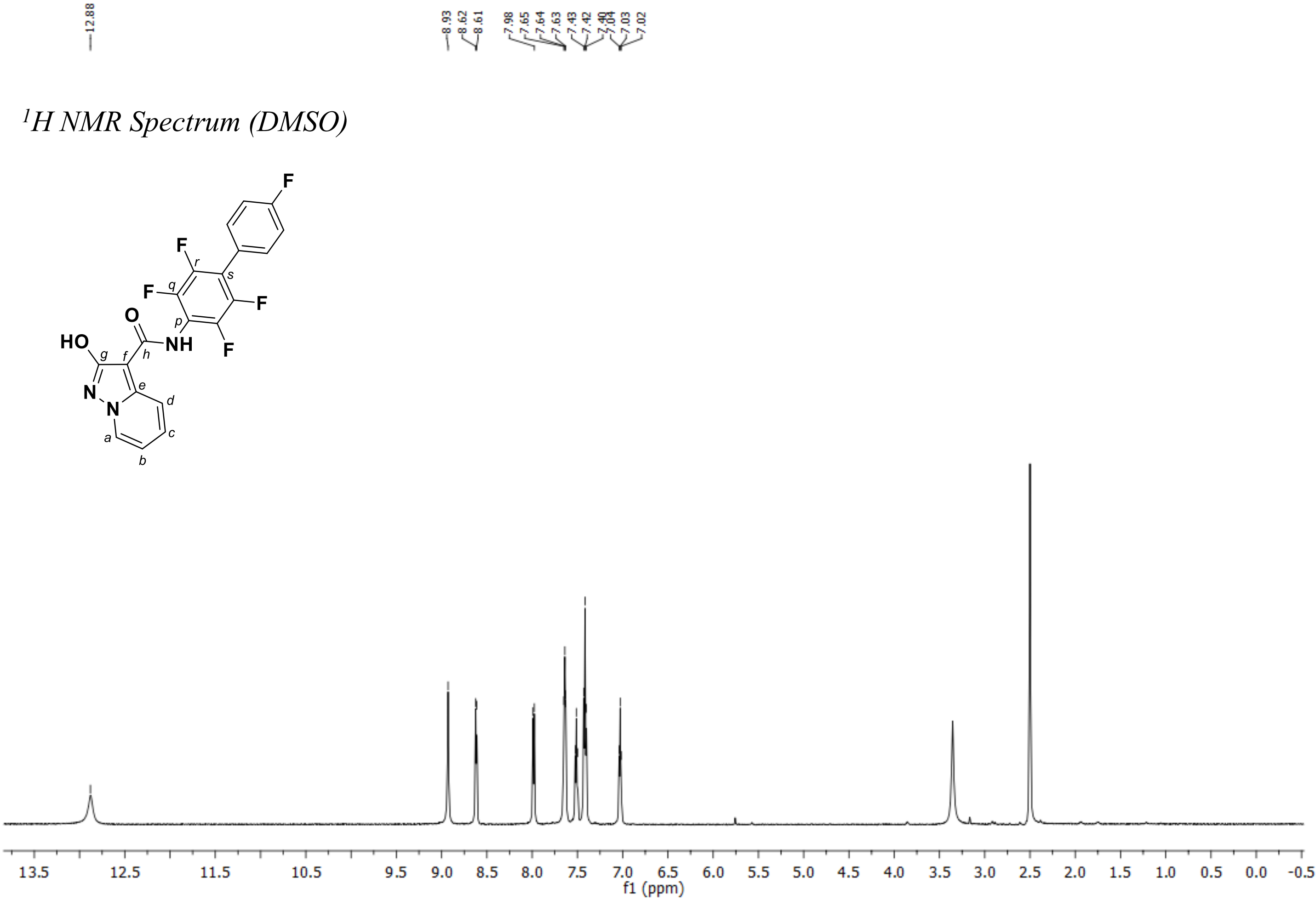

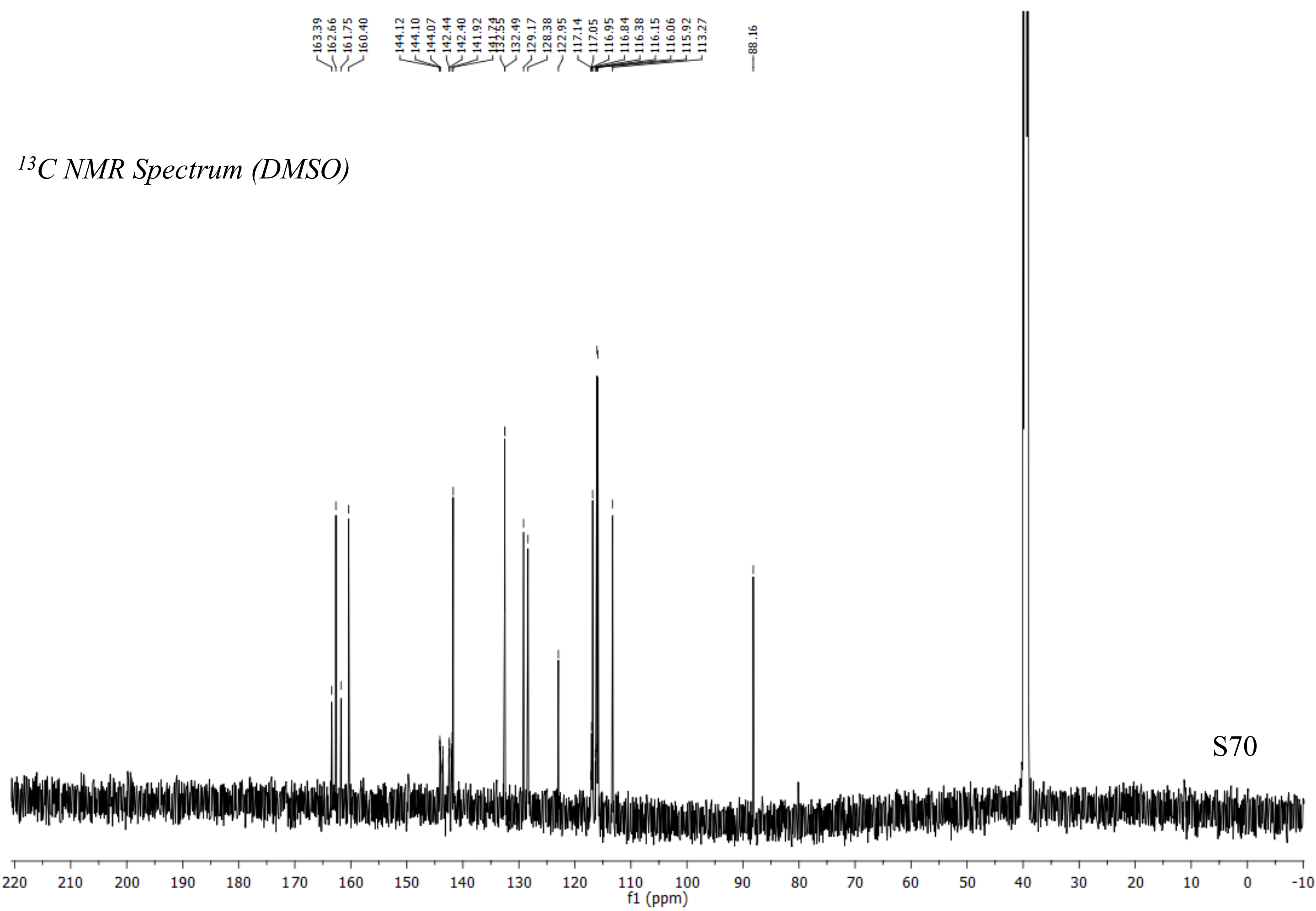

2-Hydroxy-N-(2,3,4',5,6-pentafluoro-[1,1'-biphenyl]-4-yl)pyrazolo[1,5-a]pyridine-3-carboxamide **(10)**

meds-602__10ng-ul_inf_ms-pos 01/23/20 11:49:07 MEDS-602__10ng-ul_inf_MS_30-pos

meds-602 10ng-ul inf ms-pos #1-20 RT: 0.01-0.29 AV: 20 NL: 6.40E6
T: FTMS + p ESI Full ms [300.00-580.00]

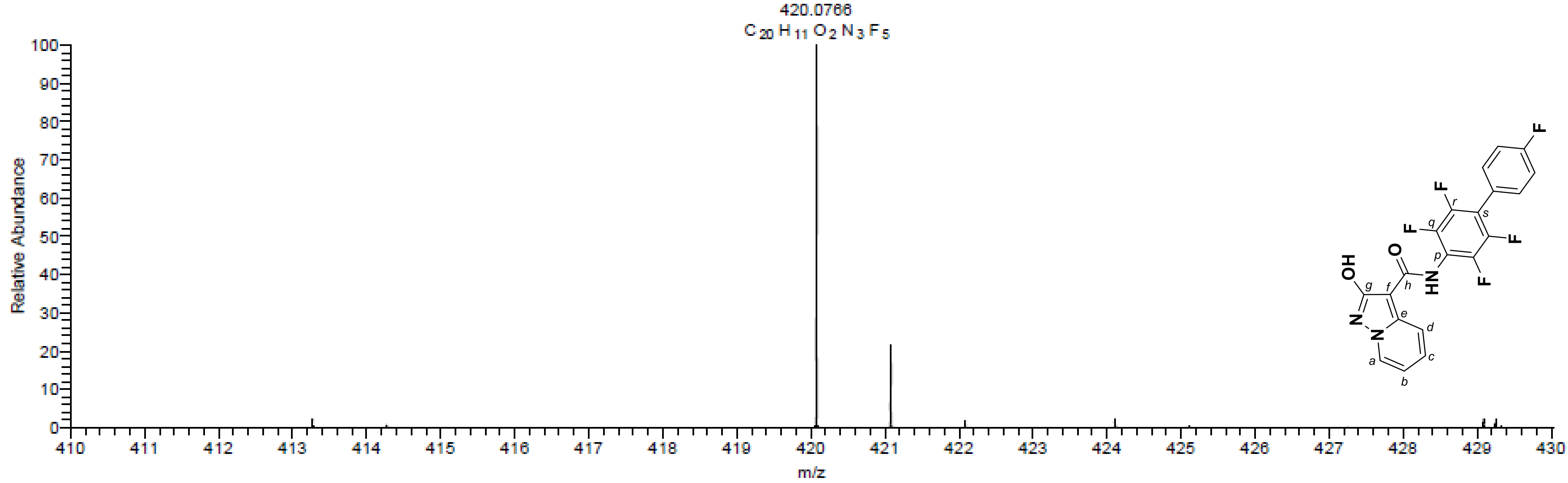

meds-602__10ng-ul_inf_ms2-hcd-35-pos #1-20 RT: 0.01-0.36 AV: 20 NL: 2.21E6
T: FTMS + p ESI Full ms2 420.00@hcd35.00 [50.00-460.00]

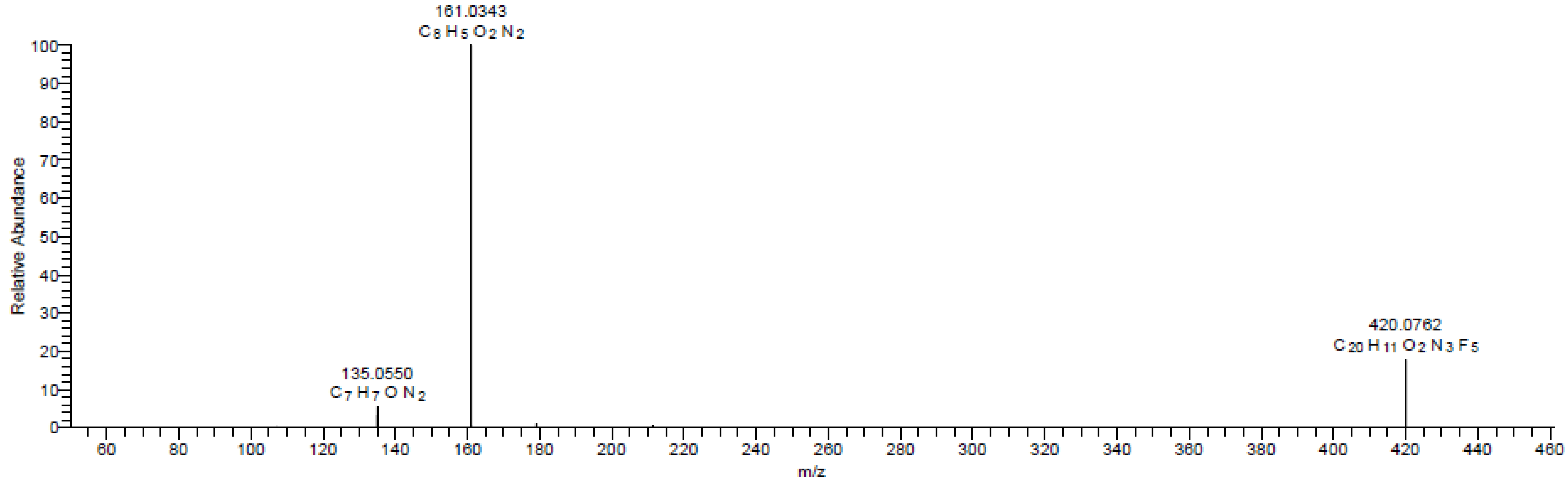

Report UHPLC

Acquisition Date/Time	5/14/2019 12:42:43 PM	Sample Name	SS1612A
Acquisition Method	B2 C18_95_ACN	Injection Volume	2
Instrument Name	UHPLC		

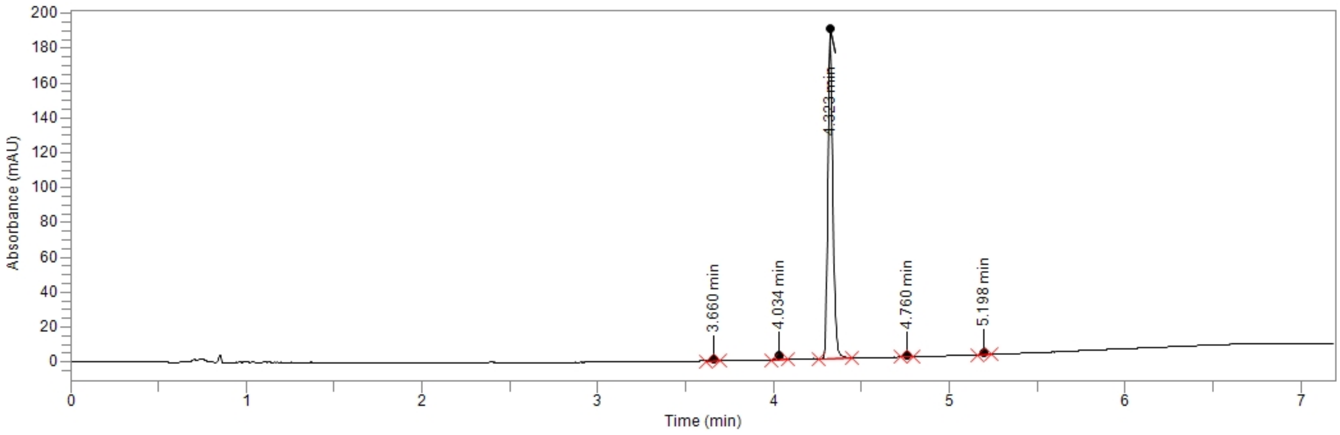

Channel Name **254:10:360:10**

Component Name	Time	Height	Area	Area %	Peak Purity	Wavelength Maximum
	3.660	976.4	2,094.9	0.55	2.18Fail	223
	4.034	2,212.7	4,279.8	1.12	5.15Fail	224
	4.323	189,665.0	372,787.7	97.50	2.62Fail	223
	4.760	861.4	1,577.5	0.41	1.12Pass	227
	5.198	787.6	1,613.1	0.42	1.08Pass	228
Total			382,353.0	100.00		

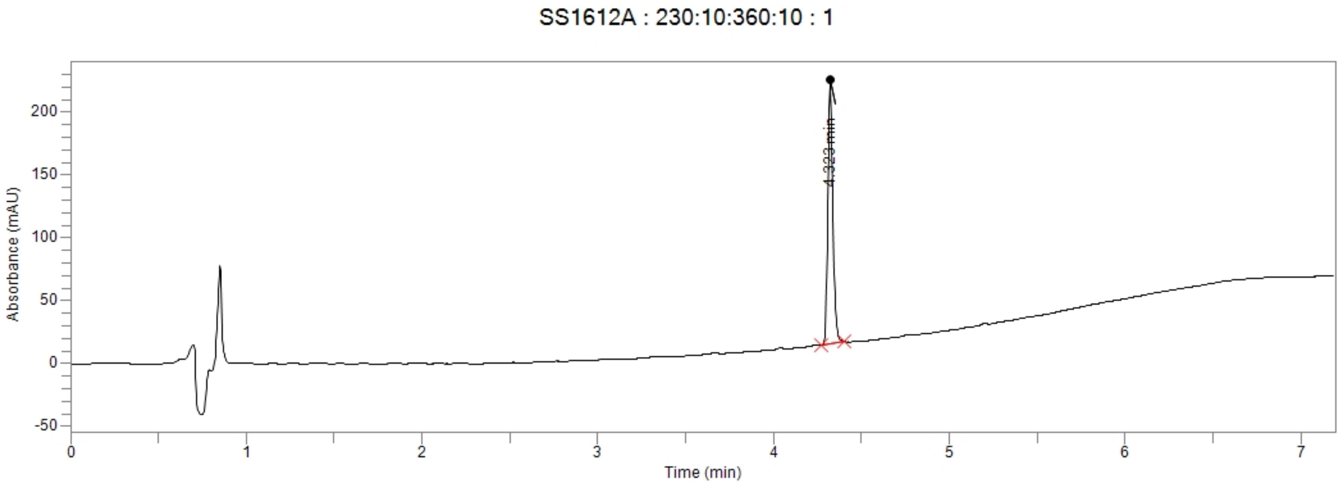

Channel Name **230:10:360:10**

Component Name	Time	Height	Area	Area %	Peak Purity	Wavelength Maximum
	4.323	210,925.4	411,333.6	100.00	2.62Fail	223
Total			411,333.6	100.00		

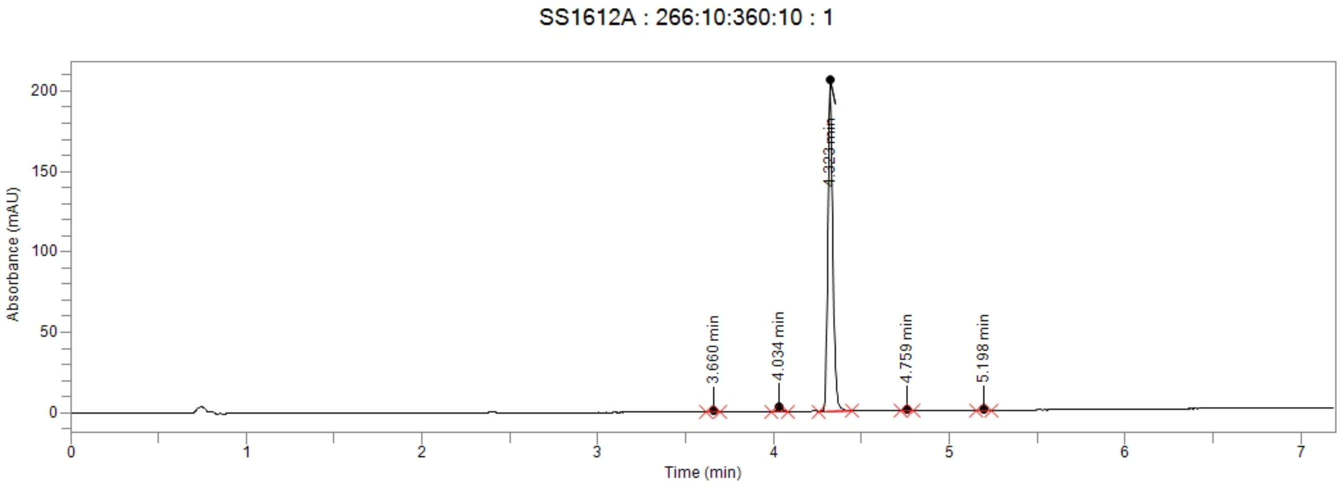

Channel Name **266:10:360:10**

Component Name	Time	Height	Area	Area %	Peak Purity	Wavelength Maximum
	3.660	922.4	2,008.4	0.48	2.18Fail	223
	4.034	2,727.7	5,344.3	1.28	5.15Fail	224
	4.323	206,499.4	406,177.9	97.40	2.62Fail	223
	4.759	905.0	1,624.7	0.39	1.10Pass	227
	5.198	917.5	1,857.7	0.45	1.09Pass	228
Total			417,012.9	100.00		

2-Hydroxy-N-(2,3,3',5,6-pentafluoro-[1,1'-biphenyl]-4-yl)pyrazolo[1,5-a]pyridine-3-carboxamide **(11)**

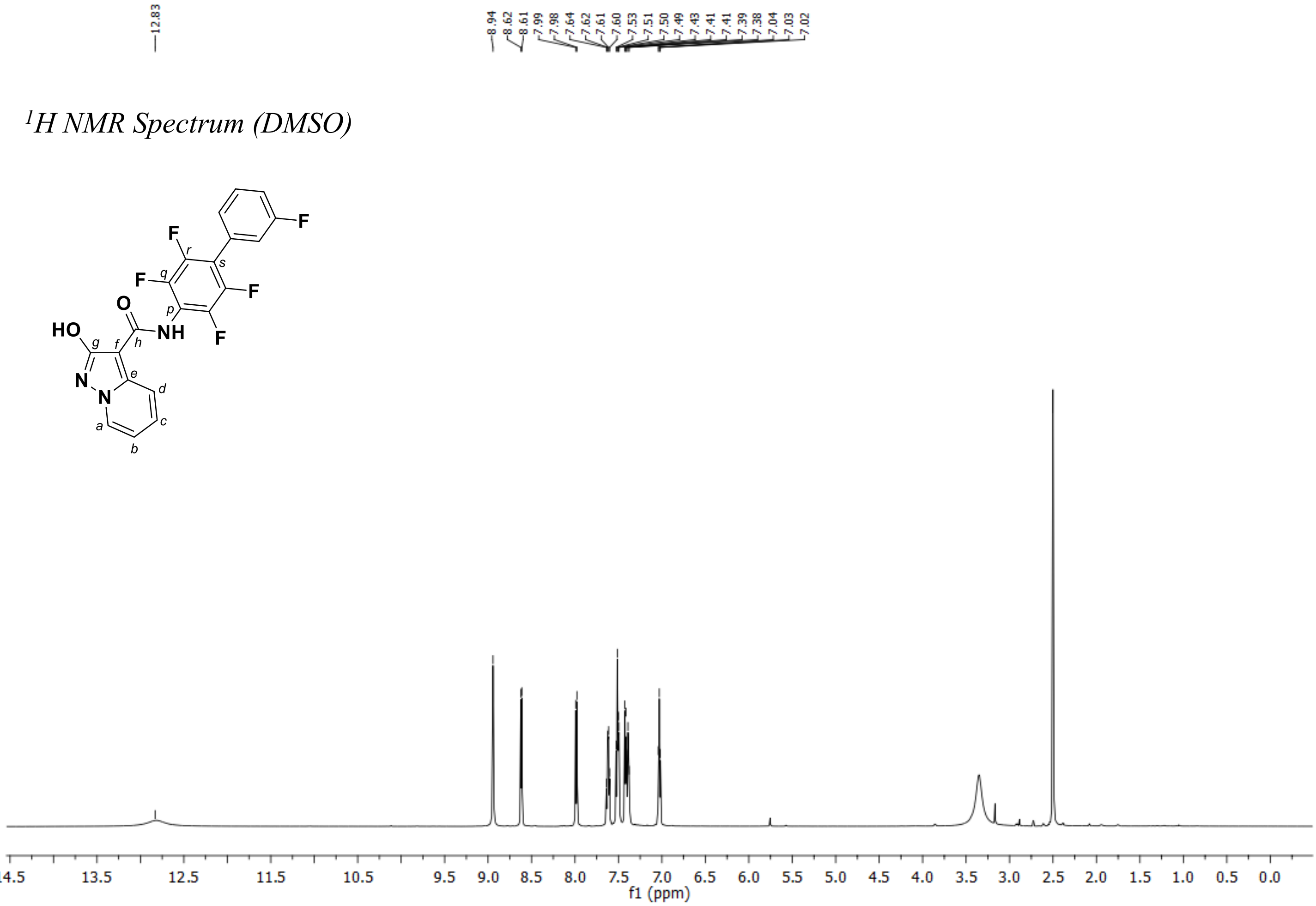

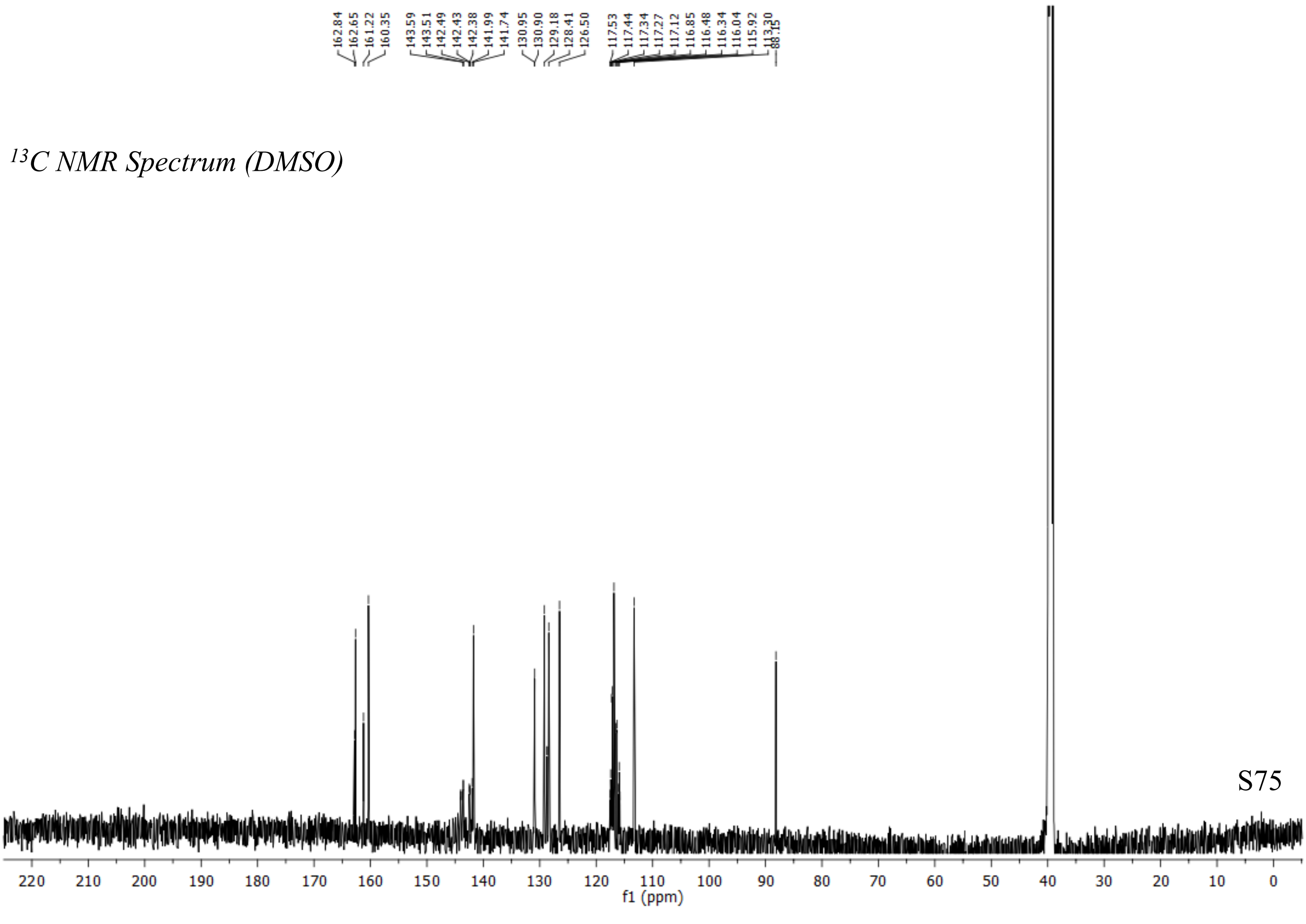

2-Hydroxy-N-(2,3,3',5,6-pentafluoro-[1,1'-biphenyl]-4-yl)pyrazolo[1,5-a]pyridine-3-carboxamide (**11**)

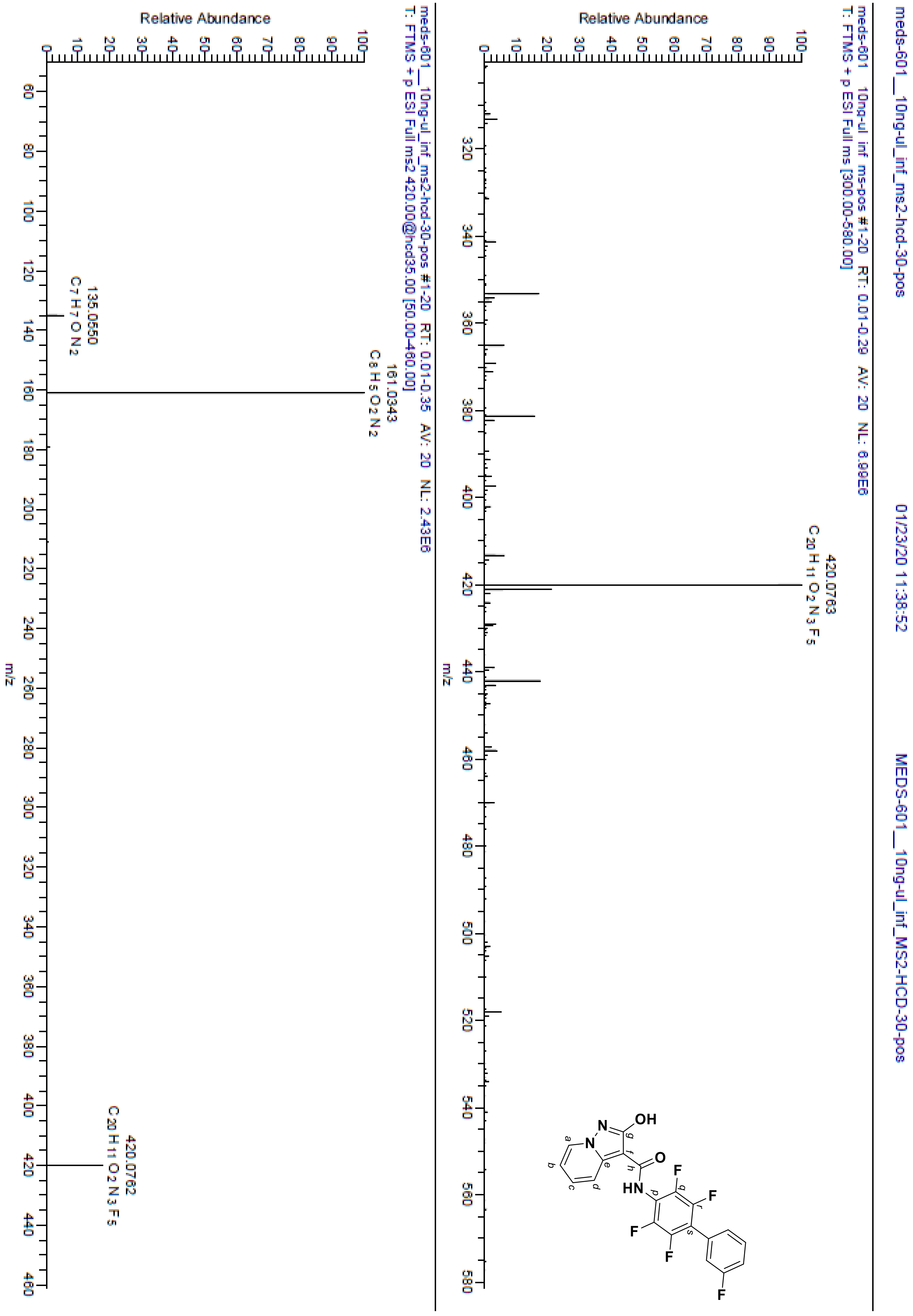

Report UHPLC

Acquisition Date/Time	5/16/2019 3:42:02 PM	Sample Name	SS1616
Acquisition Method	B1 C18	Injection Volume	2
Instrument Name	UHPLC		

SS1616 : 266:10:360:10 : 1

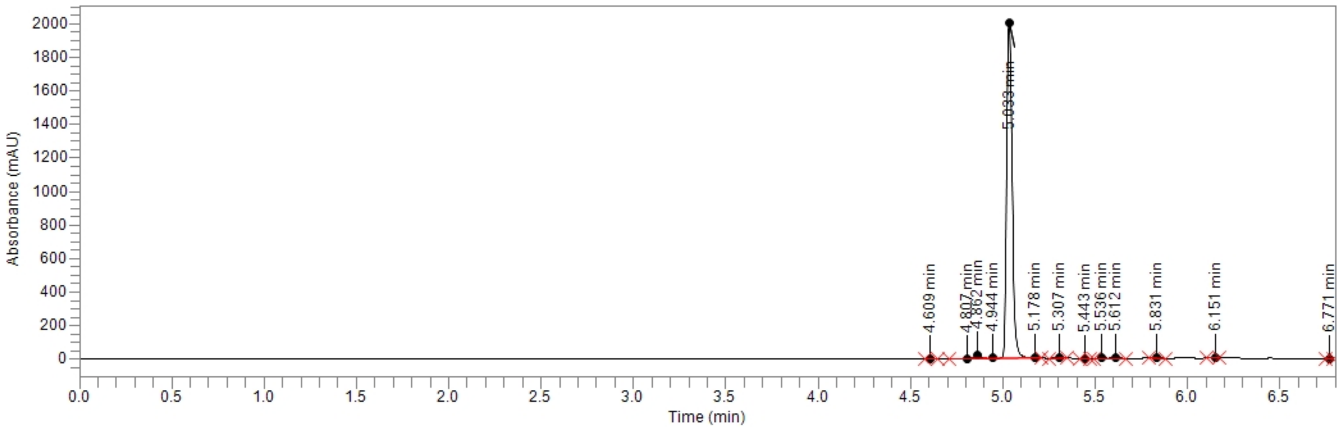

Channel Name **266:10:360:10**

Component Name	Time	Height	Area	Area %	Peak Purity	Wavelength Maximum
	4.609	862.3	1,863.9	0.04	13.86Fail	223
	4.807	1,863.6	6,220.3	0.15	1.22Pass	224
	4.862	22,875.5	51,758.1	1.22	1.23Pass	223
	4.944	1,962.1	3,814.1	0.09	1.36Pass	224
	5.033	2,002,970.6	4,154,080.7	97.71	1.30Pass	223
	5.178	1,543.2	6,007.9	0.14	1.79Fail	224
	5.307	1,135.6	3,970.0	0.09	1.22Pass	225
	5.443	459.5	678.5	0.02	1.12Pass	227
	5.536	3,630.3	6,786.6	0.16	1.23Pass	227
	5.612	5,426.2	11,090.2	0.26	1.10Pass	227
	5.831	1,749.6	3,568.0	0.08	1.12Pass	228
	6.151	617.8	1,492.1	0.04	1.10Pass	228
	6.771	278.6	312.6	0.01	1.07Pass	228
Total			4,251,643.1	100.00		

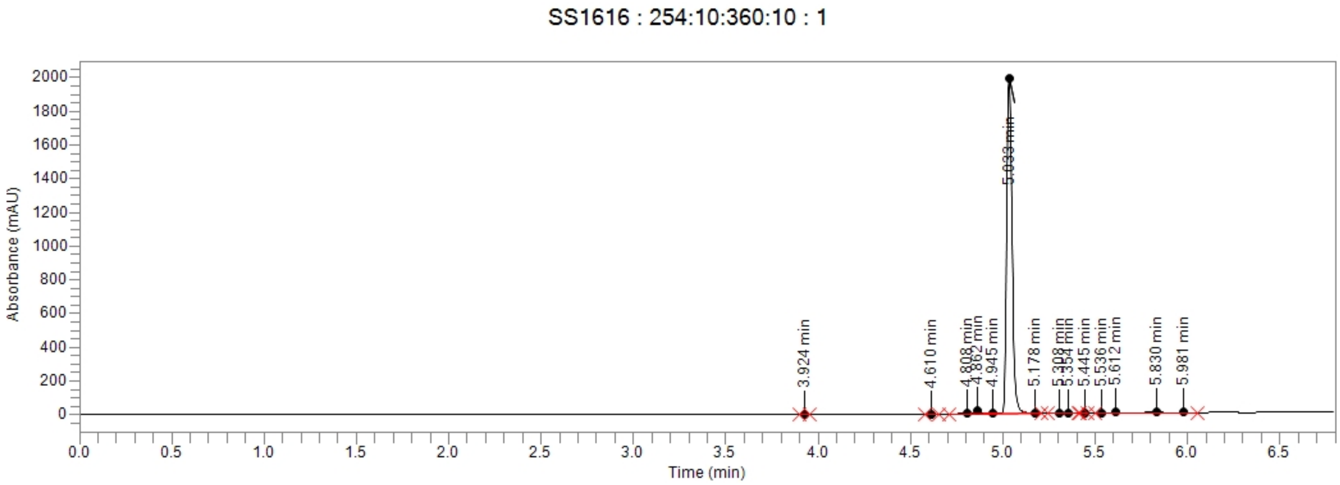

Channel Name 254:10:360:10

Component Name	Time	Height	Area	Area %	Peak Purity	Wavelength Maximum
	3.924	715.5	1,228.5	0.03	2.16Fail	200
	4.610	747.3	1,687.6	0.04	2.85Fail	223
	4.808	1,586.3	5,208.9	0.13	1.22Pass	224
	4.862	19,127.8	43,339.7	1.05	1.23Pass	223
	4.945	1,765.2	3,331.1	0.08	1.36Pass	224
	5.033	1,987,115.1	4,012,147.6	96.95	1.34Pass	223
	5.178	1,483.8	5,686.4	0.14	1.82Fail	224
	5.308	1,570.5	5,869.2	0.14	1.21Pass	227
	5.354	1,139.3	2,517.6	0.06	1.38Pass	227
	5.445	271.9	395.4	0.01	1.18Pass	227
	5.536	3,031.4	5,401.5	0.13	1.20Pass	227
	5.612	5,398.6	14,463.4	0.35	1.27Pass	227
	5.830	5,028.6	27,466.5	0.66	1.26Pass	228
	5.981	1,706.3	9,617.7	0.23	1.23Pass	227
Total			4,138,361.0	100.00		

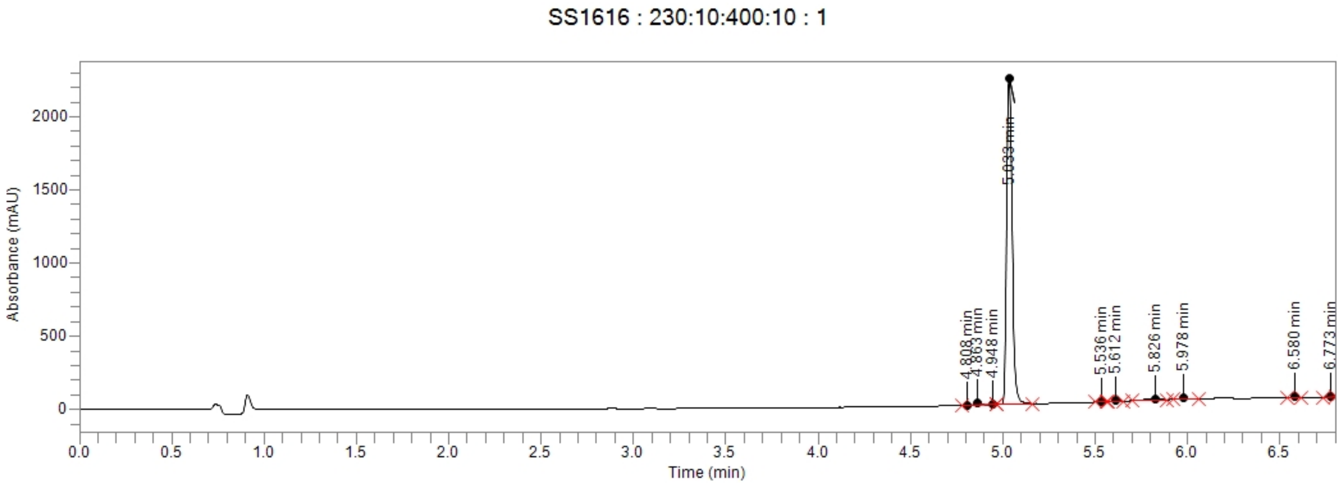

Channel Name **230:10:400:10**

Component Name	Time	Height	Area	Area %	Peak Purity	Wavelength Maximum
	4.808	1,971.6	3,404.9	0.07	1.14Pass	224
	4.863	12,383.4	26,815.0	0.56	1.20Pass	223
	4.948	1,484.8	2,560.5	0.05	1.15Pass	224
	5.033	2,229,986.9	4,646,012.4	96.50	1.30Pass	223
	5.536	6,375.6	10,556.3	0.22	1.32Pass	227
	5.612	9,216.1	17,389.7	0.36	1.13Pass	227
	5.826	10,370.4	58,732.3	1.22	1.24Pass	228
	5.978	7,508.7	30,020.3	0.62	1.40Pass	227
	6.580	4,889.7	8,513.3	0.18	1.06Pass	227
	6.773	5,061.3	10,403.7	0.22	1.04Pass	228
Total			4,814,408.3	100.00		

2-Hydroxy-N-(2,3,5,6-tetrafluoro-4'-(trifluoromethyl)-[1,1'-biphenyl]-4-yl)pyrazolo[1,5-a]pyridine-3-carboxamide (**12**)

^{1}H NMR Spectrum (DMSO)

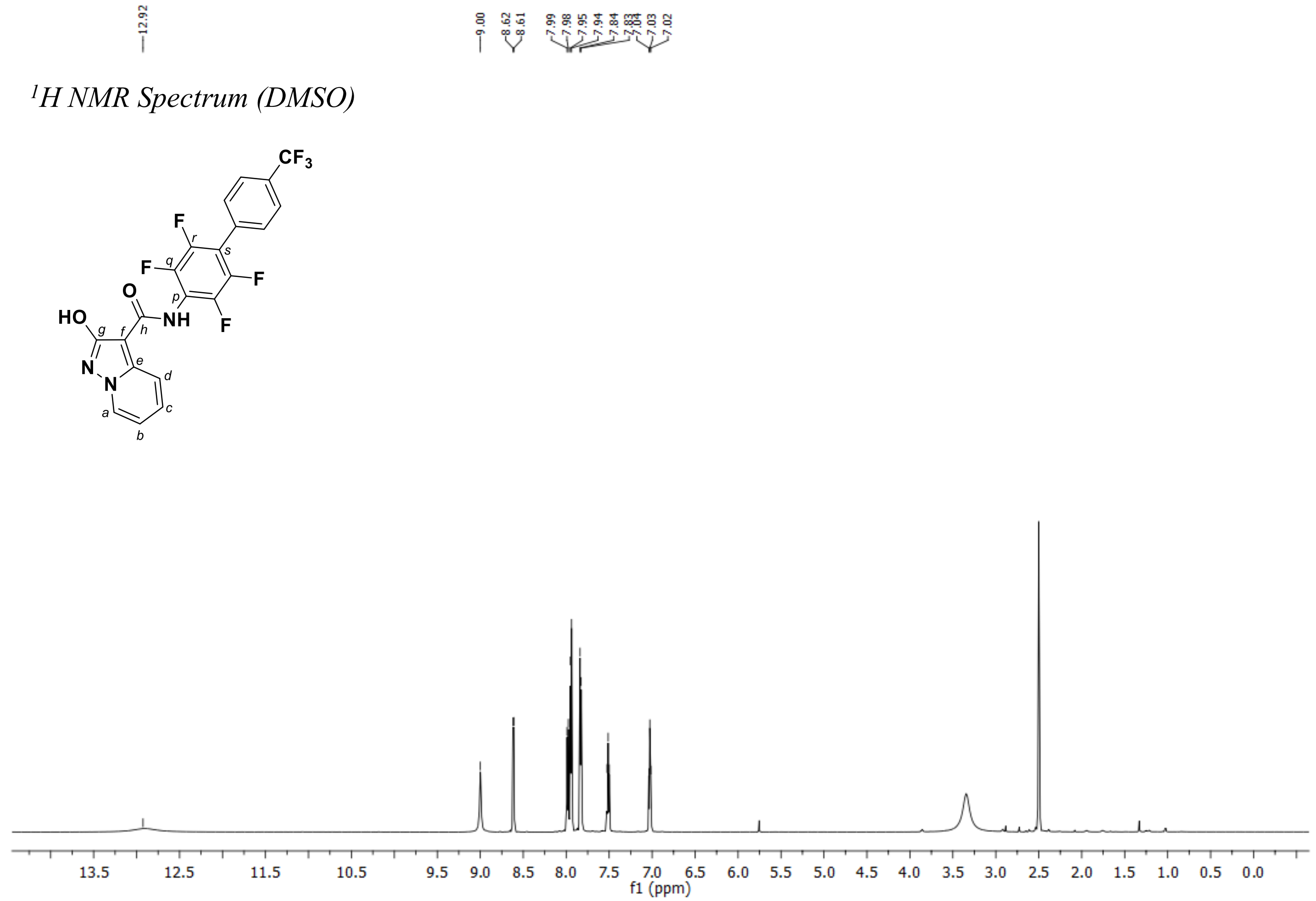

^{13}C NMR Spectrum (DMSO)

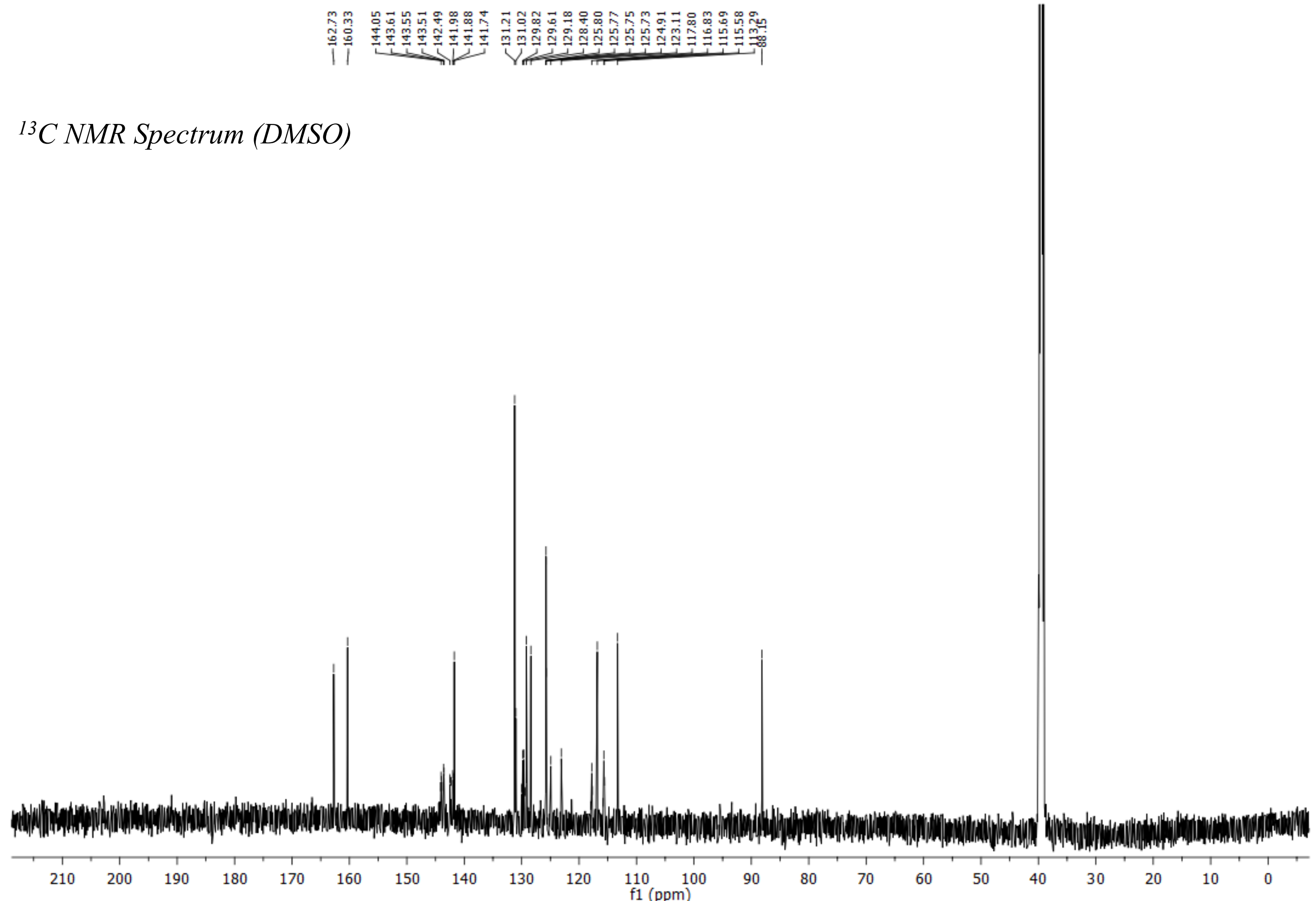

2-Hydroxy-N-(2,3,5,6-tetrafluoro-4'-(trifluoromethyl)-[1,1'-biphenyl]-4-yl)pyrazolo[1,5-a]pyridine-3-carboxamide (**12**)

meds-603__10ng-ul_inf_ms-pos 01/23/20 11:59:45 MEDS-603__10ng-ul_inf_MS-pos

meds-603 10ng-ul inf ms-pos #1-20 RT: 0.01-0.29 AV: 20 NL: 9.19E6
T: FTMS + p ESI Full ms [150.00-650.00]

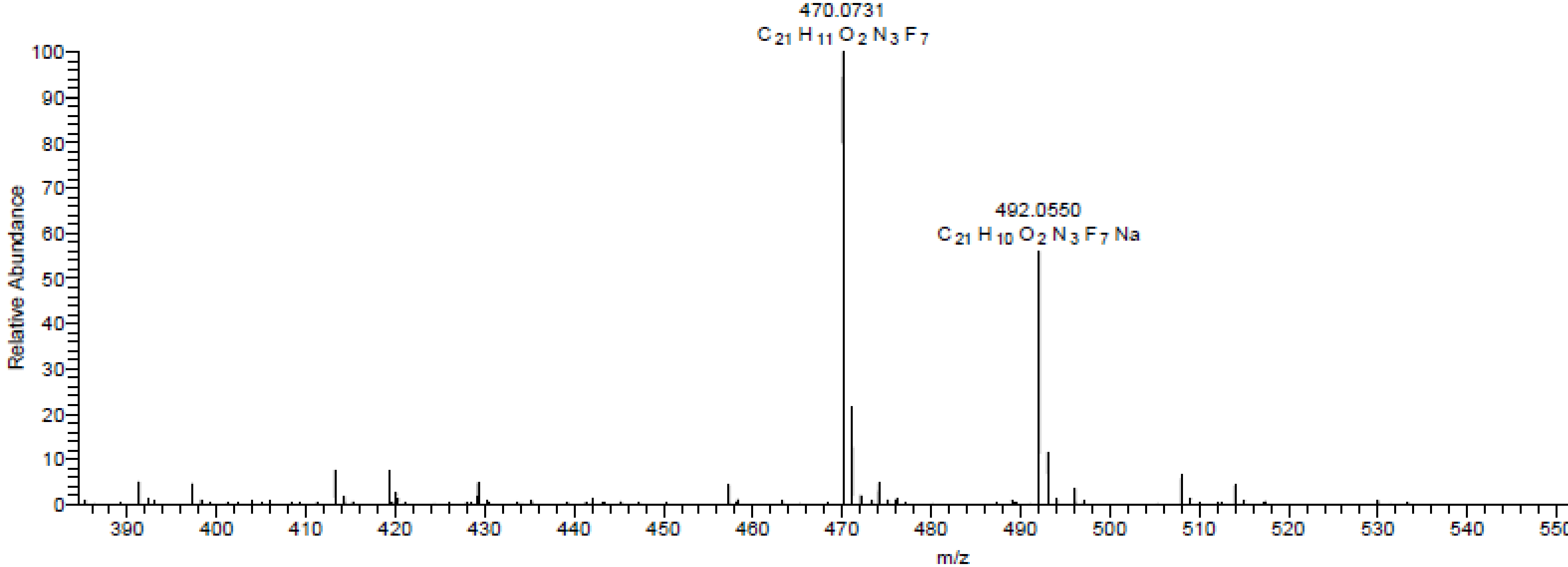

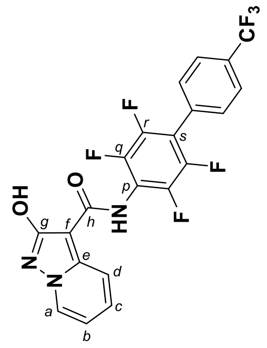

meds-603__10ng-ul_inf_ms2-hcd-35-pos #1-20 RT: 0.00-0.36 AV: 20 NL: 1.63E6
T: FTMS + p ESI Full ms2 470.00@hcd35.00 [50.00-500.00]

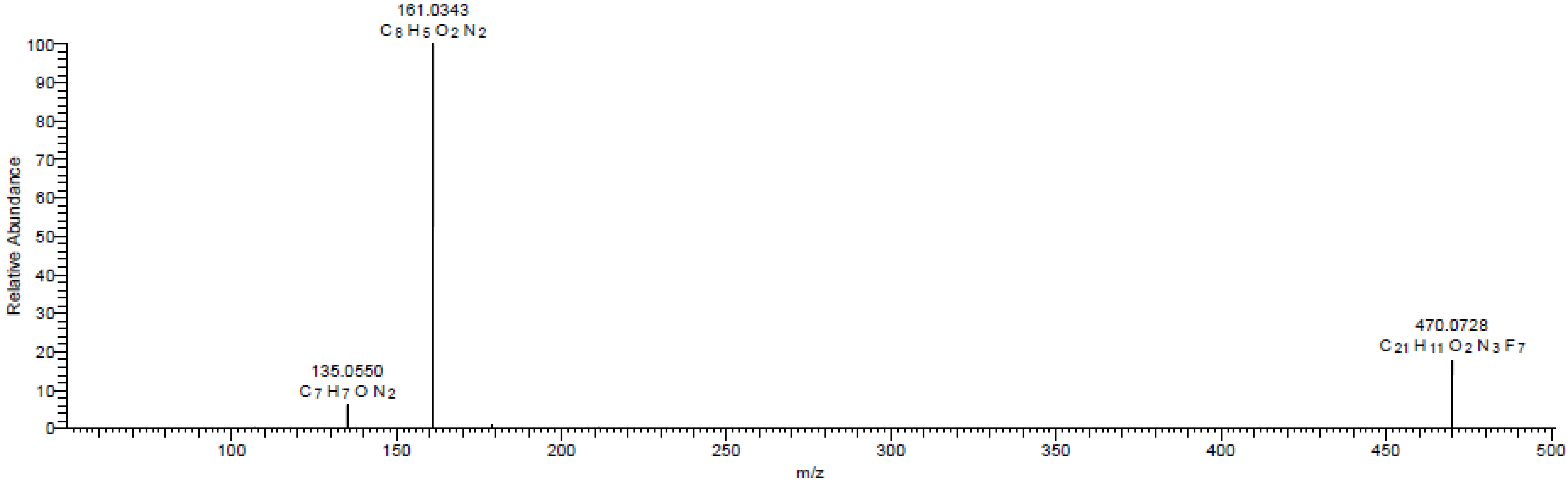

Report UHPLC

Acquisition Date/Time	5/20/2019 12:25:13 PM	Sample Name	SS1622
Acquisition Method	B2 C18_95_ACN	Injection Volume	2
Instrument Name	UHPLC		

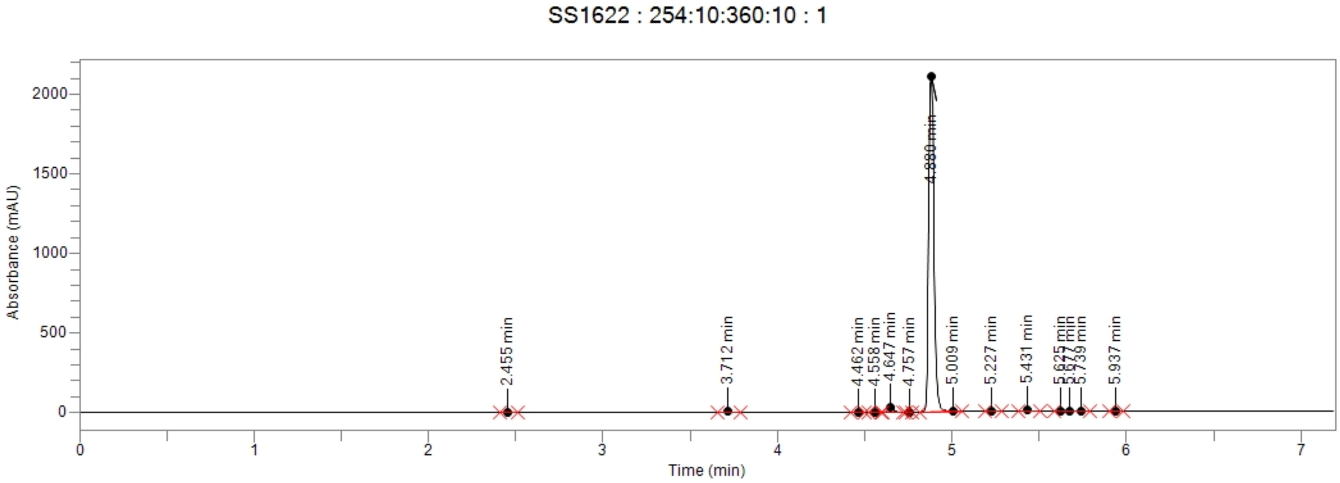

Channel Name **254:10:360:10**

Component Name	Time	Height	Area	Area %	Peak Purity	Wavelength Maximum
	2.455	670.7	2,200.2	0.05	Not Enough Valid Points	194
	3.712	5,028.4	16,148.9	0.36	1.48Pass	224
	4.462	1,029.3	2,132.1	0.05	1.14Pass	227
	4.558	1,066.9	1,894.4	0.04	1.16Pass	228
	4.647	30,417.6	59,461.5	1.31	1.22Pass	227
	4.757	306.4	403.2	0.01	1.17Pass	228
	4.880	2,117,504.5	4,396,747.1	96.93	1.24Pass	225
	5.009	3,475.8	14,285.5	0.31	2.63Fail	228
	5.227	4,392.0	8,308.9	0.18	1.11Pass	228
	5.431	12,381.4	25,073.9	0.55	1.21Pass	227
	5.625	593.1	927.9	0.02	1.13Pass	228
	5.677	791.9	2,122.0	0.05	1.09Pass	228
	5.739	1,327.4	2,748.8	0.06	1.07Pass	228
	5.937	1,793.2	3,381.8	0.07	1.06Pass	228
Total			4,535,836.1	100.00		

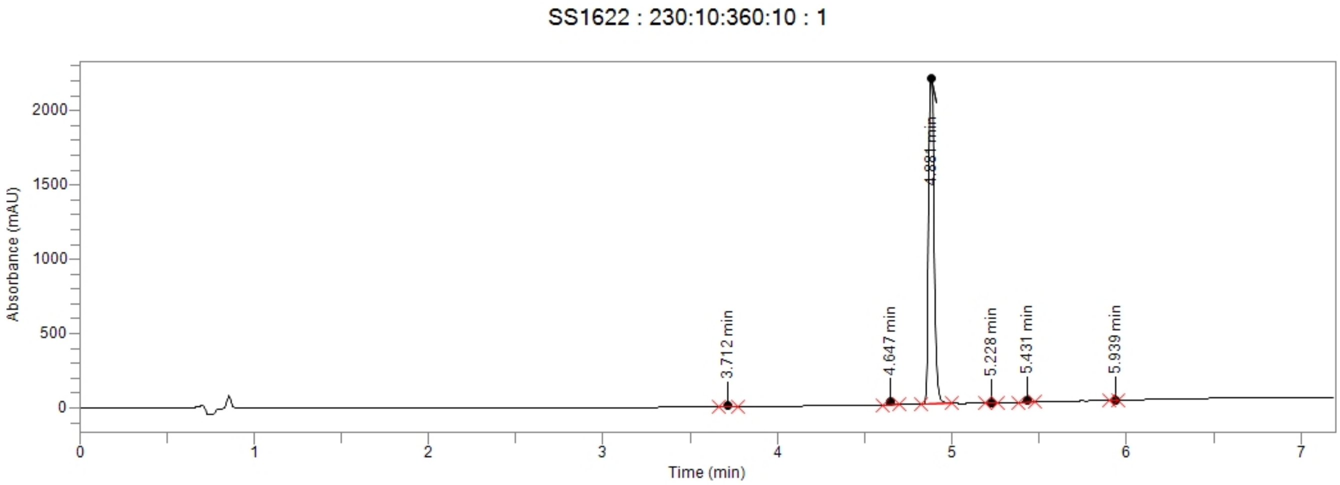

Channel Name **230:10:360:10**

Component Name	Time	Height	Area	Area %	Peak Purity	Wavelength Maximum
	3.712	6,938.2	19,631.7	0.40	2.35Fail	224
	4.647	20,572.1	39,221.6	0.80	1.22Pass	227
	4.881	2,202,322.5	4,812,978.4	98.11	1.24Pass	225
	5.228	4,087.3	7,214.5	0.15	1.13Pass	228
	5.431	12,535.3	24,157.1	0.49	1.18Pass	227
	5.939	1,506.5	2,354.4	0.05	1.05Pass	228
Total			4,905,557.9	100.00		

SS1622 : 266:10:360:10 : 1

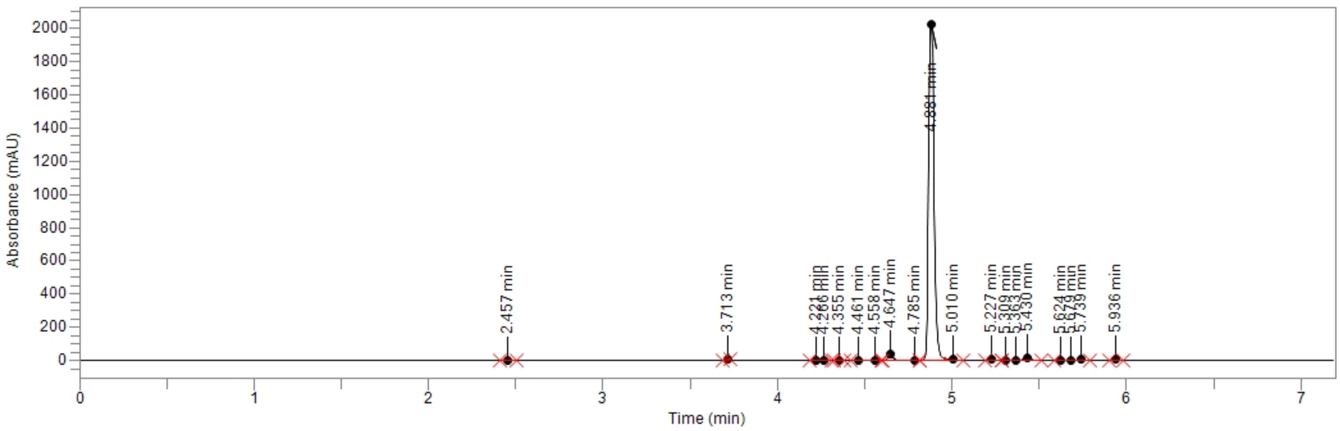

Channel Name **266:10:360:10**

Component Name	Time	Height	Area	Area %	Peak Purity	Wavelength Maximum
	2.457	564.9	1,675.2	0.04	Not Enough Valid Points	194
	3.713	2,214.7	3,807.4	0.08	6.28Fail	224
	4.221	328.5	587.6	0.01	1.30Pass	228
	4.266	783.8	1,403.2	0.03	1.25Pass	228
	4.355	495.2	843.5	0.02	1.18Pass	228
	4.461	1,285.2	3,319.0	0.07	1.23Pass	227
	4.558	1,277.3	2,549.2	0.06	1.17Pass	228
	4.647	37,220.7	73,081.9	1.62	1.22Pass	227
	4.785	670.8	2,121.1	0.05	1.18Pass	228
	4.881	2,033,322.1	4,358,043.2	96.51	1.24Pass	225
	5.010	4,007.0	17,341.2	0.38	2.83Fail	228
	5.227	5,176.7	10,087.9	0.22	1.11Pass	228
	5.309	321.1	475.9	0.01	1.17Pass	229
	5.363	419.8	736.4	0.02	1.12Pass	228
	5.430	14,433.4	29,472.7	0.65	1.21Pass	227
	5.624	542.8	945.4	0.02	1.11Pass	228
	5.679	878.9	2,230.2	0.05	1.09Pass	228
	5.739	1,541.5	3,093.6	0.07	1.05Pass	228
	5.936	2,121.2	3,965.3	0.09	1.06Pass	228
Total			4,515,780.1	100.00		

2-Hydroxy-N-(2,3,5,6-tetrafluoro-3'-(trifluoromethyl)-[1,1'-biphenyl]-4-yl)pyrazolo[1,5-a]pyridine-3-carboxamide (**13**)

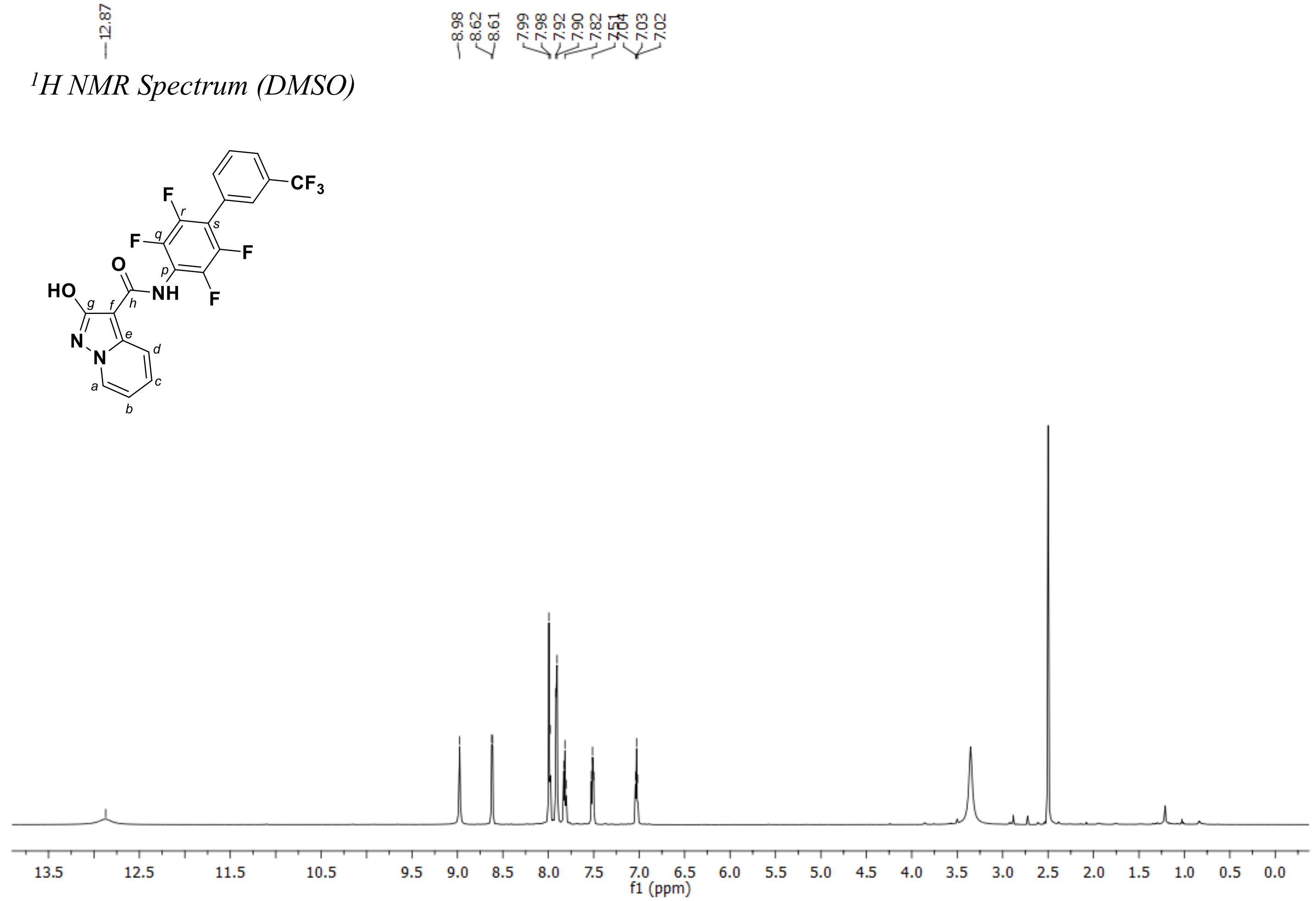

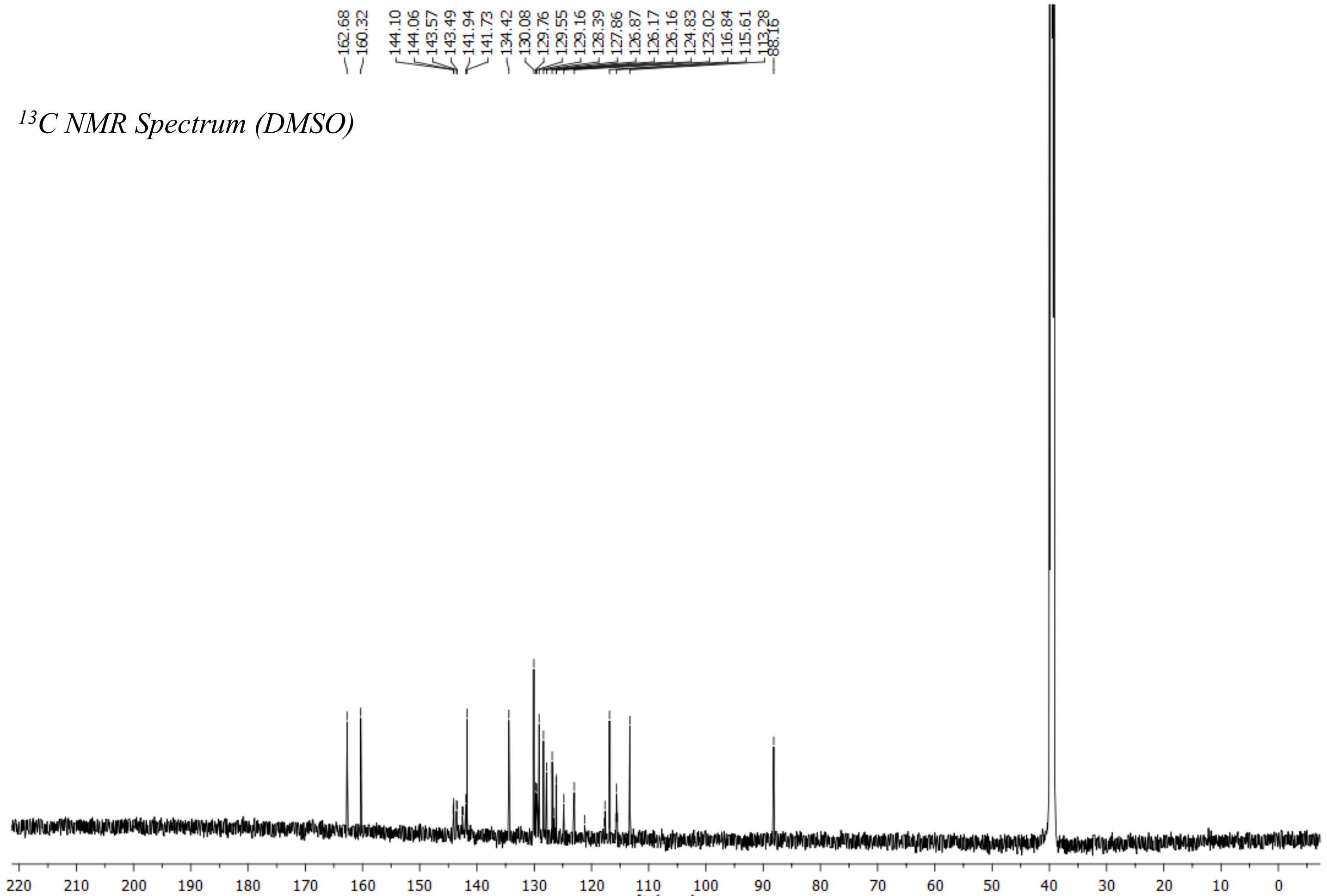

2-Hydroxy-N-(2,3,5,6-tetrafluoro-3'-(trifluoromethyl)-[1,1'-biphenyl]-4-yl)pyrazolo[1,5-a]pyridine-3-carboxamide (**13**)

MEDS-600__10ng-ul_inf_MS-pos 01/23/20 10:21:13 MEDS-600__10ng-ul_inf_MS-pos

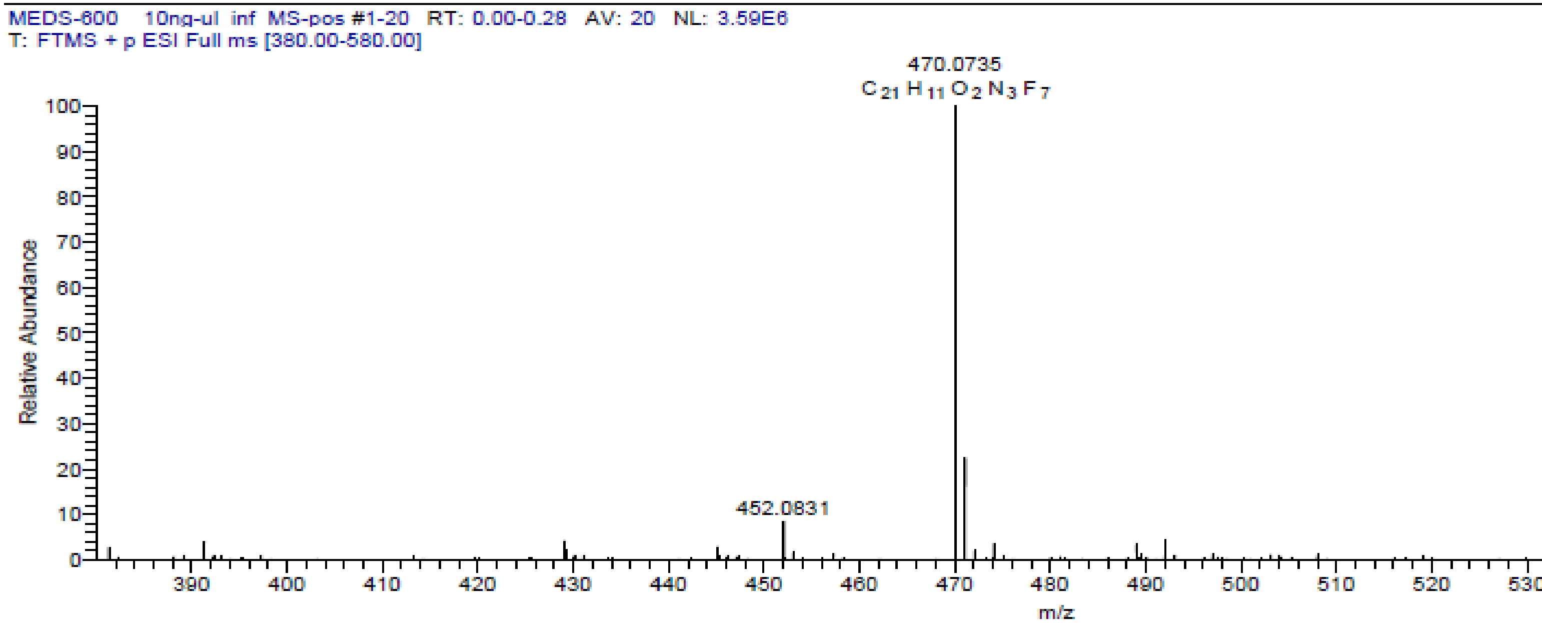

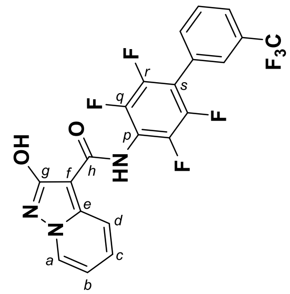

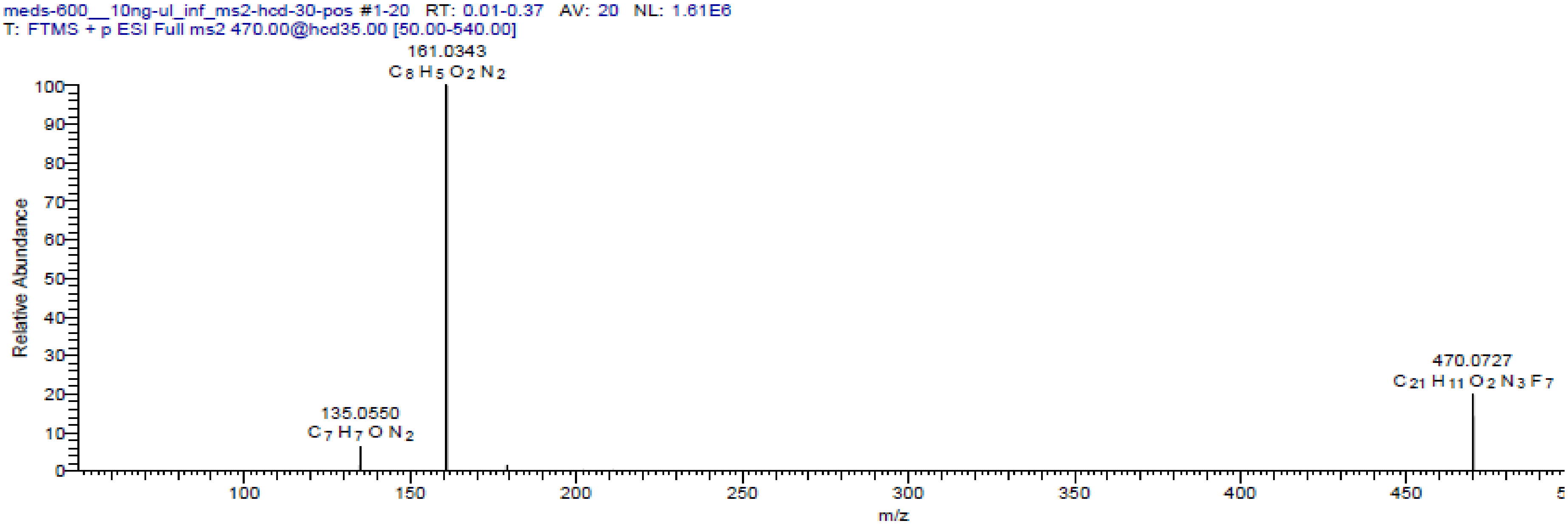

Report UHPLC

Acquisition Date/Time	5/14/2019 12:16:01 PM	Sample Name	SS1602A
Acquisition Method	B2 C18_95_ACN	Injection Volume	2
Instrument Name	UHPLC		

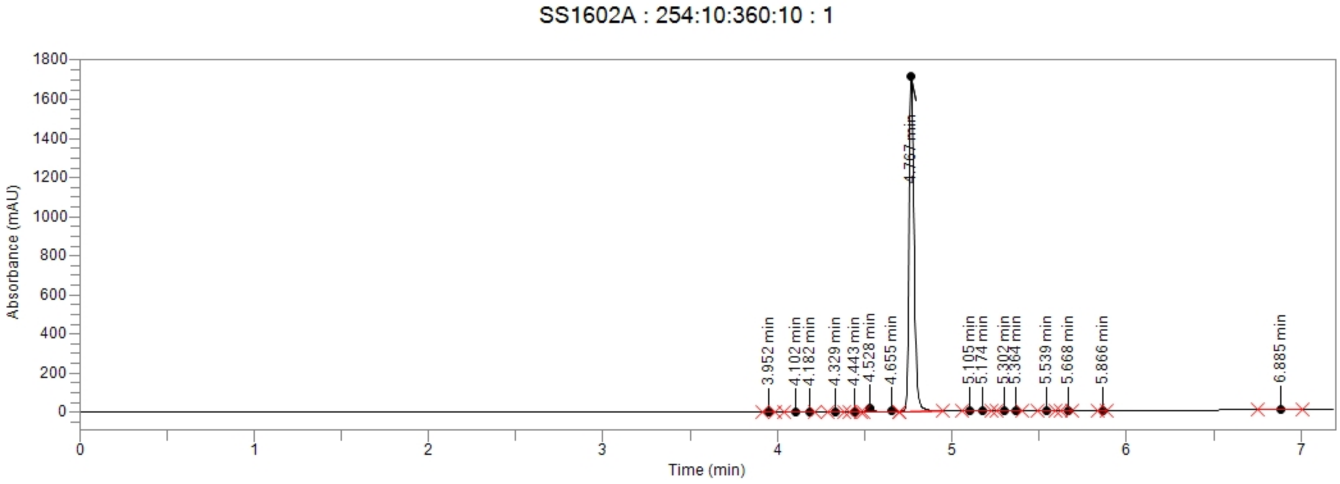

Channel Name **254:10:360:10**

Component Name	Time	Height	Area	Area %	Peak Purity	Wavelength Maximum
	3.952	493.7	1,130.7	0.03	1.40Pass	223
	4.102	1,615.6	4,837.6	0.14	1.57Fail	223
	4.182	553.6	1,098.5	0.03	1.27Pass	224
	4.329	2,176.0	4,524.1	0.13	1.39Pass	224
	4.443	846.5	1,543.4	0.04	1.33Pass	225
	4.528	21,205.8	42,167.0	1.19	1.40Pass	223
	4.655	2,066.9	4,535.1	0.13	1.21Pass	225
	4.767	1,717,232.5	3,433,445.4	97.29	1.17Pass	224
	5.105	892.8	1,920.5	0.05	1.28Pass	225
	5.174	1,012.1	2,591.9	0.07	1.16Pass	225
	5.302	1,395.1	2,925.0	0.08	1.17Pass	225
	5.364	920.2	2,032.4	0.06	1.21Pass	225
	5.539	3,110.6	6,344.2	0.18	1.13Pass	227
	5.668	313.0	938.2	0.03	1.22Pass	227
	5.866	297.8	522.4	0.01	1.09Pass	227
	6.885	2,566.4	18,406.9	0.52	1.06Pass	228
Total			3,528,963.2	100.00		

SS1602A : 230:10:360:10 : 1

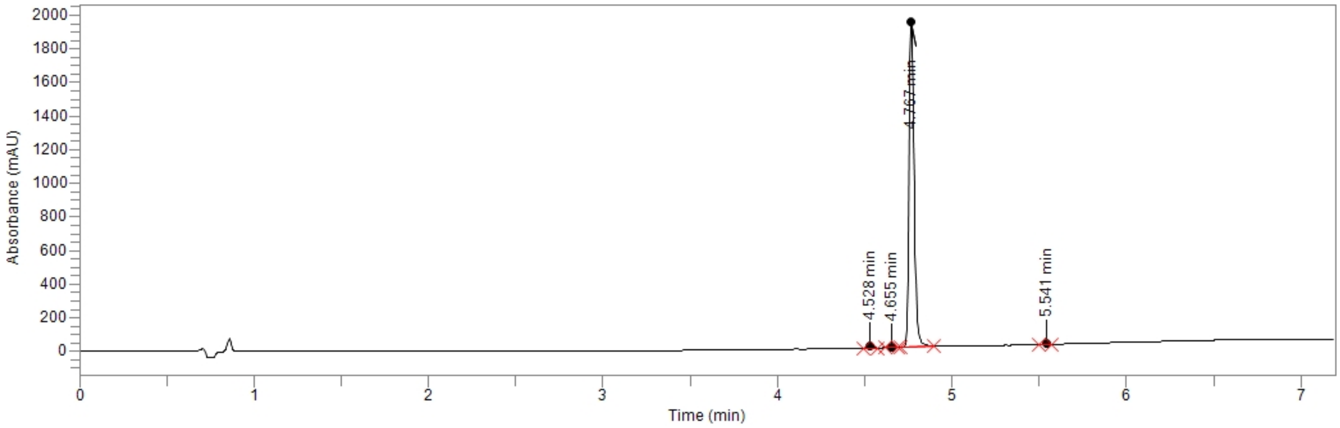

Channel Name **230:10:360:10**

Component Name	Time	Height	Area	Area %	Peak Purity	Wavelength Maximum
	4.528	14,075.4	26,409.9	0.67	1.22Pass	223
	4.655	1,924.7	4,025.2	0.10	1.27Pass	225
	4.767	1,938,704.0	3,925,891.4	99.06	1.17Pass	224
	5.541	3,858.7	6,785.2	0.17	1.18Pass	227
Total			3,963,111.7	100.00		

SS1602A : 266:10:360:10 : 1

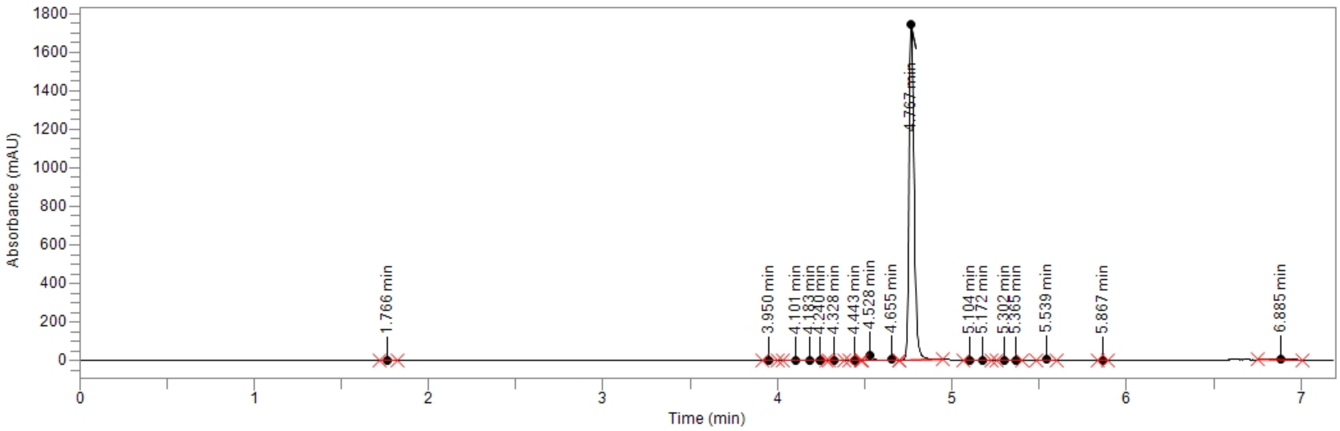

Channel Name **266:10:360:10**

Component Name	Time	Height	Area	Area %	Peak Purity	Wavelength Maximum
	1.766	820.7	2,204.3	0.06	6.15Fail	196
	3.950	691.5	1,658.7	0.05	1.40Pass	223
	4.101	2,132.9	6,685.1	0.18	1.51Fail	223
	4.183	662.3	1,375.5	0.04	1.30Pass	224
	4.240	626.9	1,376.7	0.04	1.42Pass	224
	4.328	2,787.3	5,638.9	0.15	1.39Pass	224
	4.443	958.8	1,747.2	0.05	1.33Pass	225
	4.528	25,407.3	50,598.5	1.39	1.40Pass	223
	4.655	2,139.5	4,911.9	0.14	1.21Pass	225
	4.767	1,743,683.9	3,529,806.4	97.01	1.17Pass	224
	5.104	831.8	1,874.0	0.05	1.28Pass	225
	5.172	1,073.3	2,623.4	0.07	1.20Pass	225
	5.302	1,374.5	2,937.7	0.08	1.20Pass	225
	5.365	970.2	1,963.6	0.05	1.16Pass	225
	5.539	3,225.4	6,643.7	0.18	1.17Pass	227
	5.867	320.0	587.8	0.02	1.12Pass	227
	6.885	2,233.3	15,801.9	0.43	1.06Pass	228
Total			3,638,435.4	100.00		

Ethyl 2-((tert-butoxycarbonyl)oxy)pyrazolo[1,5-a]pyridine-3-carboxylate (**40**)

^{1}H NMR Spectrum (CDCl$_3$)

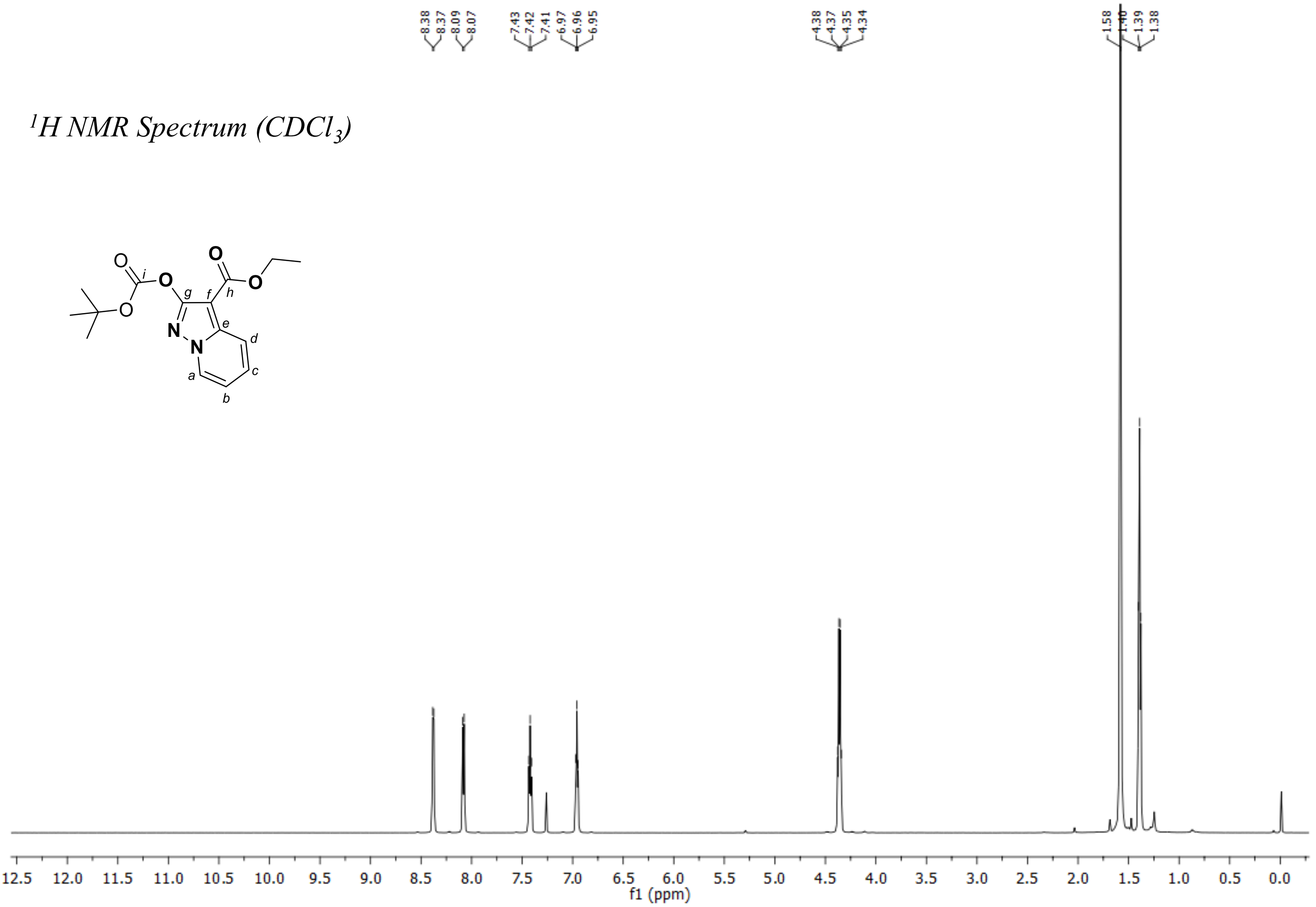

^{13}C NMR Spectrum (CDCl$_3$)

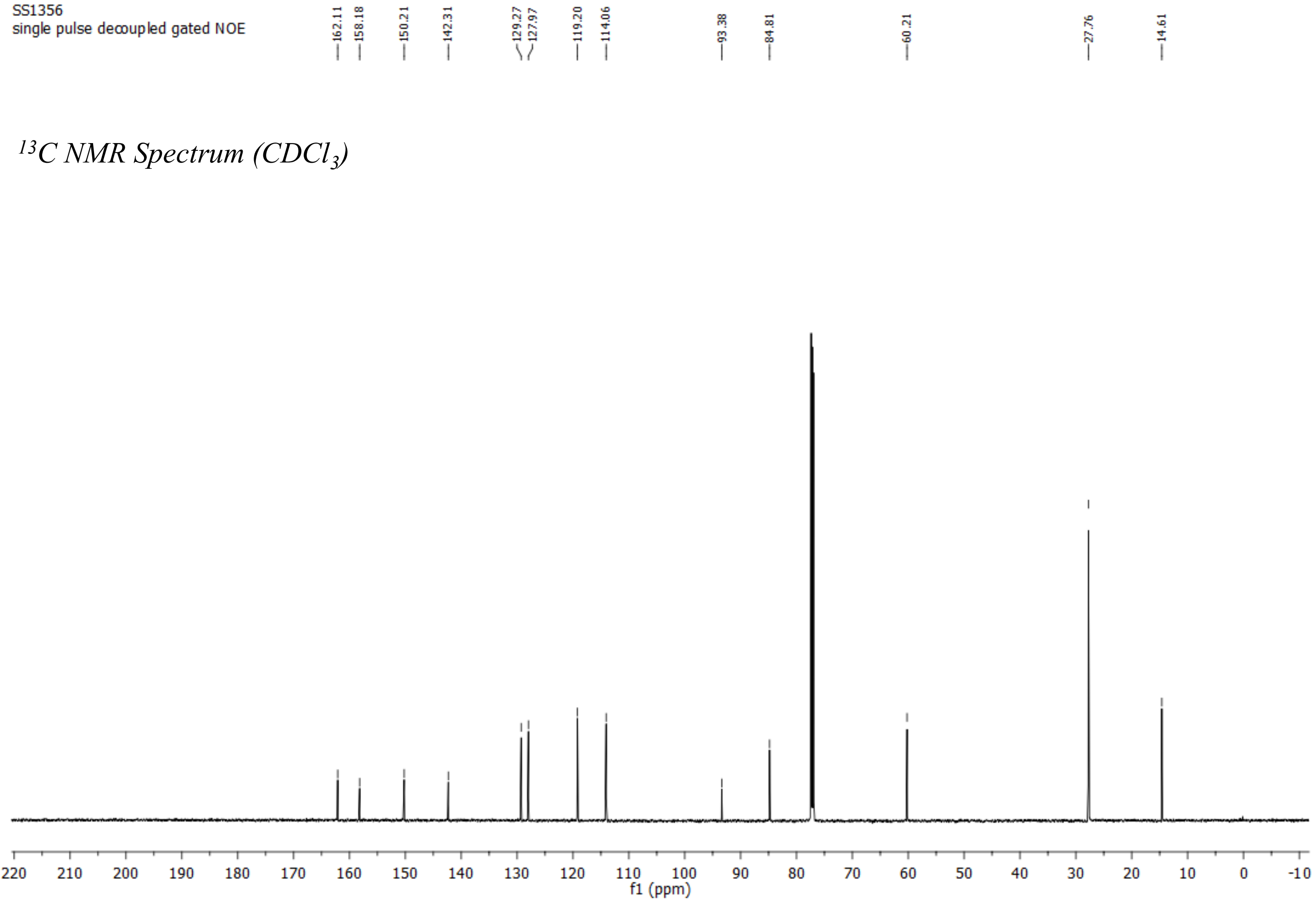

Ethyl 2-((tert-butoxycarbonyl)oxy)-7-chloropyrazolo[1,5-a]pyridine-3-carboxylate (**41**)

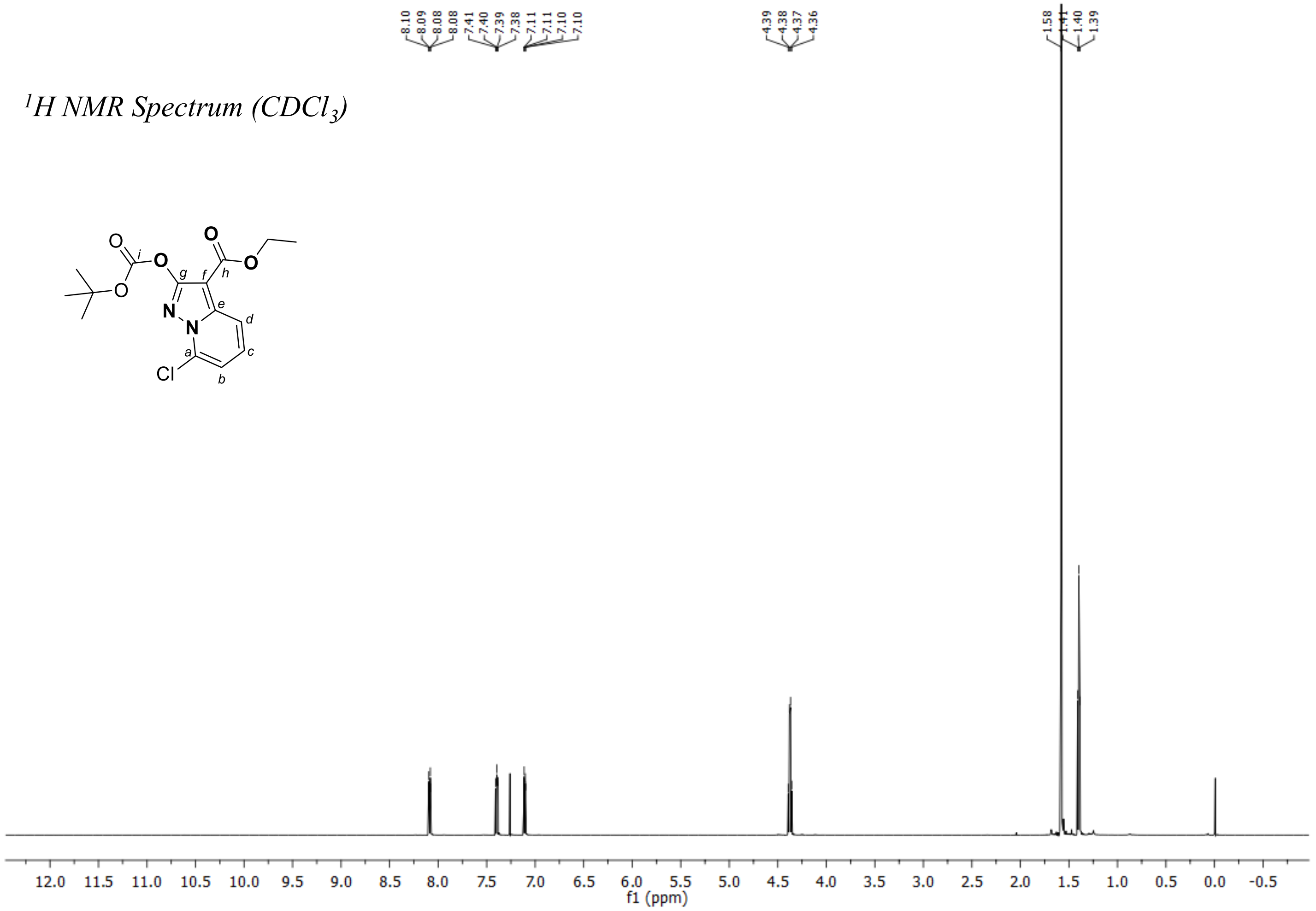

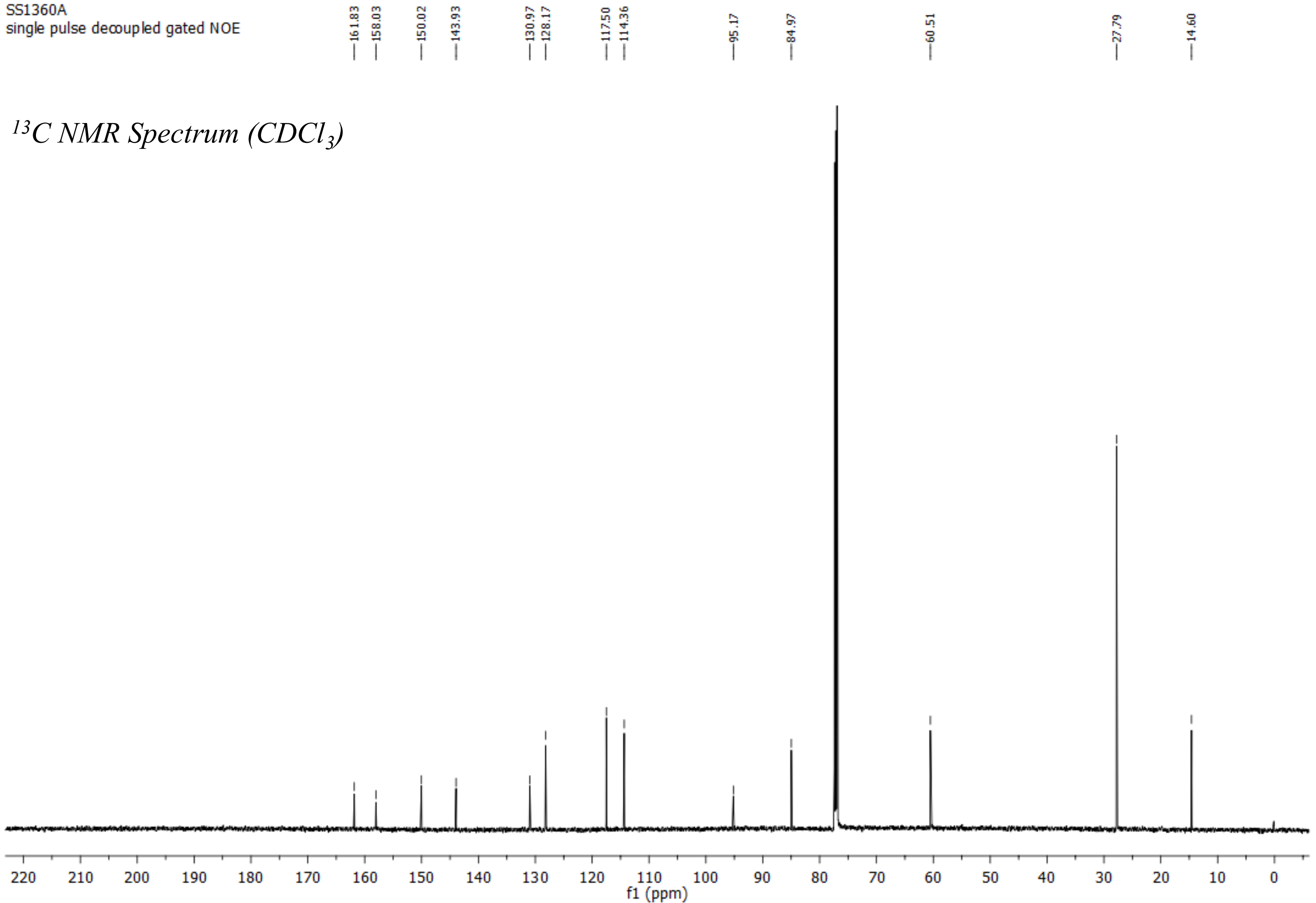

Ethyl 7-chloro-2-hydroxypyrazolo[1,5-a]pyridine-3-carboxylate (**42**)

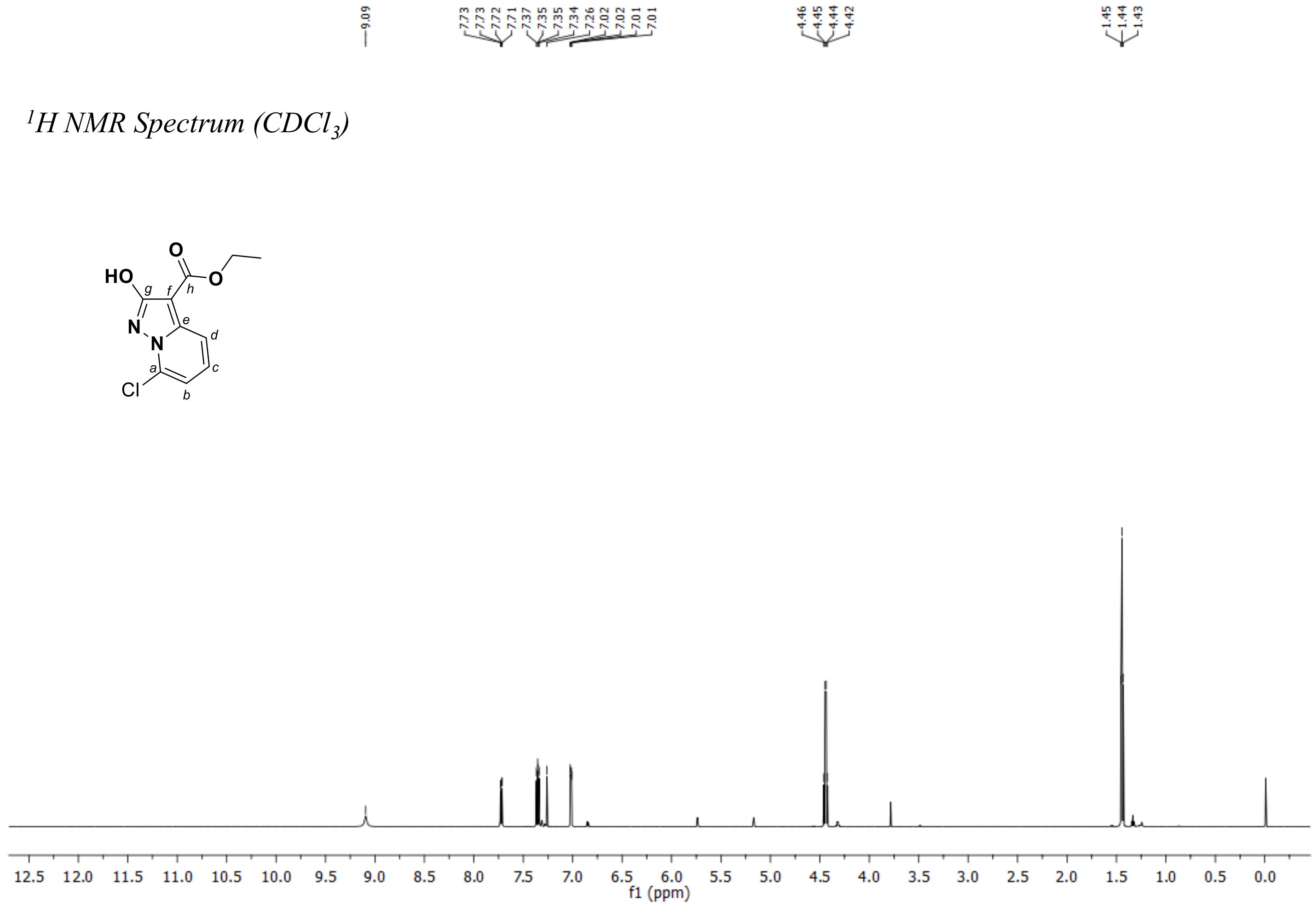

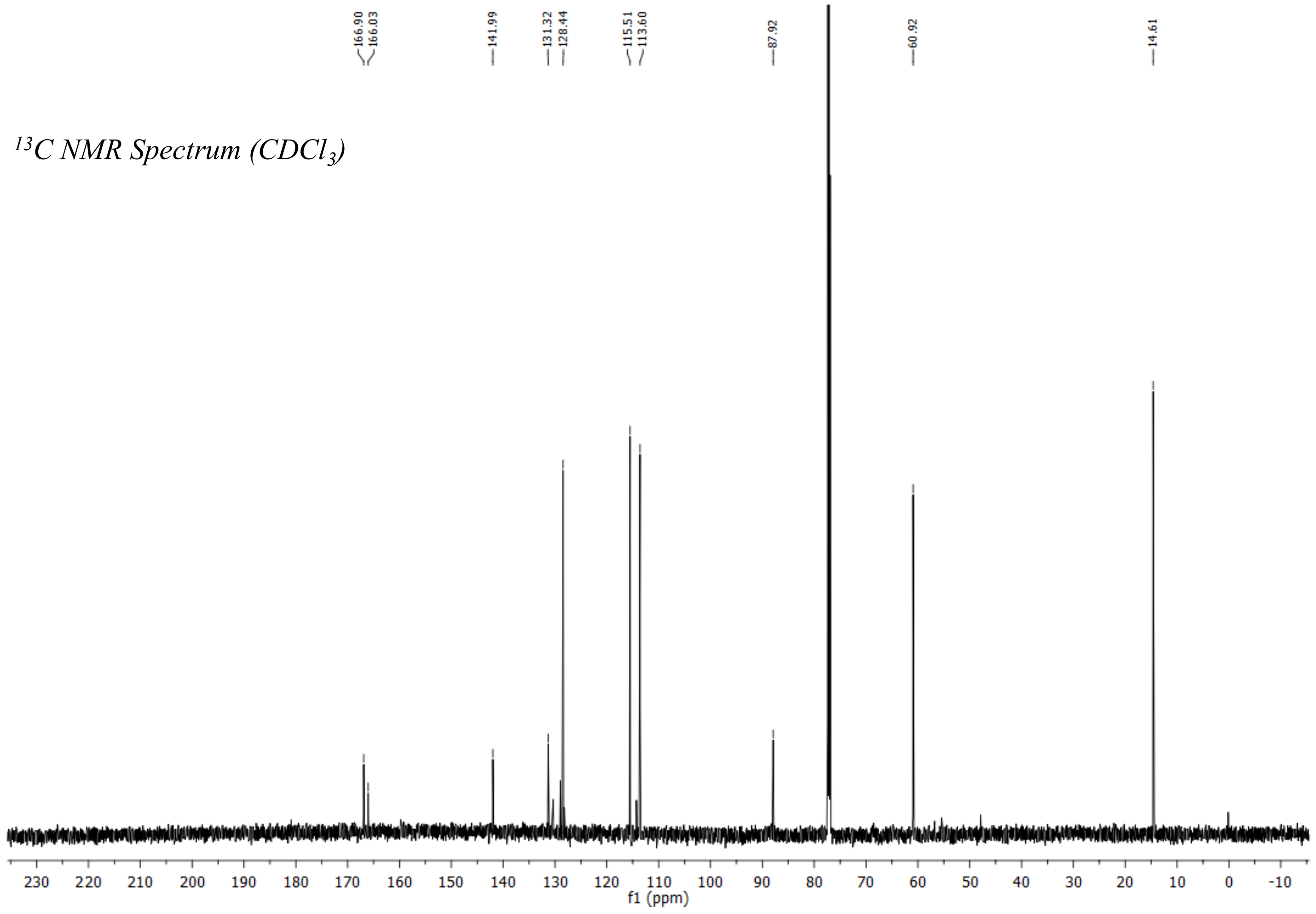

Ethyl 2-(benzyloxy)-7-chloropyrazolo[1,5-a]pyridine-3-carboxylate (**43**)

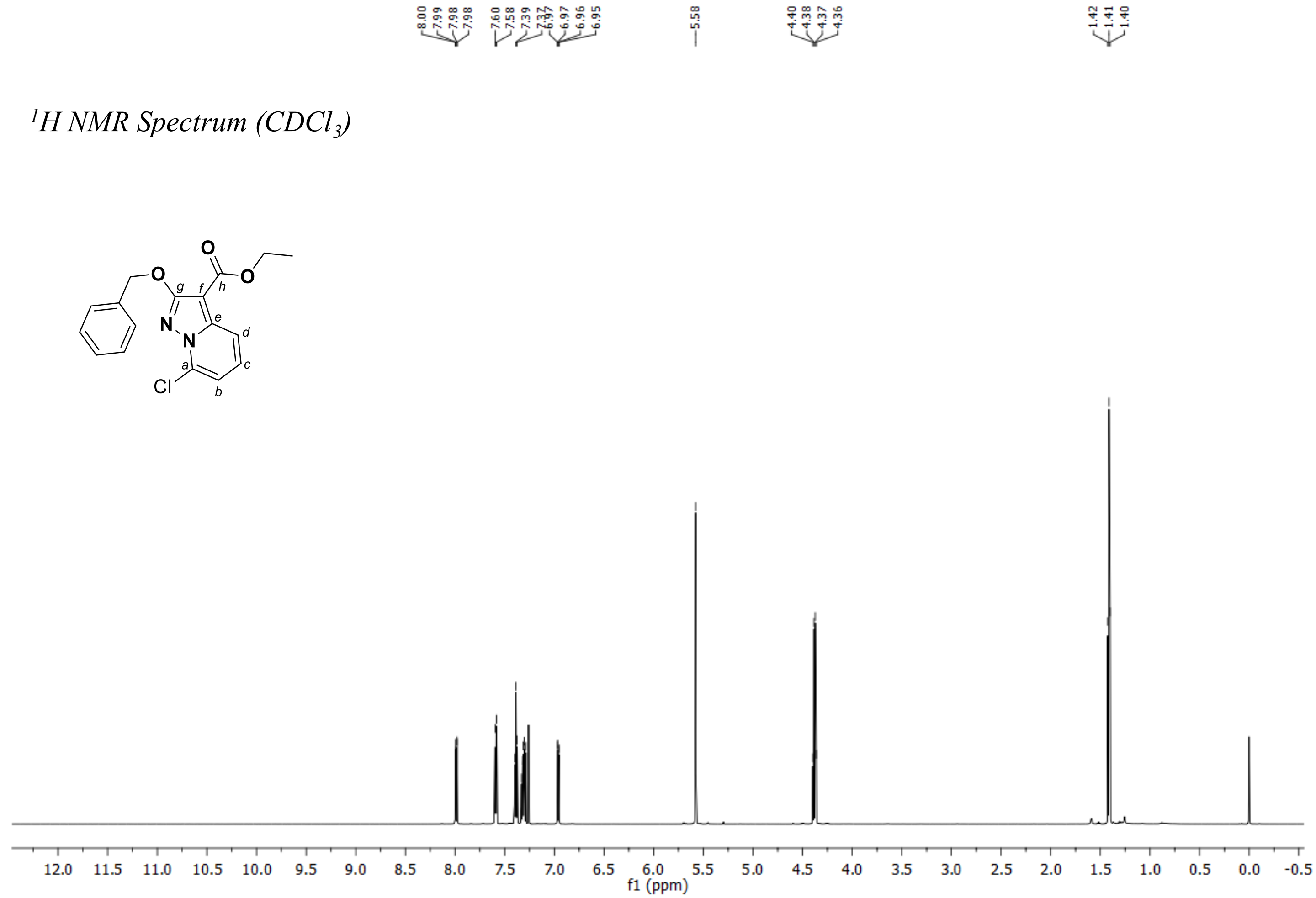

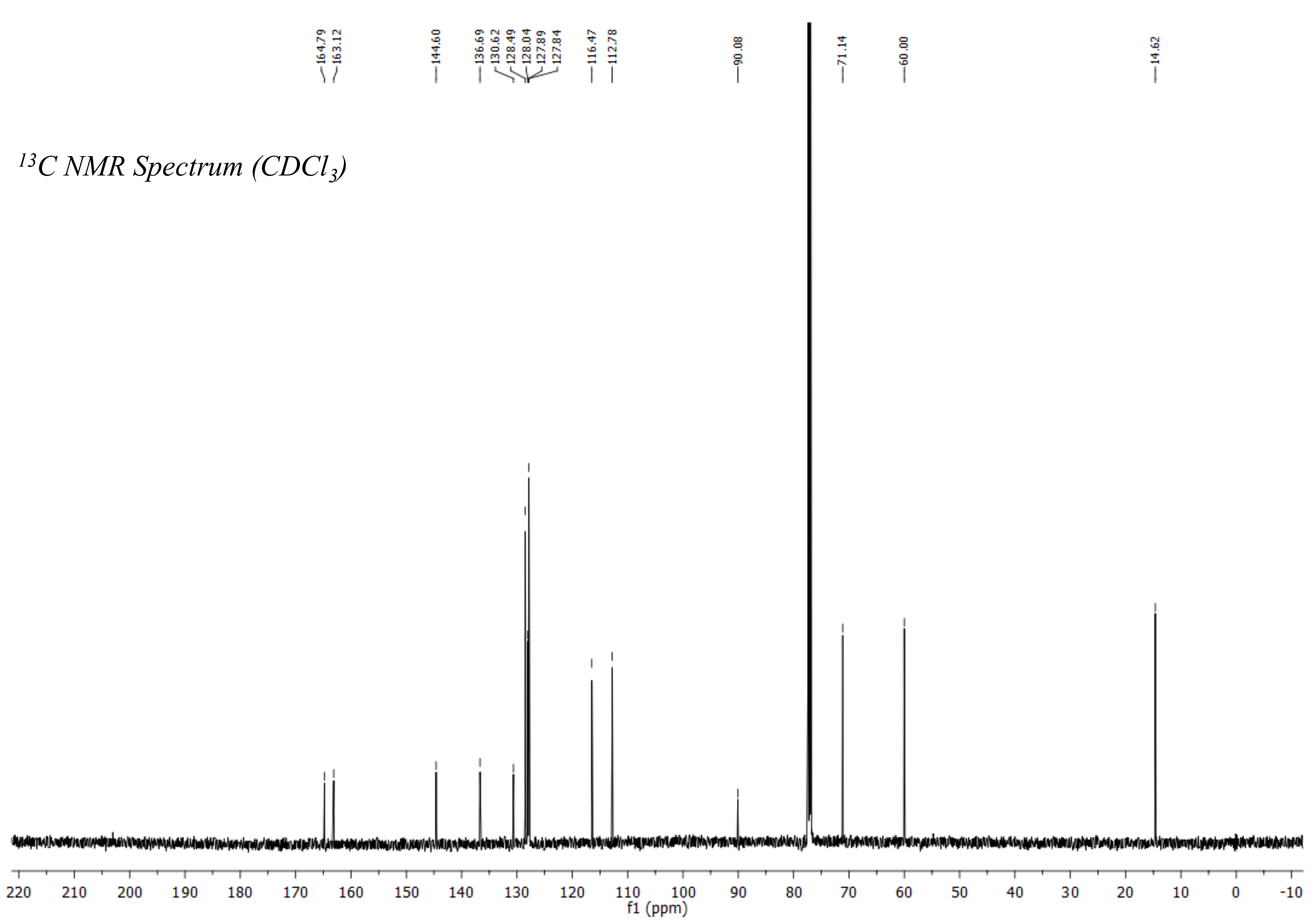

2-(Benzyloxy)-7-chloropyrazolo[1,5-a]pyridine-3-carboxylic acid **(44)**

^{1}H NMR Spectrum (DMSO)

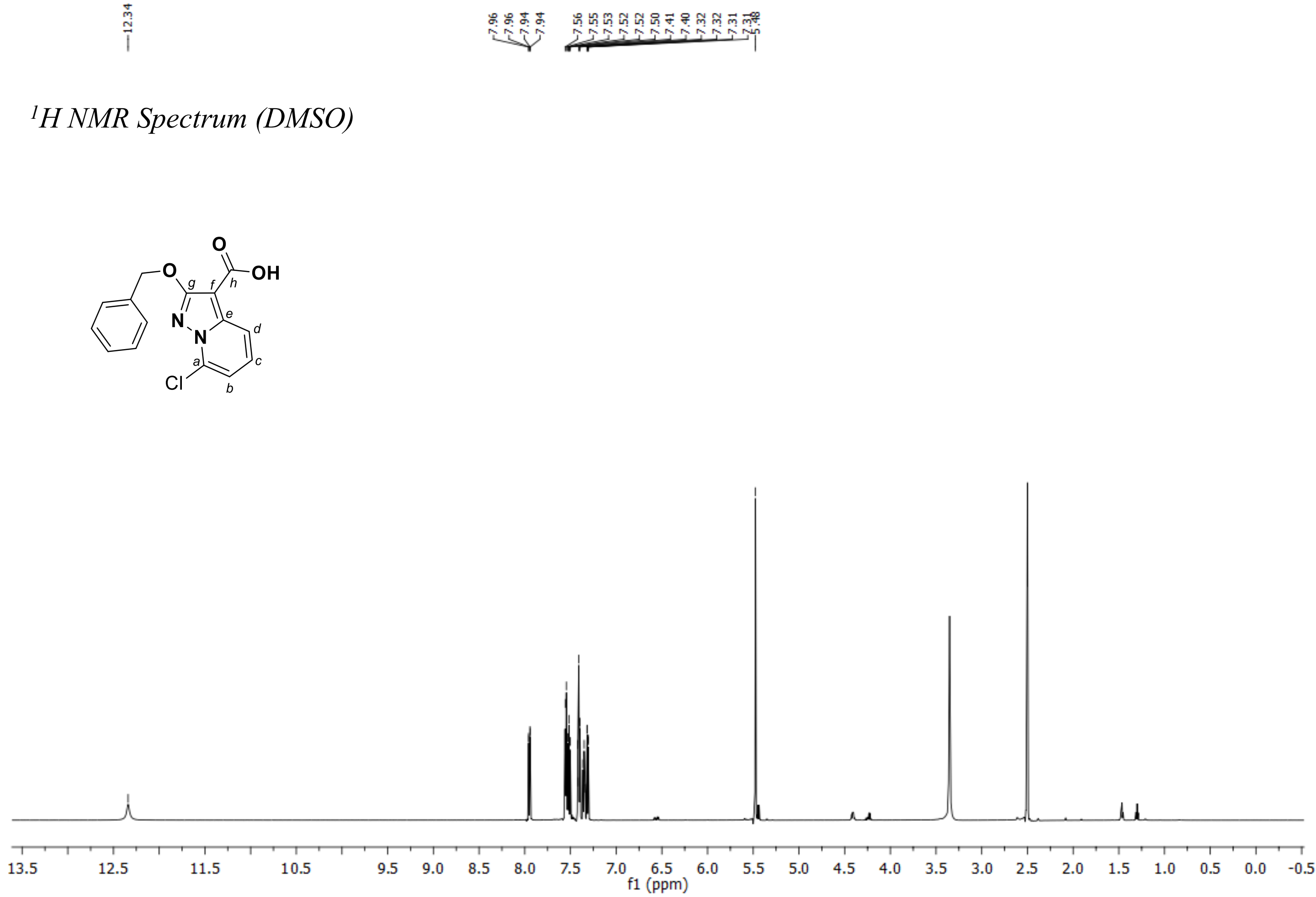

^{13}C NMR Spectrum (DMSO)

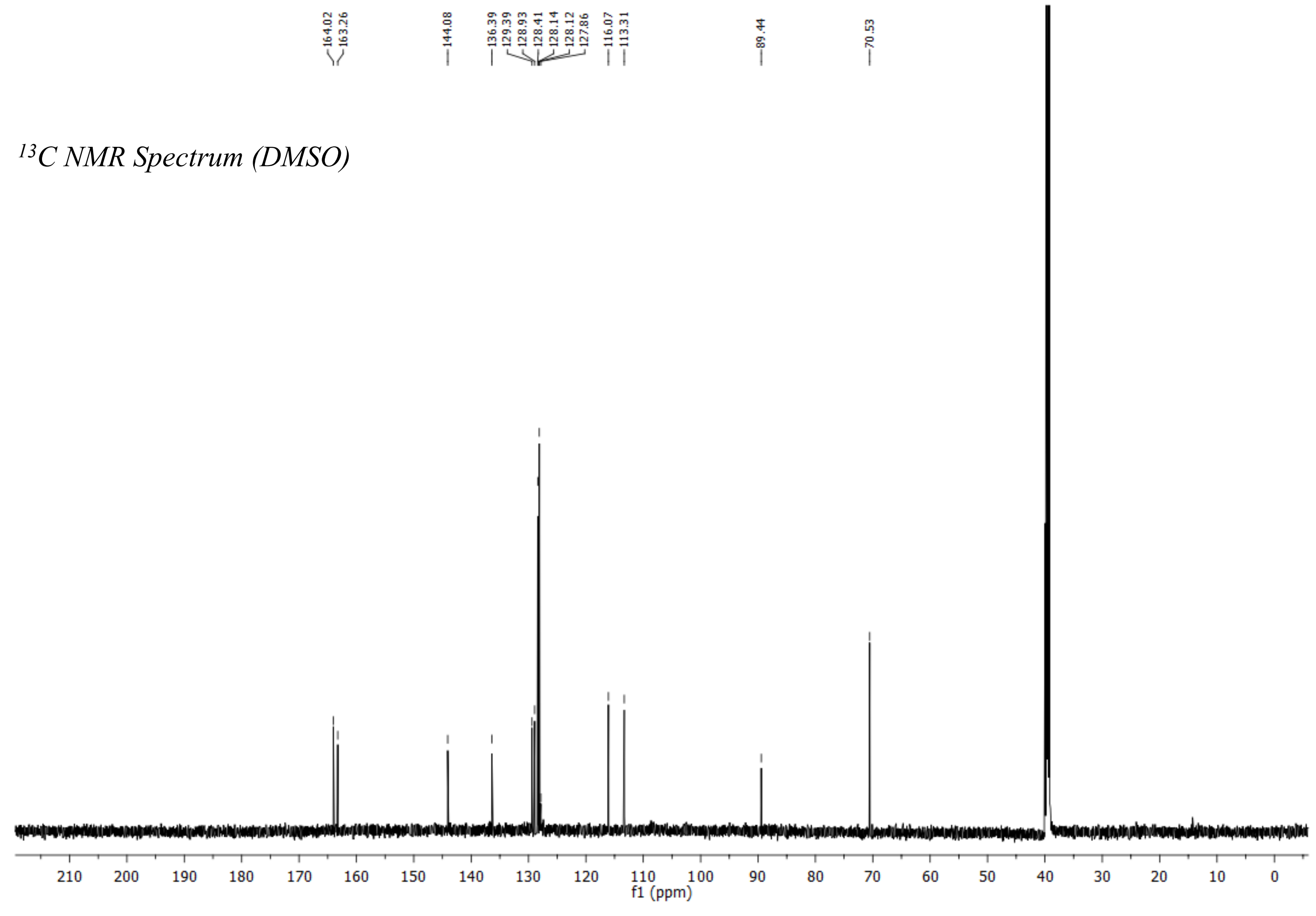

1-Benzyl-7-chloro-2-oxo-N-(2,3,5,6-tetrafluoro-[1,1'-biphenyl]-4-yl)-1,2-dihydropyrazolo[1,5-a]pyridine-3-carboxamide (**45**)

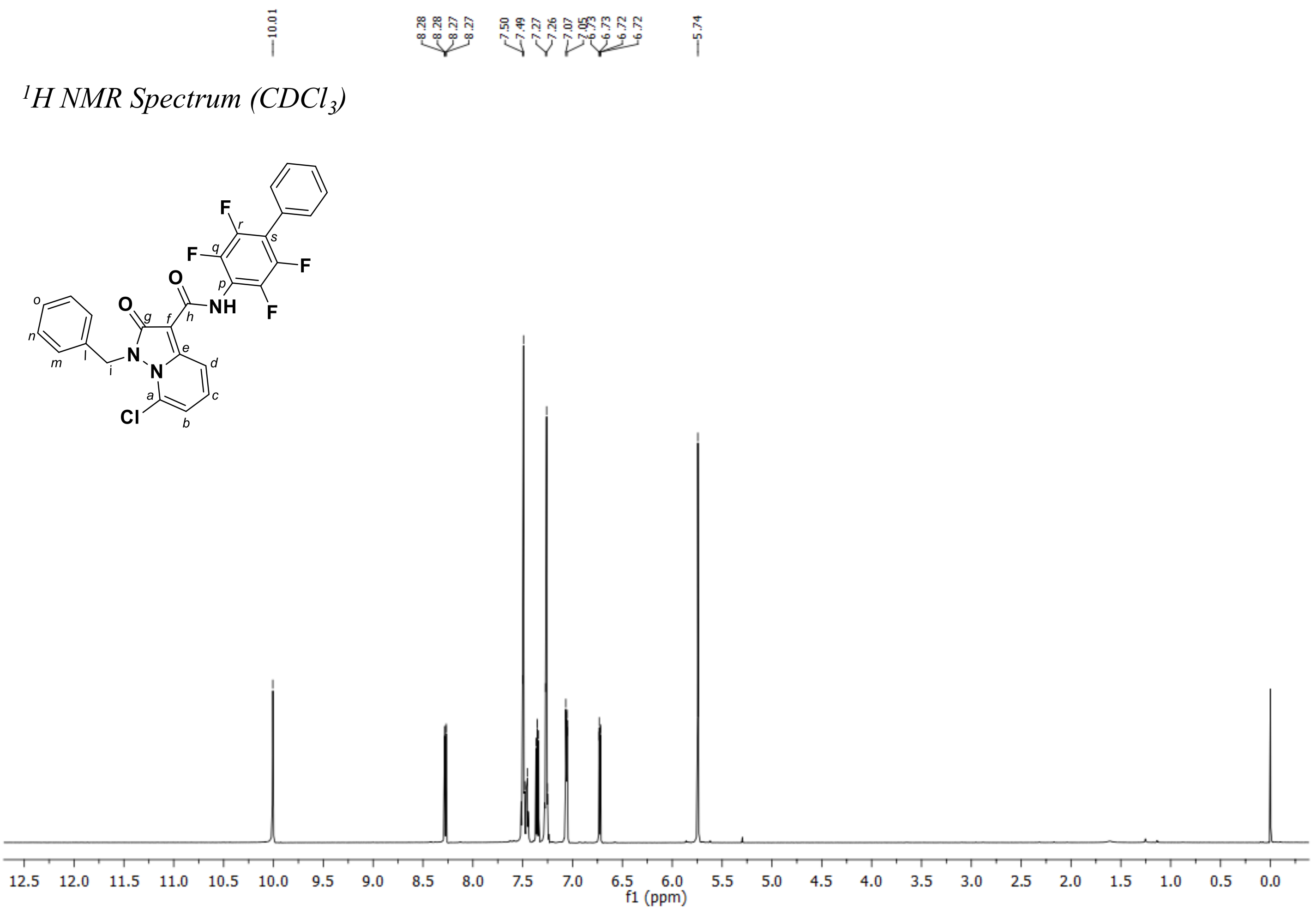

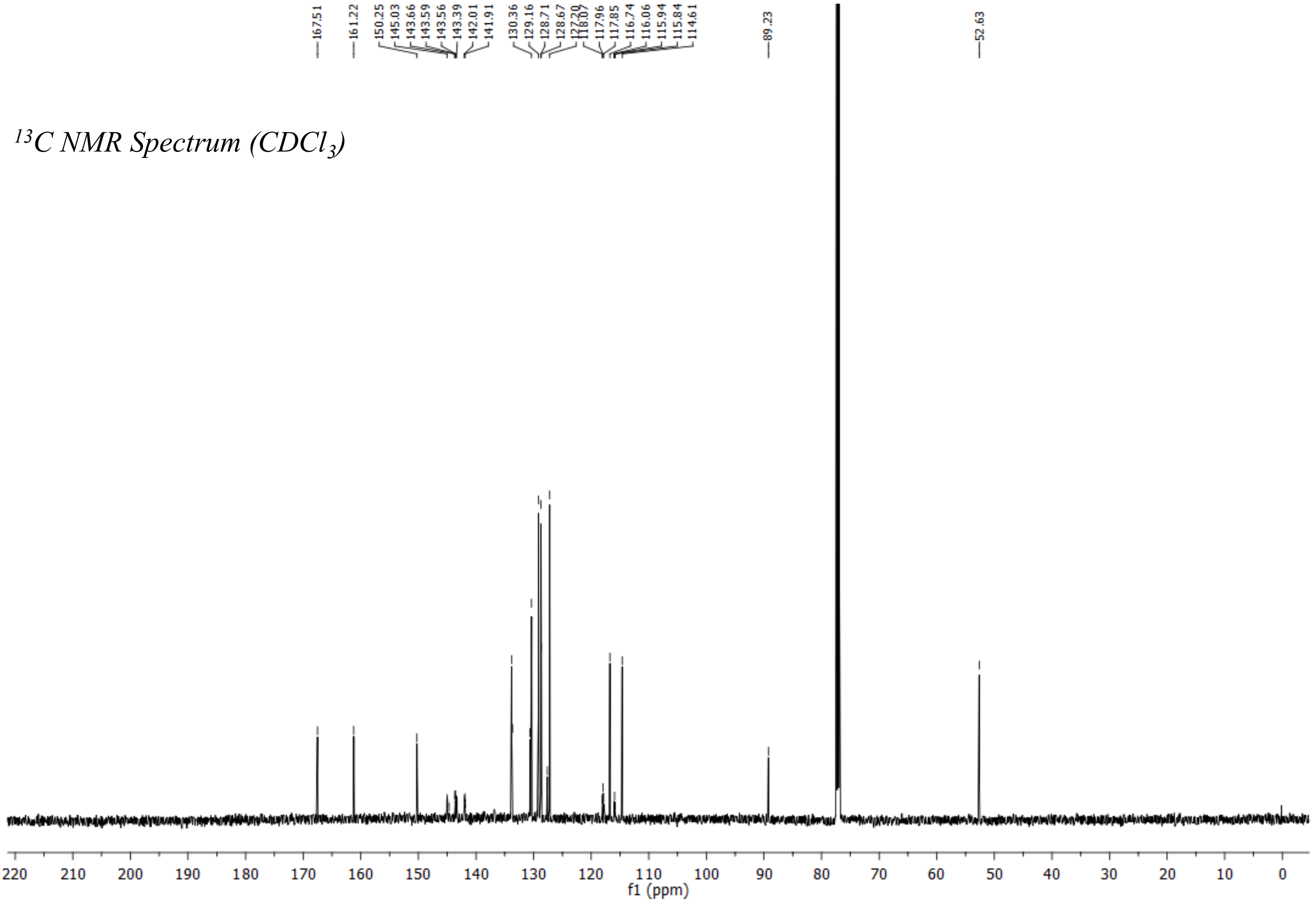

7-Chloro-2-hydroxy-N-(2,3,5,6-tetrafluoro-[1,1'-biphenyl]-4-yl)pyrazolo[1,5-a]pyridine-3-carboxamide (**46**)

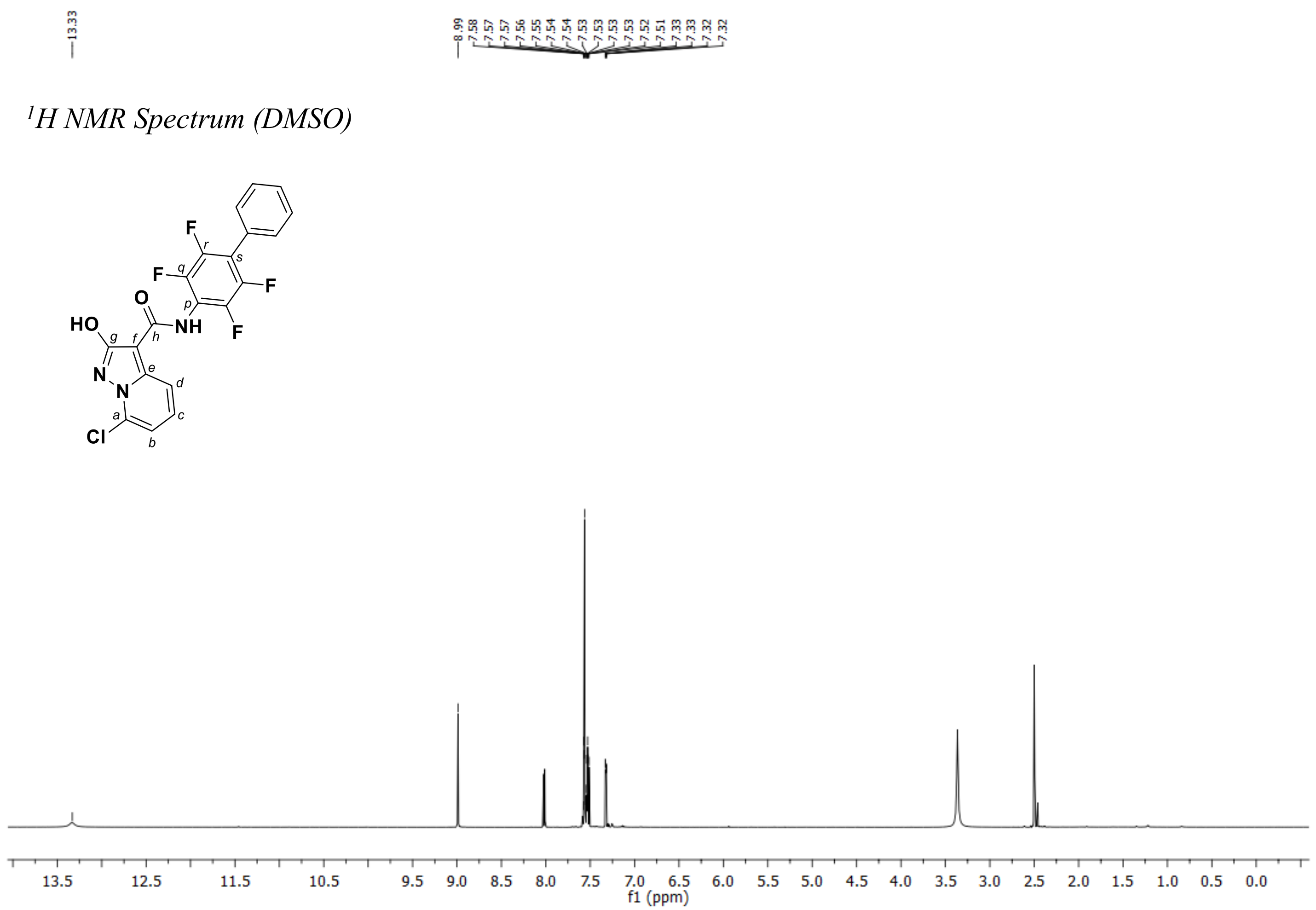

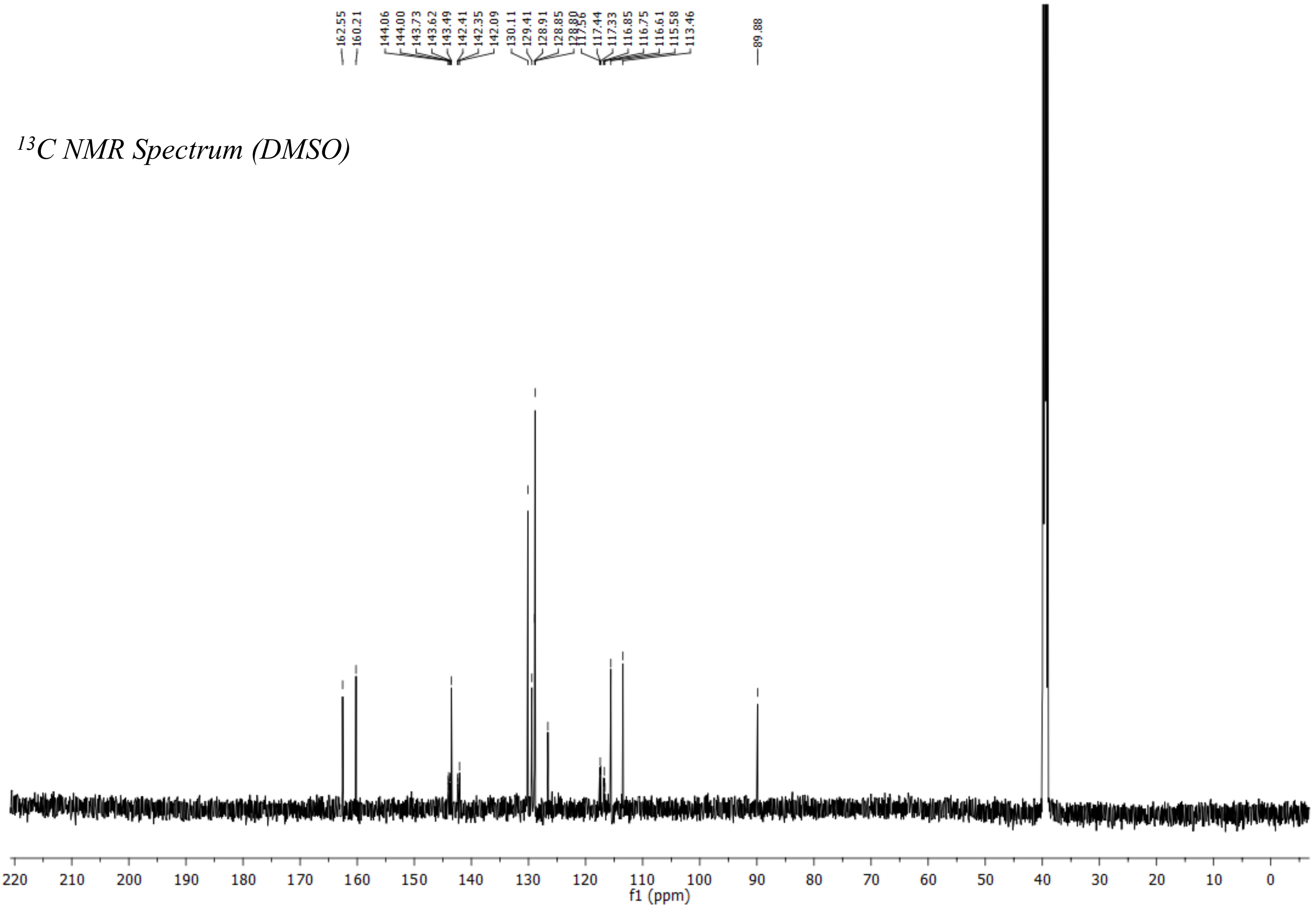

7-Chloro-2-hydroxy-N-(2,3,5,6-tetrafluoro-[1,1'-biphenyl]-4-yl)pyrazolo[1,5-a]pyridine-3-carboxamide (**46**)

meds-611__10ng-ul_inf_ms2-hcd-30-pos 01/23/20 14:02:07 MEDS-611__10ng-ul_inf_MS2-HCD-30-pos

meds-611 10ng-ul inf ms-pos #1-20 RT: 0.01-0.29 AV: 20 NL: 2.67E5
T: FTMS + p ESI Full ms [430.00-440.00]

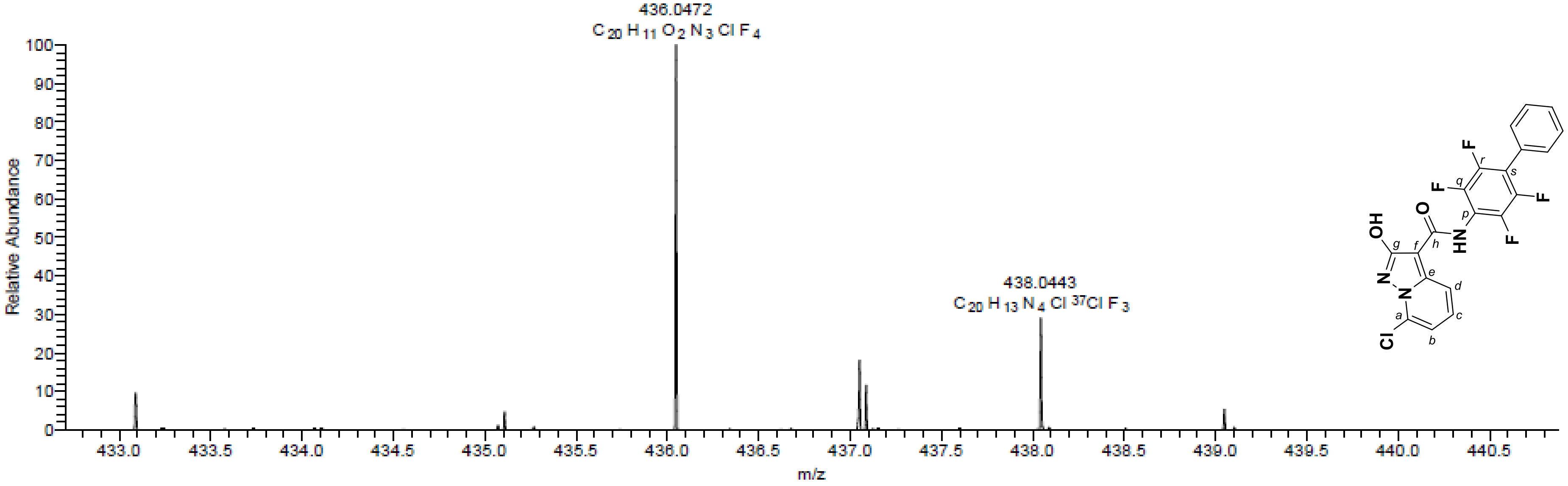

meds-611__10ng-ul_inf_ms2-hcd-30-pos #1-20 RT: 0.01-0.40 AV: 20 NL: 1.46E5
T: FTMS + p ESI Full ms2 436.00@hcd30.00 [50.00-460.00]

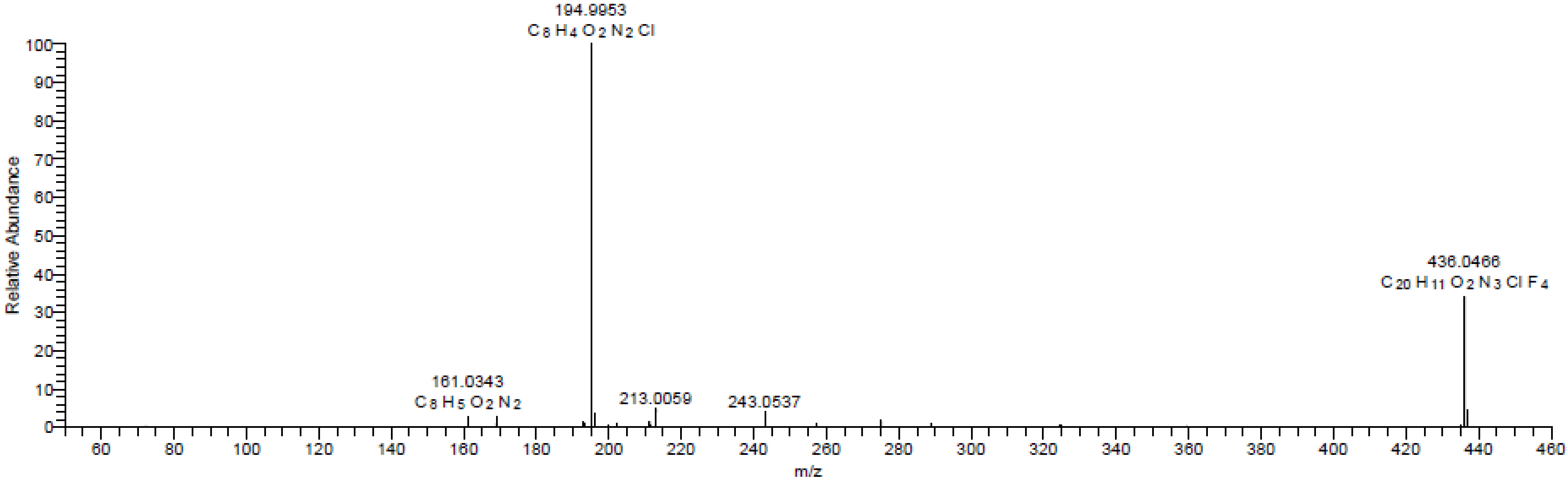

Supplement: Supplementary file 2 — jm0c01549_si_002.pdf [file jm0c01549_si_002.pdf]
